# Supplementary material for: Quantitative Data-Independent Acquisition Glycoproteomics of Sparkling Wine
Source: Mol Cell Proteomics. 2020 Dec 21;20:100020. doi: 10.1074/mcp.RA120.002181 (PMC8724608; doi:10.1074/mcp.RA120.002181)
Supplement: MCPRO_MCP-2020-002181_1 [file mmc1.pdf]

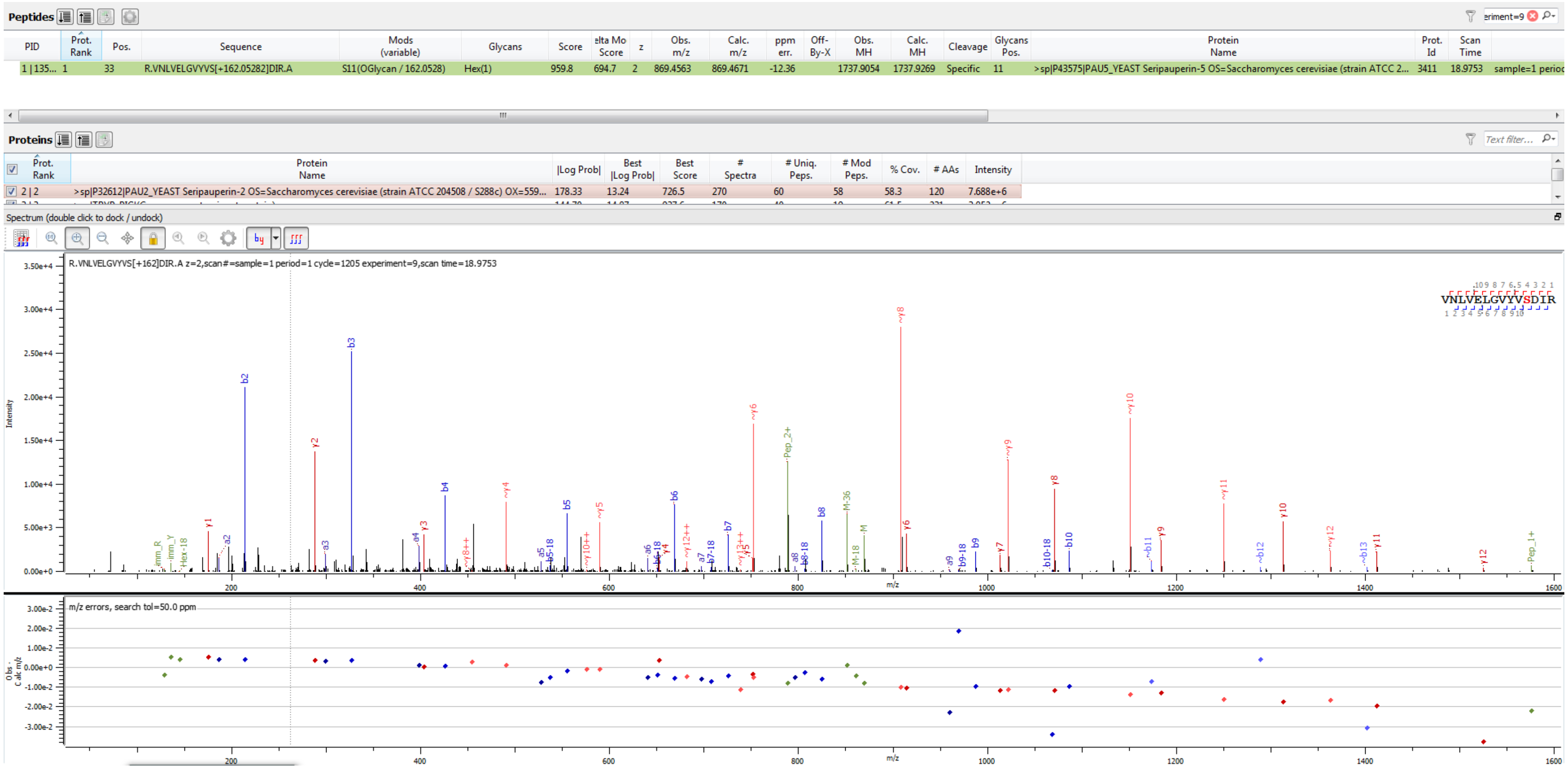

Sample S2 R.VNLVELGVYVS[+162.053]DIR.A

Peptides

eriment=3

PID	Prot. Rank	Pos.	Sequence	Mods (variable)	Glycans	Score	elta Mo Score	z	Obs. m/z	Calc. m/z	ppm err.	Off-By-X	Obs. MH	Calc. MH	Cleavage	Glycans Pos.	Protein Name	Prot. Id	Scan Time	
1 | 139...	1	33	R.VNLVELGVYVS[+162.05282]DIR.A	S11(OGlycan / 162.0528)	Hex(1)	762.5	556.0	3	579.9755	579.9805	-8.58		1737.9120	1737.9269	Specific	11	>sp|P43575|PAU5_YEAST Seripauperin-5 OS=Saccharomyces cerevisiae (strain ATCC 2...	3411	19.3134	sample=1 perioc

Proteins

Text filter...

Prot. Rank	Protein Name	|Log Prob|	Best |Log Prob|	Best Score	# Spectra	# Uniq. Peps.	# Mod Peps.	% Cov.	# AAs	Intensity
2 | 2	>sp|P32612|PAU2_YEAST Seripauperin-2 OS=Saccharomyces cerevisiae (strain ATCC 204508 / S288c) OX=559...	178.33	13.24	726.5	270	60	58	58.3	120	7.688e+6

Spectrum (double click to dock / undock)

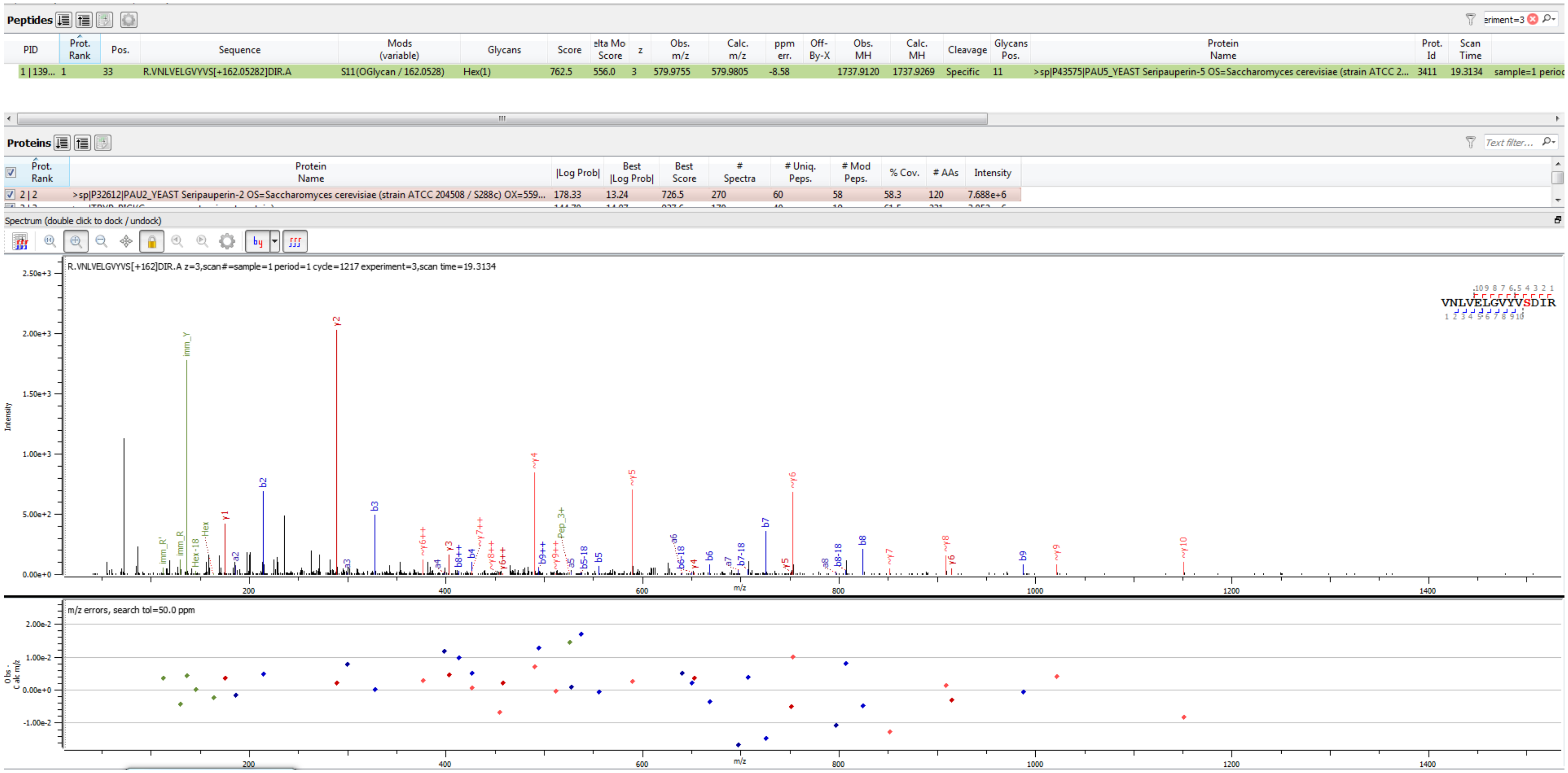

Sample S2 R.VNLVELGVYVS[+162.053]DIR.A

Peptides

PID	Prot. Rank	Pos.	Sequence	Mods (variable)	Glycans	Score	elta Mo Score	z	Obs. m/z	Calc. m/z	ppm err.	Off-By-X	Obs. MH	Calc. MH	Cleavage	Glycans Pos.	Protein Name	Prot. Id	Scan Time	
1 | 135...	1	33	R.VNLVELGVYVS[+324.10565]DIR.A	S11(OGlycan / 324.1056)	Hex(2)	1004.1	667.8	2	950.4798	950.4935	-14.42		1899.9523	1899.9797	Specific	11	>sp|P43575|PAU5_YEAST Seripauperin-5 OS=Saccharomyces cerevisiae (strain ATCC 2...	3411	18.8978	sample=1 perioc

Proteins

Prot. Rank	Protein Name	|Log Prob|	Best |Log Prob|	Best Score	# Spectra	# Uniq. Peps.	# Mod Peps.	% Cov.	# AAs	Intensity
2 | 2	>sp|P32612|PAU2_YEAST Seripauperin-2 OS=Saccharomyces cerevisiae (strain ATCC 204508 / S288c) OX=559...	178.33	13.24	726.5	270	60	58	58.3	120	7.688e+6

Spectrum (double click to dock / undock)

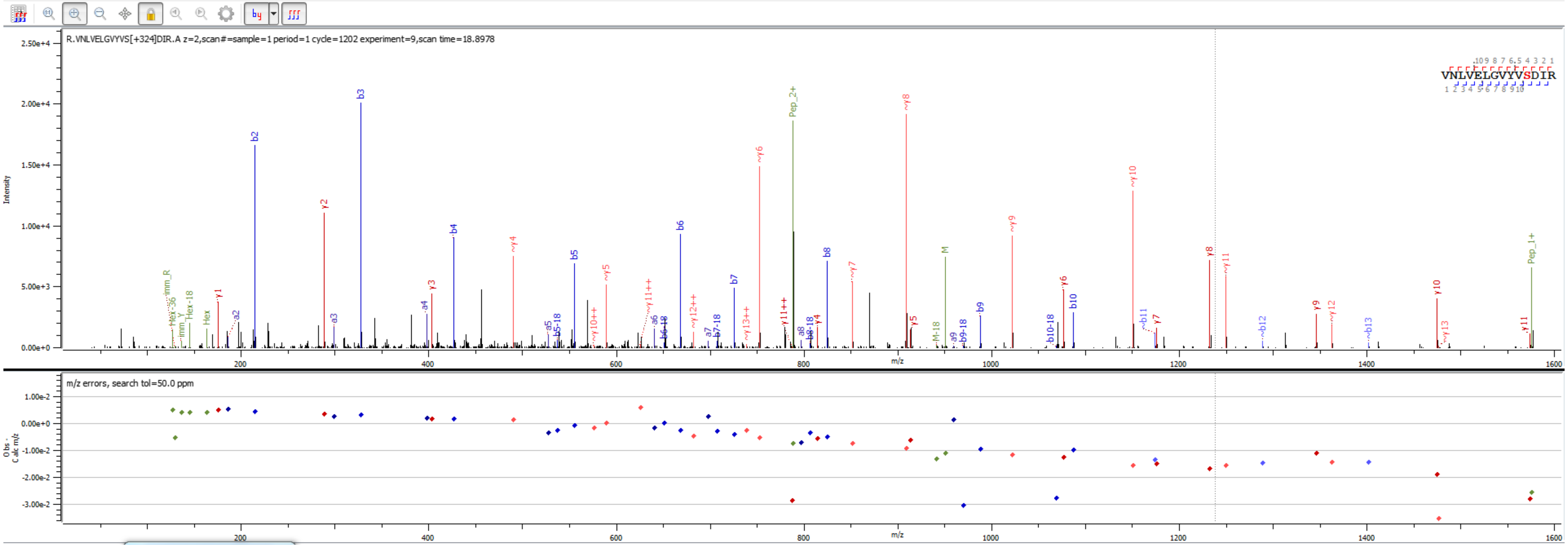

Sample S1 R.VNLVELGVYVS[+324.106]DIR.A

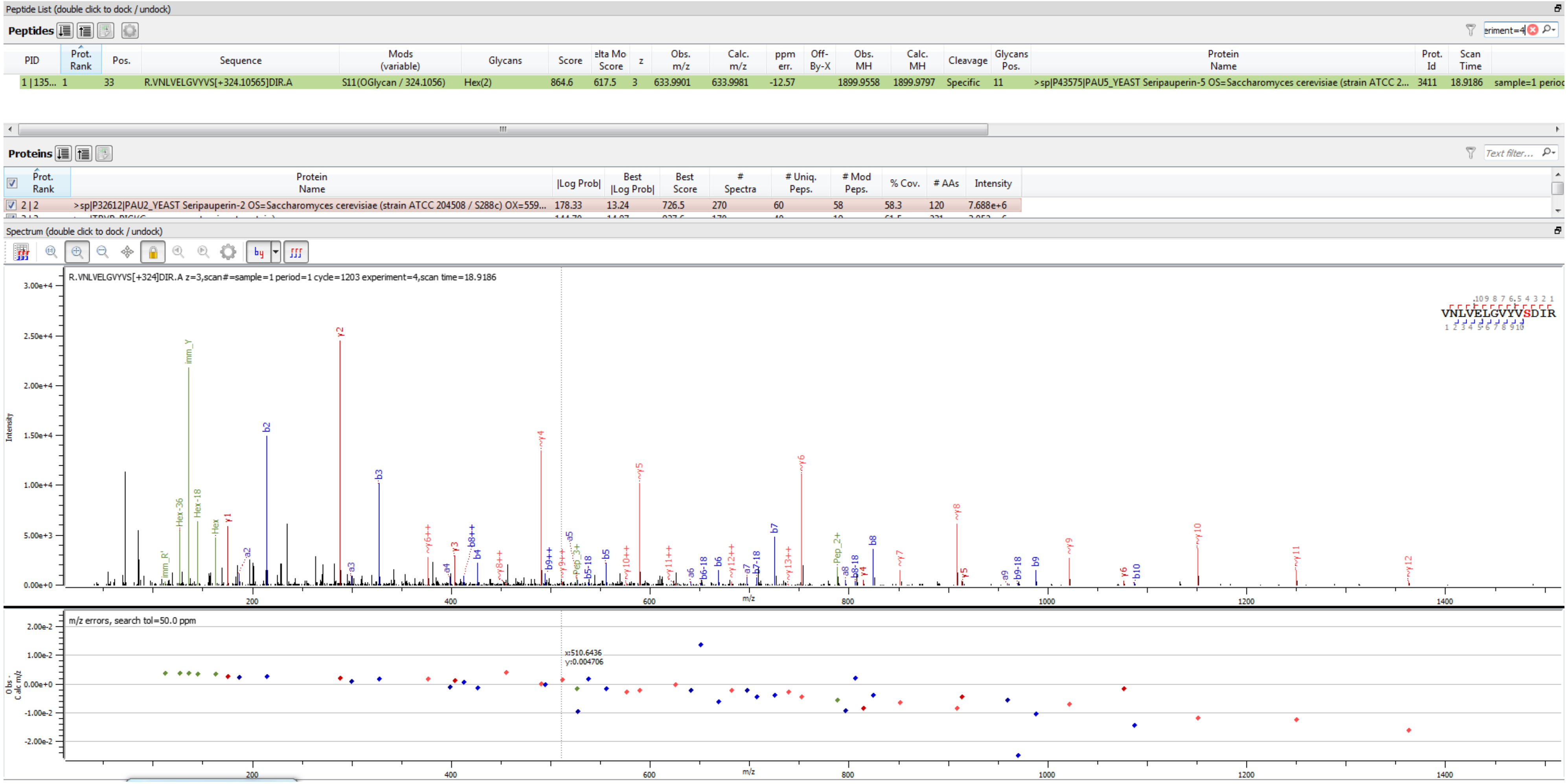

Sample S1 R.VNLVELGVYVS[+324.106]DIR.A

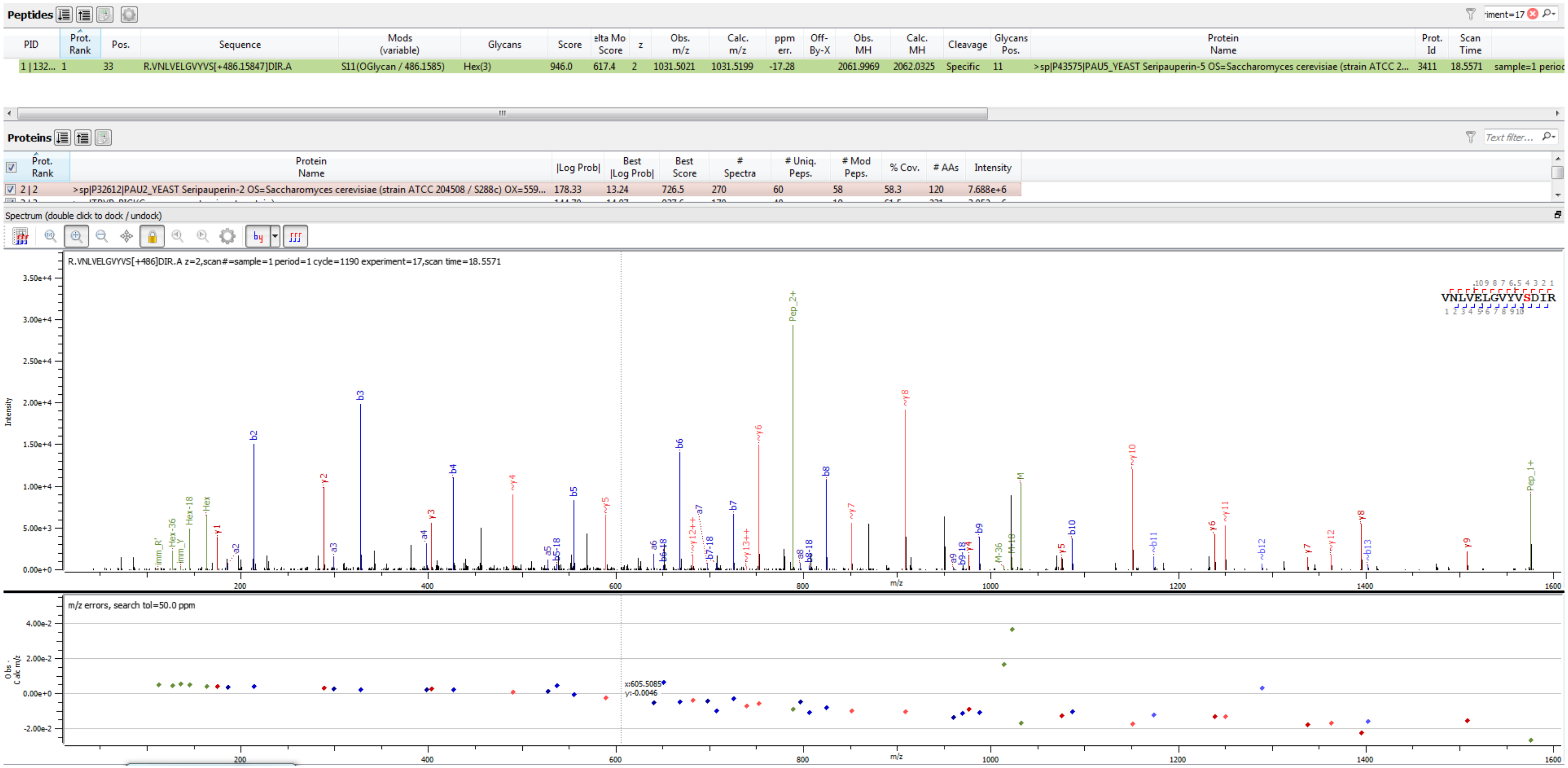

Sample S1 R.VNLVELGVYVS[+486.158]DIR.A

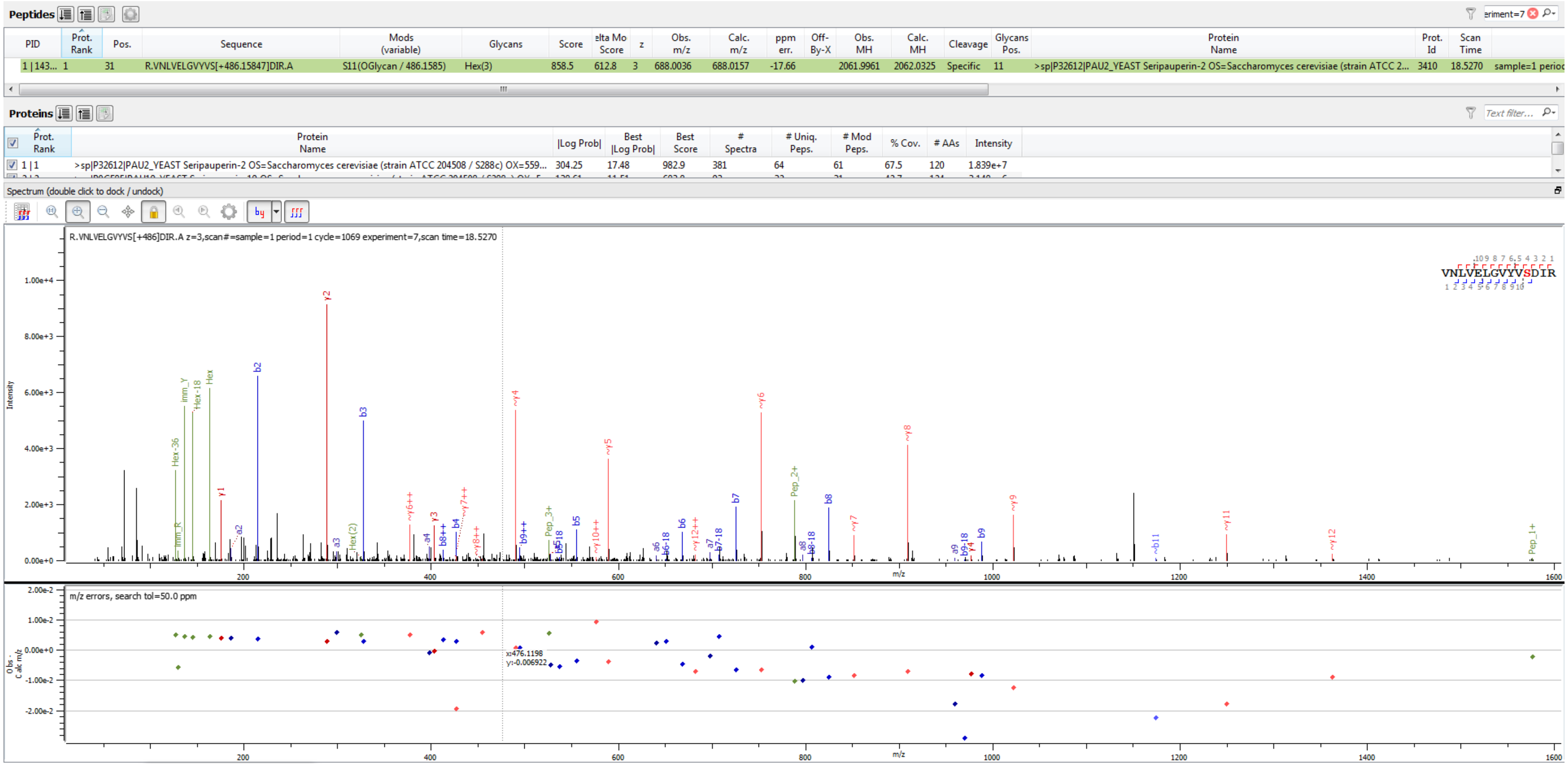

Sample S4 R.VNLVELGVYVS[+486.158]DIR.A

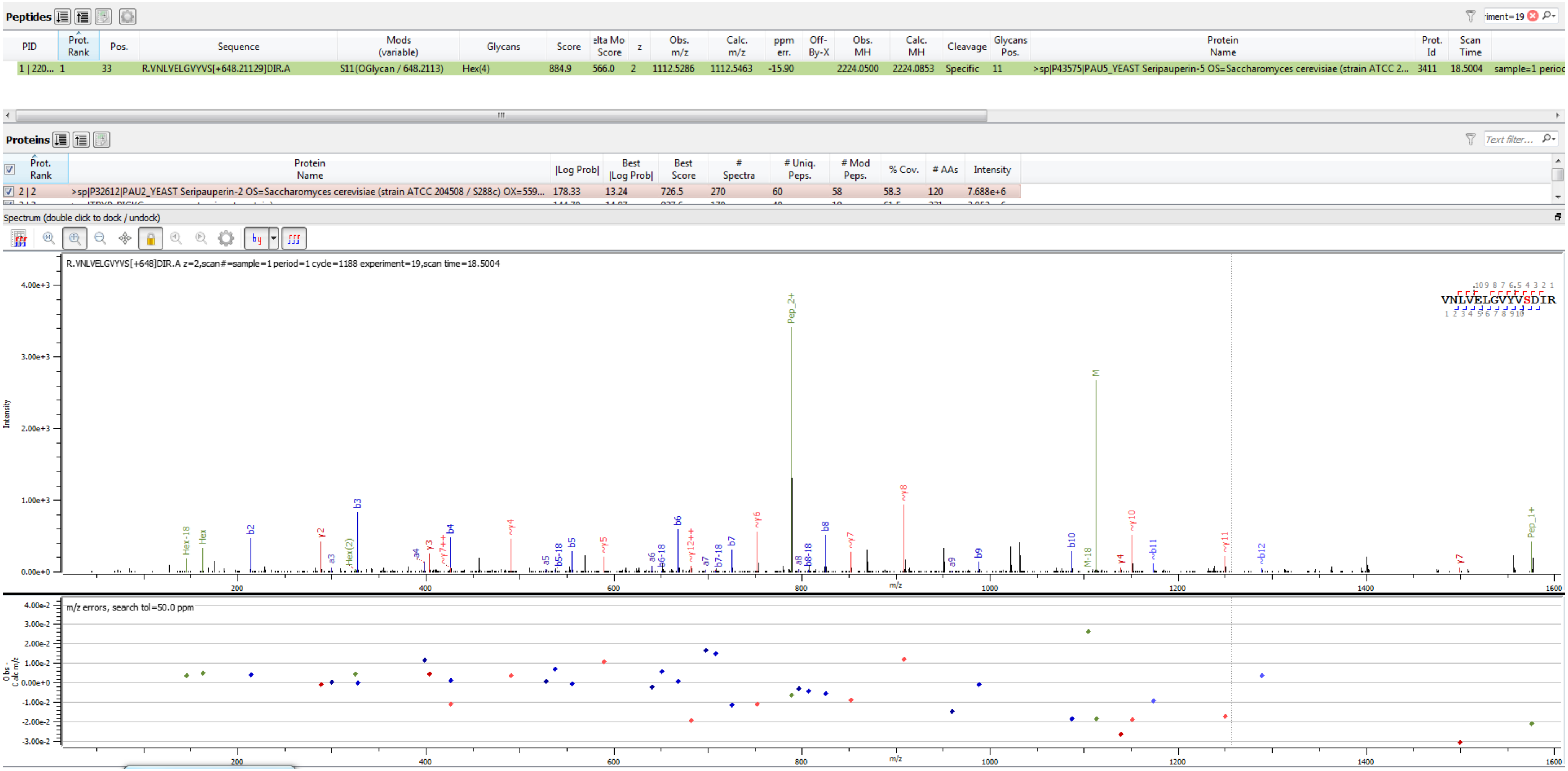

Sample S1 R.VNLVELGVYVS[+648.211]DIR.A

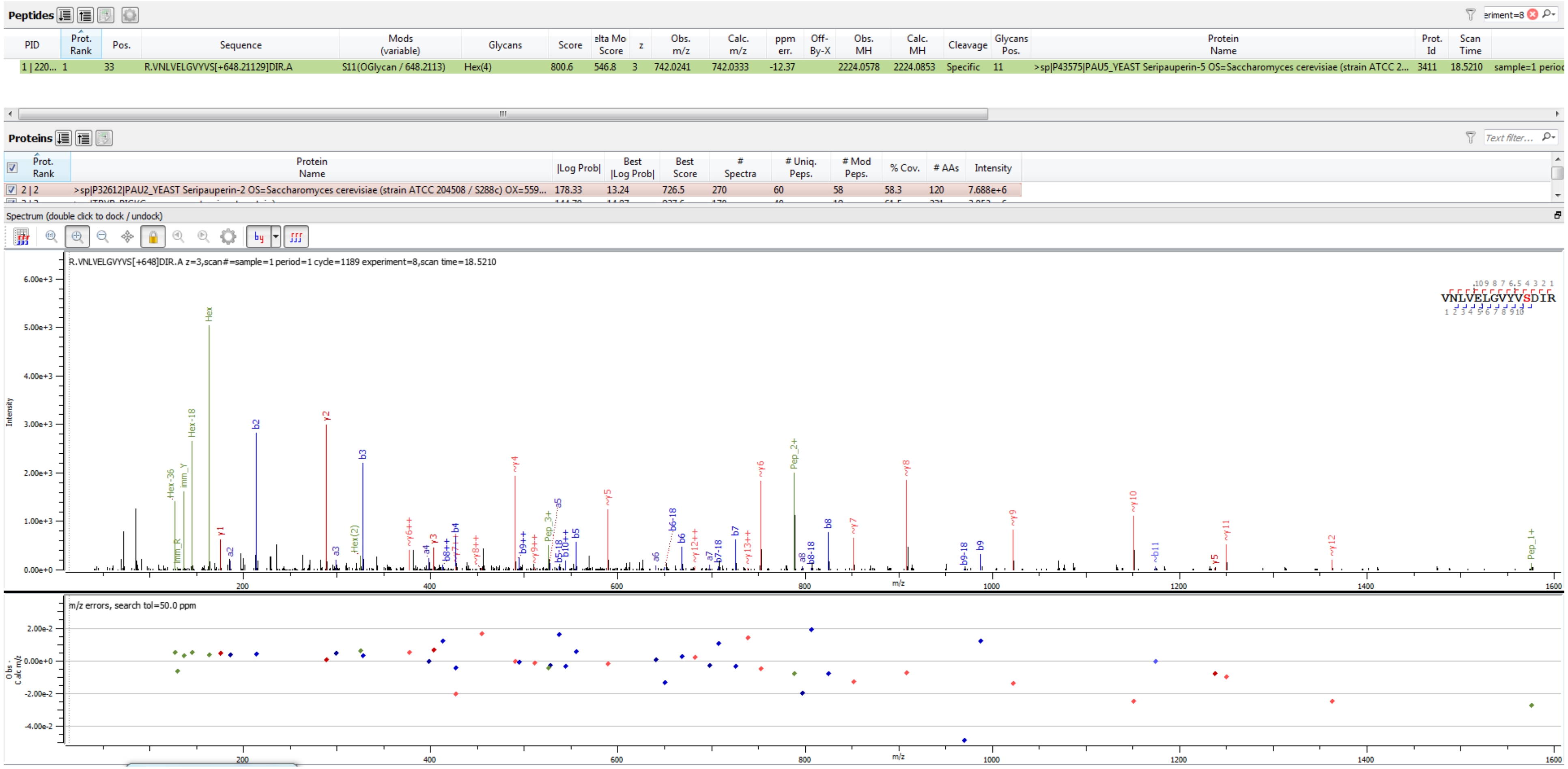

Sample S1 R.VNLVELGVYVS[+648.211]DIR.A

Peptides

PID	Prot. Rank	Pos.	Sequence	Mods (variable)	Glycans	Score	elta Mo Score	z	Obs. m/z	Calc. m/z	ppm err.	Off-By-X	Obs. MH	Calc. MH	Cleavage	Glycans Pos.	Protein Name	Prot. Id	Scan Time	
1 | 210...	1	29	D.ERVNLVELGVYVS[+324.10565]DIR.A	S13(OGlycan / 324.1056)	Hex(2)	902.7	670.1	3	729.0343	729.0460	-16.03		2185.0884	2185.1234	NRagged	13	>sp|Q3E770|PAU9_YEAST Seripauperin-9 OS=Saccharomyces cerevisiae (strain ATCC 2...	3461	17.8142	sample=1 perioc

Proteins

Prot. Rank	Protein Name	|Log Prob|	Best |Log Prob|	Best Score	# Spectra	# Uniq. Peps.	# Mod Peps.	% Cov.	# AAs	Intensity
1 | 1	>sp|Q3E770|PAU9_YEAST Seripauperin-9 OS=Saccharomyces cerevisiae (strain ATCC 204508 / S288c) OX=559...	248.32	15.68	955.8	342	58	55	67.5	120	1.797e+7

Spectrum (double click to dock / undock)

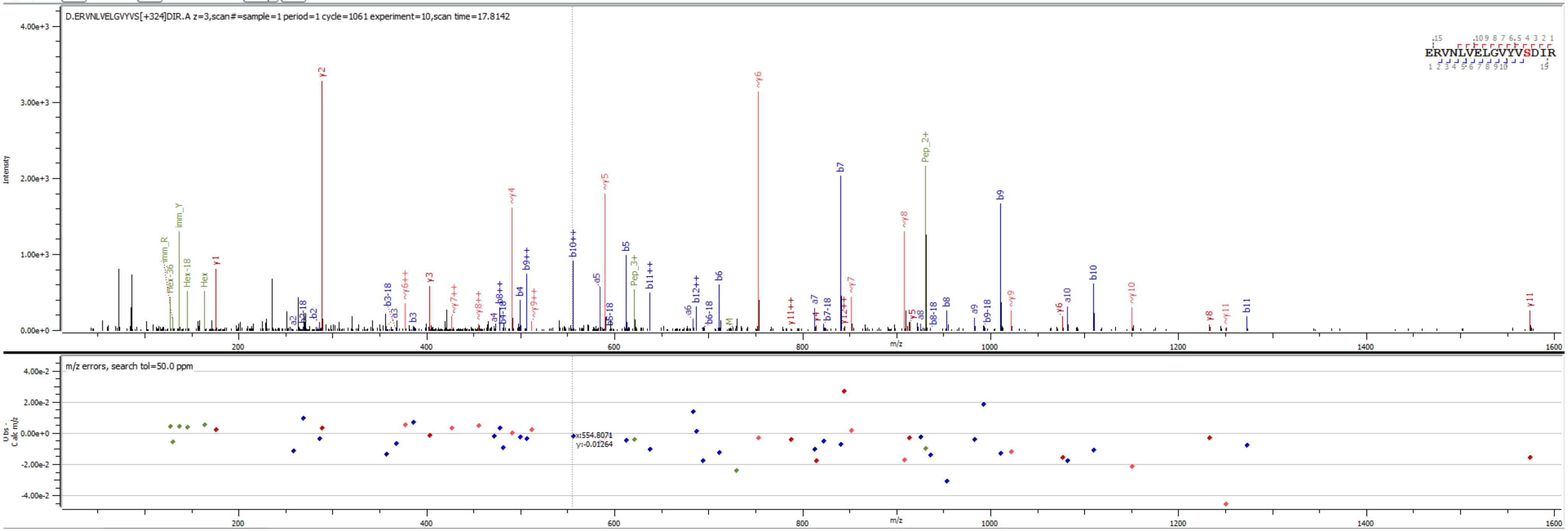

Sample S3 D.ERVNLVELGVYVS[+324.106]DIR.A

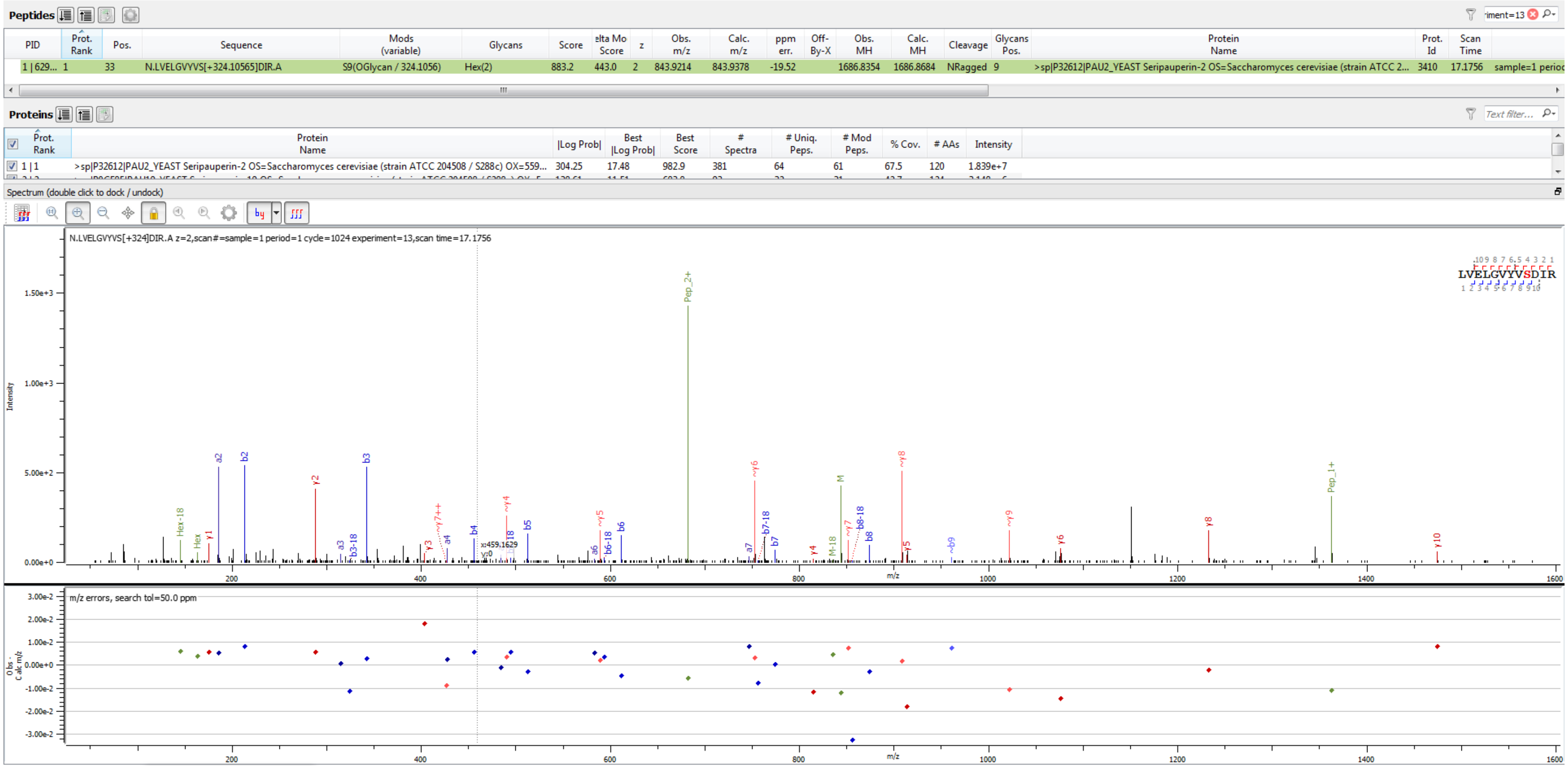

Sample S4 N.LVELGVYVS[+324.106]DIR.A

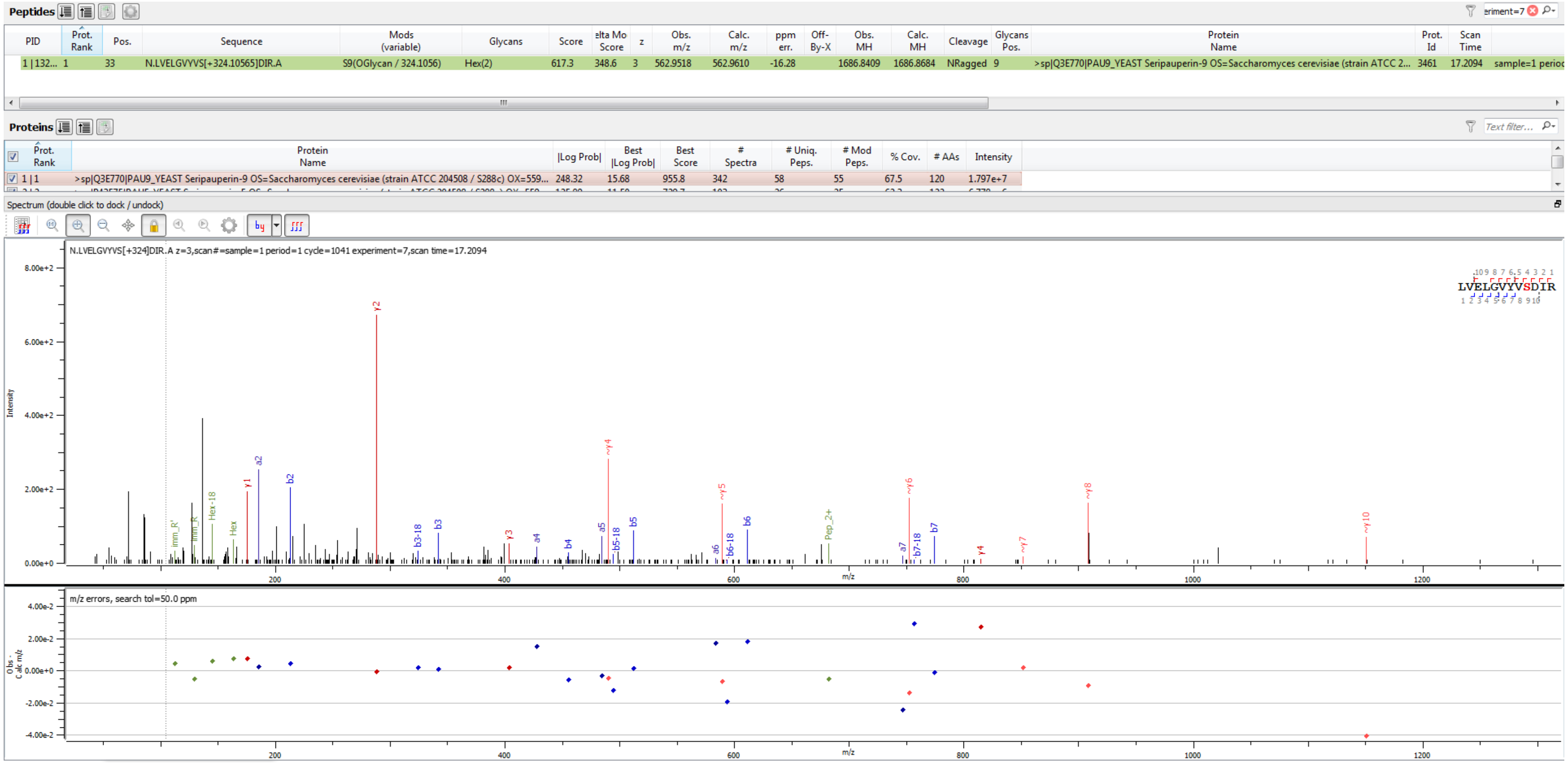

Sample S3 N.LVELGVYVS[+324.106]DIR.A

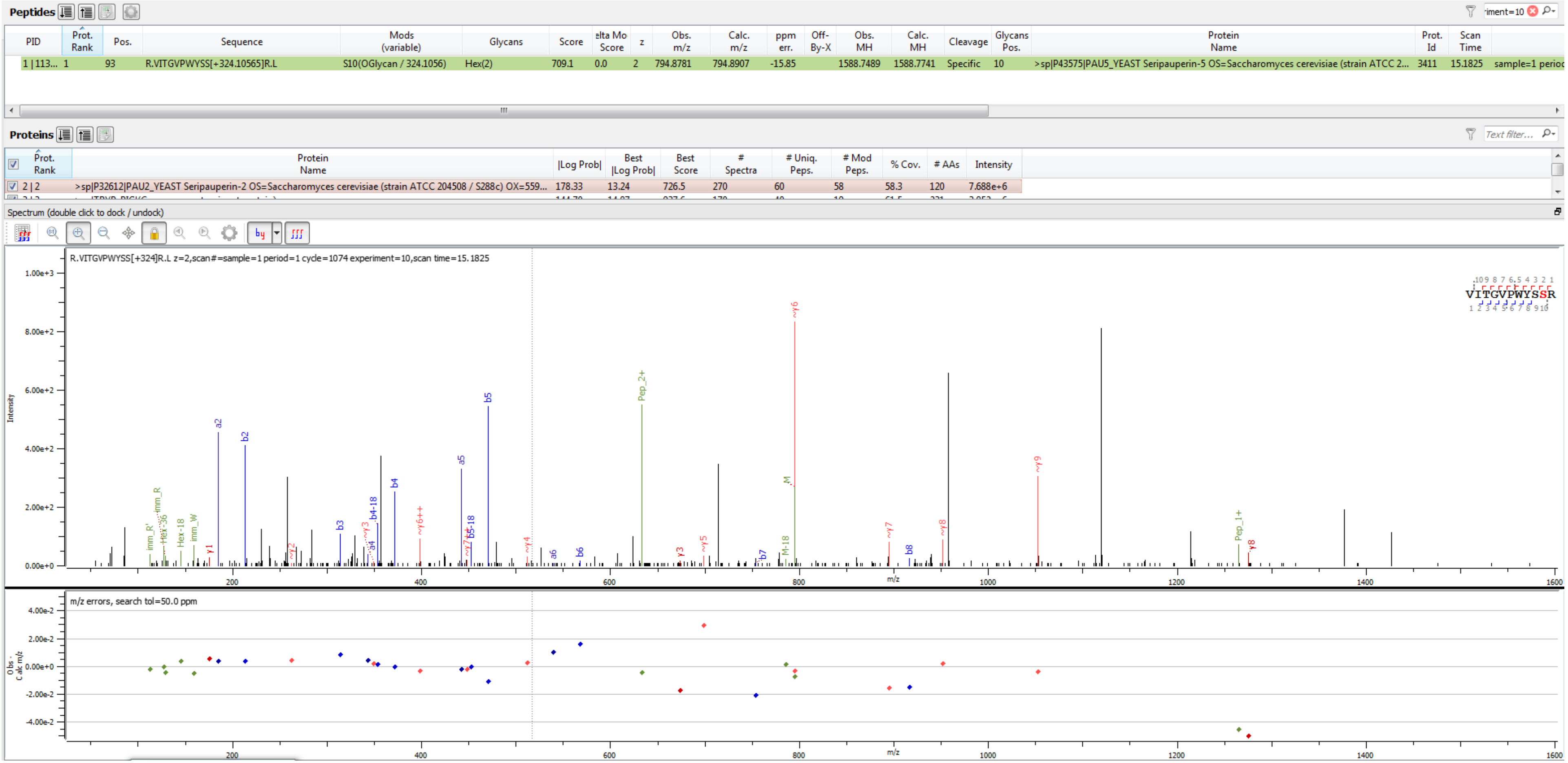

Sample S1 R.VITGVPWYS[+324.106]SR.L

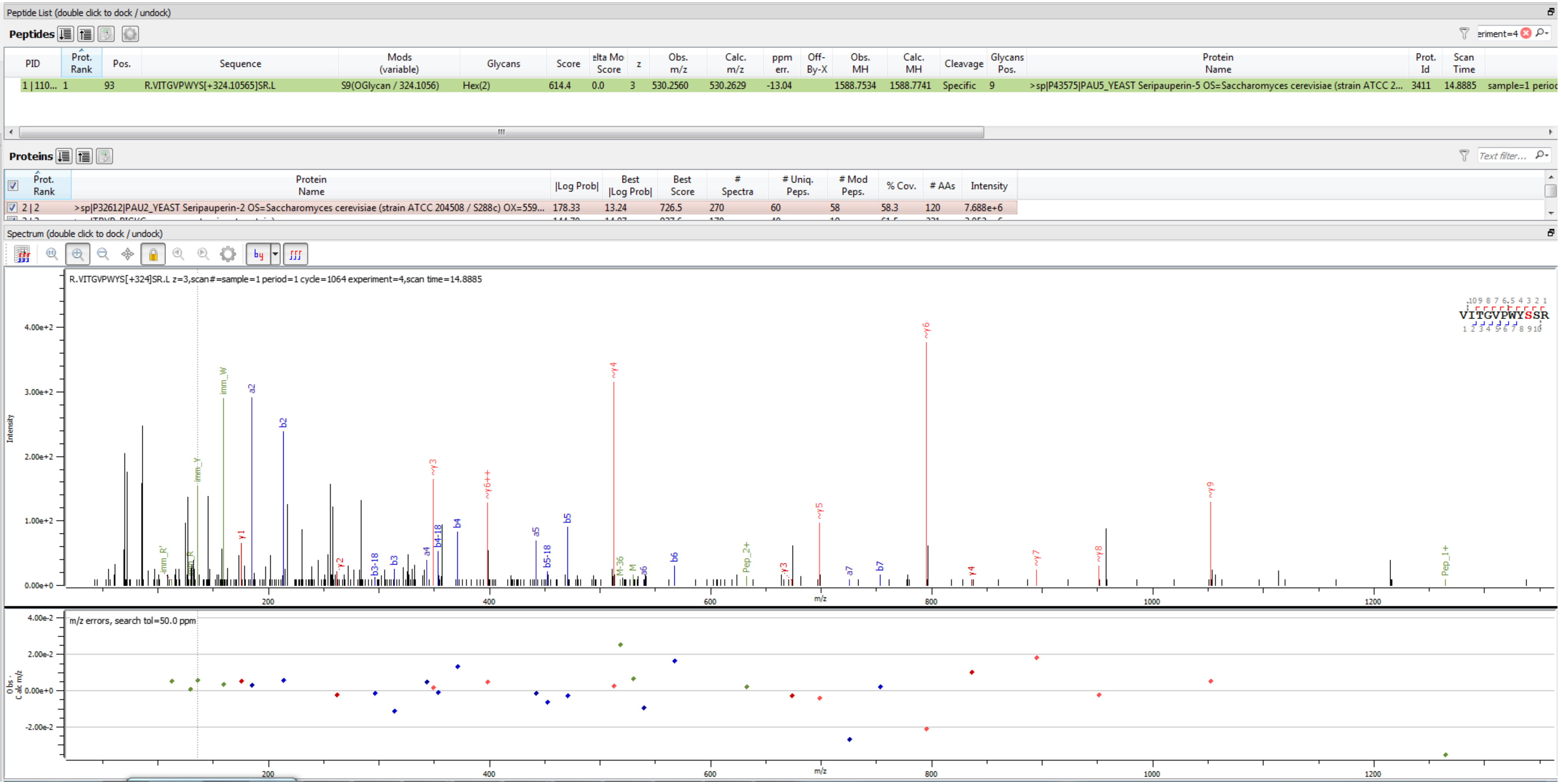

Sample S1 R.VITGVPWYS[+324.106]SR.L

Peptides

PID	Prot. Rank	Pos.	Sequence	Mods (variable)	Glycans	Score	elta Mo Score	z	Obs. m/z	Calc. m/z	ppm err.	Off-By-X	Obs. MH	Calc. MH	Cleavage	Glycans Pos.	Protein Name	Prot. Id	Scan Time	
1 | 110...	1	93	R.VITGVPWYS[+486.15847]SR.L	S9(OGlycan / 486.1585)	Hex(3)	747.1	11.2	2	875.9020	875.9171	-17.27		1750.7967	1750.8269	Specific	9	>sp|P43575|PAU5_YEAST Seripauperin-5 OS=Saccharomyces cerevisiae (strain ATCC 2...	3411	14.8687	sample=1 perioc

Proteins

Prot. Rank	Protein Name	|Log Prob|	Best |Log Prob|	Best Score	# Spectra	# Uniq. Peps.	# Mod Peps.	% Cov.	# AAs	Intensity
2 | 2	>sp|P32612|PAU2_YEAST Seripauperin-2 OS=Saccharomyces cerevisiae (strain ATCC 204508 / S288c) OX=559...	178.33	13.24	726.5	270	60	58	58.3	120	7.688e+6

Spectrum (double click to dock / undock)

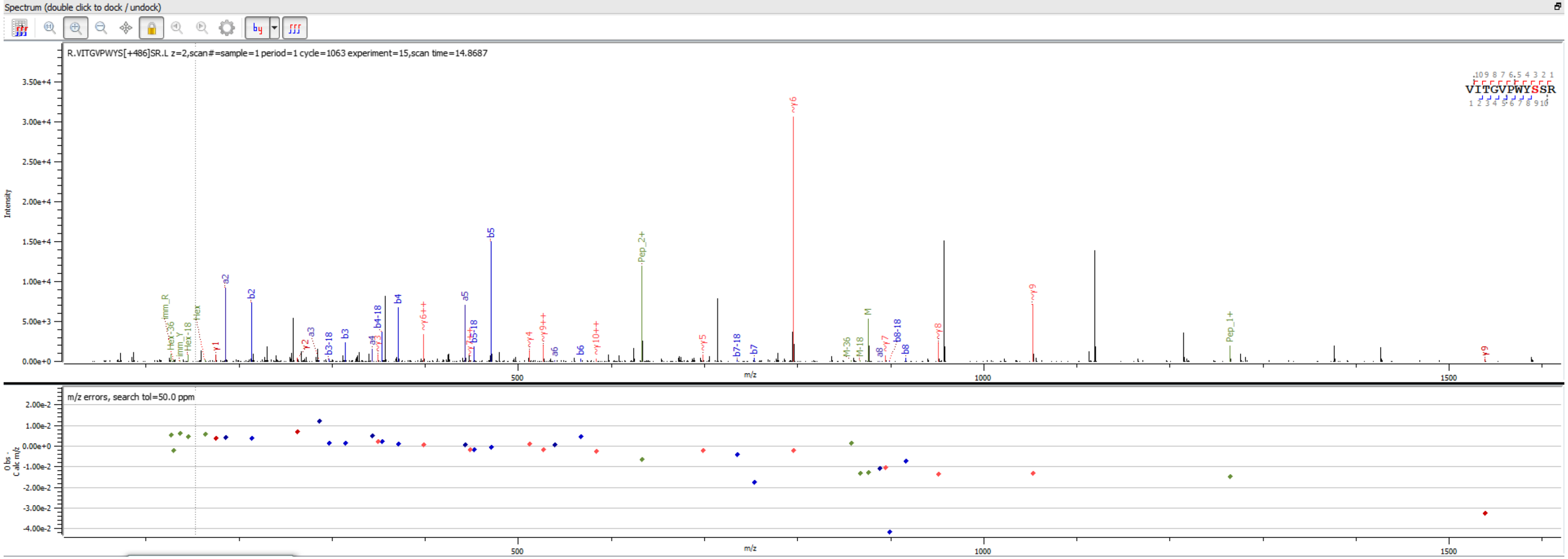

Sample S1 R.VITGVPWYS[+486.158]SR.L

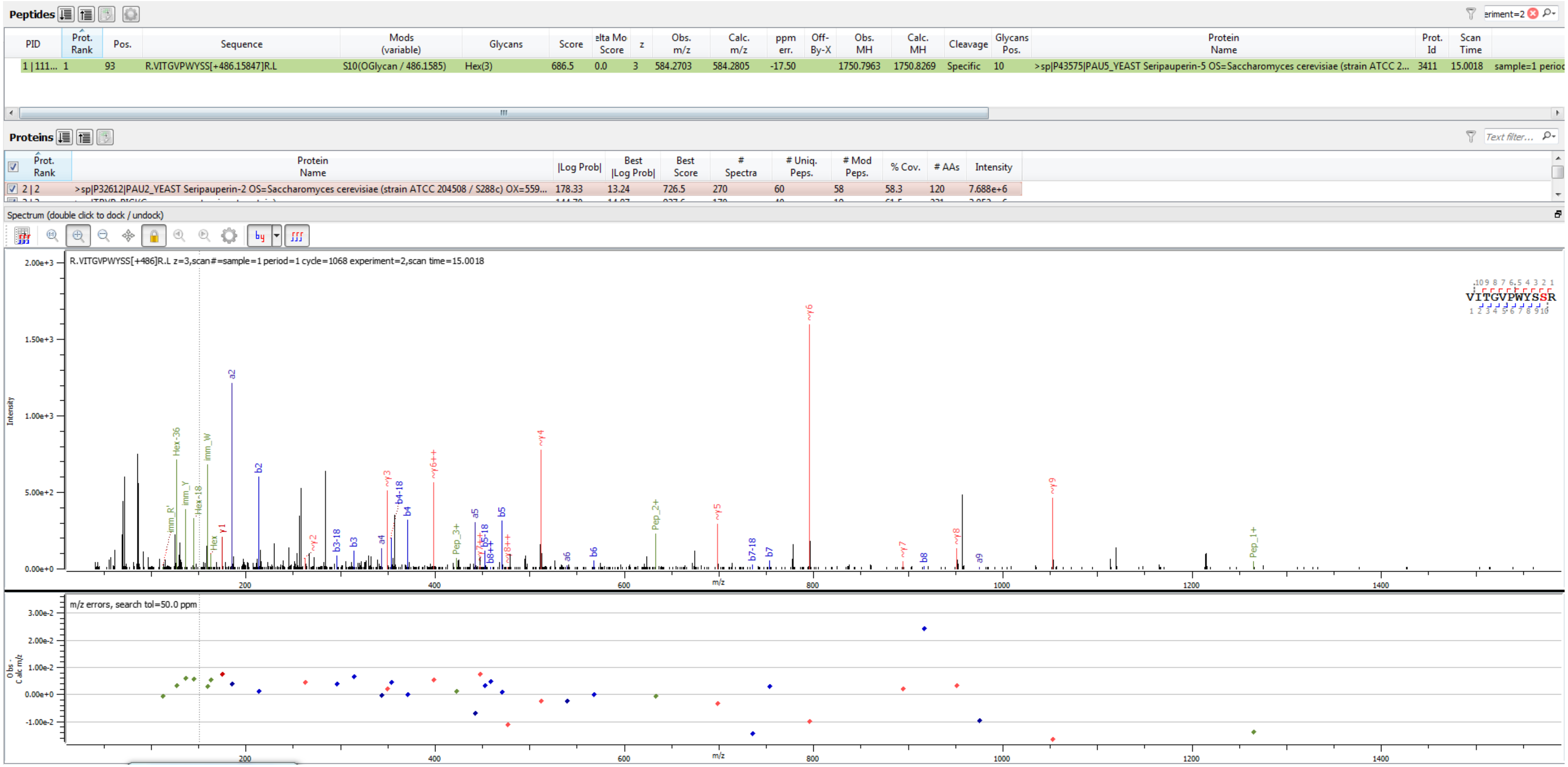

Sample S1 R.VITGVPWYS[+486.158]SR.L

Peptides

PID	Prot. Rank	Pos.	Sequence	Mods (variable)	Glycans	Score	elta Mo Score	z	Obs. m/z	Calc. m/z	ppm err.	Off-By-X	Obs. MH	Calc. MH	Cleavage	Glycans Pos.	Protein Name	Prot. Id	Scan Time	
1 | 108...	1	93	R.VITGVPWYS[+648.21129]SR.L	S9(OGlycan / 648.2113)	Hex(4)	674.6	0.0	2	956.9261	956.9435	-18.15		1912.8450	1912.8797	Specific	9	>sp|P43575|PAU5_YEAST Seripauperin-5 OS=Saccharomyces cerevisiae (strain ATCC 2...	3411	14.6700	sample=1 perioc

Proteins

Prot. Rank	Protein Name	|Log Prob|	Best |Log Prob|	Best Score	# Spectra	# Uniq. Peps.	# Mod Peps.	% Cov.	# AAs	Intensity
2 | 2	>sp|P32612|PAU2_YEAST Seripauperin-2 OS=Saccharomyces cerevisiae (strain ATCC 204508 / S288c) OX=559...	178.33	13.24	726.5	270	60	58	58.3	120	7.688e+6

Spectrum (double click to dock / undock)

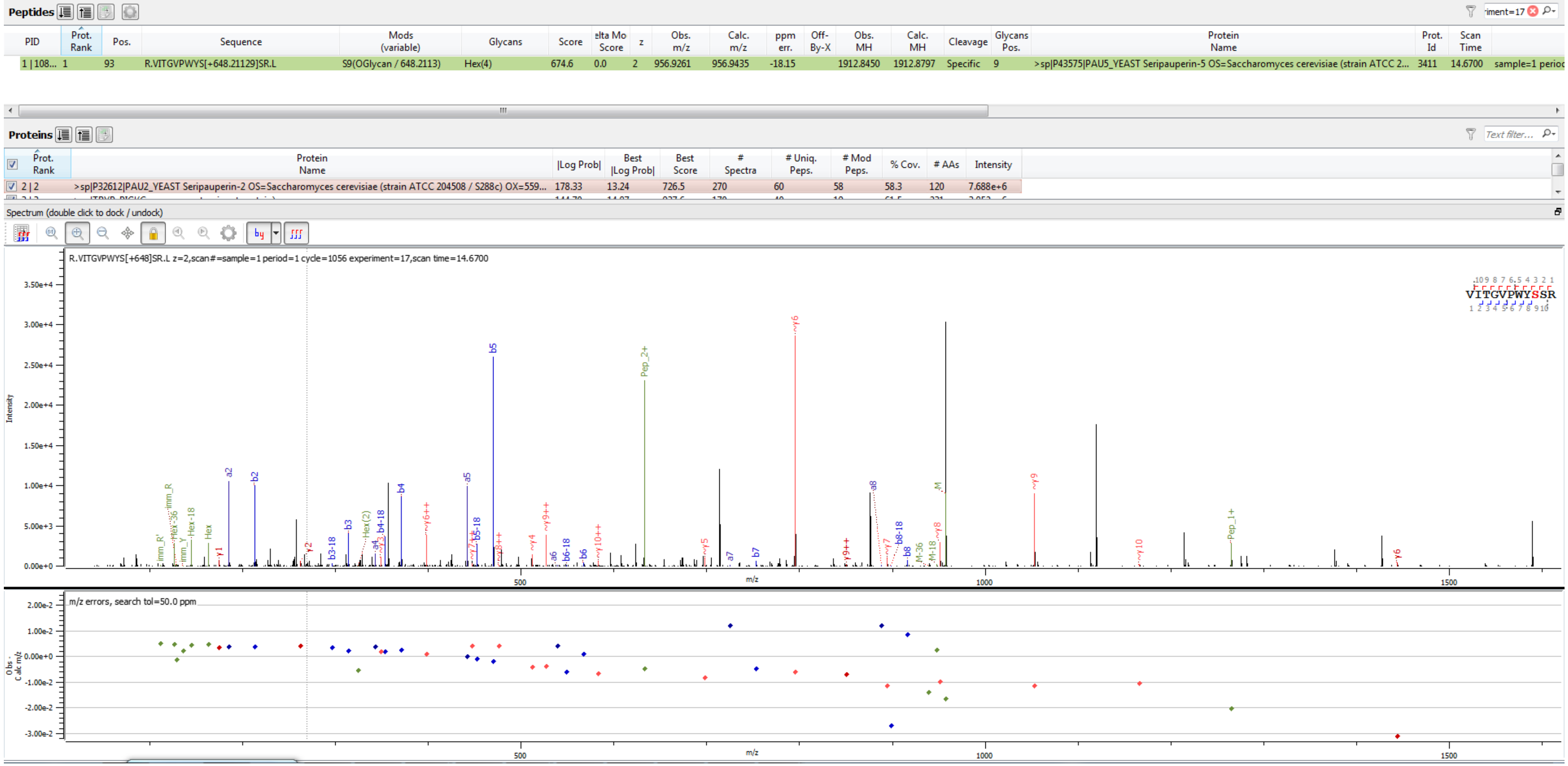

Sample S1 R.VITGVPWYS[+648.211]SR.L

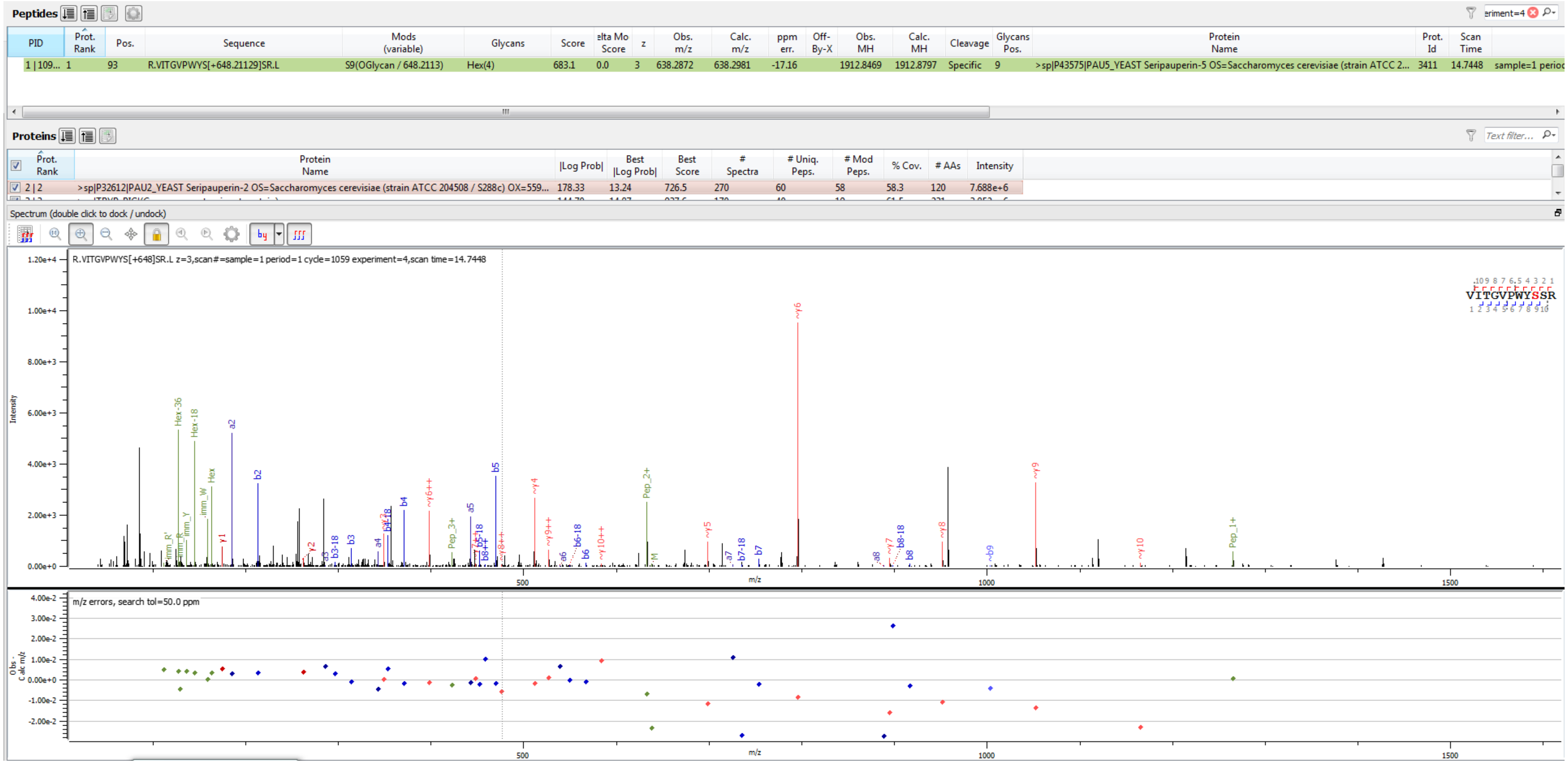

Sample S1 R.VITGVPWYS[+648.211]SR.L

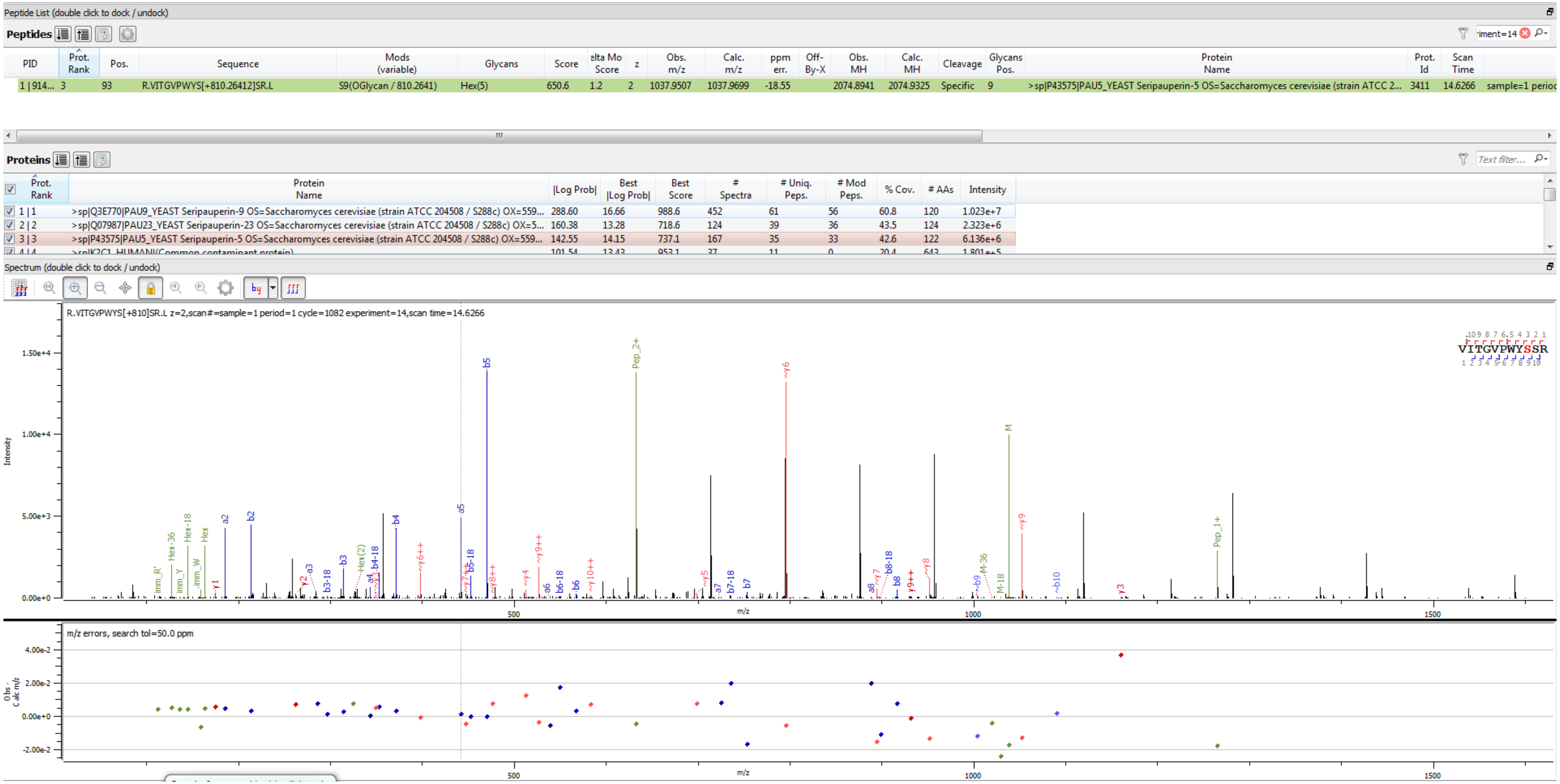

Sample S6 R.VITGVPWYS[+810.264]SR.L

Peptides

eriment=6

PID	Prot. Rank	Pos.	Sequence	Mods (variable)	Glycans	Score	elta Mo Score	z	Obs. m/z	Calc. m/z	ppm err.	Off-By-X	Obs. MH	Calc. MH	Cleavage	Glycans Pos.	Protein Name	Prot. Id	Scan Time	
1 | 107...	1	93	R.VITGVPWYS[+810.26412]SR.L	S9(OGlycan / 810.2641)	Hex(5)	725.4	1.2	3	692.3033	692.3157	-17.91		2074.8954	2074.9325	Specific	9	>sp|P43575|PAU5_YEAST Seripauperin-5 OS=Saccharomyces cerevisiae (strain ATCC 2...	3411	14.5472	sample=1 perioc

Proteins

Text filter...

	Prot. Rank	Protein Name	|Log Prob|	Best |Log Prob|	Best Score	# Spectra	# Uniq. Peps.	# Mod Peps.	% Cov.	# AAs	Intensity
☑	1 | 1	>sp|P43575|PAU5_YEAST Seripauperin-5 OS=Saccharomyces cerevisiae (strain ATCC 204508 / S288c) OX=559...	327.47	16.74	1004.1	632	70	67	68.9	122	3.742e+7
☑	2 | 2	>sp|P32612|PAU2_YEAST Seripauperin-2 OS=Saccharomyces cerevisiae (strain ATCC 204508 / S288c) OX=559...	178.33	13.24	726.5	270	60	58	58.3	120	7.688e+6

Spectrum (double click to dock / undock)

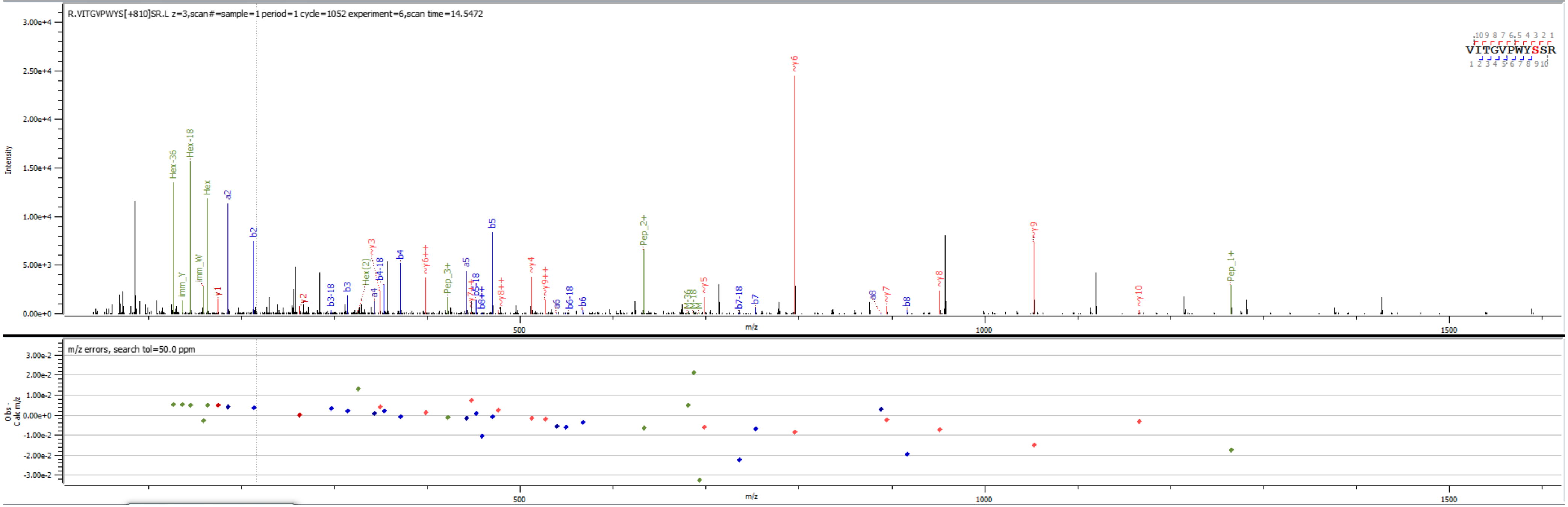

Sample S1 R.VITGVPWYS[+810.264]SR.L

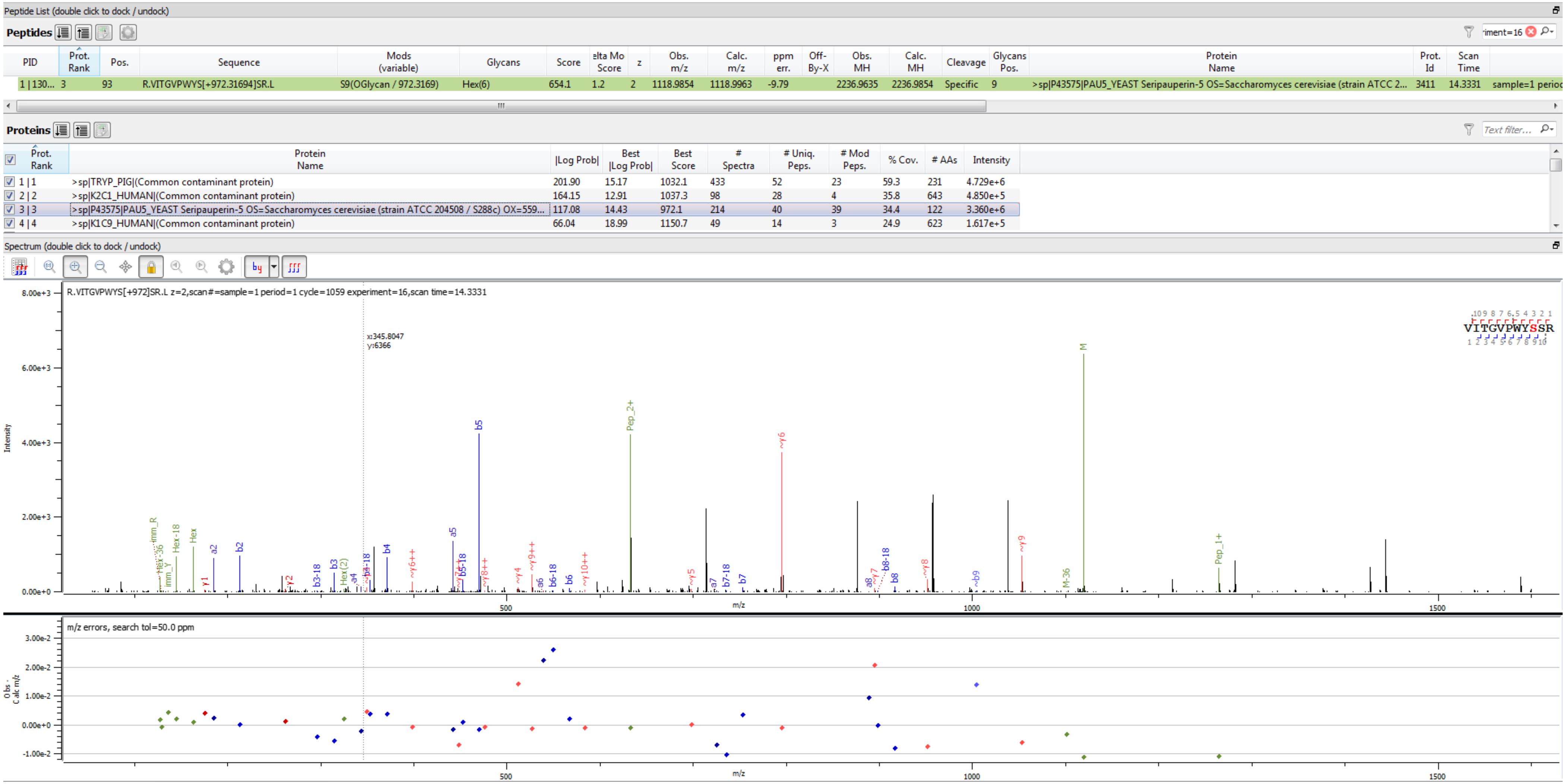

Sample S2 R.VITGVPWYS[+972.317]SR.L

Peptides

PID	Prot. Rank	Pos.	Sequence	Mods (variable)	Glycans	Score	elta Mo Score	z	Obs. m/z	Calc. m/z	ppm err.	Off-By-X	Obs. MH	Calc. MH	Cleavage	Glycans Pos.	Protein Name	Prot. Id	Scan Time	
1 | 180...	1	93	R.VITGVPWYS[+972.31694]SR.L	S9(OGlycan / 972.3169)	Hex(6)	712.2	1.2	3	746.3205	746.3333	-17.15		2236.9470	2236.9854	Specific	9	>sp|P43575|PAU5_YEAST Seripauperin-5 OS=Saccharomyces cerevisiae (strain ATCC 2...	3411	14.2101	sample=1 perioc

Proteins

	Prot. Rank	Protein Name	|Log Prob|	Best |Log Prob|	Best Score	# Spectra	# Uniq. Peps.	# Mod Peps.	% Cov.	# AAs	Intensity
☑	1 | 1	>sp|P43575|PAU5_YEAST Seripauperin-5 OS=Saccharomyces cerevisiae (strain ATCC 204508 / S288c) OX=559...	327.47	16.74	1004.1	632	70	67	68.9	122	3.742e+7
☑	2 | 2	>sp|P32612|PAU2_YEAST Seripauperin-2 OS=Saccharomyces cerevisiae (strain ATCC 204508 / S288c) OX=559...	178.33	13.24	726.5	270	60	58	58.3	120	7.688e+6

Spectrum (double click to dock / undock)

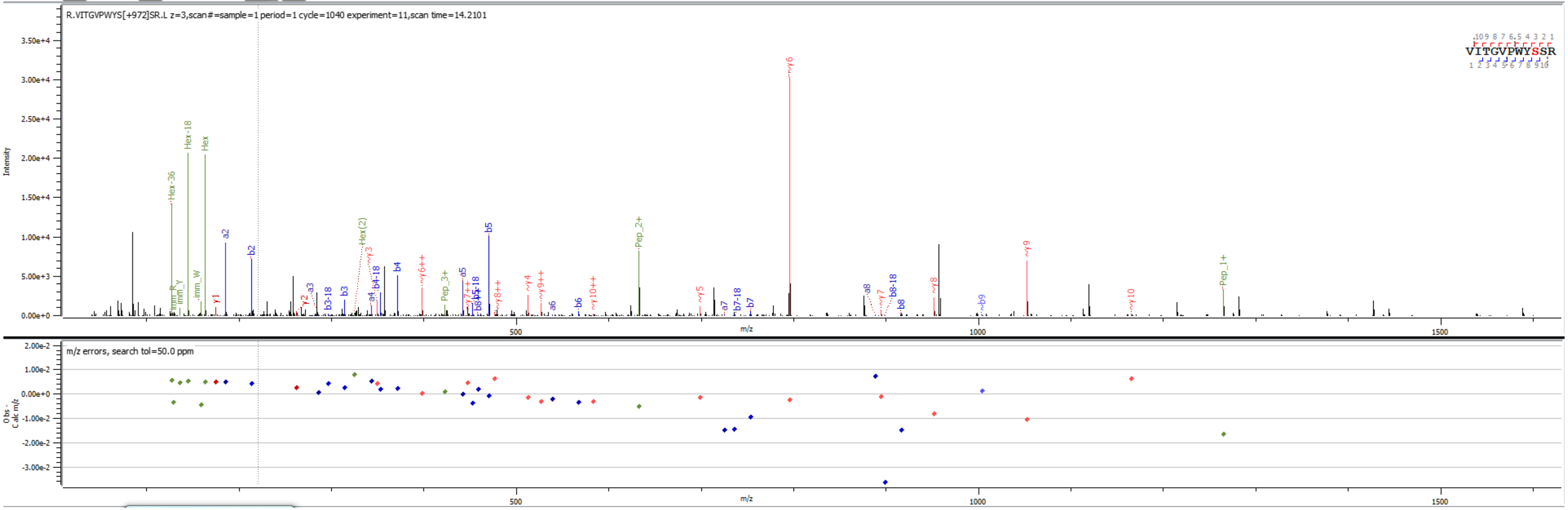

Sample S1 R.VITGVPWYS[+972.317]SR.L

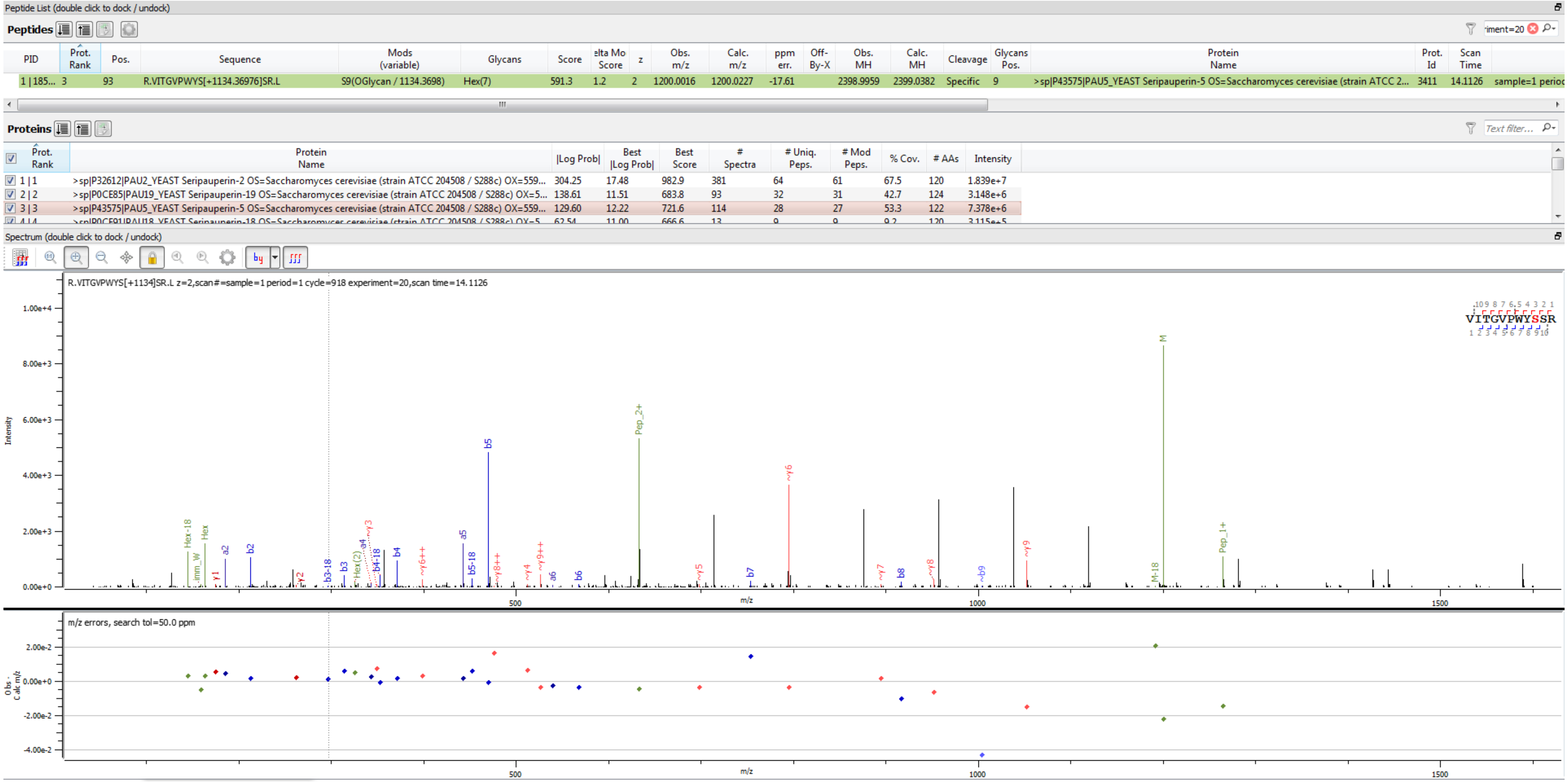

Sample S4 R.VITGVPWYS[+1134.370]SR.L

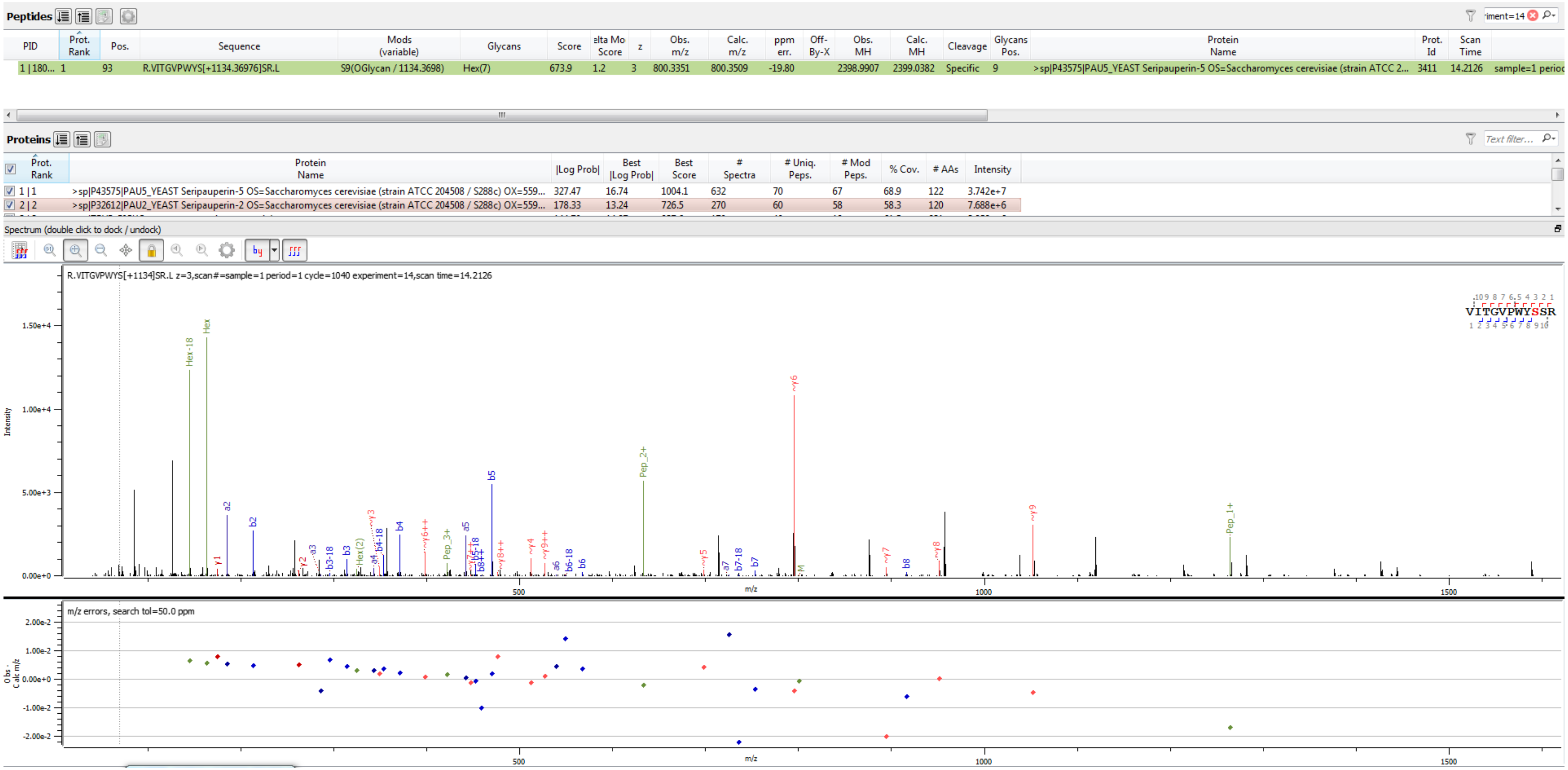

Sample S1 R.VITGVPWYS[+1134.370]SR.L

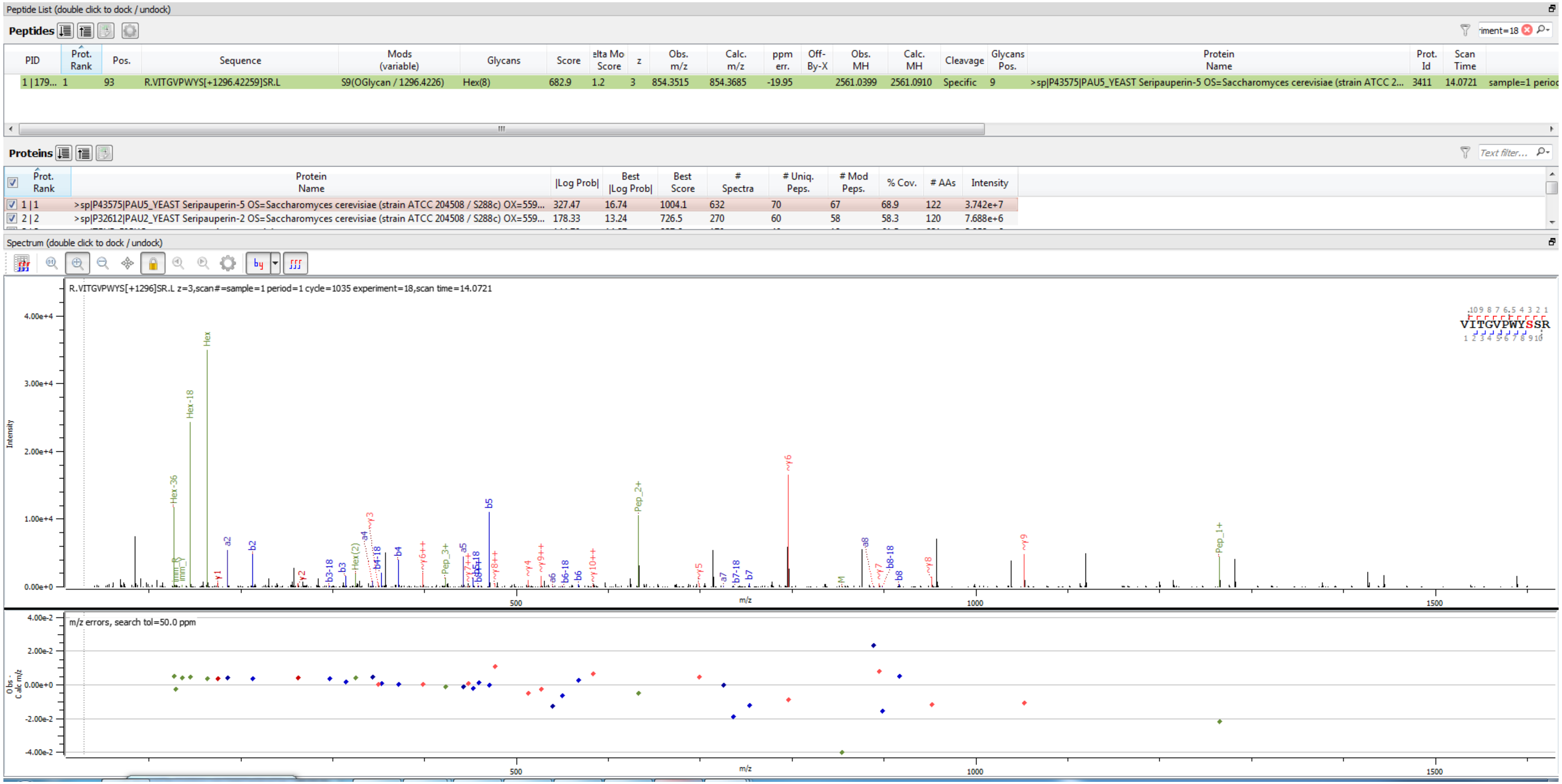

Sample S1 R.VITGVPWYS[+1296.423]SR.L

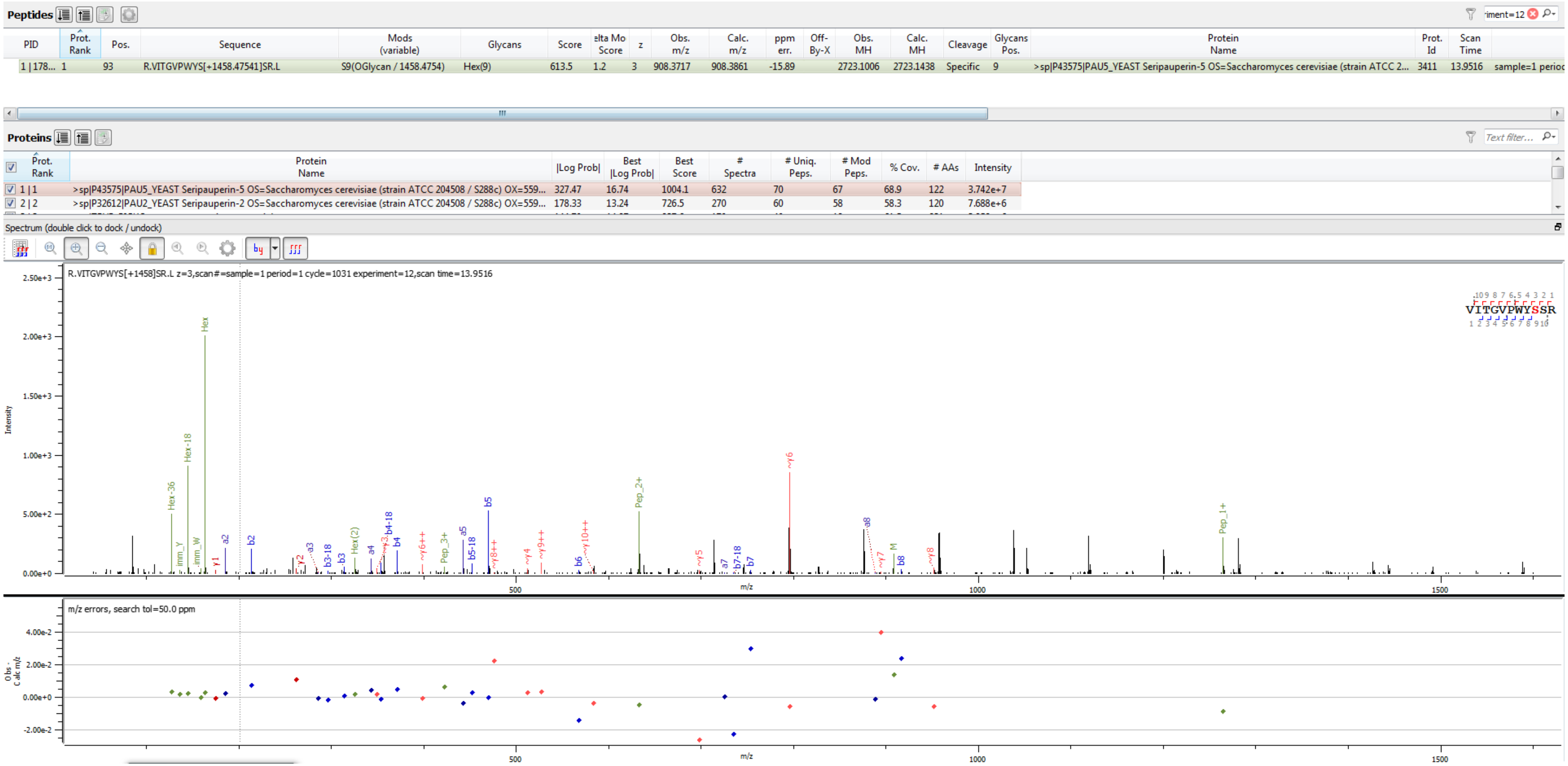

Sample S1 R.VITGVPWYS[+1458.475]SR.L

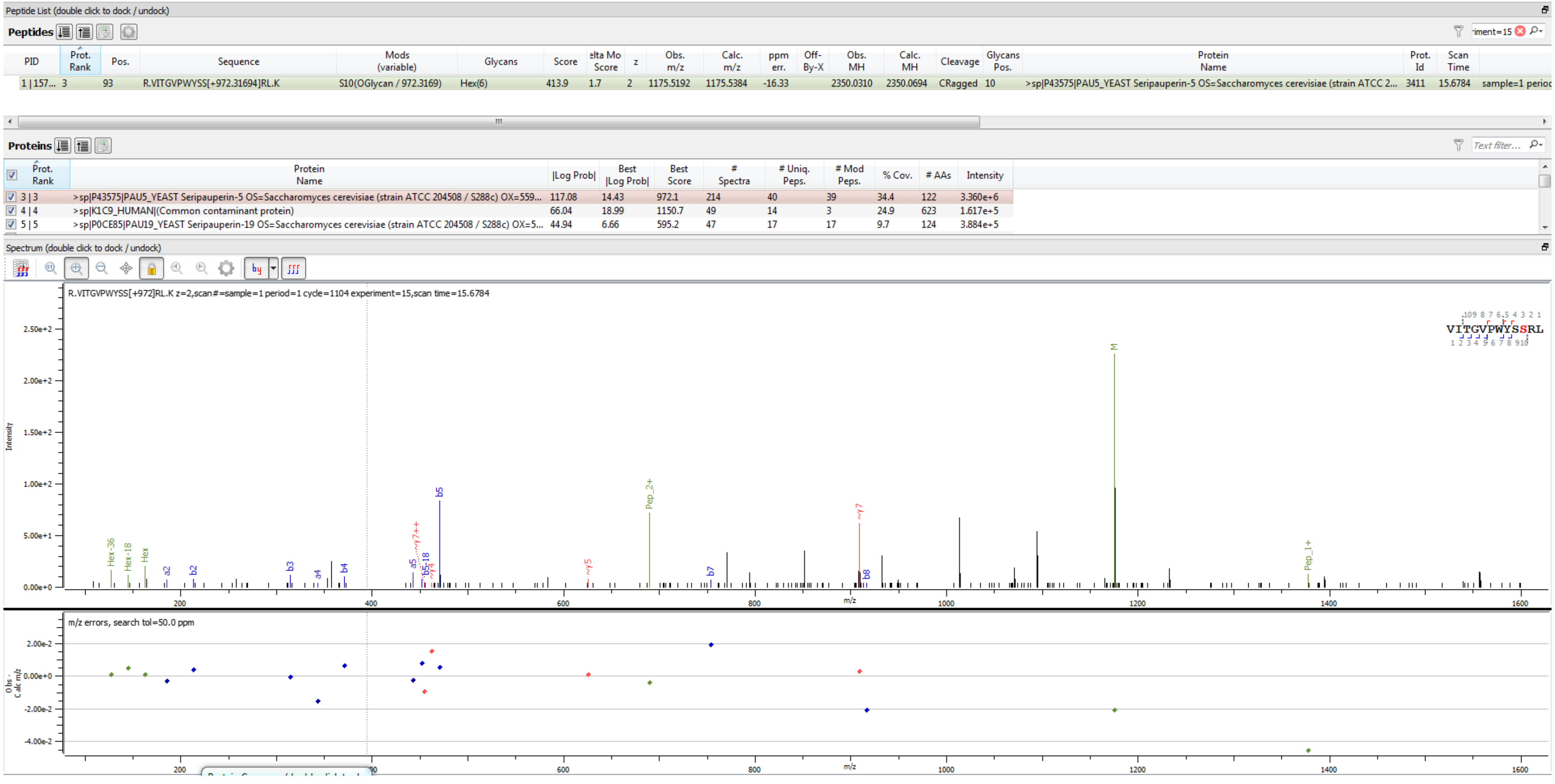

Sample S2 R.VITGVPWYSS[+972.317]RL.K

Peptides

PID	Prot. Rank	Pos.	Sequence	Mods (variable)	Glycans	Score	elta Mo Score	z	Obs. m/z	Calc. m/z	ppm err.	Off-By-X	Obs. MH	Calc. MH	Cleavage	Glycans Pos.	Protein Name	Prot. Id	Scan Time	
1 | 157...	3	93	R.VITGVPWYS[+972.31694]SRL.K	S9(OGlycan / 972.3169)	Hex(6)	577.7	1.2	3	784.0136	784.0280	-18.33		2350.0264	2350.0694	CRagged	9	>sp|P43575|PAU5_YEAST Seripauperin-5 OS=Saccharomyces cerevisiae (strain ATCC 2...	3411	15.6986	sample=1 perioc

Proteins

Prot. Rank	Protein Name	|Log Prob|	Best |Log Prob|	Best Score	# Spectra	# Uniq. Peps.	# Mod Peps.	% Cov.	# AAs	Intensity
3 | 3	>sp|P43575|PAU5_YEAST Seripauperin-5 OS=Saccharomyces cerevisiae (strain ATCC 204508 / S288c) OX=559...	117.08	14.43	972.1	214	40	39	34.4	122	3.360e+6
4 | 4	>sp|K1C9_HUMAN|(Common contaminant protein)	66.04	18.99	1150.7	49	14	3	24.9	623	1.617e+5
5 | 5	>sp|P0CE85|PAU19_YEAST Seripauperin-19 OS=Saccharomyces cerevisiae (strain ATCC 204508 / S288c) OX=5...	44.94	6.66	595.2	47	17	17	9.7	124	3.884e+5

Spectrum (double click to dock / undock)

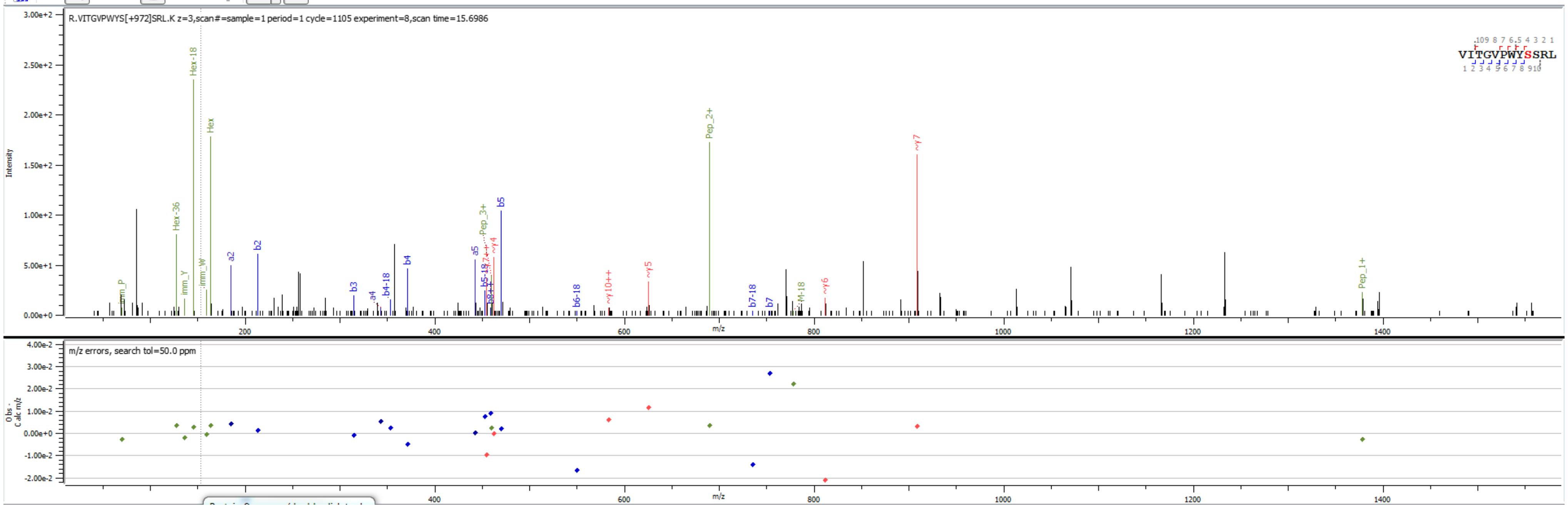

Sample S2 R.VITGVPWYS[+972.317]SRL.K

Peptides

PID	Prot. Rank	Pos.	Sequence	Mods (variable)	Glycans	Score	elta Mo Score	z	Obs. m/z	Calc. m/z	ppm err.	Off-By-X	Obs. MH	Calc. MH	Cleavage	Glycans Pos.	Protein Name	Prot. Id	Scan Time	
1 | 169...	3	93	R.VITGVPWYS[+1134.36976]SRL.K	S9(OGlycan / 1134.3698)	Hex(7)	582.9	1.2	3	838.0314	838.0456	-17.01		2512.0795	2512.1223	CRagged	9	>sp|P43575|PAU5_YEAST Seripauperin-5 OS=Saccharomyces cerevisiae (strain ATCC 2...	3411	15.4899	sample=1 perioc

Proteins

Prot. Rank	Protein Name	|Log Prob|	Best |Log Prob|	Best Score	# Spectra	# Uniq. Peps.	# Mod Peps.	% Cov.	# AAs	Intensity
3 | 3	>sp|P43575|PAU5_YEAST Seripauperin-5 OS=Saccharomyces cerevisiae (strain ATCC 204508 / S288c) OX=559...	117.08	14.43	972.1	214	40	39	34.4	122	3.360e+6

Spectrum (double click to dock / undock)

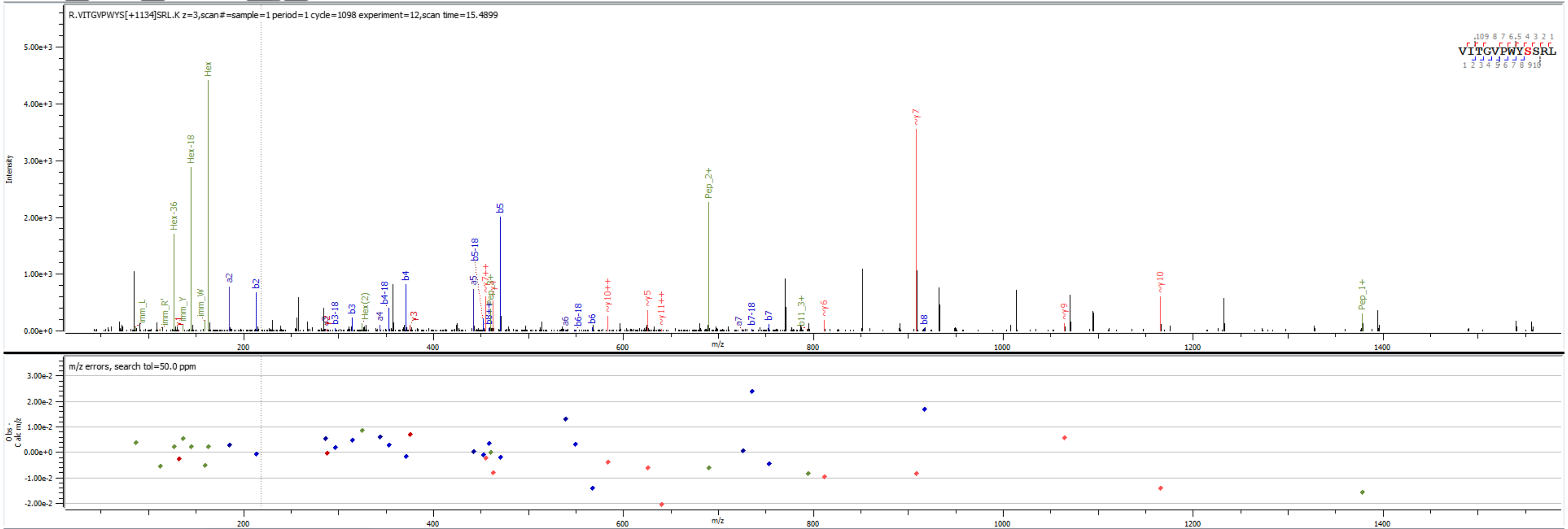

Sample S2 R.VITGVPWYS[+1134.370]SRL.K

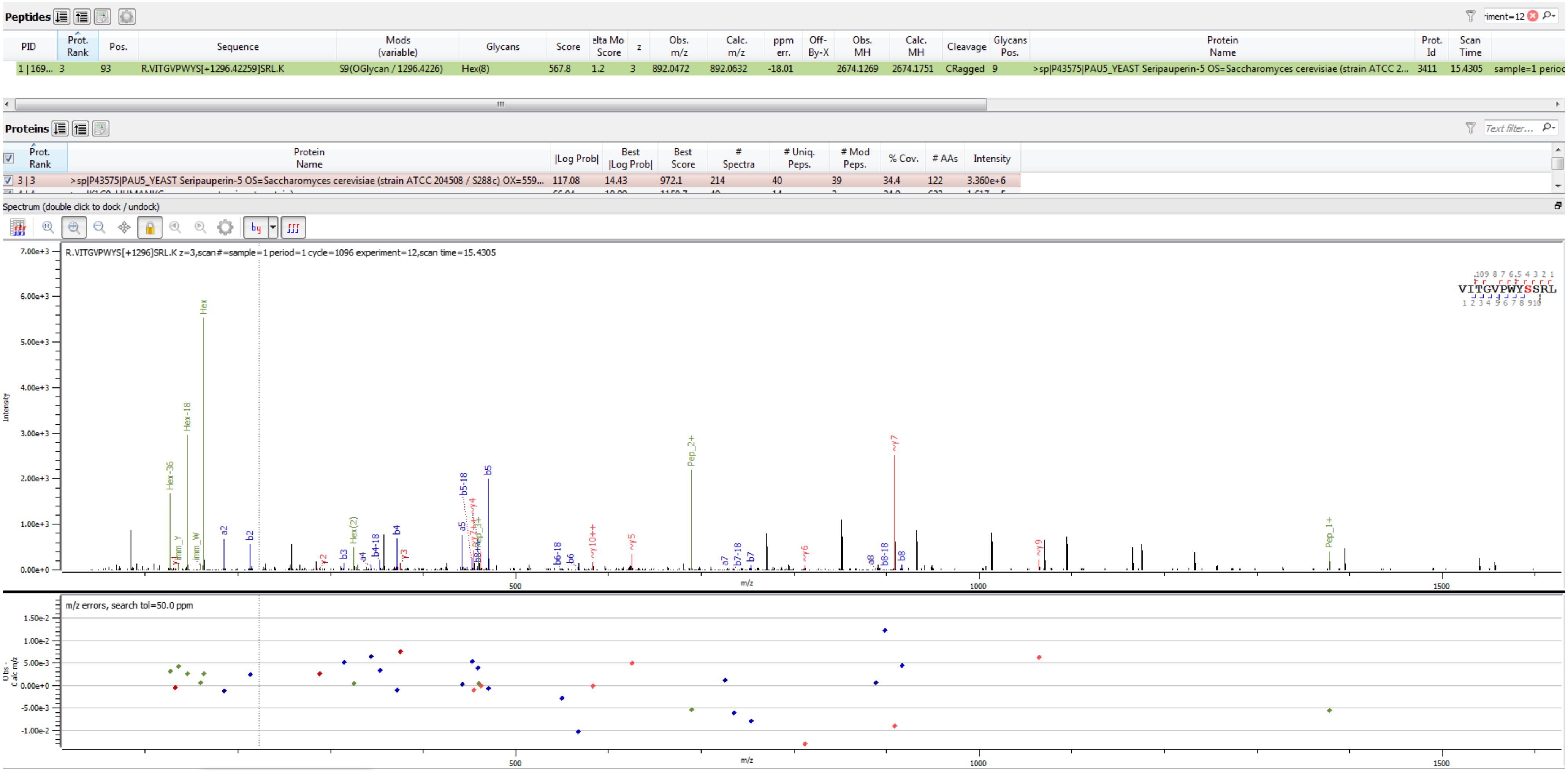

Sample S2 R.VITGVPWYS[+1296.423]SRL.K

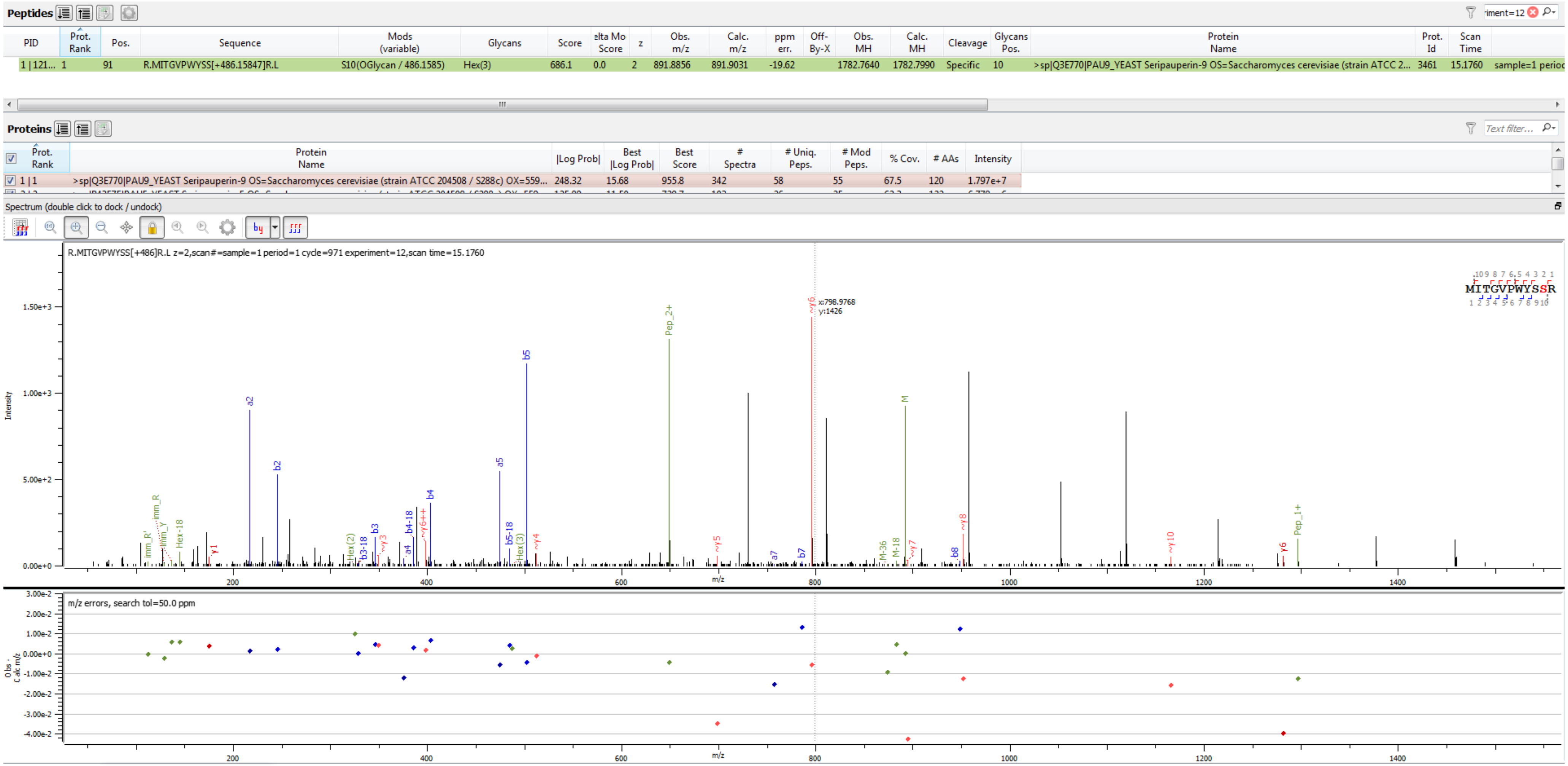

Sample S3 R.MITGVPWYSS[+486.158]R.L

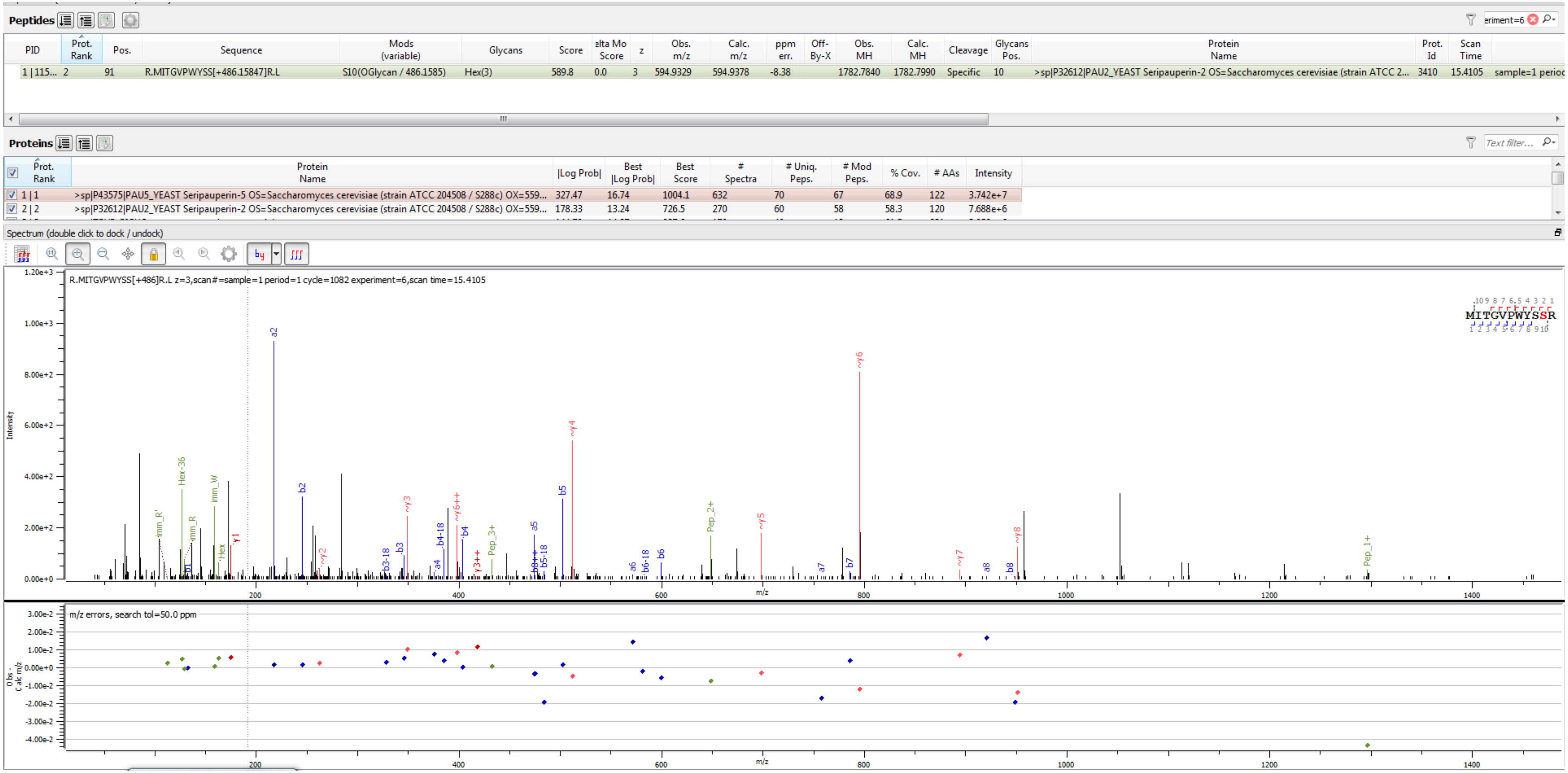

Peptides

PID	Prot. Rank	Pos.	Sequence	Mods (variable)	Glycans	Score	elta Mo Score	z	Obs. m/z	Calc. m/z	ppm err.	Off-By-X	Obs. MH	Calc. MH	Cleavage	Glycans Pos.	Protein Name	Prot. Id	Scan Time	
1 | 115...	2	91	R.MITGVPWYSS[+486.15847]R.L	S10(OGlycan / 486.1585)	Hex(3)	589.8	0.0	3	594.9329	594.9378	-8.38		1782.7840	1782.7990	Specific	10	>sp|P32612|PAU2_YEAST Seripauperin-2 OS=Saccharomyces cerevisiae (strain ATCC 2...	3410	15.4105	sample=1 perioc

Proteins

Prot. Rank	Protein Name	|Log Prob|	Best |Log Prob|	Best Score	# Spectra	# Uniq. Peps.	# Mod Peps.	% Cov.	# AAs	Intensity
1 | 1	>sp|P43575|PAU5_YEAST Seripauperin-5 OS=Saccharomyces cerevisiae (strain ATCC 204508 / S288c) OX=559...	327.47	16.74	1004.1	632	70	67	68.9	122	3.742e+7
2 | 2	>sp|P32612|PAU2_YEAST Seripauperin-2 OS=Saccharomyces cerevisiae (strain ATCC 204508 / S288c) OX=559...	178.33	13.24	726.5	270	60	58	58.3	120	7.688e+6

Sample S1 R.MITGVPWYSS[+486.158]R.L

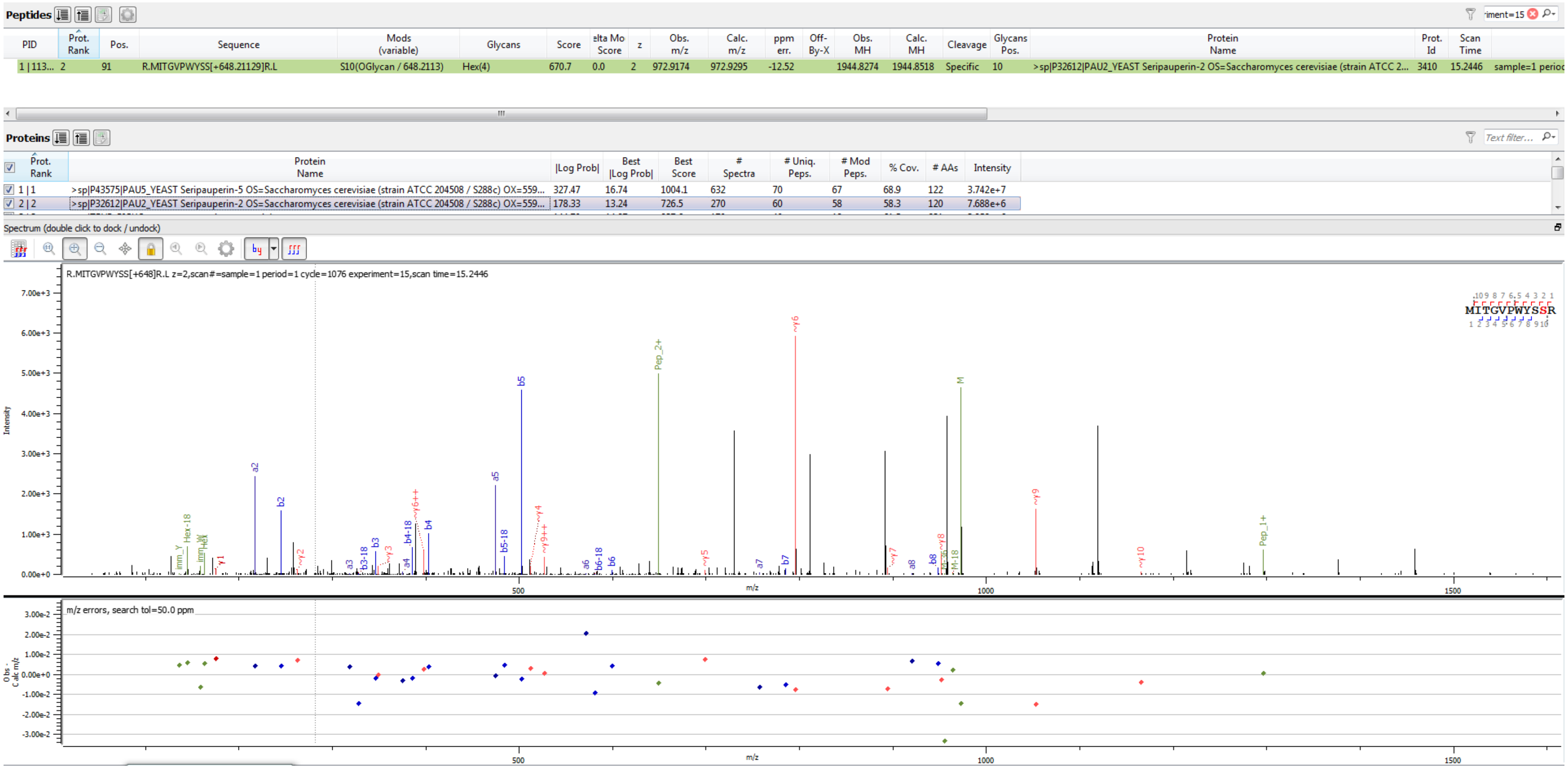

Sample S1 R.MITGVPWYSS[+648.211]R.L

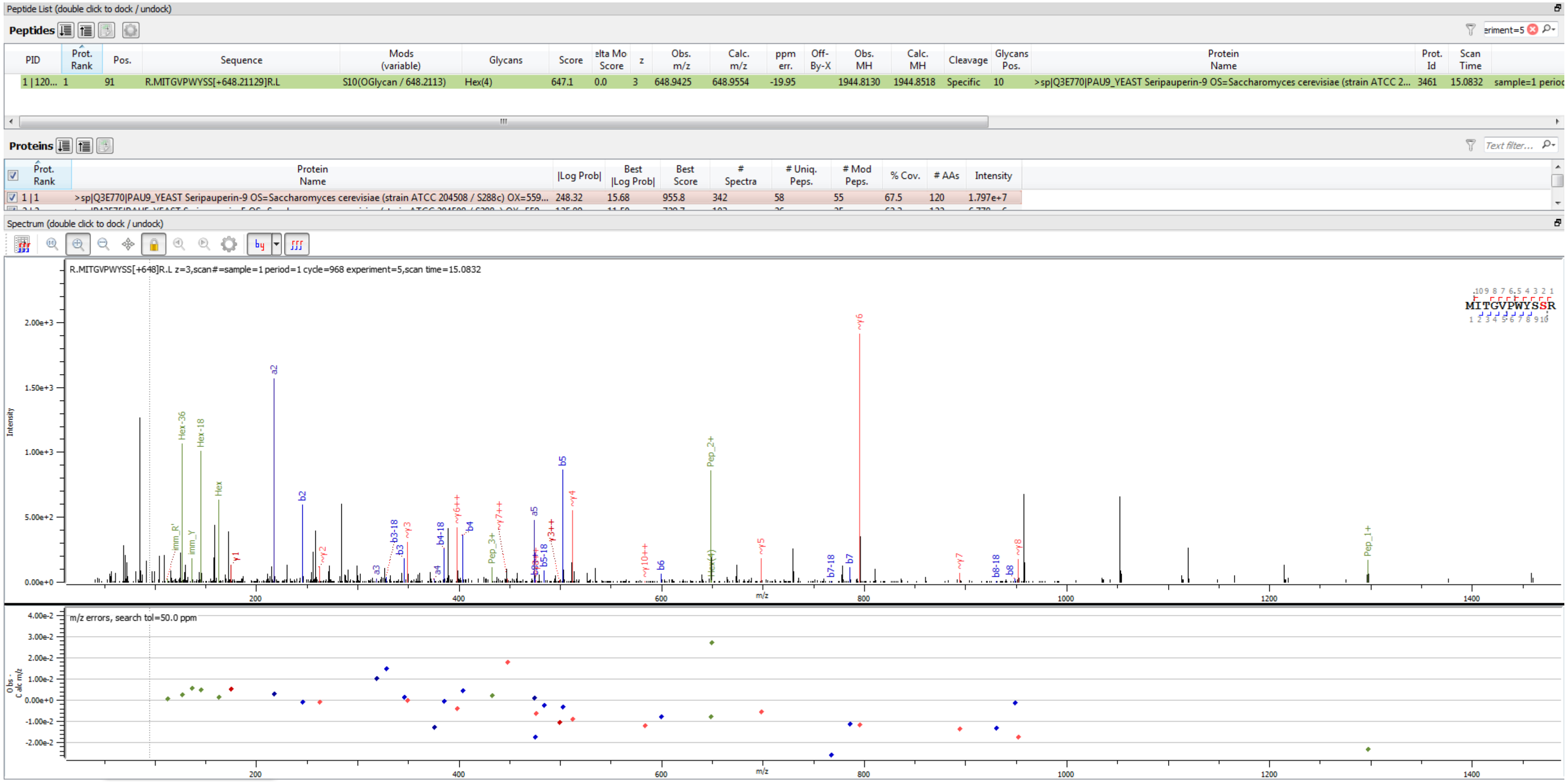

Sample S3   R.MITGVPWYSS[+648.211]R.L

Peptides

PID	Prot. Rank	Pos.	Sequence	Mods (variable)	Glycans	Score	elta Mo Score	z	Obs. m/z	Calc. m/z	ppm err.	Off-By-X	Obs. MH	Calc. MH	Cleavage	Glycans Pos.	Protein Name	Prot. Id	Scan Time	
1 | 187...	2	91	R.MITGVPWYS[+810.26412]SR.L	S9(OGlycan / 810.2641)	Hex(5)	658.5	1.2	2	1053.9396	1053.9559	-15.52		2106.8719	2106.9046	Specific	9	>sp|P32612|PAU2_YEAST Seripauperin-2 OS=Saccharomyces cerevisiae (strain ATCC 2...	3410	15.0441	sample=1 perioc

Proteins

Prot. Rank	Protein Name	|Log Prob|	Best |Log Prob|	Best Score	# Spectra	# Uniq. Peps.	# Mod Peps.	% Cov.	# AAs	Intensity
1 | 1	>sp|P43575|PAU5_YEAST Seripauperin-5 OS=Saccharomyces cerevisiae (strain ATCC 204508 / S288c) OX=559...	327.47	16.74	1004.1	632	70	67	68.9	122	3.742e+7
2 | 2	>sp|P32612|PAU2_YEAST Seripauperin-2 OS=Saccharomyces cerevisiae (strain ATCC 204508 / S288c) OX=559...	178.33	13.24	726.5	270	60	58	58.3	120	7.688e+6

Spectrum (double click to dock / undock)

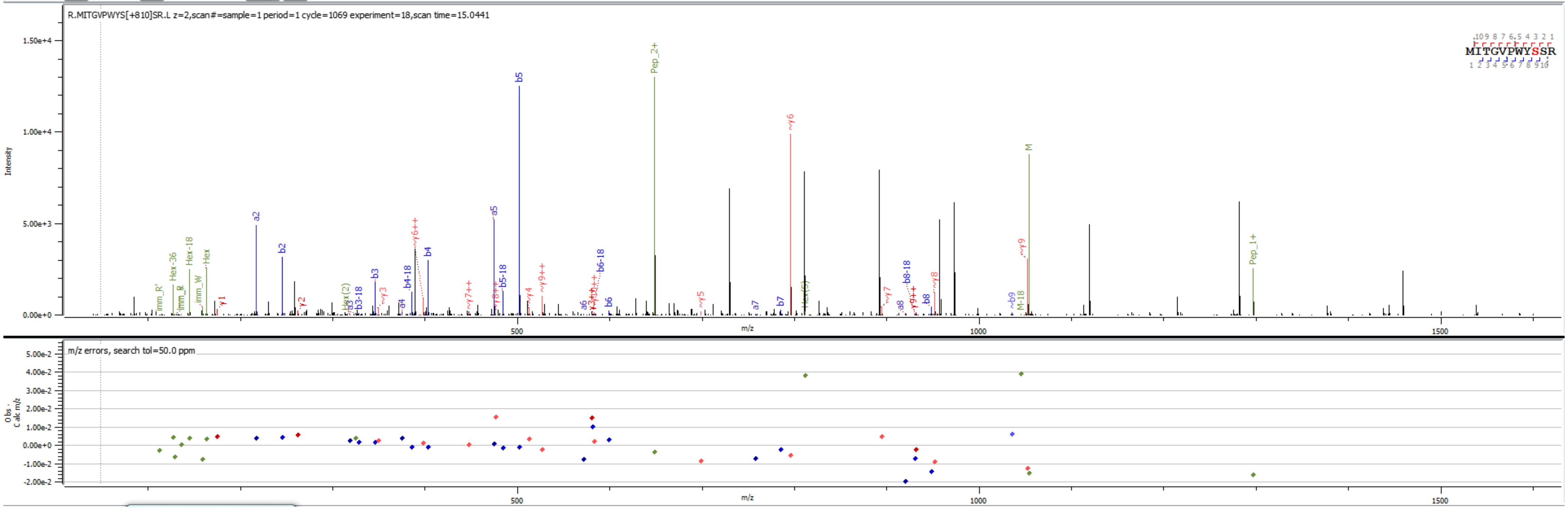

Sample S1 R.MITGVPWYS[+810.264]SR.L

Peptides

PID	Prot. Rank	Pos.	Sequence	Mods (variable)	Glycans	Score	elta Mo Score	z	Obs. m/z	Calc. m/z	ppm err.	Off-By-X	Obs. MH	Calc. MH	Cleavage	Glycans Pos.	Protein Name	Prot. Id	Scan Time	
1 | 187...	2	91	R.MITGVPWYS[+810.26412]SR.L	S9(OGlycan / 810.2641)	Hex(5)	726.5	1.2	3	702.9597	702.9731	-19.03		2106.8645	2106.9046	Specific	9	>sp|P32612|PAU2_YEAST Seripauperin-2 OS=Saccharomyces cerevisiae (strain ATCC 2...	3410	15.0671	sample=1 perioc

Proteins

Prot. Rank	Protein Name	|Log Prob|	Best |Log Prob|	Best Score	# Spectra	# Uniq. Peps.	# Mod Peps.	% Cov.	# AAs	Intensity
1 | 1	>sp|P43575|PAU5_YEAST Seripauperin-5 OS=Saccharomyces cerevisiae (strain ATCC 204508 / S288c) OX=559...	327.47	16.74	1004.1	632	70	67	68.9	122	3.742e+7
2 | 2	>sp|P32612|PAU2_YEAST Seripauperin-2 OS=Saccharomyces cerevisiae (strain ATCC 204508 / S288c) OX=559...	178.33	13.24	726.5	270	60	58	58.3	120	7.688e+6

Spectrum (double click to dock / undock)

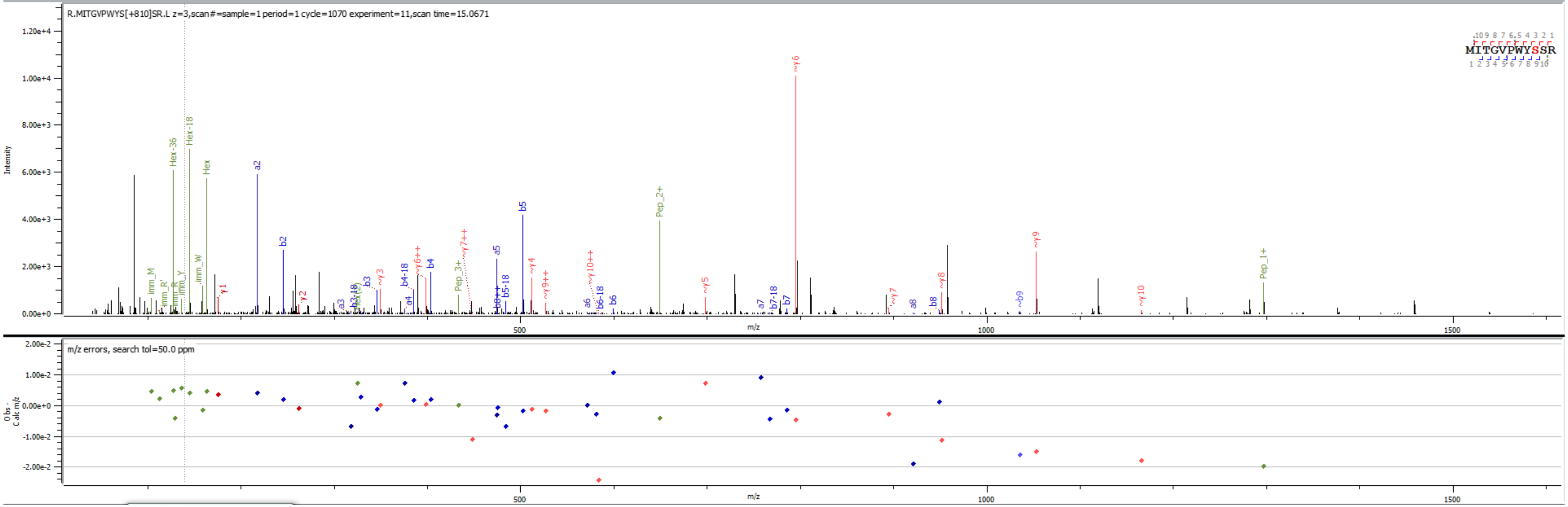

Sample S1 R.MITGVPWYS[+810.264]SR.L

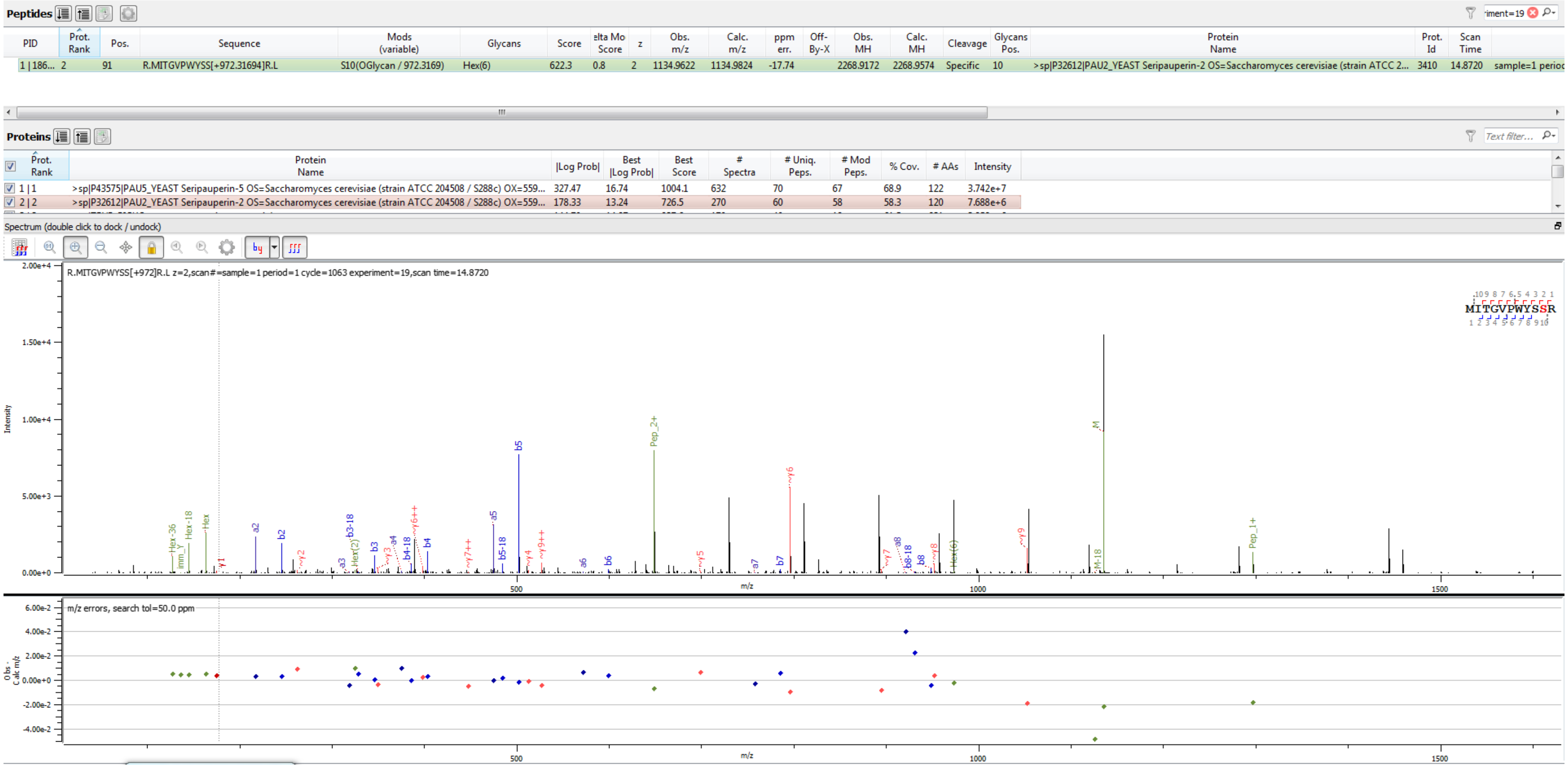

Sample S1 R.MITGVPWYS[+972.317]SR.L

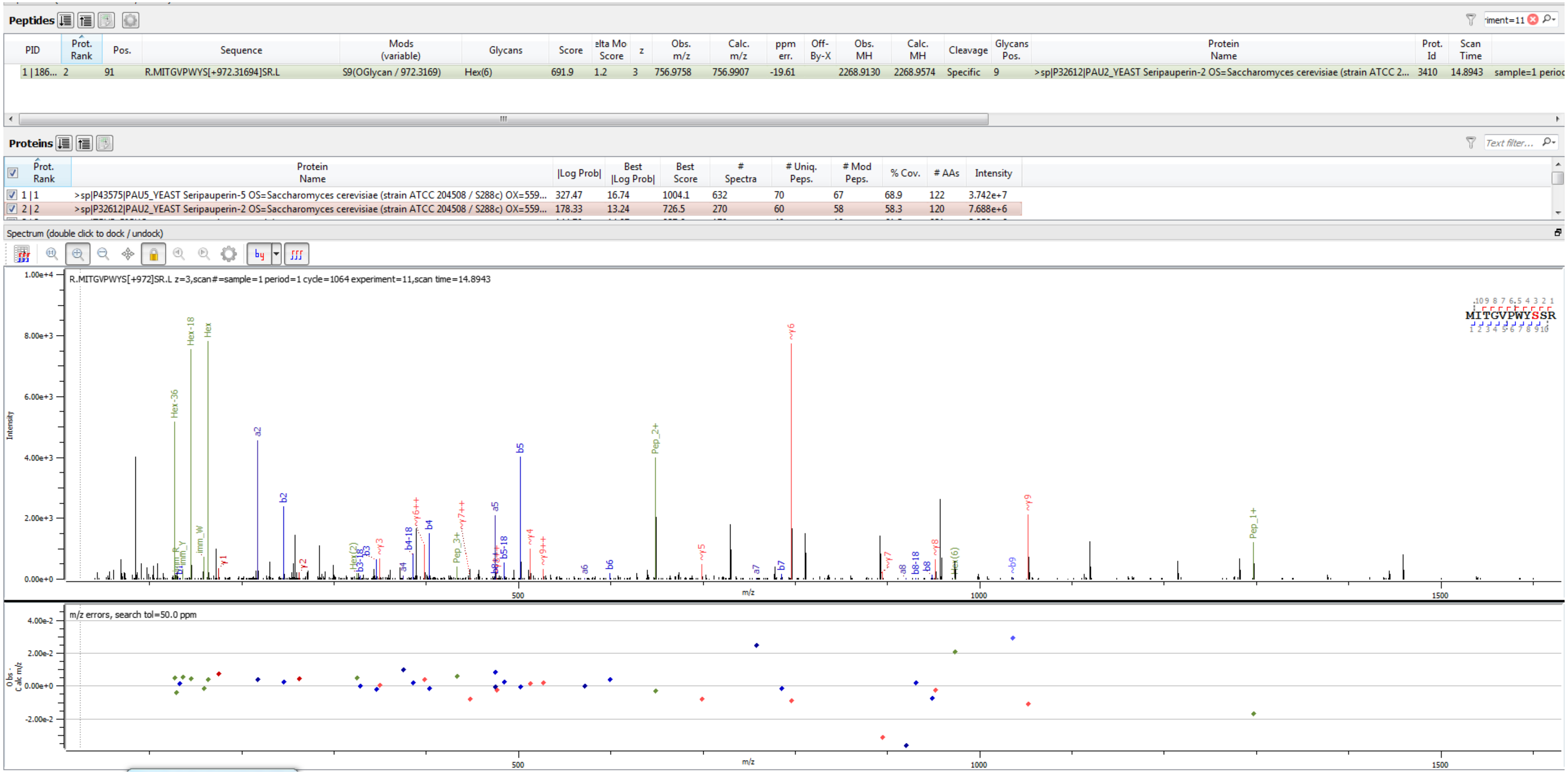

Sample S1 R.MITGVPWYS[+972.317]SR.L

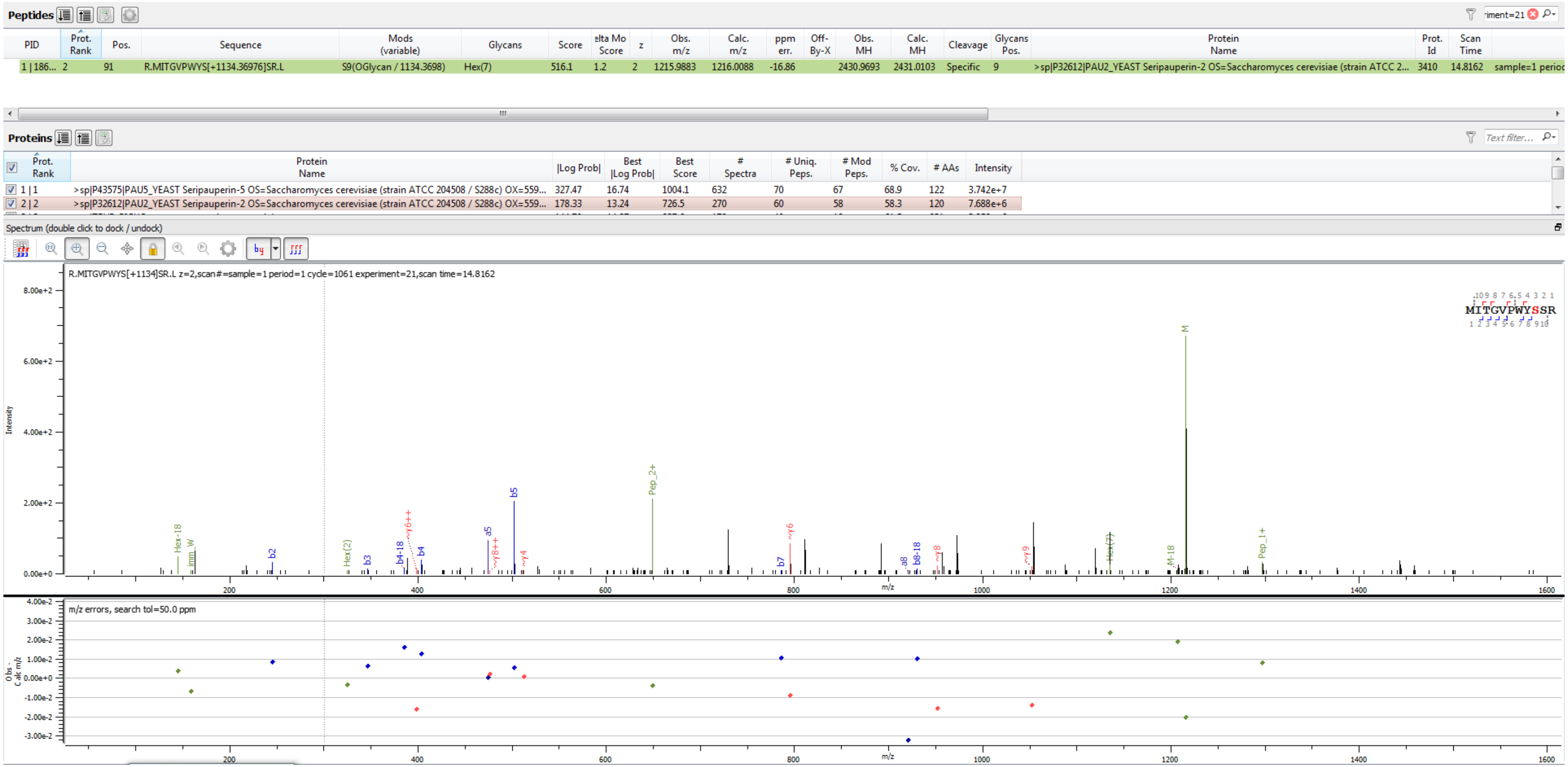

Sample S1 R.MITGVPWYS[+1134.370]SR.L

Peptides

PID	Prot. Rank	Pos.	Sequence	Mods (variable)	Glycans	Score	elta Mo Score	z	Obs. m/z	Calc. m/z	ppm err.	Off-By-X	Obs. MH	Calc. MH	Cleavage	Glycans Pos.	Protein Name	Prot. Id	Scan Time	
1 | 132...	1	91	R.MITGVPWYS[+1134.36976]SR.L	S9(OGlycan / 1134.3698)	Hex(7)	636.2	1.2	3	810.9955	811.0083	-15.75		2430.9720	2431.0103	Specific	9	>sp|Q3E770|PAU9_YEAST Seripauperin-9 OS=Saccharomyces cerevisiae (strain ATCC 2...	3461	14.7986	sample=1 perioc

Proteins

Prot. Rank	Protein Name	|Log Prob|	Best |Log Prob|	Best Score	# Spectra	# Uniq. Peps.	# Mod Peps.	% Cov.	# AAs	Intensity
1 | 1	>sp|Q3E770|PAU9_YEAST Seripauperin-9 OS=Saccharomyces cerevisiae (strain ATCC 204508 / S288c) OX=559...	288.60	16.66	988.6	452	61	56	60.8	120	1.023e+7
2 | 2	>sp|Q07987|PAU23_YEAST Seripauperin-23 OS=Saccharomyces cerevisiae (strain ATCC 204508 / S288c) OX=5...	160.38	13.28	718.6	124	39	36	43.5	124	2.323e+6
3 | 3	>sp|P43575|PAU5_YEAST Seripauperin-5 OS=Saccharomyces cerevisiae (strain ATCC 204508 / S288c) OX=559...	142.55	14.15	737.1	167	35	33	42.6	122	6.136e+6
4 | 4	>sp|K2C1_HUMAN|(Common contaminant protein)	101.54	13.43	953.1	37	11	0	20.4	643	1.801e+5

Spectrum (double click to dock / undock)

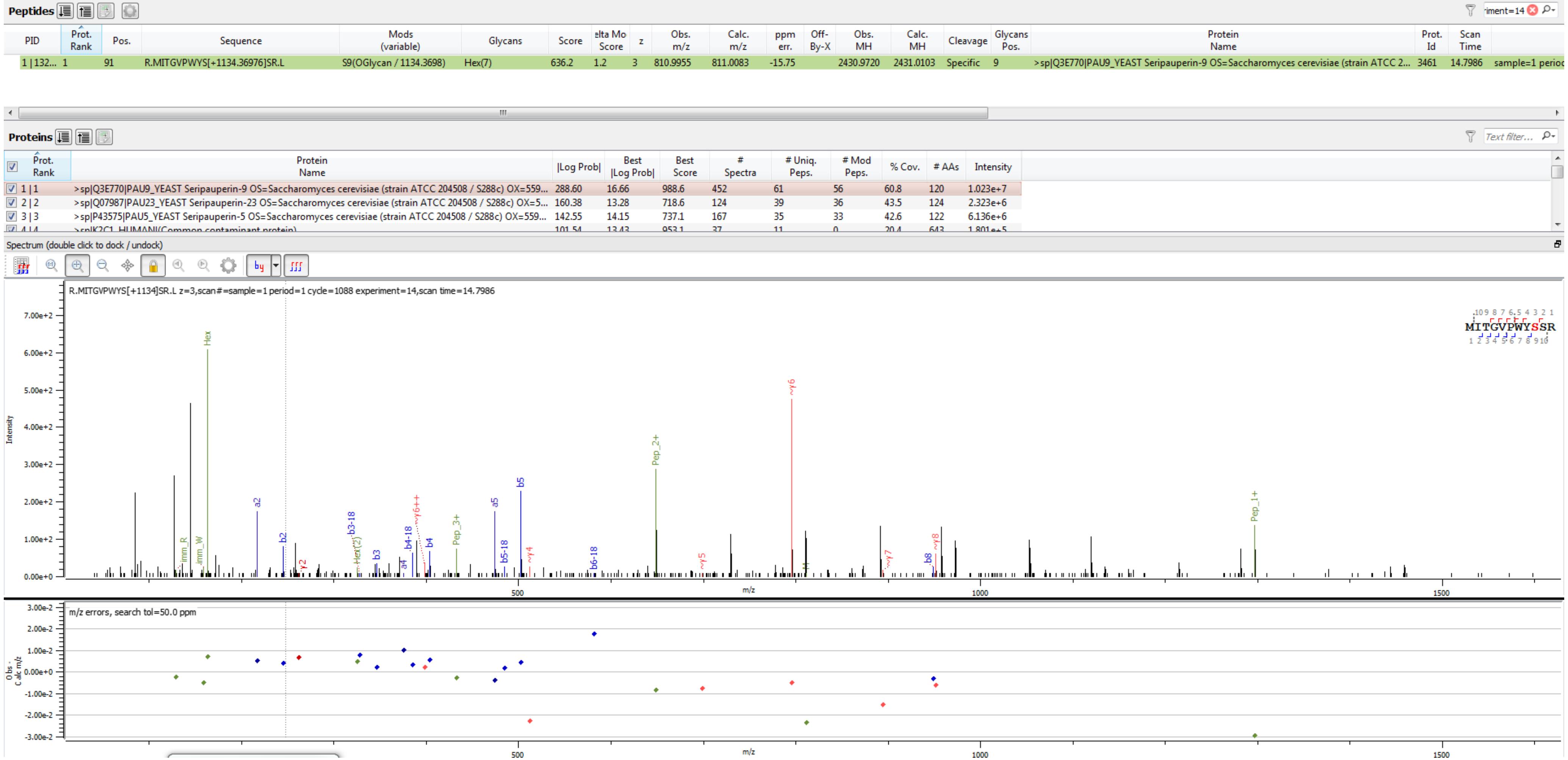

Sample S6 R.MITGVPWYS[+1134.370]SR.L

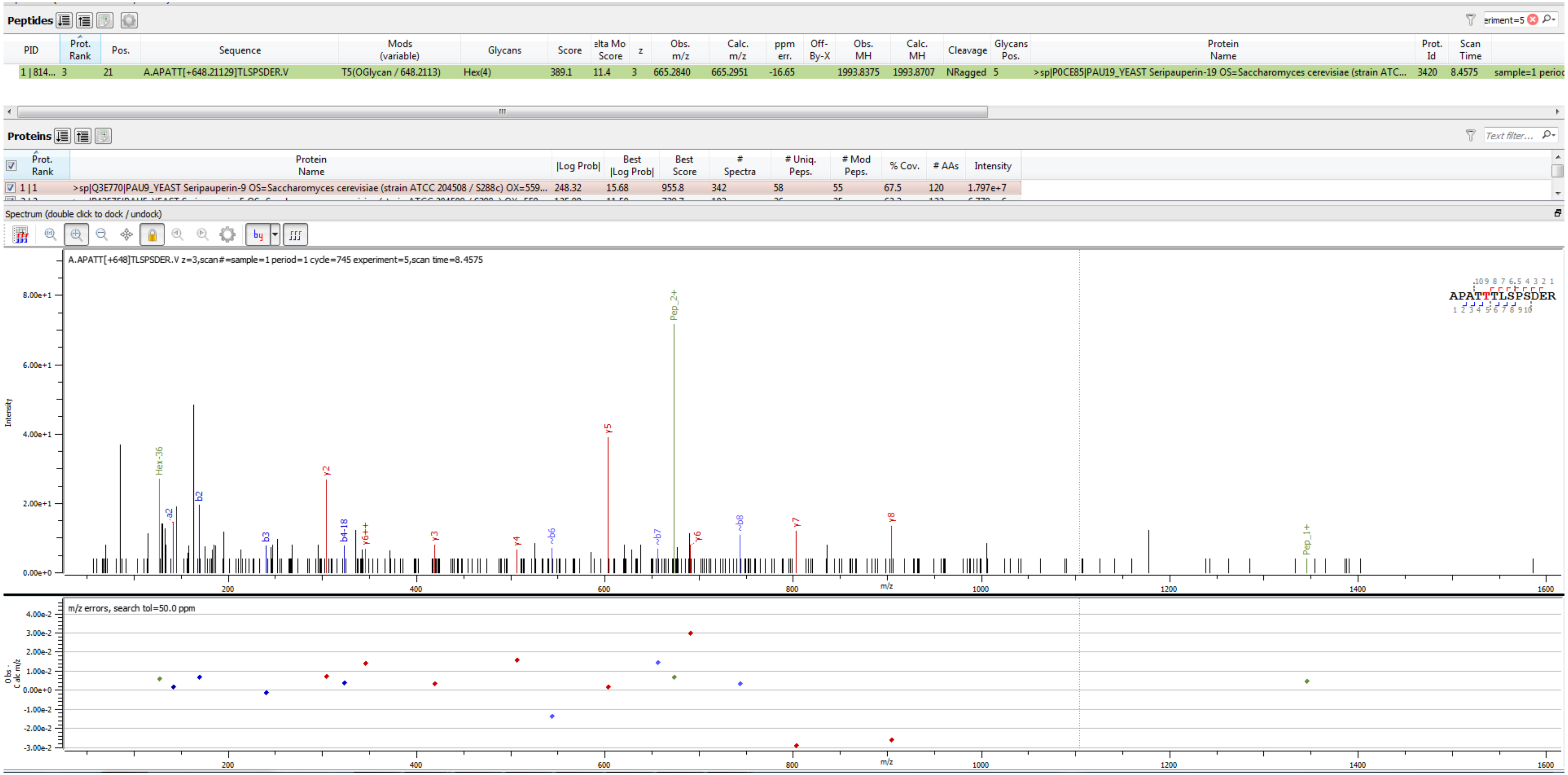

Sample S3 A.APATT[+648.211]TLSPSDER.V

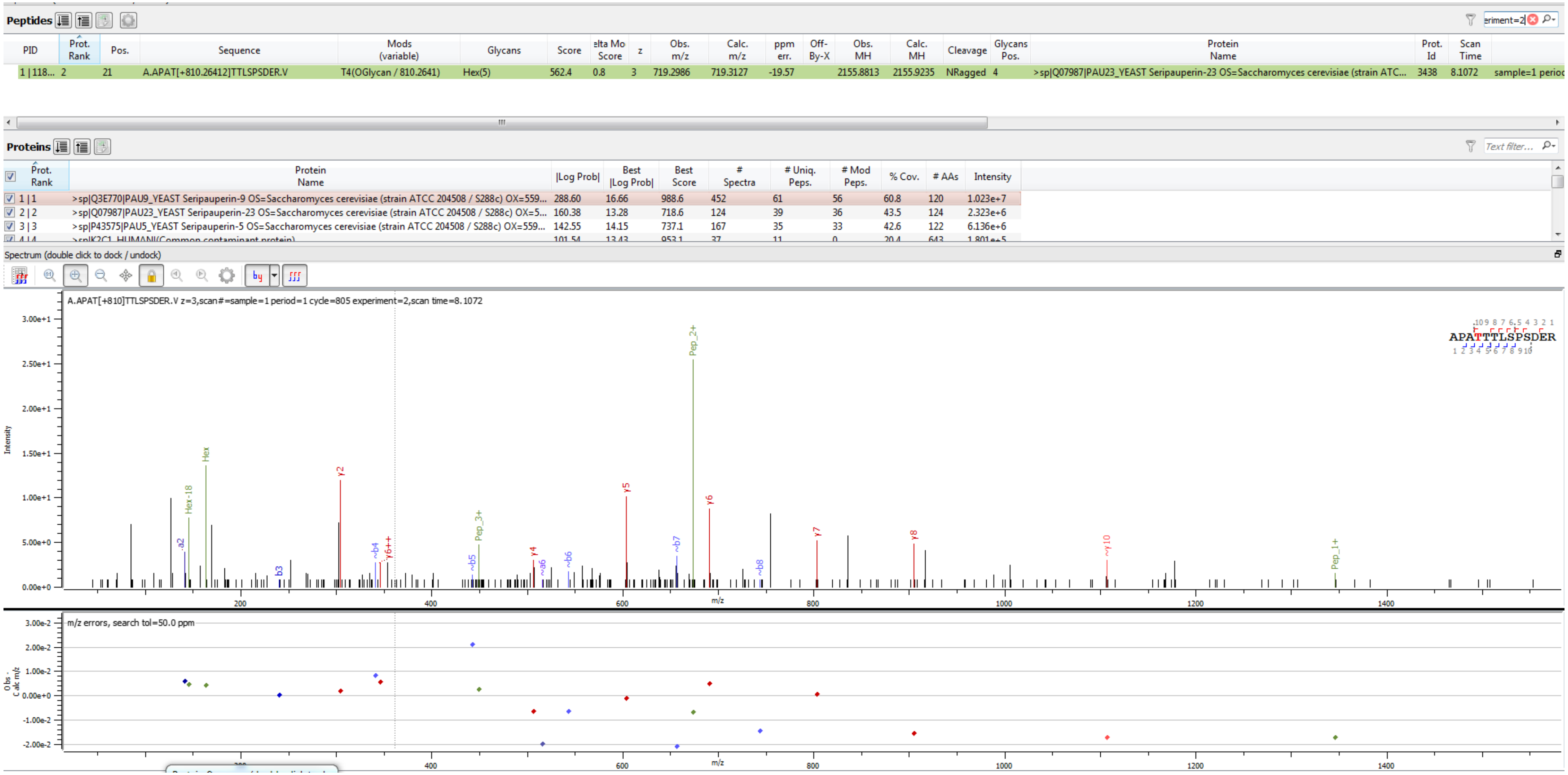

Sample S6 A.APAT[+810.264]TTLSPSDER.V

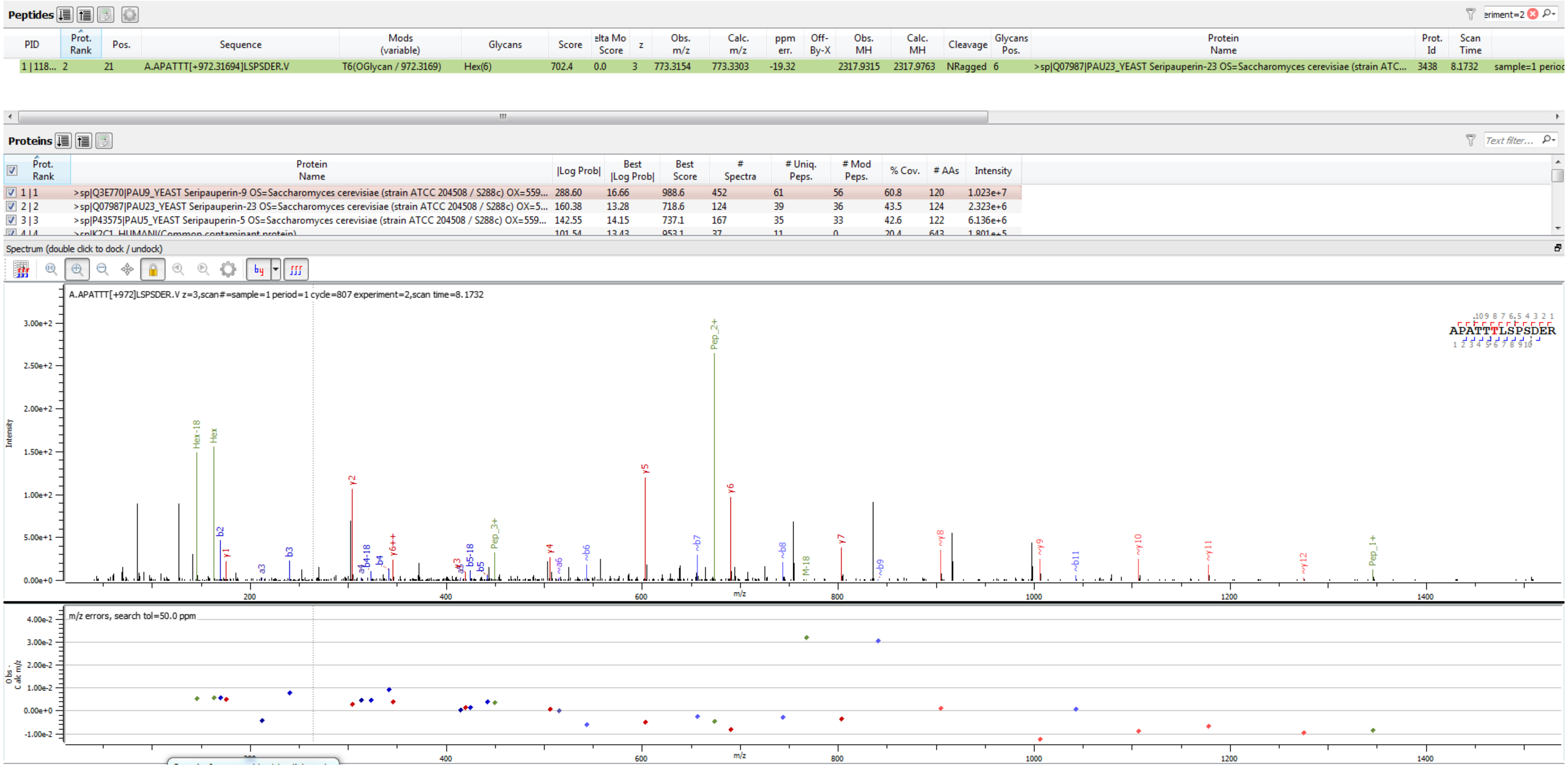

Sample S6 A.APATTT[+972.317]LSPSDER.V

Peptides

PID	Prot. Rank	Pos.	Sequence	Mods (variable)	Glycans	Score	elta Mo Score	z	Obs. m/z	Calc. m/z	ppm err.	Off-By-X	Obs. MH	Calc. MH	Cleavage	Glycans Pos.	Protein Name	Prot. Id	Scan Time	
1 | 163...	12	237	Q.GNTITVQTT[+1296.42259]FVQR.F	T9(OGlycan / 1296.4226)	Hex(8)	776.9	0.0	3	921.0549	921.0726	-19.17		2761.1502	2761.2031	NRagged	9	>sp|P17260|KRE1_YEAST Protein KRE1 OS=Saccharomyces cerevisiae (strain ATCC 204...	1763	11.8741	sample=1 perioc

Proteins

Prot. Rank	Protein Name	|Log Prob|	Best |Log Prob|	Best Score	# Spectra	# Uniq. Peps.	# Mod Peps.	% Cov.	# AAs	Intensity
10 | 10	>sp|Q03674|PLB2_YEAST Lysophospholipase 2 OS=Saccharomyces cerevisiae (strain ATCC 204508 / S288c) O...	11.70	6.05	597.1	5	2	0	3.5	706	1.207e+4
11 | 11	>sp|Q03180|PIR3_YEAST Cell wall mannoprotein PIR3 OS=Saccharomyces cerevisiae (strain ATCC 204508 / S2...	10.18	5.74	637.9	17	11	11	5.8	325	5.629e+4
12 | 12	>sp|P17260|KRE1_YEAST Protein KRE1 OS=Saccharomyces cerevisiae (strain ATCC 204508 / S288c) OX=55929...	9.22	9.16	776.9	3	2	2	7.7	313	1.120e+4
13 | 13	>sp|Q05164|HPF1_YEAST Haze protective factor 1 OS=Saccharomyces cerevisiae (strain ATCC 204508 / S288c)...	8.27	8.22	749.3	4	1	0	1.1	967	1.081e+4

Spectrum (double click to dock / undock)

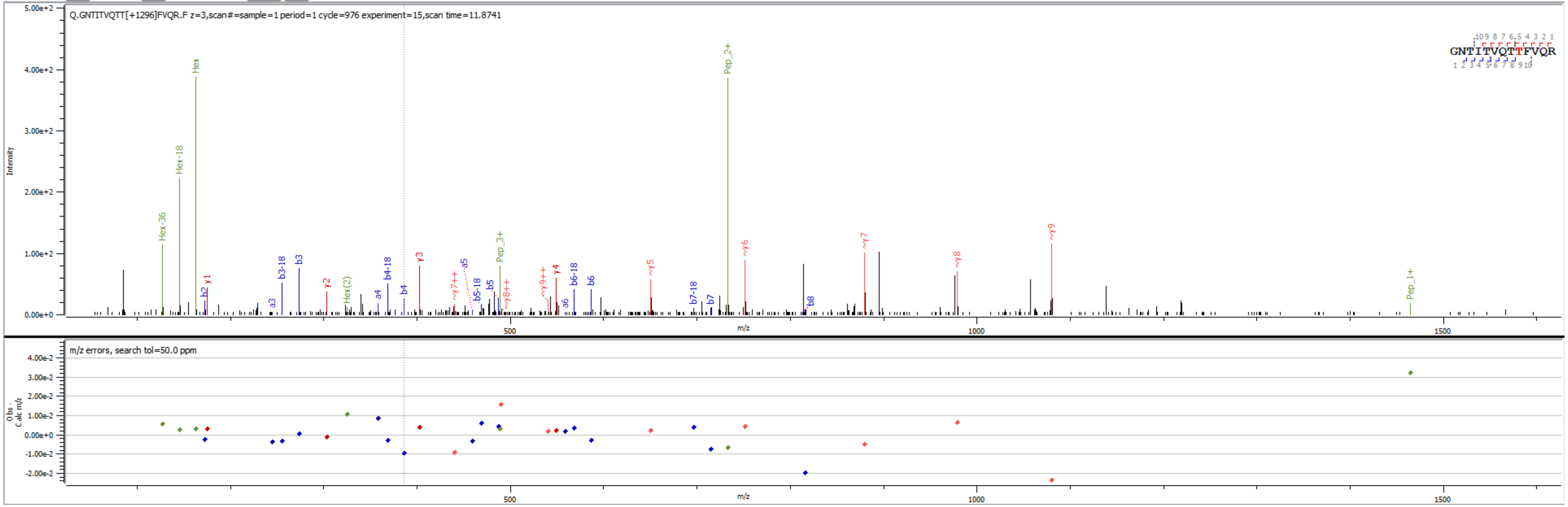

Sample S2 Q.GNTITVQTT[+1296.423]FVQR.F

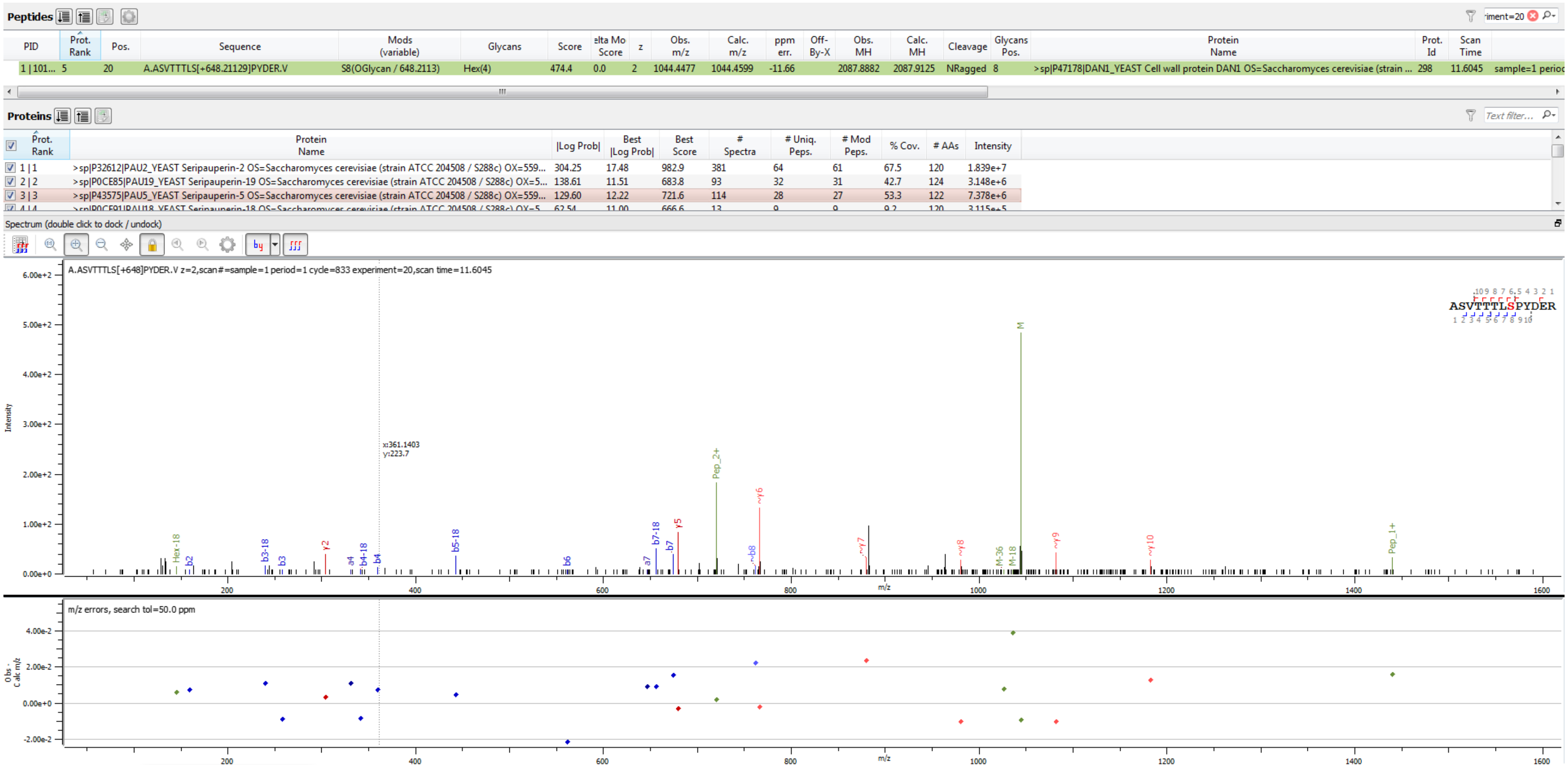

Peptides

PID	Prot. Rank	Pos.	Sequence	Mods (variable)	Glycans	Score	elta Mo Score	z	Obs. m/z	Calc. m/z	ppm err.	Off-By-X	Obs. MH	Calc. MH	Cleavage	Glycans Pos.	Protein Name	Prot. Id	Scan Time	
1 | 101...	5	20	A.ASVTTTLS[+648.21129]PYDER.V	S8(OGlycan / 648.2113)	Hex(4)	474.4	0.0	2	1044.4477	1044.4599	-11.66		2087.8882	2087.9125	NRagged	8	>sp|P47178|DAN1_YEAST Cell wall protein DAN1 OS=Saccharomyces cerevisiae (strain ...	298	11.6045	sample=1 perioc

Proteins

Prot. Rank	Protein Name	|Log Prob|	Best |Log Prob|	Best Score	# Spectra	# Uniq. Peps.	# Mod Peps.	% Cov.	# AAs	Intensity
1 | 1	>sp|P32612|PAU2_YEAST Seripauperin-2 OS=Saccharomyces cerevisiae (strain ATCC 204508 / S288c) OX=559...	304.25	17.48	982.9	381	64	61	67.5	120	1.839e+7
2 | 2	>sp|P0CE85|PAU19_YEAST Seripauperin-19 OS=Saccharomyces cerevisiae (strain ATCC 204508 / S288c) OX=5...	138.61	11.51	683.8	93	32	31	42.7	124	3.148e+6
3 | 3	>sp|P43575|PAU5_YEAST Seripauperin-5 OS=Saccharomyces cerevisiae (strain ATCC 204508 / S288c) OX=559...	129.60	12.22	721.6	114	28	27	53.3	122	7.378e+6
4 | 4	>sp|P0CE91|PAU18_YEAST Seripauperin-18 OS=Saccharomyces cerevisiae (strain ATCC 204508 / S288c) OX=5...	62.54	11.00	666.6	13	9	9	9.2	120	3.115e+5

Sample S4 A.ASVTTTLS[+648.211]PYDER.V

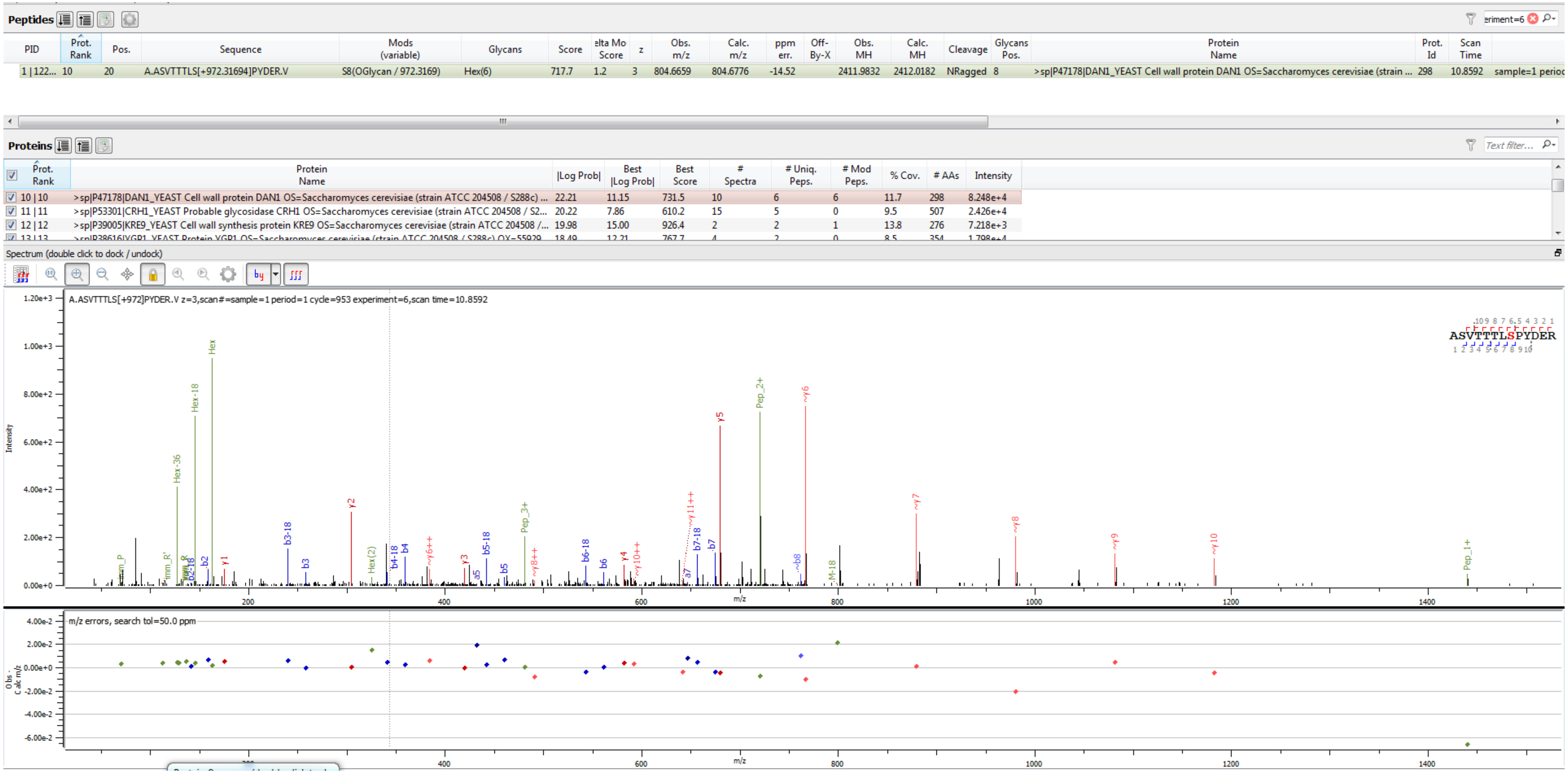

PID	Prot. Rank	Pos.	Sequence	Mods (variable)	Glycans	Score	elta Mo Score	z	Obs. m/z	Calc. m/z	ppm err.	Off-By-X	Obs. MH	Calc. MH	Cleavage	Glycans Pos.	Protein Name	Prot. Id	Scan Time	
1 | 122...	10	20	A.ASVTTTLS[+972.31694]PYDER.V	S8(OGlycan / 972.3169)	Hex(6)	717.7	1.2	3	804.6659	804.6776	-14.52		2411.9832	2412.0182	NRagged	8	>sp|P47178|DAN1_YEAST Cell wall protein DAN1 OS=Saccharomyces cerevisiae (strain ...	298	10.8592	sample=1 perioc

Prot. Rank	Protein Name	|Log Prob|	Best |Log Prob|	Best Score	# Spectra	# Uniq. Peps.	# Mod Peps.	% Cov.	# AAs	Intensity
10 | 10	>sp|P47178|DAN1_YEAST Cell wall protein DAN1 OS=Saccharomyces cerevisiae (strain ATCC 204508 / S288c) ...	22.21	11.15	731.5	10	6	6	11.7	298	8.248e+4
11 | 11	>sp|P53301|CRH1_YEAST Probable glycosidase CRH1 OS=Saccharomyces cerevisiae (strain ATCC 204508 / S2...	20.22	7.86	610.2	15	5	0	9.5	507	2.426e+4
12 | 12	>sp|P39005|KRE9_YEAST Cell wall synthesis protein KRE9 OS=Saccharomyces cerevisiae (strain ATCC 204508 /...	19.98	15.00	926.4	2	2	1	13.8	276	7.218e+3
13 | 13	>sp|P38616|YGP1_YEAST Protein YGP1 OS=Saccharomyces cerevisiae (strain ATCC 204508 / S288c) OX=55929	18.49	12.21	767.7	4	2	0	8.5	354	1.798e+4

Sample S6 A.ASVTTTLS[+972.317]PYDER.V

Peptides

PID	Prot. Rank	Pos.	Sequence	Mods (variable)	Glycans	Score	elta Mo Score	z	Obs. m/z	Calc. m/z	ppm err.	Off-By-X	Obs. MH	Calc. MH	Cleavage	Glycans Pos.	Protein Name	Prot. Id	Scan Time	
1 | 356...	2	116	K.DGIYT[+324.10565]AIPK.-	T5(OGlycan / 324.1056)	Hex(2)	683.8	321.7	2	651.3173	651.3216	-6.59		1301.6273	1301.6358	Specific	5	>sp|P0CE85|PAU19_YEAST Seripauperin-19 OS=Saccharomyces cerevisiae (strain ATC...	3420	13.3804	sample=1 perioc

Proteins

Prot. Rank	Protein Name	|Log Prob|	Best |Log Prob|	Best Score	# Spectra	# Uniq. Peps.	# Mod Peps.	% Cov.	# AAs	Intensity
1 | 1	>sp|P32612|PAU2_YEAST Seripauperin-2 OS=Saccharomyces cerevisiae (strain ATCC 204508 / S288c) OX=559...	304.25	17.48	982.9	381	64	61	67.5	120	1.839e+7
2 | 2	>sp|P0CE85|PAU19_YEAST Seripauperin-19 OS=Saccharomyces cerevisiae (strain ATCC 204508 / S288c) OX=5...	138.61	11.51	683.8	93	32	31	42.7	124	3.148e+6
3 | 3	>sp|P43575|PAU5_YEAST Seripauperin-5 OS=Saccharomyces cerevisiae (strain ATCC 204508 / S288c) OX=559...	129.60	12.22	721.6	114	28	27	53.3	122	7.378e+6
4 | 4	>sp|P0CF01|PAU18_YEAST Seripauperin-18 OS=Saccharomyces cerevisiae (strain ATCC 204508 / S288c) OX=5...	62.54	11.00	666.6	13	9	9	9.2	120	3.115e+5

Spectrum (double click to dock / undock)

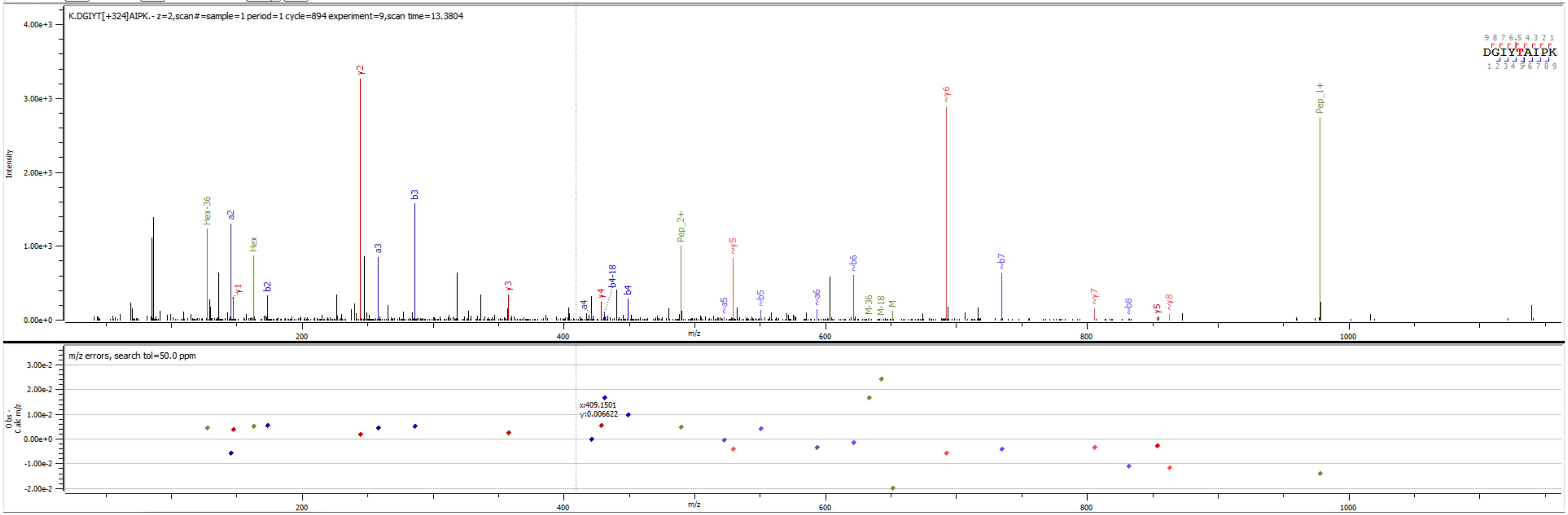

Sample S4 K.DGIYT[+324.106]AIPK.-

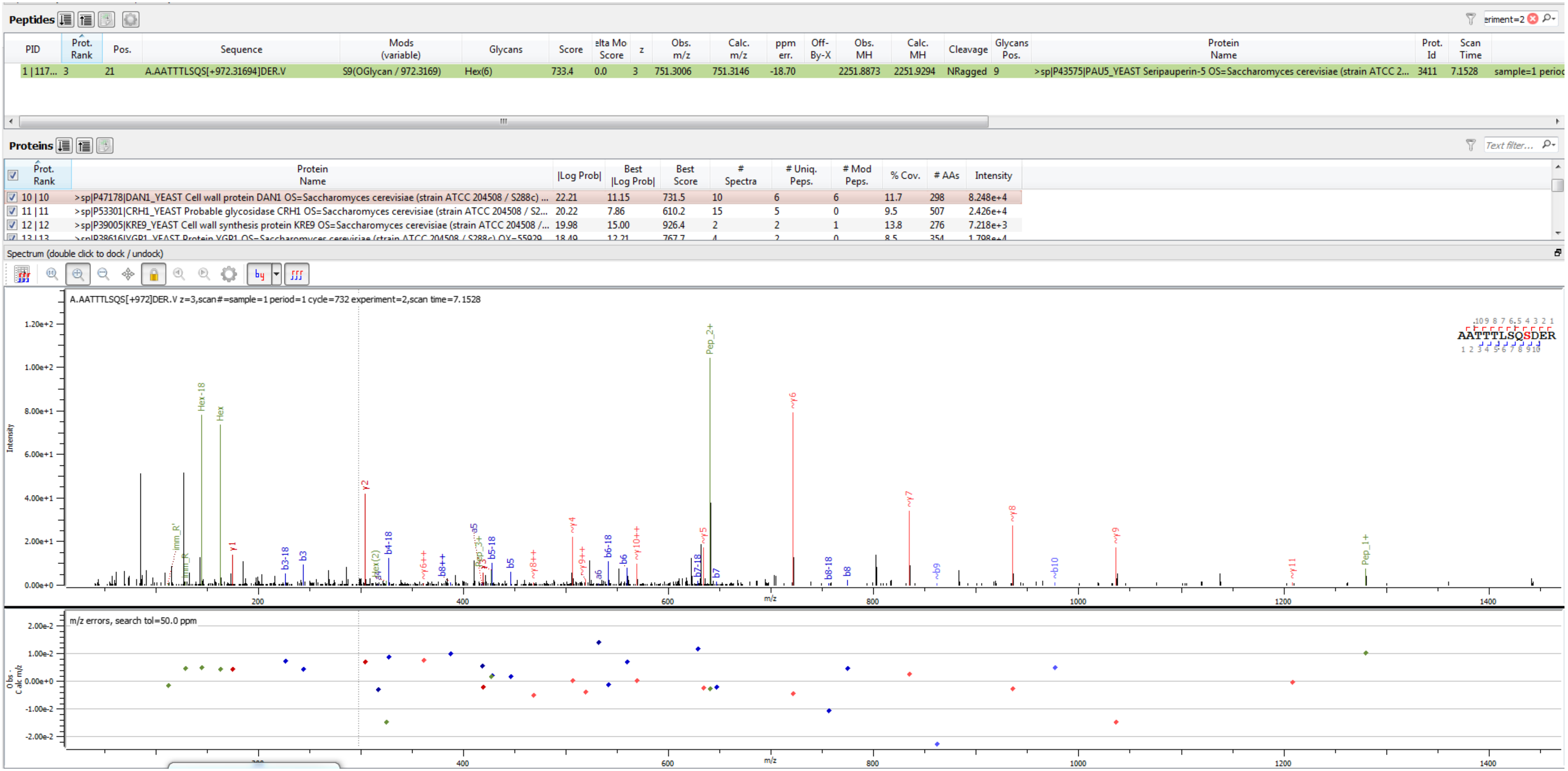

Sample S6 A.AATTTLSQS[+972.317]DER.V

Peptides

PID	Prot. Rank	Pos.	Sequence	Mods (variable)	Glycans	Score	elta Mo Score	z	Obs. m/z	Calc. m/z	ppm err.	Off-By-X	Obs. MH	Calc. MH	Cleavage	Glycans Pos.	Protein Name	Prot. Id	Scan Time	
1 | 106...	14	109	K.ALSKDGIYT[+648.21129]IAN.-	T9(OGlycan / 648.2113)	Hex(4)	519.5	8.6	2	957.4411	957.4461	-5.16		1913.8750	1913.8849	Specific	9	>sp|P53427|PAU4_YEAST Seripauperin-4 OS=Saccharomyces cerevisiae (strain ATCC 2...	3439	12.3100	sample=1 perioc

Proteins

Prot. Rank	Protein Name	|Log Prob|	Best |Log Prob|	Best Score	# Spectra	# Uniq. Peps.	# Mod Peps.	% Cov.	# AAs	Intensity
1 | 1	>sp|P32612|PAU2_YEAST Seripauperin-2 OS=Saccharomyces cerevisiae (strain ATCC 204508 / S288c) OX=559...	304.25	17.48	982.9	381	64	61	67.5	120	1.839e+7
2 | 2	>sp|P0CE85|PAU19_YEAST Seripauperin-19 OS=Saccharomyces cerevisiae (strain ATCC 204508 / S288c) OX=5...	138.61	11.51	683.8	93	32	31	42.7	124	3.148e+6
3 | 3	>sp|P43575|PAU5_YEAST Seripauperin-5 OS=Saccharomyces cerevisiae (strain ATCC 204508 / S288c) OX=559...	129.60	12.22	721.6	114	28	27	53.3	122	7.378e+6
4 | 4	>sp|P0CE91|PAU18_YEAST Seripauperin-18 OS=Saccharomyces cerevisiae (strain ATCC 204508 / S288c) OX=5...	62.54	11.00	666.6	13	9	9	9.2	120	3.115e+5

Spectrum (double click to dock / undock)

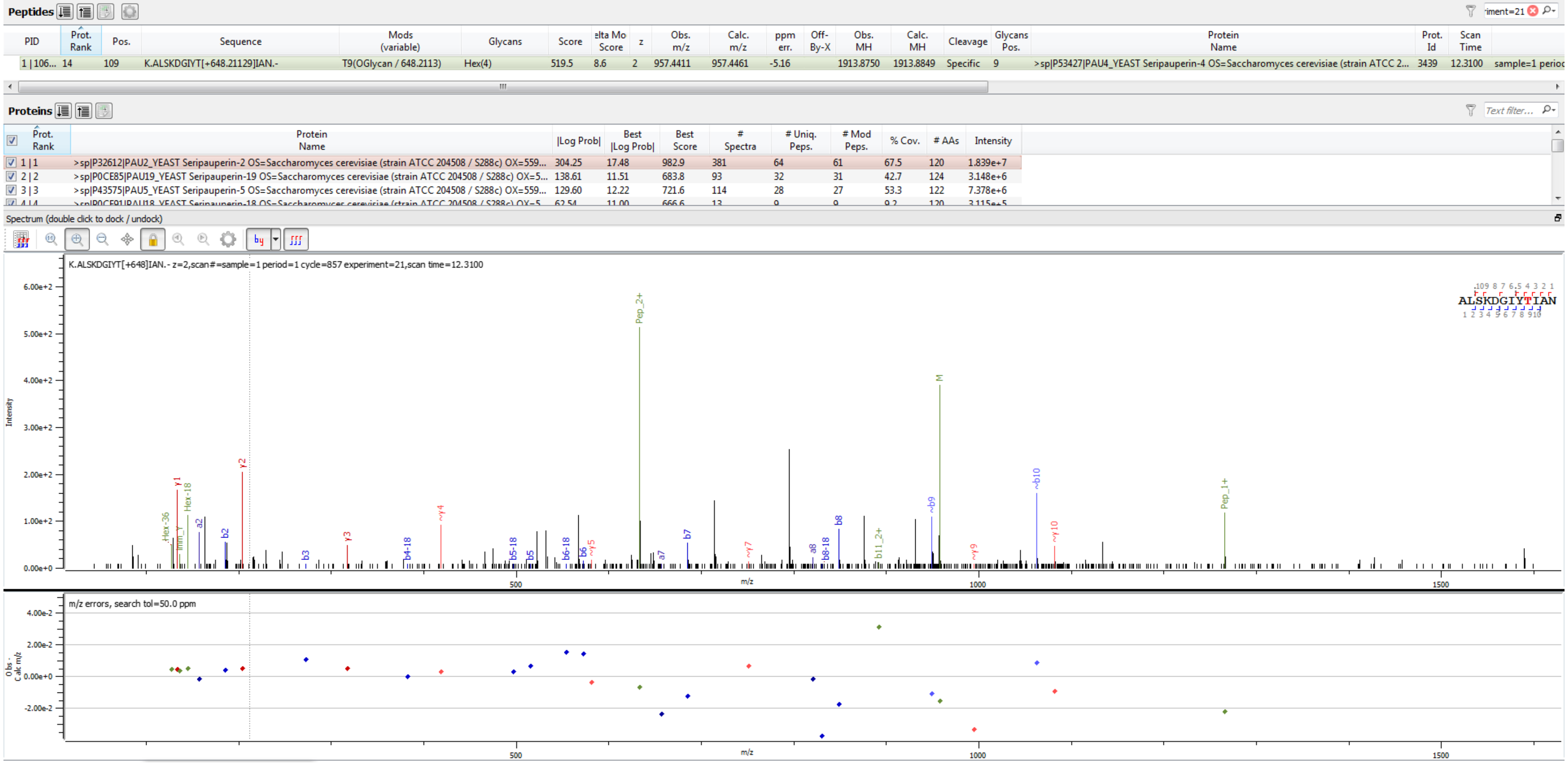

Sample S4 K.ALSKDGIYT[+648.211]IAN.-

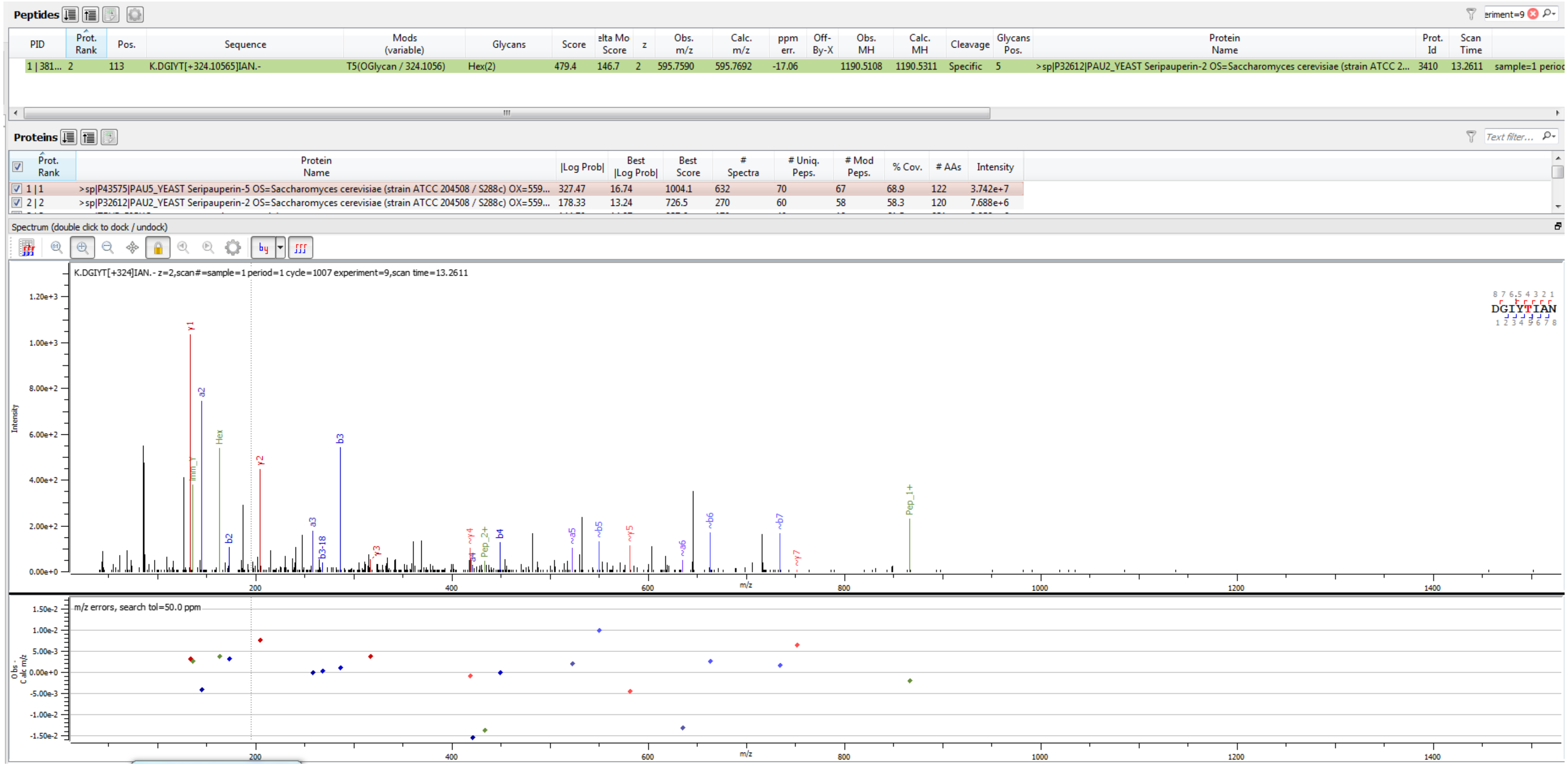

Sample S1 K.DGIYT[+324.106]IAN.-

Peptides

PID	Prot. Rank	Pos.	Sequence	Mods (variable)	Glycans	Score	elta Mo Score	z	Obs. m/z	Calc. m/z	ppm err.	Off-By-X	Obs. MH	Calc. MH	Cleavage	Glycans Pos.	Protein Name	Prot. Id	Scan Time	
1 | 368...	11	24	K.DPWST[+648.21129]LTPS.A	T5(OGlycan / 648.2113)	Hex(4)	395.5	0.0	2	826.3449	826.3458	-1.17		1651.6824	1651.6844	CRagged	5	>sp|Q03180|PIR3_YEAST Cell wall mannoprotein PIR3 OS=Saccharomyces cerevisiae (s...	3365	13.6993	sample=1 perioc

Proteins

Prot. Rank	Protein Name	|Log Prob|	Best |Log Prob|	Best Score	# Spectra	# Uniq. Peps.	# Mod Peps.	% Cov.	# AAs	Intensity
10 | 10	>sp|Q03674|PLB2_YEAST Lysophospholipase 2 OS=Saccharomyces cerevisiae (strain ATCC 204508 / S288c) O...	11.70	6.05	597.1	5	2	0	3.5	706	1.207e+4
11 | 11	>sp|Q03180|PIR3_YEAST Cell wall mannoprotein PIR3 OS=Saccharomyces cerevisiae (strain ATCC 204508 / S2...	10.18	5.74	637.9	17	11	11	5.8	325	5.629e+4
12 | 12	>sp|P17260|KRE1_YEAST Protein KRE1 OS=Saccharomyces cerevisiae (strain ATCC 204508 / S288c) OX=55929...	9.22	9.16	776.9	3	2	2	7.7	313	1.120e+4
13 | 13	>sp|Q05164|HPF1_YEAST Haze protective factor 1 OS=Saccharomyces cerevisiae (strain ATCC 204508 / S288c)...	8.27	8.22	749.3	4	1	0	1.1	967	1.081e+4

Spectrum (double click to dock / undock)

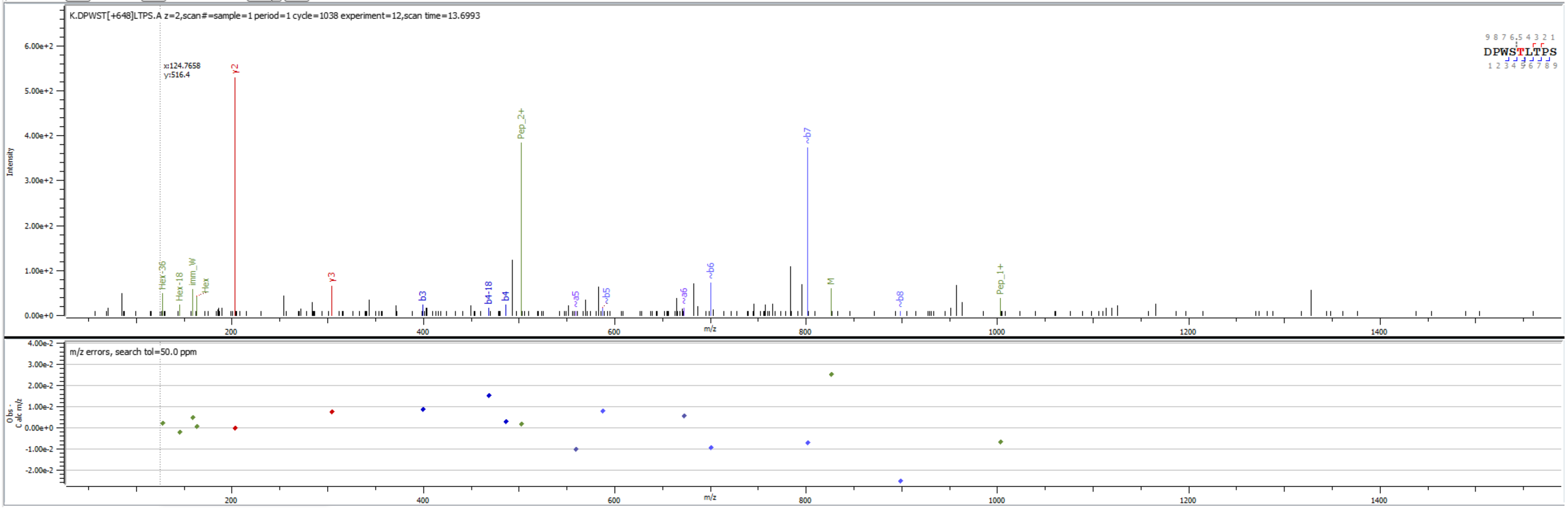

Sample S2 K.DPWST[+648.211]LTPS.A

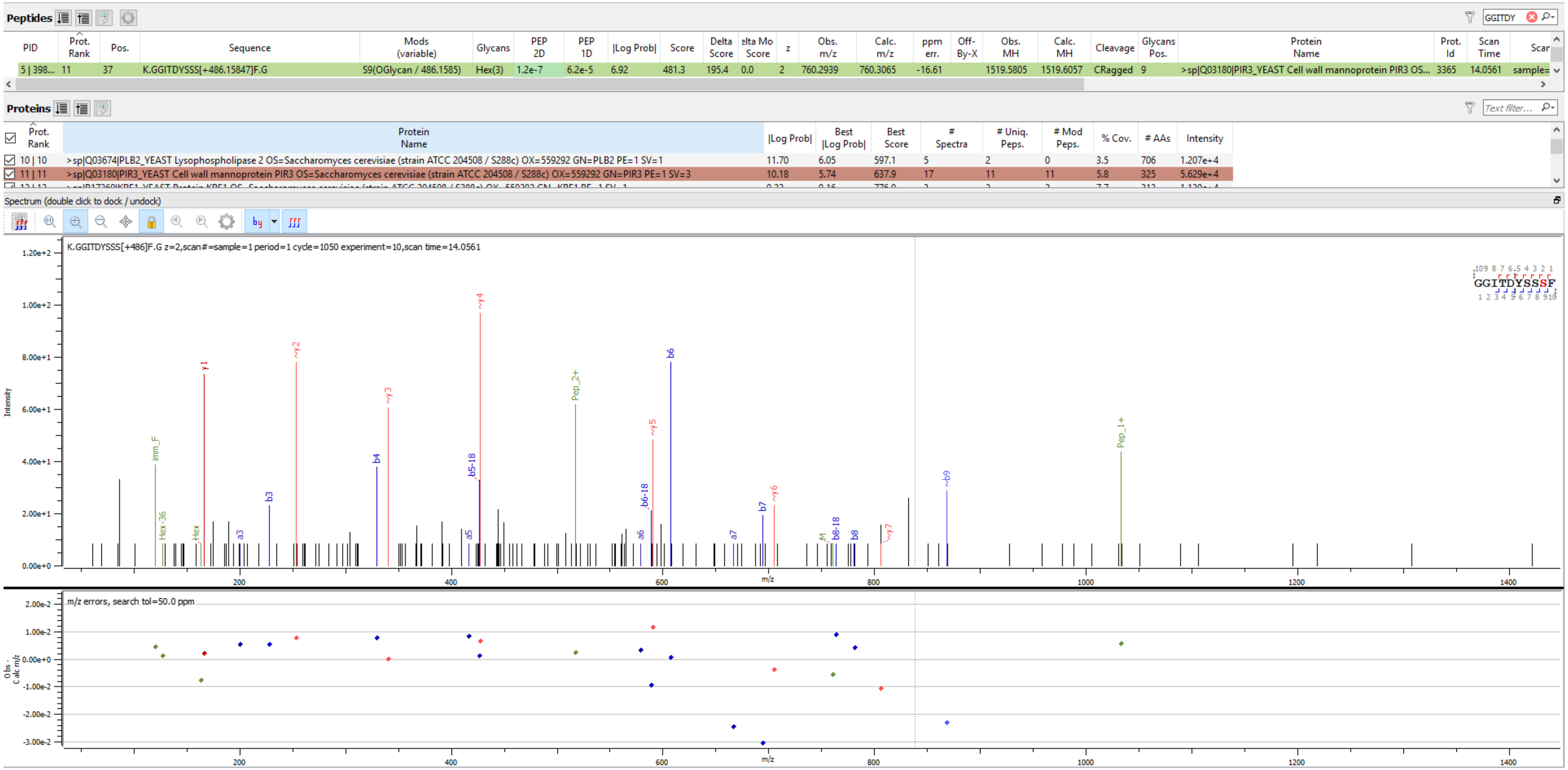

Sample S2 K.GGITDYSSS[+486.158]F.G

Peptides

PID	Prot. Rank	Pos.	Sequence	Mods (variable)	Glycans	Score	elta Mo Score	z	Obs. m/z	Calc. m/z	ppm err.	Off-By-X	Obs. MH	Calc. MH	Cleavage	Glycans Pos.	Protein Name	Prot. Id	Scan Time	
1 | 342...	11	37	K.GGITDYS[+648.21129]SSF.G	S7(OGlycan / 648.2113)	Hex(4)	637.9	0.0	2	841.3211	841.3329	-14.04		1681.6350	1681.6586	CRagged	7	>sp|Q03180|PIR3_YEAST Cell wall mannoprotein PIR3 OS=Saccharomyces cerevisiae (s...	3365	13.4938	sample=1 perioc

Proteins

Prot. Rank	Protein Name	|Log Prob|	Best |Log Prob|	Best Score	# Spectra	# Uniq. Peps.	# Mod Peps.	% Cov.	# AAs	Intensity
10 | 10	>sp|Q03674|PLB2_YEAST Lysophospholipase 2 OS=Saccharomyces cerevisiae (strain ATCC 204508 / S288c) O...	11.70	6.05	597.1	5	2	0	3.5	706	1.207e+4
11 | 11	>sp|Q03180|PIR3_YEAST Cell wall mannoprotein PIR3 OS=Saccharomyces cerevisiae (strain ATCC 204508 / S2...	10.18	5.74	637.9	17	11	11	5.8	325	5.629e+4
12 | 12	>sp|P17260|KRE1_YEAST Protein KRE1 OS=Saccharomyces cerevisiae (strain ATCC 204508 / S288c) OX=55929...	9.22	9.16	776.9	3	2	2	7.7	313	1.120e+4
13 | 13	>sp|Q05164|HPF1_YEAST Haze protective factor 1 OS=Saccharomyces cerevisiae (strain ATCC 204508 / S288c)...	8.27	8.22	749.3	4	1	0	1.1	967	1.081e+4

Spectrum (double click to dock / undock)

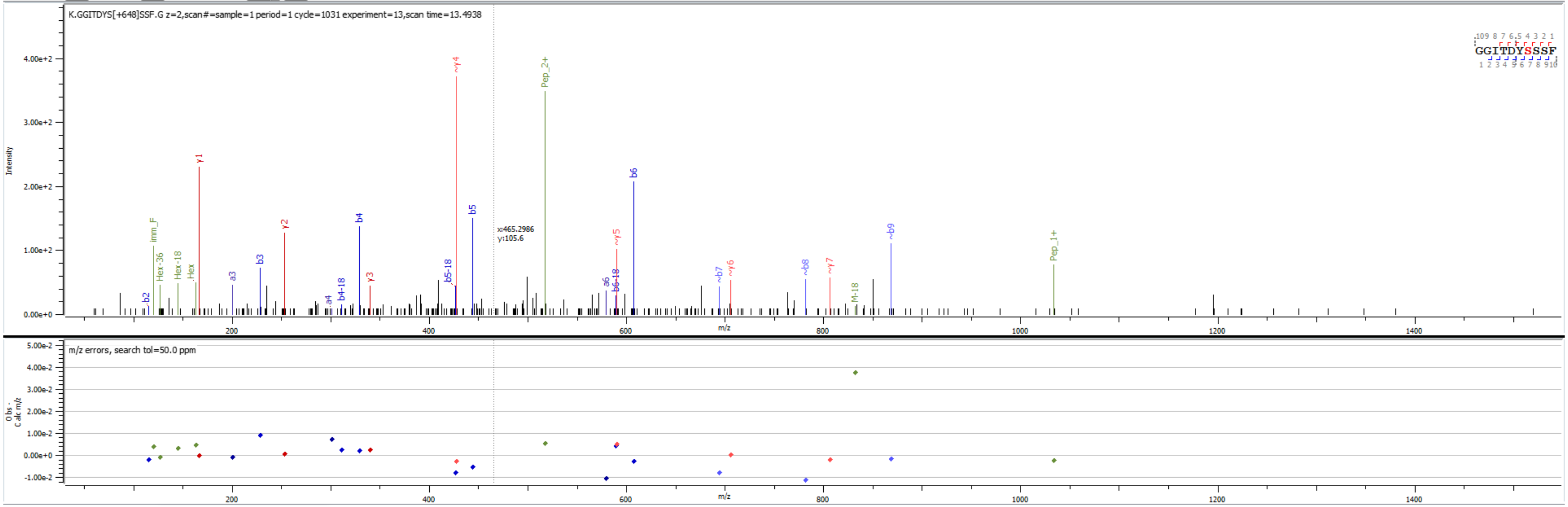

Sample S2 K.GGITDYS[+648.211]SSF.G

Peptides

PID	Prot. Rank	Pos.	Sequence	Mods (variable)	Glycans	Score	elta Mo Score	z	Obs. m/z	Calc. m/z	ppm err.	Off-By-X	Obs. MH	Calc. MH	Cleavage	Glycans Pos.	Protein Name	Prot. Id	Scan Time	
1 | 464...	2	94	R.VITGVPWYST[+324.10565]R.L	T10(OGlycan / 324.1056)	Hex(2)	567.4	0.0	2	801.8850	801.8985	-16.88		1602.7627	1602.7897	Specific	10	>sp|Q07987|PAU23_YEAST Seripauperin-23 OS=Saccharomyces cerevisiae (strain ATC...	3438	14.5391	sample=1 perioc

Proteins

Prot. Rank	Protein Name	|Log Prob|	Best |Log Prob|	Best Score	# Spectra	# Uniq. Peps.	# Mod Peps.	% Cov.	# AAs	Intensity
1 | 1	>sp|Q3E770|PAU9_YEAST Seripauperin-9 OS=Saccharomyces cerevisiae (strain ATCC 204508 / S288c) OX=559...	288.60	16.66	988.6	452	61	56	60.8	120	1.023e+7
2 | 2	>sp|Q07987|PAU23_YEAST Seripauperin-23 OS=Saccharomyces cerevisiae (strain ATCC 204508 / S288c) OX=5...	160.38	13.28	718.6	124	39	36	43.5	124	2.323e+6
3 | 3	>sp|P43575|PAU5_YEAST Seripauperin-5 OS=Saccharomyces cerevisiae (strain ATCC 204508 / S288c) OX=559...	142.55	14.15	737.1	167	35	33	42.6	122	6.136e+6
4 | 4	>sp|K2C1_HUMAN|(Common contaminant protein)	101.54	13.43	953.1	37	11	0	20.4	643	1.801e+5

Spectrum (double click to dock / undock)

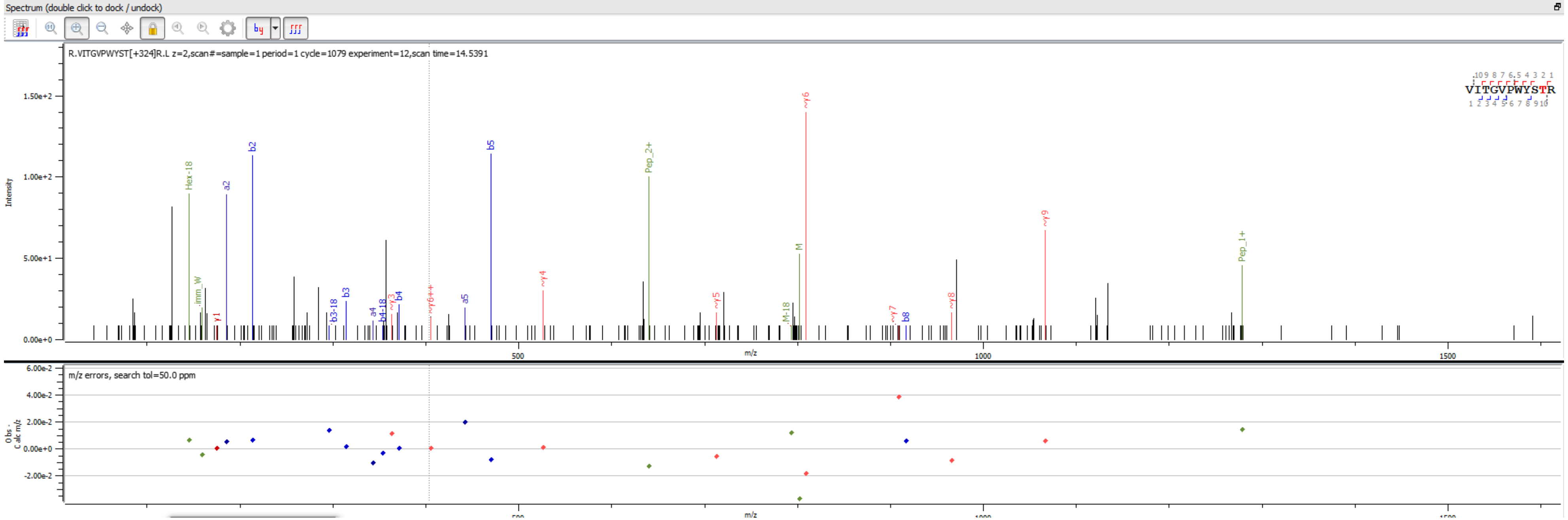

Sample S6 R.VITGVPWYST[+324.106]R.L

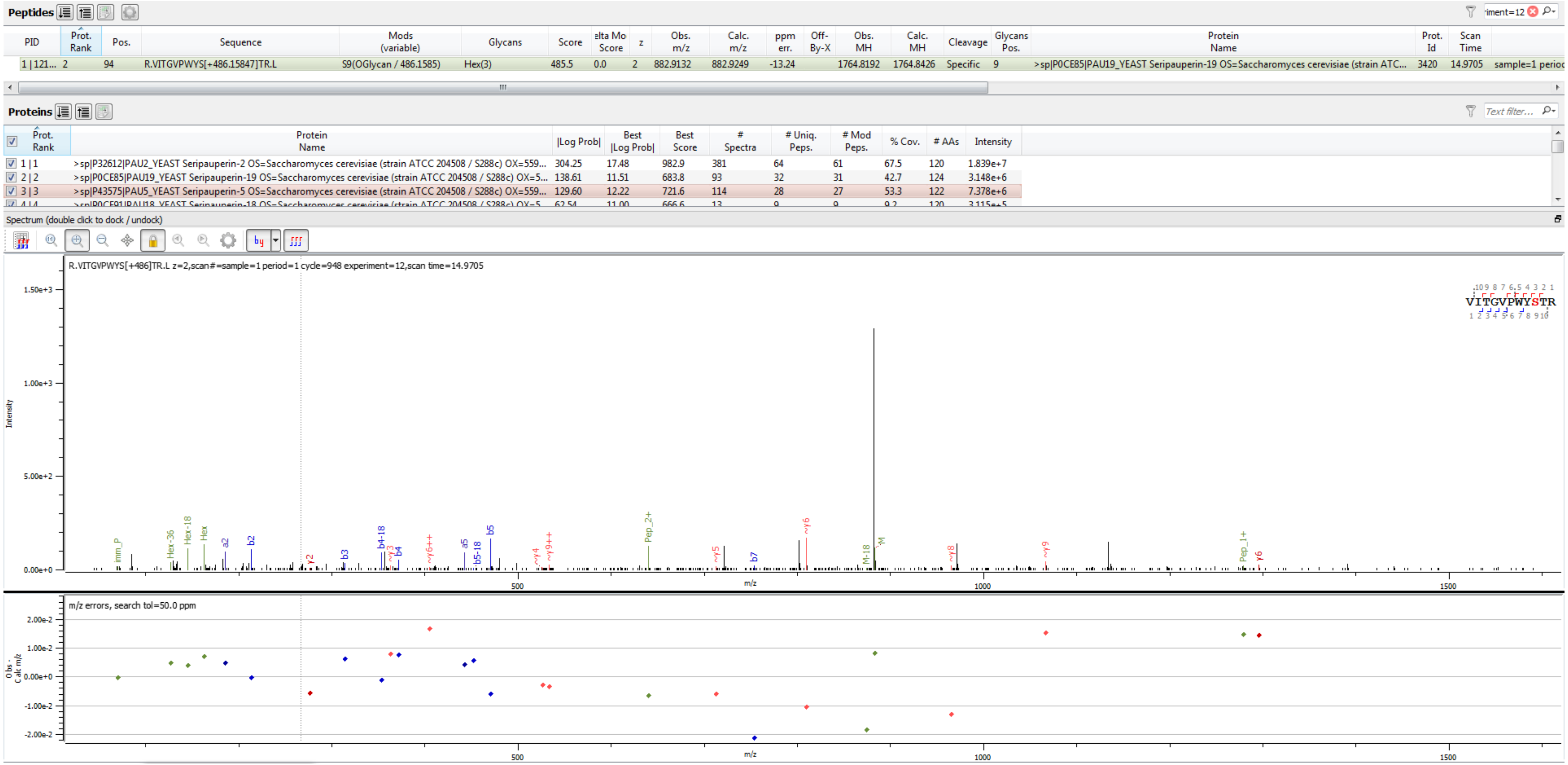

Peptides

PID	Prot. Rank	Pos.	Sequence	Mods (variable)	Glycans	Score	elta Mo Score	z	Obs. m/z	Calc. m/z	ppm err.	Off-By-X	Obs. MH	Calc. MH	Cleavage	Glycans Pos.	Protein Name	Prot. Id	Scan Time	
1 | 121...	2	94	R.VITGVPWYS[+486.15847]TR.L	S9(OGlycan / 486.1585)	Hex(3)	485.5	0.0	2	882.9132	882.9249	-13.24		1764.8192	1764.8426	Specific	9	>sp|P0CE85|PAU19_YEAST Seripauperin-19 OS=Saccharomyces cerevisiae (strain ATC...	3420	14.9705	sample=1 perioc

Proteins

Prot. Rank	Protein Name	|Log Prob|	Best |Log Prob|	Best Score	# Spectra	# Uniq. Peps.	# Mod Peps.	% Cov.	# AAs	Intensity
1 | 1	>sp|P32612|PAU2_YEAST Seripauperin-2 OS=Saccharomyces cerevisiae (strain ATCC 204508 / S288c) OX=559...	304.25	17.48	982.9	381	64	61	67.5	120	1.839e+7
2 | 2	>sp|P0CE85|PAU19_YEAST Seripauperin-19 OS=Saccharomyces cerevisiae (strain ATCC 204508 / S288c) OX=5...	138.61	11.51	683.8	93	32	31	42.7	124	3.148e+6
3 | 3	>sp|P43575|PAU5_YEAST Seripauperin-5 OS=Saccharomyces cerevisiae (strain ATCC 204508 / S288c) OX=559...	129.60	12.22	721.6	114	28	27	53.3	122	7.378e+6
4 | 4	>sp|P0CE91|PAU18_YEAST Seripauperin-18 OS=Saccharomyces cerevisiae (strain ATCC 204508 / S288c) OX=5...	62.54	11.00	666.6	13	9	9	9.2	120	3.115e+5

Sample S4 R.VITGVPWYS[+486.158]TR.L

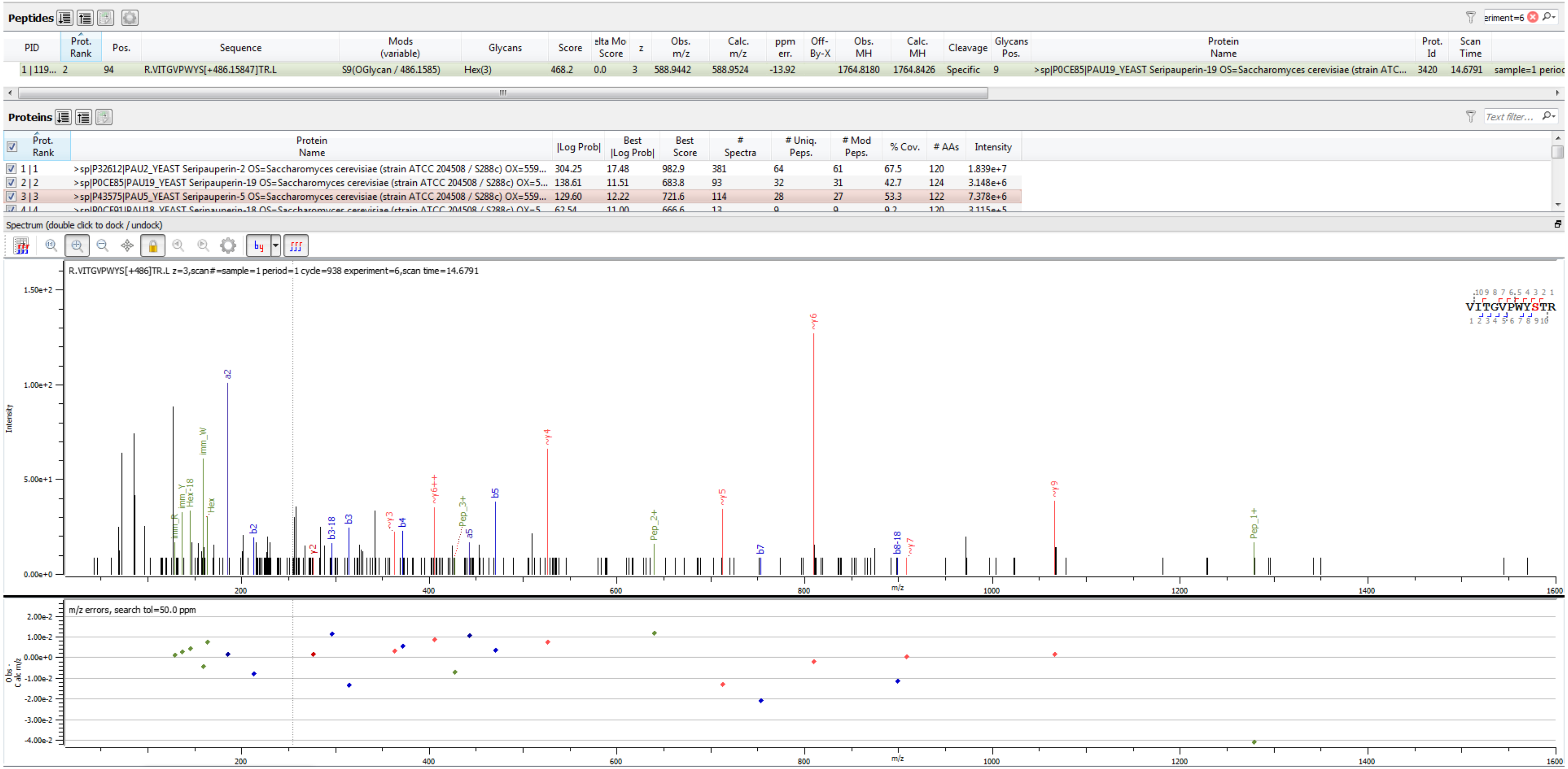

Sample S4 R.VITGVPWYS[+486.158]TR.L

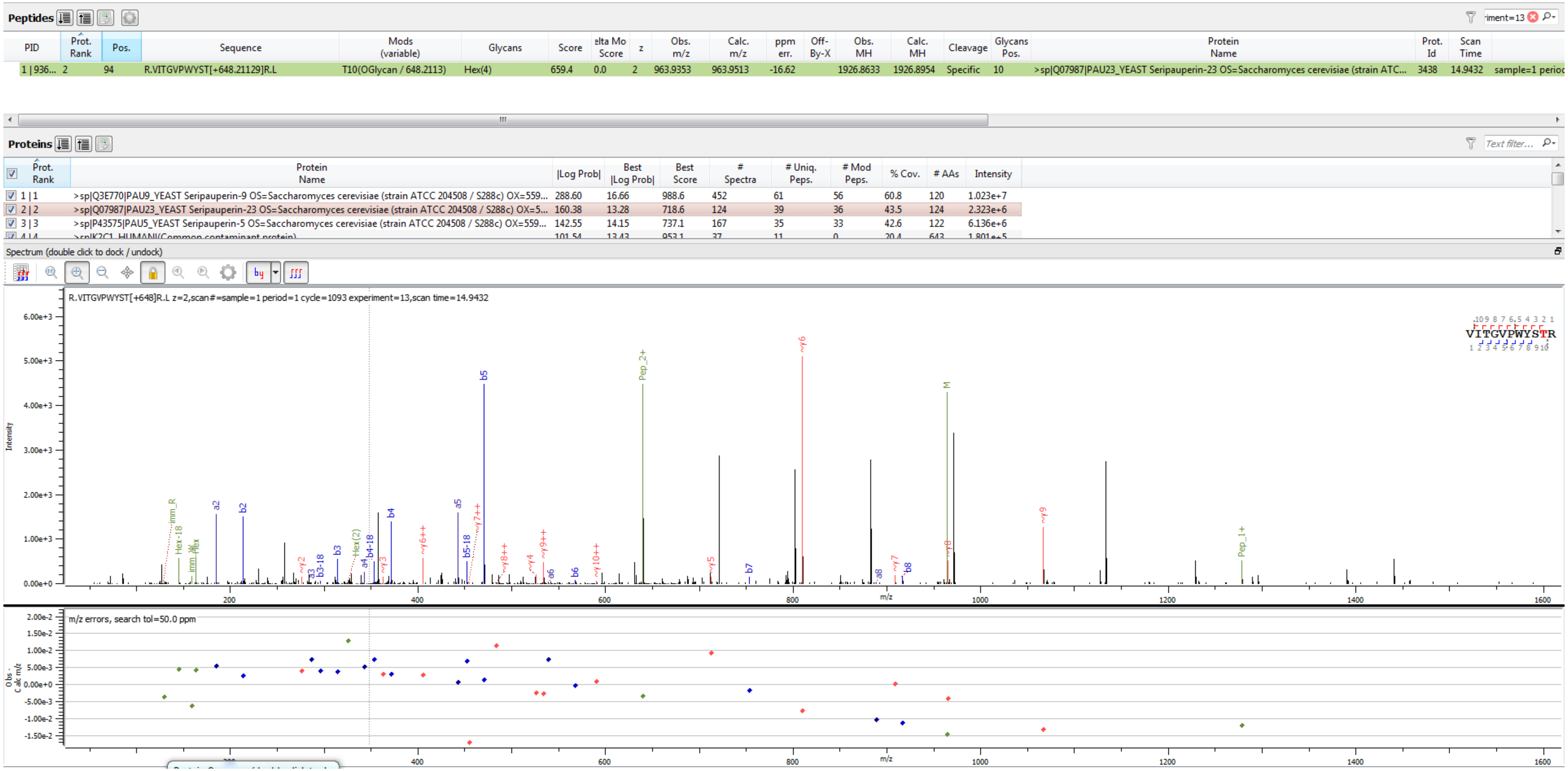

Sample S6 R.VITGVPWYST[+648.211]R.L

Peptides

PID	Prot. Rank	Pos.	Sequence	Mods (variable)	Glycans	Score	elta Mo Score	z	Obs. m/z	Calc. m/z	ppm err.	Off-By-X	Obs. MH	Calc. MH	Cleavage	Glycans Pos.	Protein Name	Prot. Id	Scan Time	
1 | 115...	3	94	R.VITGVPWYST[+648.21129]R.L	T10(OGlycan / 648.2113)	Hex(4)	594.7	0.0	3	642.9572	642.9700	-19.83		1926.8572	1926.8954	Specific	10	>sp|P0CE85|PAU19_YEAST Seripauperin-19 OS=Saccharomyces cerevisiae (strain ATC...	3420	14.4496	sample=1 perioc

Proteins

Prot. Rank	Protein Name	|Log Prob|	Best |Log Prob|	Best Score	# Spectra	# Uniq. Peps.	# Mod Peps.	% Cov.	# AAs	Intensity
1 | 1	>sp|Q3E770|PAU9_YEAST Seripauperin-9 OS=Saccharomyces cerevisiae (strain ATCC 204508 / S288c) OX=559...	248.32	15.68	955.8	342	58	55	67.5	120	1.797e+7

Spectrum (double click to dock / undock)

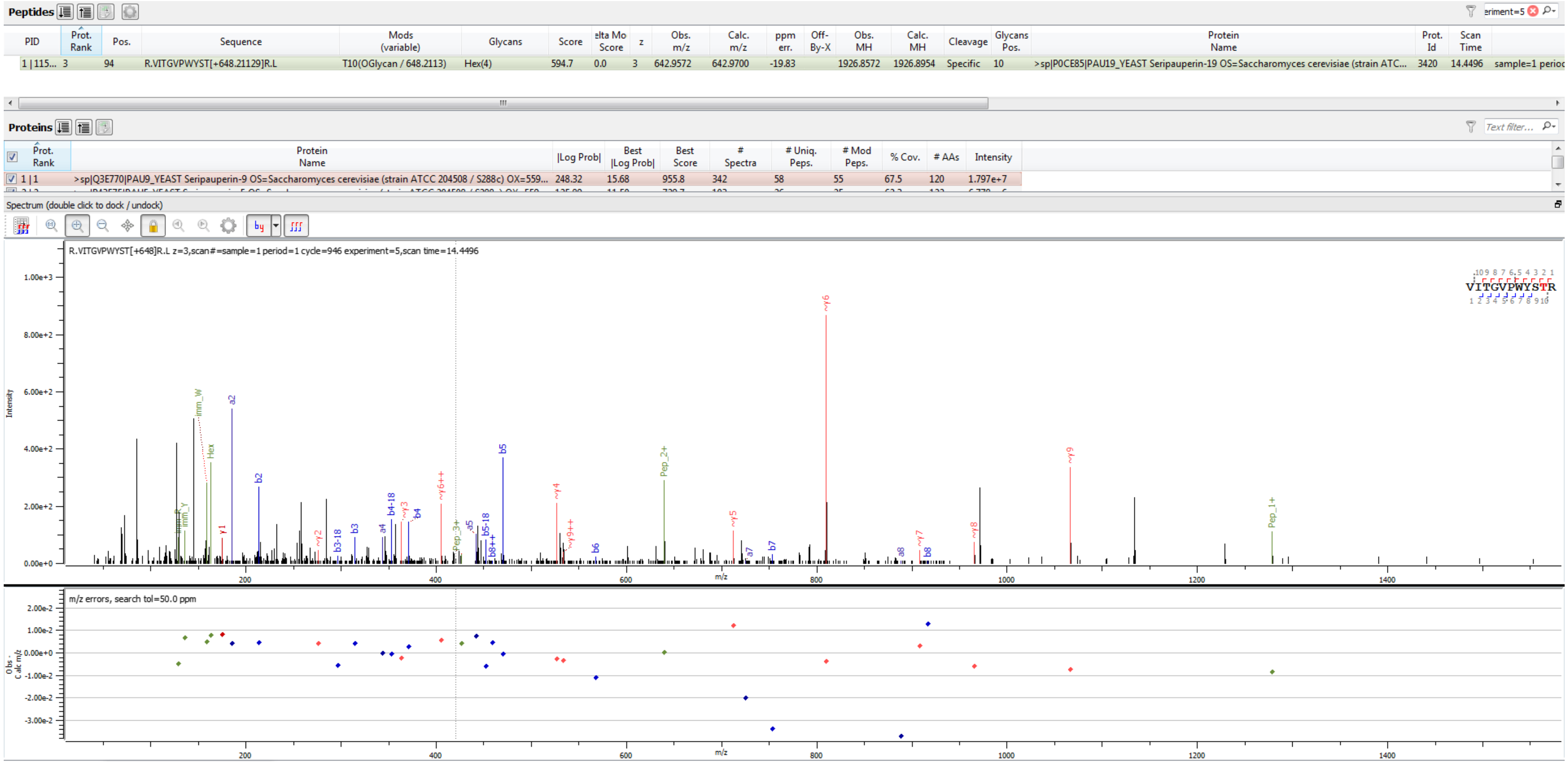

Sample S3 R.VITGVPWYST[+648.211]R.L

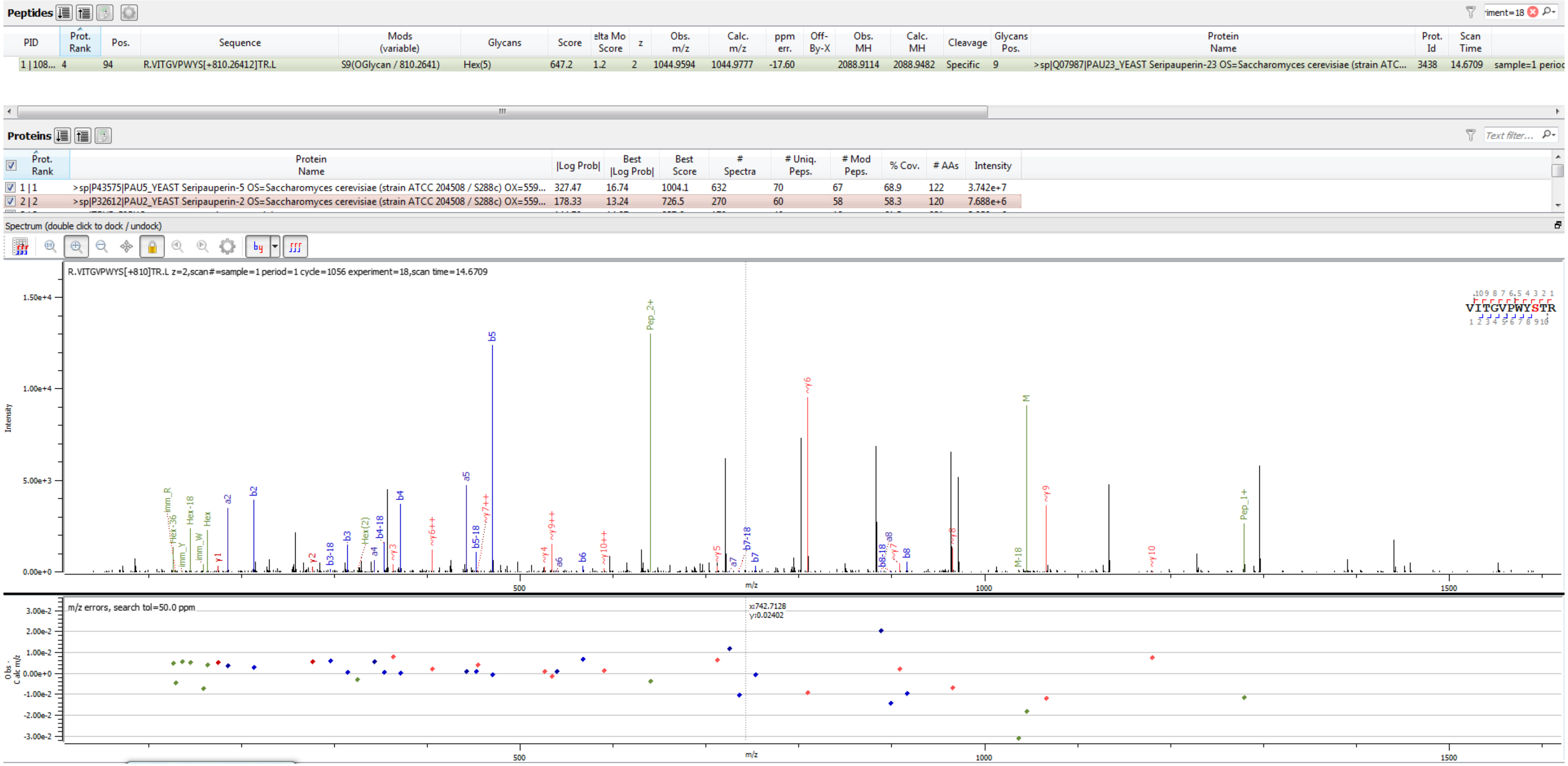

Sample S1 R.VITGVPWYS[+810.264]TR.L

Peptides

PID	Prot. Rank	Pos.	Sequence	Mods (variable)	Glycans	Score	elta Mo Score	z	Obs. m/z	Calc. m/z	ppm err.	Off-By-X	Obs. MH	Calc. MH	Cleavage	Glycans Pos.	Protein Name	Prot. Id	Scan Time	
1 | 927...	2	94	R.VITGVPWYS[+810.26412]TR.L	S9(OGlycan / 810.2641)	Hex(5)	711.5	1.2	3	696.9746	696.9876	-18.69		2088.9092	2088.9482	Specific	9	>sp|Q07987|PAU23_YEAST Seripauperin-23 OS=Saccharomyces cerevisiae (strain ATC...	3438	14.8259	sample=1 perioc

Proteins

Prot. Rank	Protein Name	|Log Prob|	Best |Log Prob|	Best Score	# Spectra	# Uniq. Peps.	# Mod Peps.	% Cov.	# AAs	Intensity
1 | 1	>sp|Q3E770|PAU9_YEAST Seripauperin-9 OS=Saccharomyces cerevisiae (strain ATCC 204508 / S288c) OX=559...	288.60	16.66	988.6	452	61	56	60.8	120	1.023e+7
2 | 2	>sp|Q07987|PAU23_YEAST Seripauperin-23 OS=Saccharomyces cerevisiae (strain ATCC 204508 / S288c) OX=5...	160.38	13.28	718.6	124	39	36	43.5	124	2.323e+6
3 | 3	>sp|P43575|PAU5_YEAST Seripauperin-5 OS=Saccharomyces cerevisiae (strain ATCC 204508 / S288c) OX=559...	142.55	14.15	737.1	167	35	33	42.6	122	6.136e+6
4 | 4	>sp|K2C1_HUMAN|(Common contaminant protein)	101.54	13.43	953.1	37	11	0	20.4	643	1.801e+5

Spectrum (double click to dock / undock)

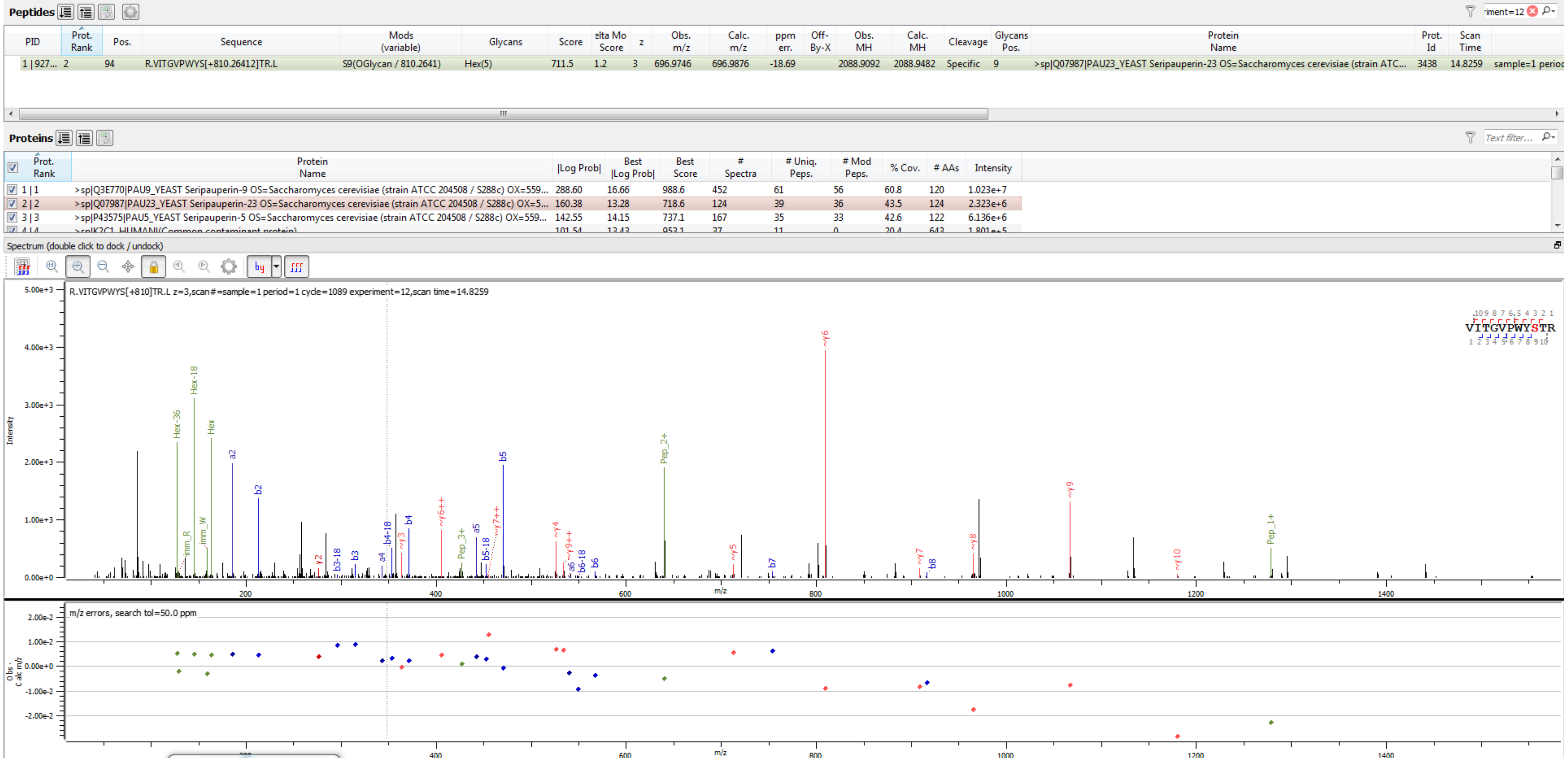

Sample S6 R.VITGVPWYS[+810.264]TR.L

Peptides

PID	Prot. Rank	Pos.	Sequence	Mods (variable)	Glycans	Score	elta Mo Score	z	Obs. m/z	Calc. m/z	ppm err.	Off-By-X	Obs. MH	Calc. MH	Cleavage	Glycans Pos.	Protein Name	Prot. Id	Scan Time	
1 | 187...	2	94	R.VITGVPWYS[+972.31694]TR.L	S9(OGlycan / 972.3169)	Hex(6)	622.4	1.2	2	1125.9857	1126.0041	-16.40		2250.9641	2251.0010	Specific	9	>sp|P0CE85|PAU19_YEAST Seripauperin-19 OS=Saccharomyces cerevisiae (strain ATC...	3420	14.3431	sample=1 perioc

Proteins

Prot. Rank	Protein Name	|Log Prob|	Best |Log Prob|	Best Score	# Spectra	# Uniq. Peps.	# Mod Peps.	% Cov.	# AAs	Intensity
1 | 1	>sp|P32612|PAU2_YEAST Seripauperin-2 OS=Saccharomyces cerevisiae (strain ATCC 204508 / S288c) OX=559...	304.25	17.48	982.9	381	64	61	67.5	120	1.839e+7
2 | 2	>sp|P0CE85|PAU19_YEAST Seripauperin-19 OS=Saccharomyces cerevisiae (strain ATCC 204508 / S288c) OX=5...	138.61	11.51	683.8	93	32	31	42.7	124	3.148e+6
3 | 3	>sp|P43575|PAU5_YEAST Seripauperin-5 OS=Saccharomyces cerevisiae (strain ATCC 204508 / S288c) OX=559...	129.60	12.22	721.6	114	28	27	53.3	122	7.378e+6
4 | 4	>sp|P0CE91|PAU18_YEAST Seripauperin-18 OS=Saccharomyces cerevisiae (strain ATCC 204508 / S288c) OX=5...	62.54	11.00	666.6	13	9	9	9.2	120	3.115e+5

Spectrum (double click to dock / undock)

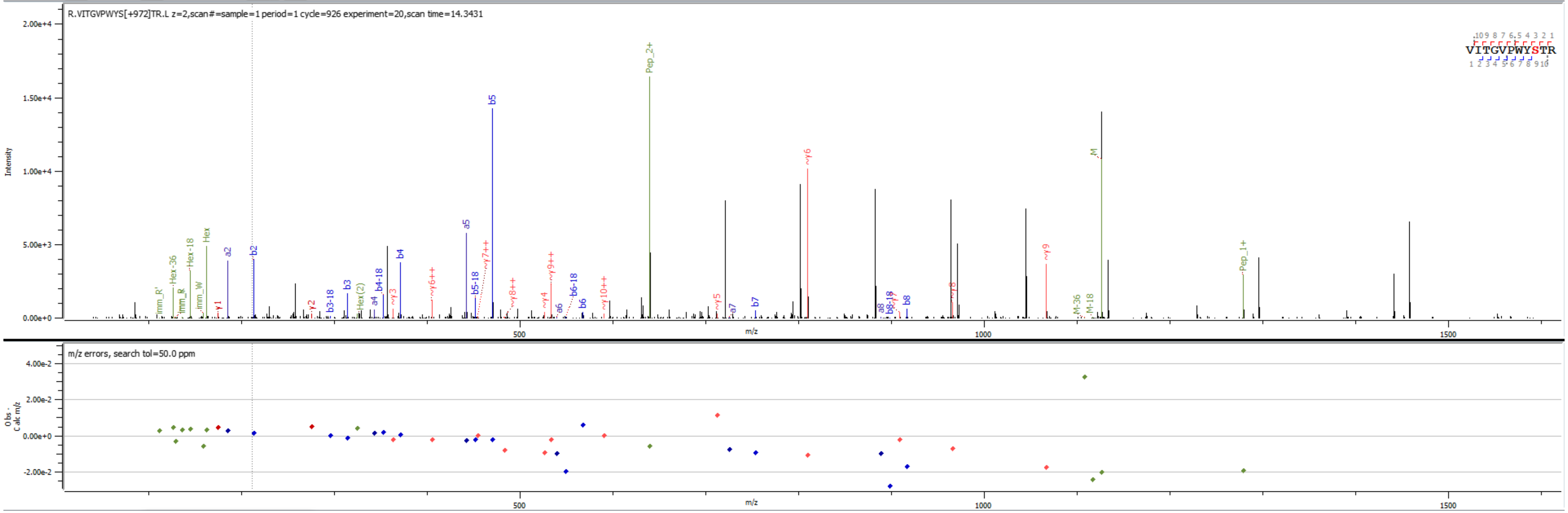

Sample S4 R.VITGVPWYS[+972.317]TR.L

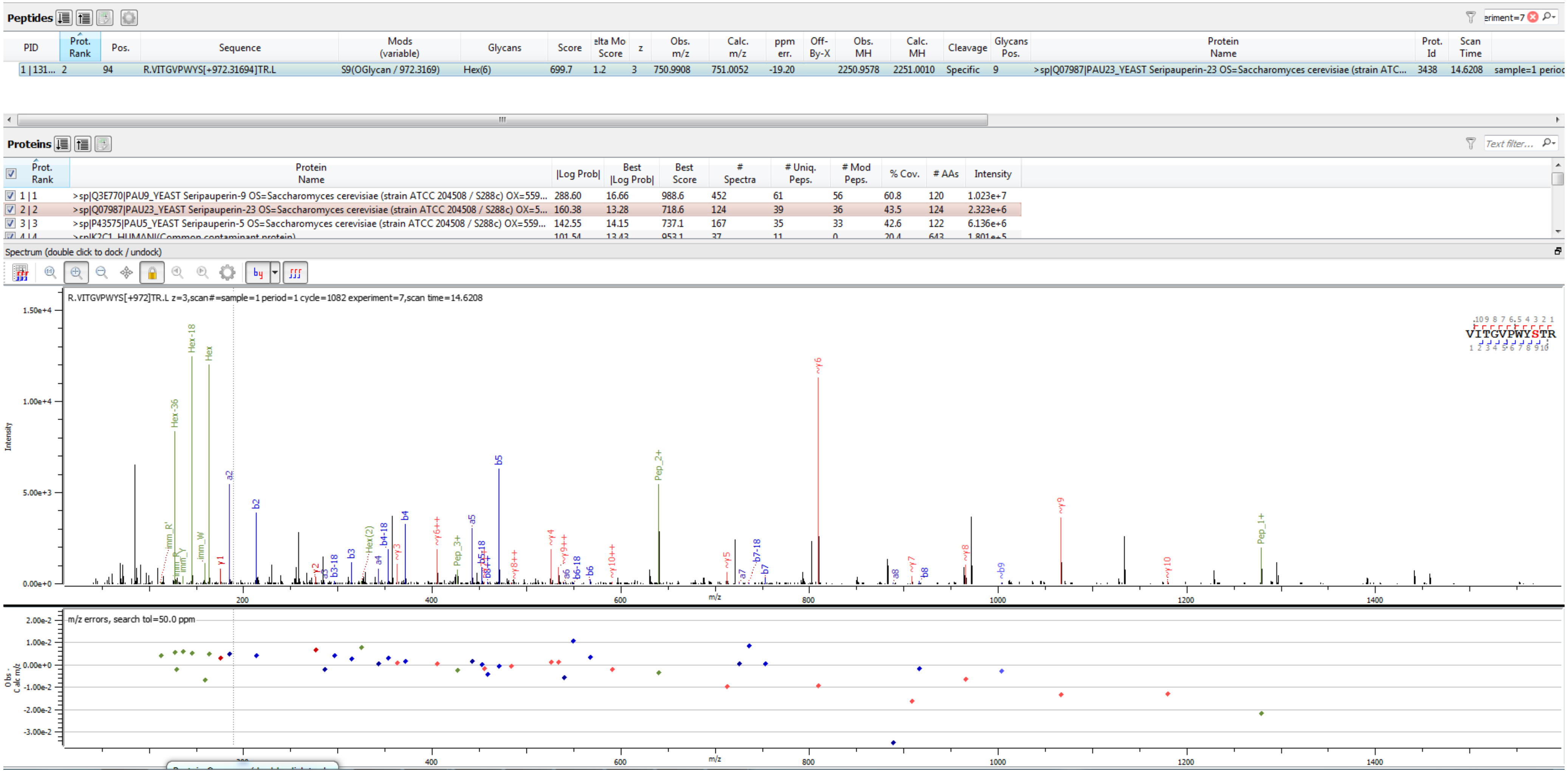

Sample S6 R.VITGVPWYS[+972.317]TR.L

Peptides

PID	Prot. Rank	Pos.	Sequence	Mods (variable)	Glycans	Score	elta Mo Score	z	Obs. m/z	Calc. m/z	ppm err.	Off-By-X	Obs. MH	Calc. MH	Cleavage	Glycans Pos.	Protein Name	Prot. Id	Scan Time	
1 | 186...	2	94	R.VITGVPWYS[+1134.36976]TR.L	S9(OGlycan / 1134.3698)	Hex(7)	604.1	1.2	2	1207.0075	1207.0306	-19.11		2413.0077	2413.0538	Specific	9	>sp|P0CE85|PAU19_YEAST Seripauperin-19 OS=Saccharomyces cerevisiae (strain ATC...	3420	14.2868	sample=1 perioc

Proteins

Prot. Rank	Protein Name	|Log Prob|	Best |Log Prob|	Best Score	# Spectra	# Uniq. Peps.	# Mod Peps.	% Cov.	# AAs	Intensity
1 | 1	>sp|P32612|PAU2_YEAST Seripauperin-2 OS=Saccharomyces cerevisiae (strain ATCC 204508 / S288c) OX=559...	304.25	17.48	982.9	381	64	61	67.5	120	1.839e+7
2 | 2	>sp|P0CE85|PAU19_YEAST Seripauperin-19 OS=Saccharomyces cerevisiae (strain ATCC 204508 / S288c) OX=5...	138.61	11.51	683.8	93	32	31	42.7	124	3.148e+6
3 | 3	>sp|P43575|PAU5_YEAST Seripauperin-5 OS=Saccharomyces cerevisiae (strain ATCC 204508 / S288c) OX=559...	129.60	12.22	721.6	114	28	27	53.3	122	7.378e+6
4 | 4	>sp|P0CE91|PAU18_YEAST Seripauperin-18 OS=Saccharomyces cerevisiae (strain ATCC 204508 / S288c) OX=5...	62.54	11.00	666.6	13	9	9	9.2	120	3.115e+5

Spectrum (double click to dock / undock)

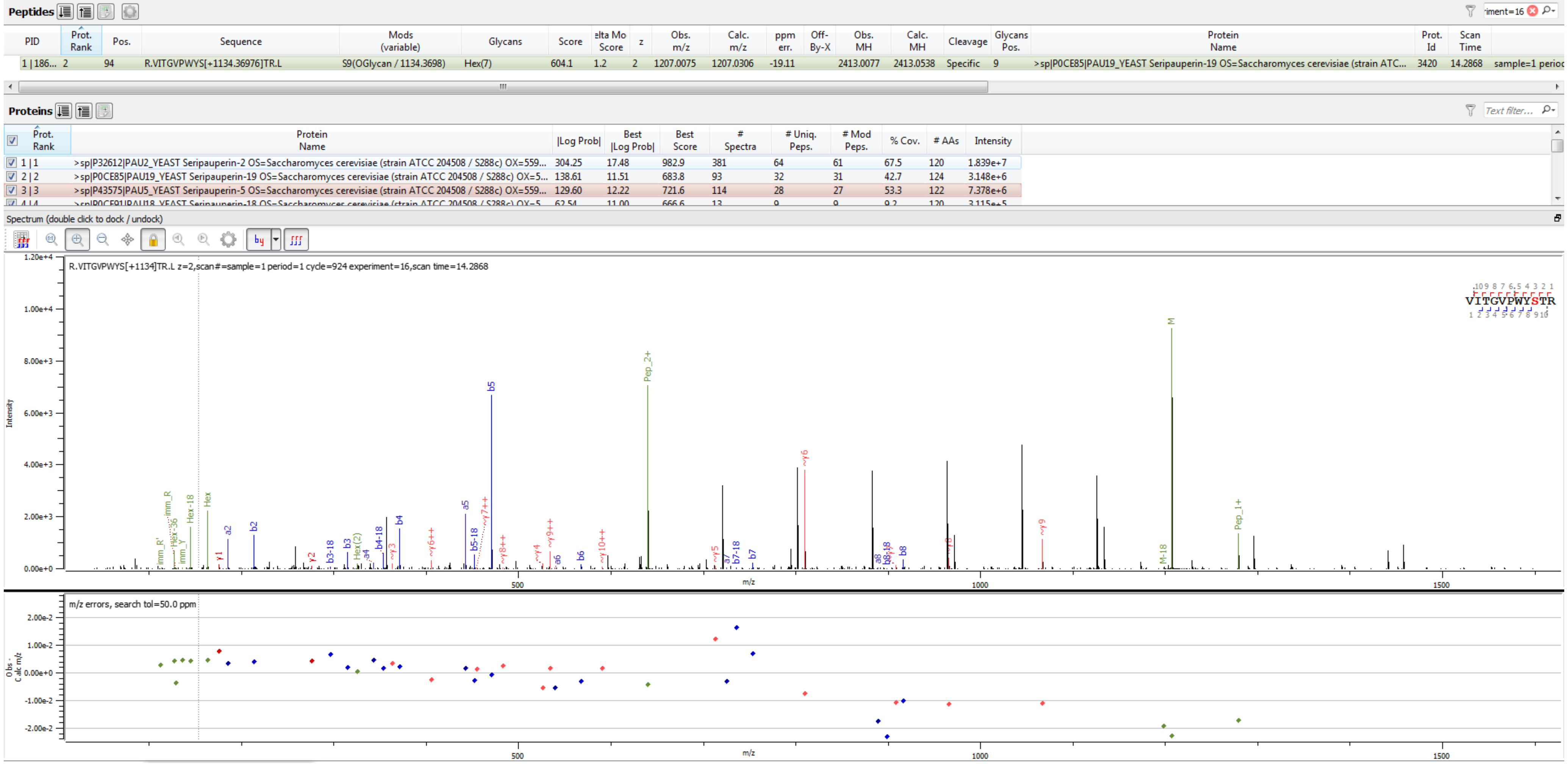

Sample S4 R.VITGVPWYS[+1134.370]TR.L

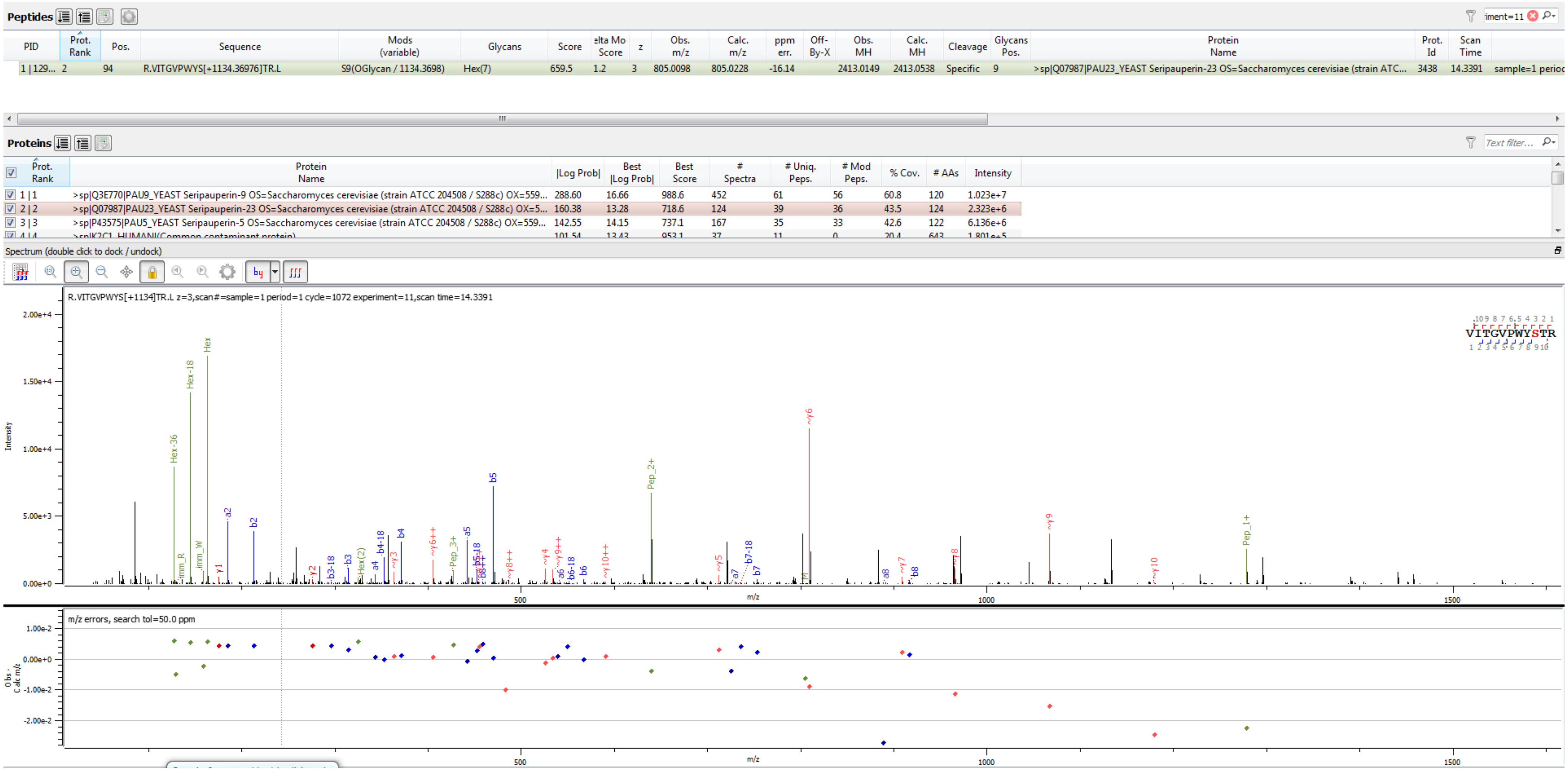

Sample S6 R.VITGVPWYS[+1134.370]TR.L

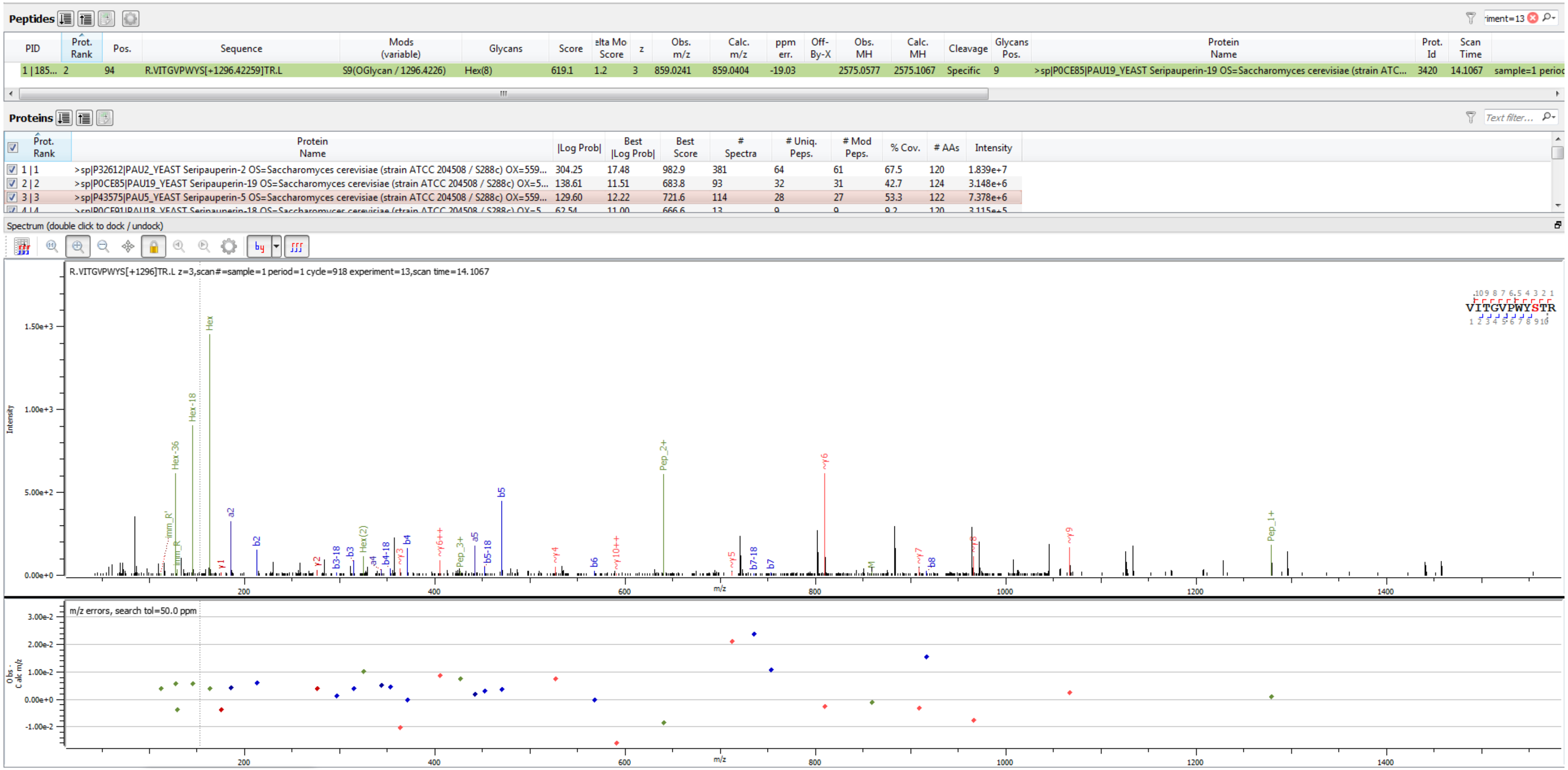

Sample S4 R.VITGVPWYS[+1296.423]TR.L

Peptides

PID	Prot. Rank	Pos.	Sequence	Mods (variable)	Glycans	Score	elta Mo Score	z	Obs. m/z	Calc. m/z	ppm err.	Off-By-X	Obs. MH	Calc. MH	Cleavage	Glycans Pos.	Protein Name	Prot. Id	Scan Time	
1 | 185...	2	94	R.VITGVPWYS[+1458.47541]TR.L	S9(OGlycan / 1458.4754)	Hex(9)	455.1	1.2	3	913.0484	913.0580	-10.59		2737.1305	2737.1595	Specific	9	>sp|P0CE85|PAU19_YEAST Seripauperin-19 OS=Saccharomyces cerevisiae (strain ATC...	3420	14.1388	sample=1 period

Proteins

Prot. Rank	Protein Name	|Log Prob|	Best |Log Prob|	Best Score	# Spectra	# Uniq. Peps.	# Mod Peps.	% Cov.	# AAs	Intensity
1 | 1	>sp|P32612|PAU2_YEAST Seripauperin-2 OS=Saccharomyces cerevisiae (strain ATCC 204508 / S288c) OX=559...	304.25	17.48	982.9	381	64	61	67.5	120	1.839e+7
2 | 2	>sp|P0CE85|PAU19_YEAST Seripauperin-19 OS=Saccharomyces cerevisiae (strain ATCC 204508 / S288c) OX=5...	138.61	11.51	683.8	93	32	31	42.7	124	3.148e+6
3 | 3	>sp|P43575|PAU5_YEAST Seripauperin-5 OS=Saccharomyces cerevisiae (strain ATCC 204508 / S288c) OX=559...	129.60	12.22	721.6	114	28	27	53.3	122	7.378e+6
4 | 4	>sp|P0CE91|PAU18_YEAST Seripauperin-18 OS=Saccharomyces cerevisiae (strain ATCC 204508 / S288c) OX=5...	62.54	11.00	666.6	13	9	9	9.2	120	3.115e+5

Spectrum (double click to dock / undock)

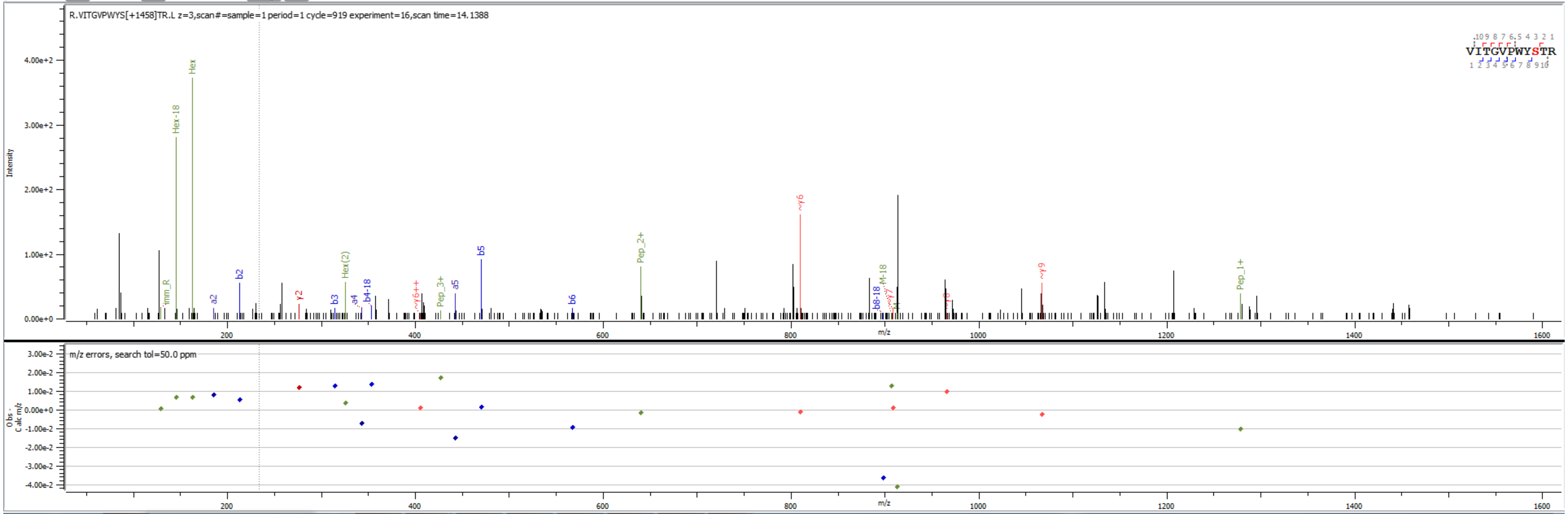

Sample S4 R.VITGVPWYS[+1458.475]TR.L

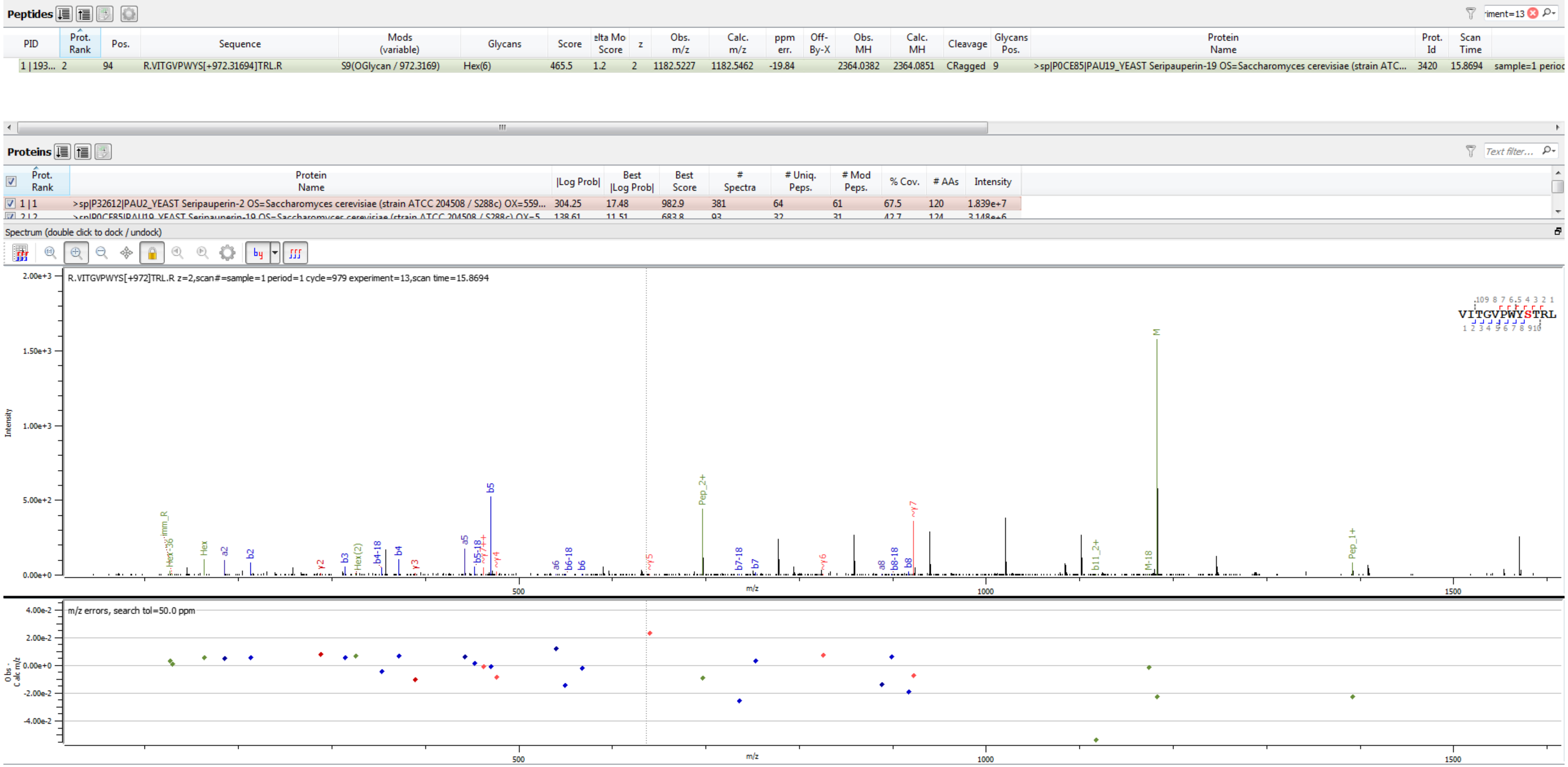

Sample S   R.VITGVPWYS[+972.317]TRL.R

Peptides

PID	Prot. Rank	Pos.	Sequence	Mods (variable)	Glycans	Score	elta Mo Score	z	Obs. m/z	Calc. m/z	ppm err.	Off-By-X	Obs. MH	Calc. MH	Cleavage	Glycans Pos.	Protein Name	Prot. Id	Scan Time	
1 | 203...	3	94	R.VITGVPWYST[+972.31694]RL.R	T10(OGlycan / 972.3169)	Hex(6)	586.9	0.8	3	788.6845	788.6999	-19.53		2364.0389	2364.0851	CRagged	10	>sp|P0CE85|PAU19_YEAST Seripauperin-19 OS=Saccharomyces cerevisiae (strain ATC...	3420	15.8658	sample=1 perioc

Proteins

Prot. Rank	Protein Name	|Log Prob|	Best |Log Prob|	Best Score	# Spectra	# Uniq. Peps.	# Mod Peps.	% Cov.	# AAs	Intensity
3 | 3	>sp|P0CE85|PAU19_YEAST Seripauperin-19 OS=Saccharomyces cerevisiae (strain ATCC 204508 / S288c) OX=5...	123.82	11.06	692.3	77	31	30	42.7	124	2.619e+6
4 | 4	>sp|K2C1_HUMAN|(Common contaminant protein)	75.52	13.70	921.5	17	8	0	13.8	643	1.827e+5

Spectrum (double click to dock / undock)

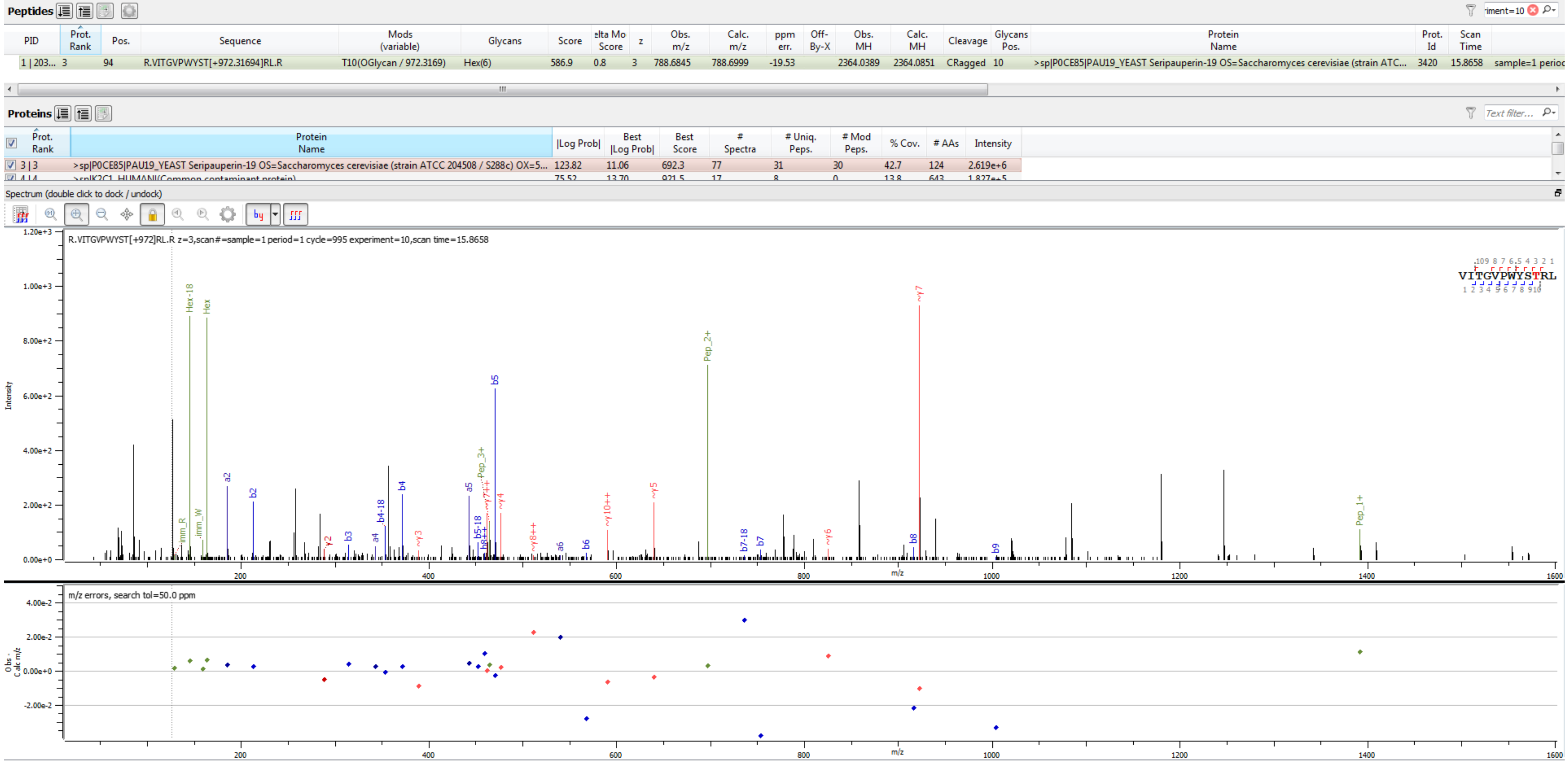

Sample S3 R.VITGVPWYST[+972.317]RL.R

Peptides

PID	Prot. Rank	Pos.	Sequence	Mods (variable)	Glycans	Score	elta Mo Score	z	Obs. m/z	Calc. m/z	ppm err.	Off-By-X	Obs. MH	Calc. MH	Cleavage	Glycans Pos.	Protein Name	Prot. Id	Scan Time	
1 | 104...	4	94	R.VITGVPWYS[+1134.36976]TRL.R	S9(OGlycan / 1134.3698)	Hex(7)	574.4	1.2	3	842.7011	842.7175	-19.51		2526.0886	2526.1379	CRagged	9	>sp|Q07987|PAU23_YEAST Seripauperin-23 OS=Saccharomyces cerevisiae (strain ATC...	3438	15.8940	sample=1 perioc

Proteins

Prot. Rank	Protein Name	|Log Prob|	Best |Log Prob|	Best Score	# Spectra	# Uniq. Peps.	# Mod Peps.	% Cov.	# AAs	Intensity
1 | 1	>sp|P43575|PAU5_YEAST Seripauperin-5 OS=Saccharomyces cerevisiae (strain ATCC 204508 / S288c) OX=559...	136.57	14.25	968.4	163	30	27	28.7	122	1.440e+6

Spectrum (double click to dock / undock)

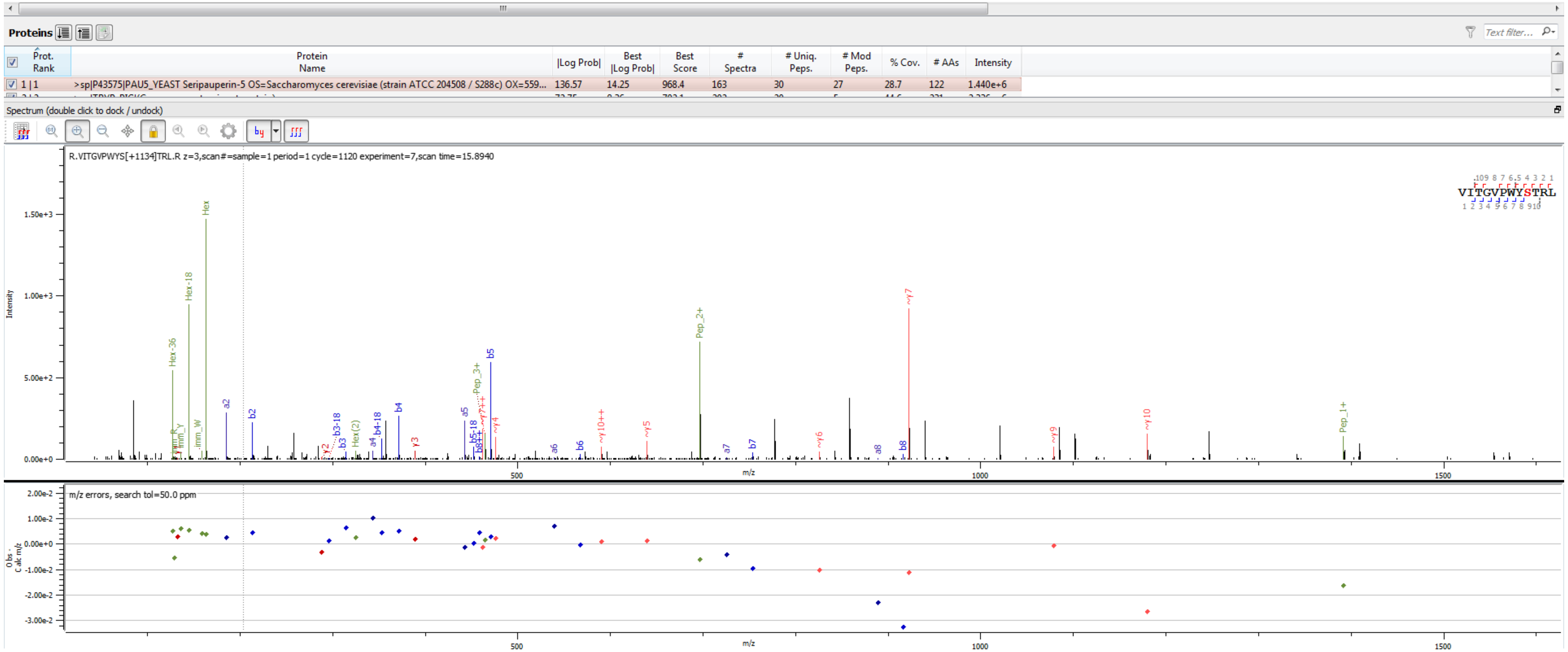

Sample S5 R.VITGVPWYS[+1134.370]TRL.R

Peptides

PID	Prot. Rank	Pos.	Sequence	Mods (variable)	Glycans	Score	elta Mo Score	z	Obs. m/z	Calc. m/z	ppm err.	Off-By-X	Obs. MH	Calc. MH	Cleavage	Glycans Pos.	Protein Name	Prot. Id	Scan Time	
1 | 170...	5	94	R.VITGVPWYS[+1296.42259]TRL.R	S9(OGlycan / 1296.4226)	Hex(8)	536.3	1.2	3	896.7207	896.7351	-16.04		2688.1476	2688.1907	CRagged	9	>sp|P0CE85|PAU19_YEAST Seripauperin-19 OS=Saccharomyces cerevisiae (strain ATC...	3420	15.7642	sample=1 perioc

Proteins

Prot. Rank	Protein Name	|Log Prob|	Best |Log Prob|	Best Score	# Spectra	# Uniq. Peps.	# Mod Peps.	% Cov.	# AAs	Intensity
3 | 3	>sp|P43575|PAU5_YEAST Seripauperin-5 OS=Saccharomyces cerevisiae (strain ATCC 204508 / S288c) OX=559...	117.08	14.43	972.1	214	40	39	34.4	122	3.360e+6

Spectrum (double click to dock / undock)

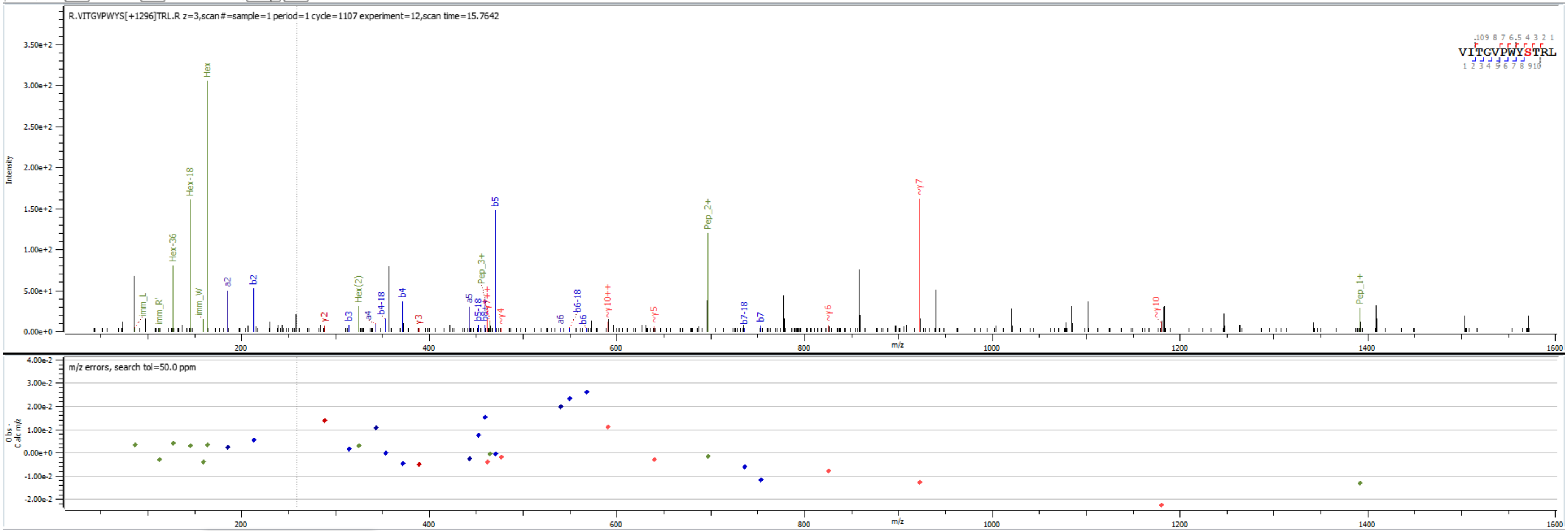

Sample S2 R.VITGVPWYS[+1296.423]TRL.R

Peptides

PID	Prot. Rank	Pos.	Sequence	Mods (variable)	Glycans	Score	elta Mo Score	z	Obs. m/z	Calc. m/z	ppm err.	Off-By-X	Obs. MH	Calc. MH	Cleavage	Glycans Pos.	Protein Name	Prot. Id	Scan Time	
1 | 125...	4	91	R.MITGVPWYS[+486.15847]TR.L	S9(OGlycan / 486.1585)	Hex(3)	549.4	0.0	2	898.8985	898.9109	-13.91		1796.7896	1796.8146	Specific	9	>sp|P0CE91|PAU18_YEAST Seripauperin-18 OS=Saccharomyces cerevisiae (strain ATC...	3409	15.4065	sample=1 perioc

Proteins

Prot. Rank	Protein Name	|Log Prob|	Best |Log Prob|	Best Score	# Spectra	# Uniq. Peps.	# Mod Peps.	% Cov.	# AAs	Intensity
1 | 1	>sp|P32612|PAU2_YEAST Seripauperin-2 OS=Saccharomyces cerevisiae (strain ATCC 204508 / S288c) OX=559...	304.25	17.48	982.9	381	64	61	67.5	120	1.839e+7
2 | 2	>sp|P0CE85|PAU19_YEAST Seripauperin-19 OS=Saccharomyces cerevisiae (strain ATCC 204508 / S288c) OX=5...	138.61	11.51	683.8	93	32	31	42.7	124	3.148e+6
3 | 3	>sp|P43575|PAU5_YEAST Seripauperin-5 OS=Saccharomyces cerevisiae (strain ATCC 204508 / S288c) OX=559...	129.60	12.22	721.6	114	28	27	53.3	122	7.378e+6
4 | 4	>sp|P0CE91|PAU18_YEAST Seripauperin-18 OS=Saccharomyces cerevisiae (strain ATCC 204508 / S288c) OX=5...	62.54	11.00	666.6	13	9	9	9.2	120	3.115e+5

Spectrum (double click to dock / undock)

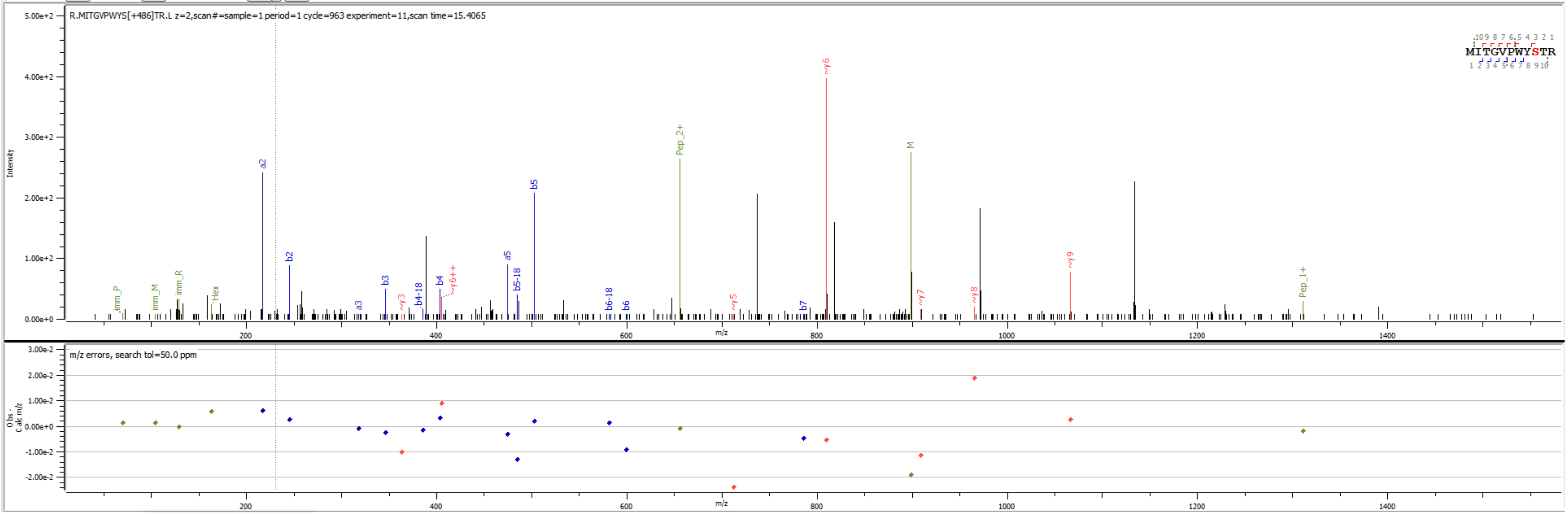

Sample S4 R.MITGVPWYS[+486.158]TR.L

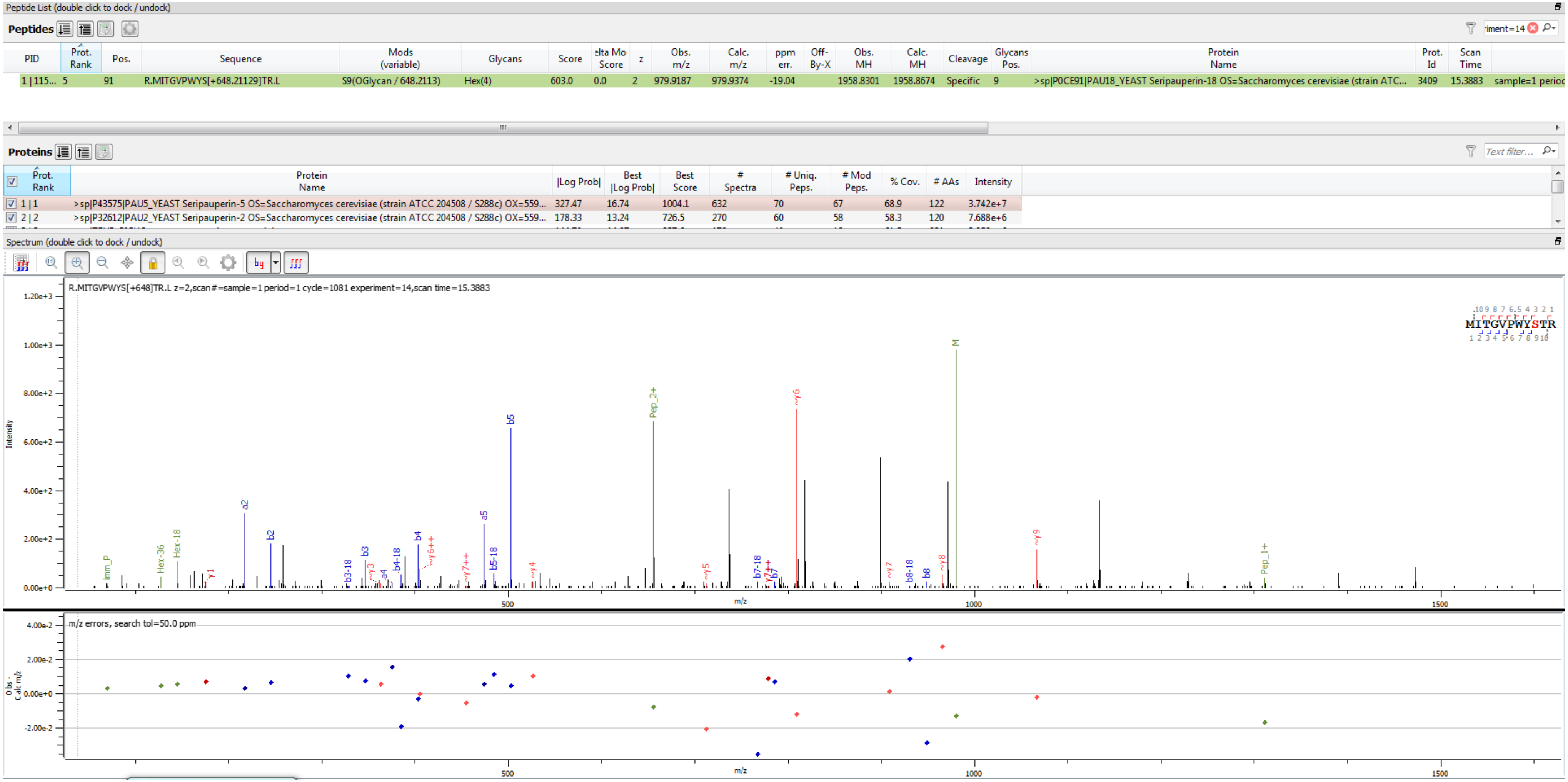

Sample S1 R.MITGVPWYS[+648.211]TR.L

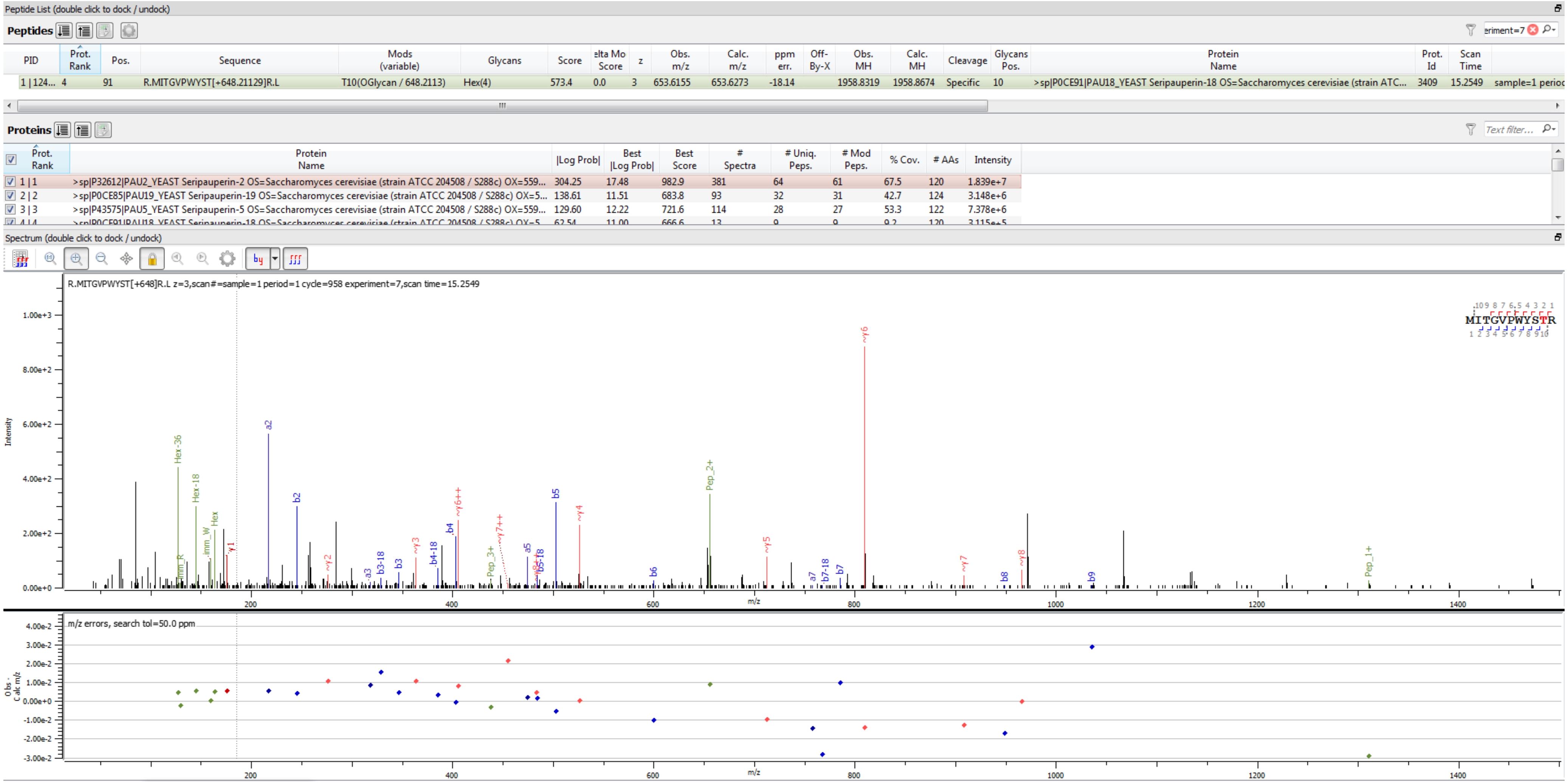

Sample S4 R.MITGVPWYS[+648.211]TR.L

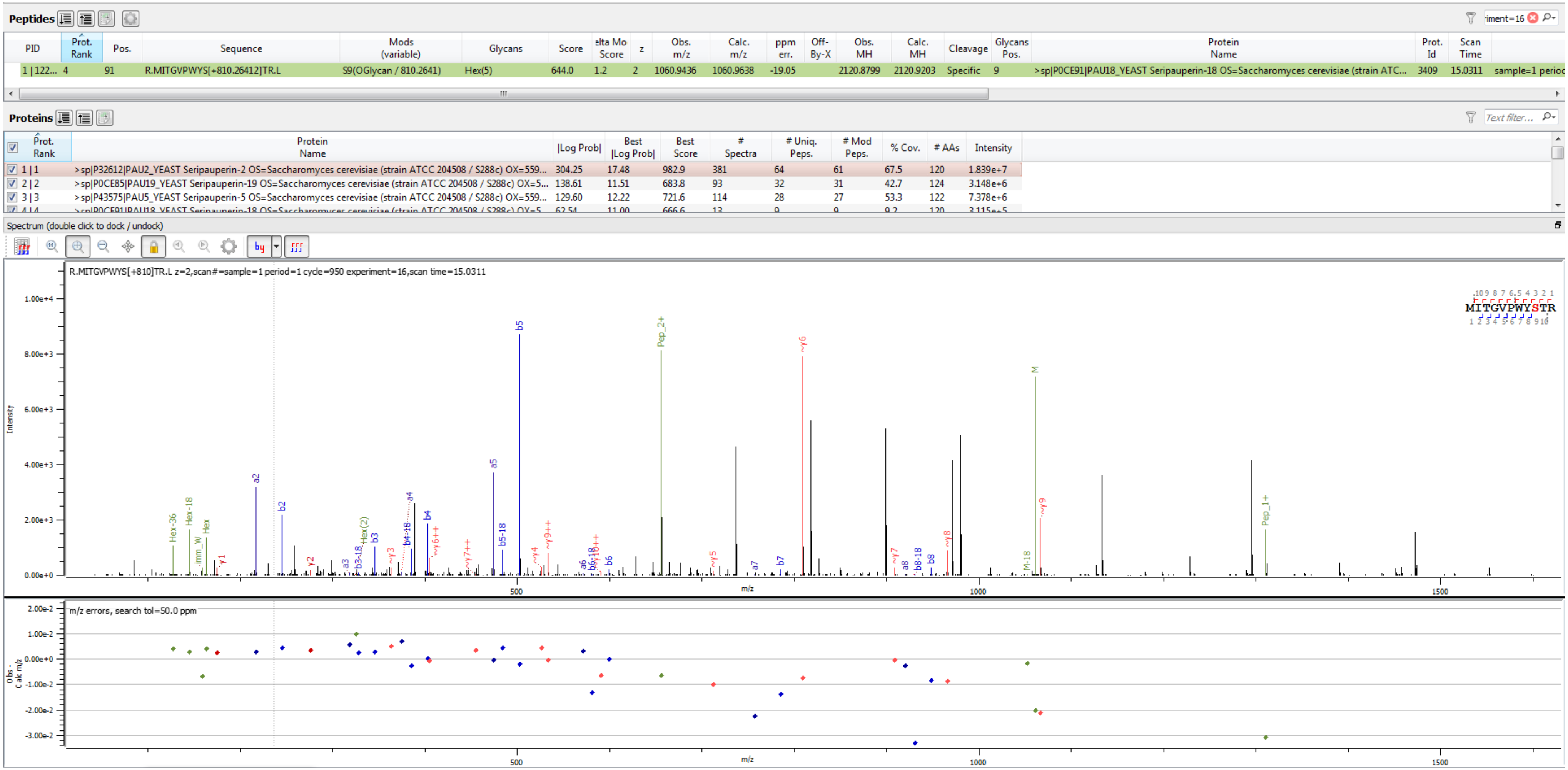

Peptides

PID	Prot. Rank	Pos.	Sequence	Mods (variable)	Glycans	Score	elta Mo Score	z	Obs. m/z	Calc. m/z	ppm err.	Off-By-X	Obs. MH	Calc. MH	Cleavage	Glycans Pos.	Protein Name	Prot. Id	Scan Time	
1 | 122...	4	91	R.MITGVPWYS[+810.26412]TR.L	S9(OGlycan / 810.2641)	Hex(5)	644.0	1.2	2	1060.9436	1060.9638	-19.05		2120.8799	2120.9203	Specific	9	>sp|P0CE91|PAU18_YEAST Seripauperin-18 OS=Saccharomyces cerevisiae (strain ATC...	3409	15.0311	sample=1 perioc

Proteins

Prot. Rank	Protein Name	|Log Prob|	Best |Log Prob|	Best Score	# Spectra	# Uniq. Peps.	# Mod Peps.	% Cov.	# AAs	Intensity
1 | 1	>sp|P32612|PAU2_YEAST Seripauperin-2 OS=Saccharomyces cerevisiae (strain ATCC 204508 / S288c) OX=559...	304.25	17.48	982.9	381	64	61	67.5	120	1.839e+7
2 | 2	>sp|P0CE85|PAU19_YEAST Seripauperin-19 OS=Saccharomyces cerevisiae (strain ATCC 204508 / S288c) OX=5...	138.61	11.51	683.8	93	32	31	42.7	124	3.148e+6
3 | 3	>sp|P43575|PAU5_YEAST Seripauperin-5 OS=Saccharomyces cerevisiae (strain ATCC 204508 / S288c) OX=559...	129.60	12.22	721.6	114	28	27	53.3	122	7.378e+6

Sample S4 R.MITGVPWYS[+810.264]TR.L

Peptides

PID	Prot. Rank	Pos.	Sequence	Mods (variable)	Glycans	Score	elta Mo Score	z	Obs. m/z	Calc. m/z	ppm err.	Off-By-X	Obs. MH	Calc. MH	Cleavage	Glycans Pos.	Protein Name	Prot. Id	Scan Time	
1 | 957...	6	91	R.MITGVPWYS[+810.26412]TR.L	S9(OGlycan / 810.2641)	Hex(5)	684.2	1.2	3	707.6310	707.6449	-19.79		2120.8783	2120.9203	Specific	9	>sp|P0CE91|PAU18_YEAST Seripauperin-18 OS=Saccharomyces cerevisiae (strain ATC...	3409	15.2348	sample=1 perio

Proteins

Prot. Rank	Protein Name	|Log Prob|	Best |Log Prob|	Best Score	# Spectra	# Uniq. Peps.	# Mod Peps.	% Cov.	# AAs	Intensity
10 | 10	>sp|P47178|DAN1_YEAST Cell wall protein DAN1 OS=Saccharomyces cerevisiae (strain ATCC 204508 / S288c) ...	22.21	11.15	731.5	10	6	6	11.7	298	8.248e+4
11 | 11	>sp|P53301|CRH1_YEAST Probable glycosidase CRH1 OS=Saccharomyces cerevisiae (strain ATCC 204508 / S2...	20.22	7.86	610.2	15	5	0	9.5	507	2.426e+4
12 | 12	>sp|P39005|KRE9_YEAST Cell wall synthesis protein KRE9 OS=Saccharomyces cerevisiae (strain ATCC 204508 /...	19.98	15.00	926.4	2	2	1	13.8	276	7.218e+3
13 | 13	>sp|P38616|YGP1_YEAST Protein YGP1 OS=Saccharomyces cerevisiae (strain ATCC 204508 / S288c) OX=55929	18.49	12.21	767.7	4	2	0	8.5	354	1.798e+4

Spectrum (double click to dock / undock)

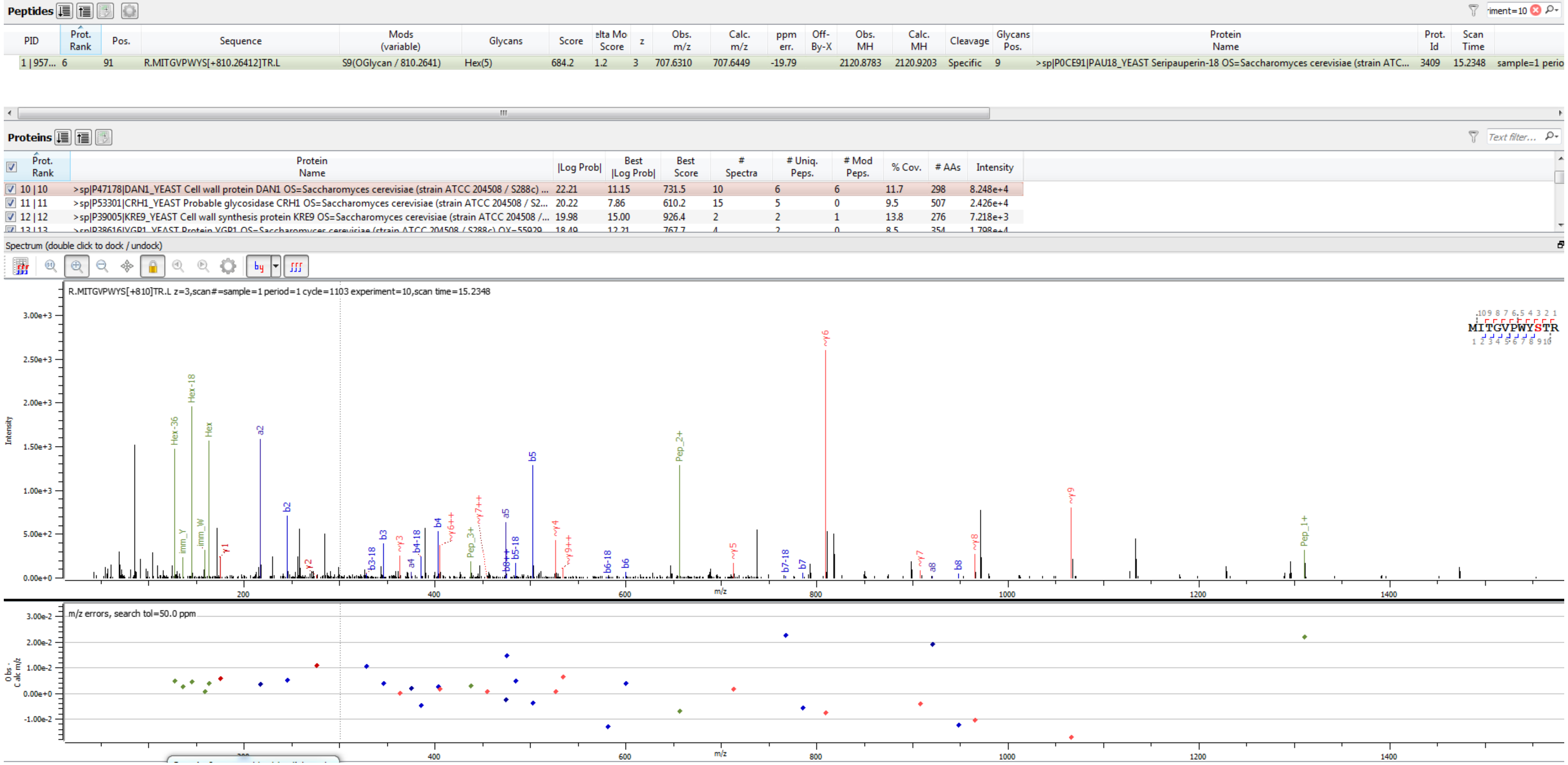

Sample S6 R.MITGVPWYS[+810.264]TR.L

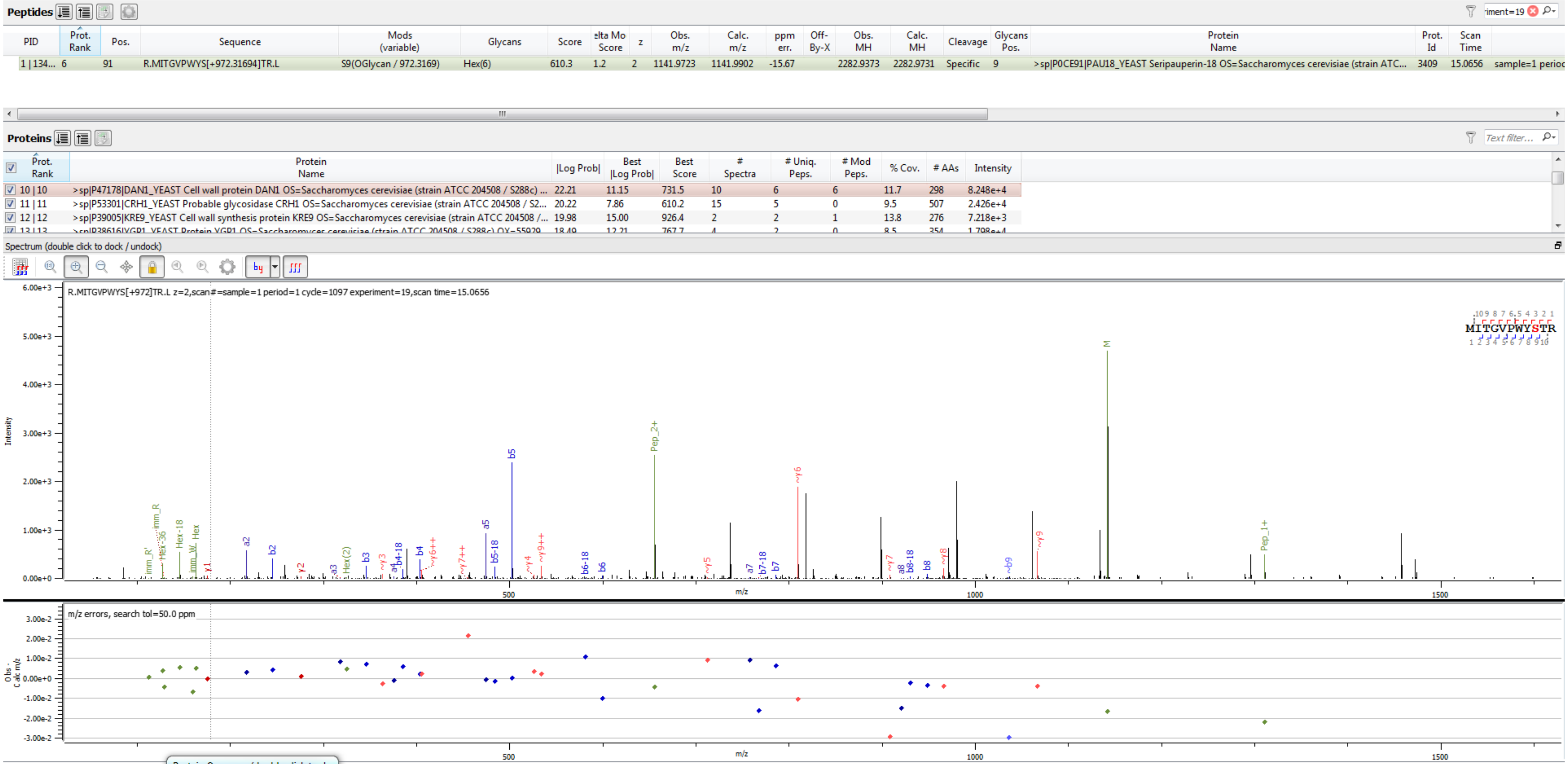

Sample S6 R.MITGVPWYS[+972.317]TR.L

Peptides iment=11

PID	Prot. Rank	Pos.	Sequence	Mods (variable)	Glycans	Score	elta Mo Score	z	Obs. m/z	Calc. m/z	ppm err.	Off-By-X	Obs. MH	Calc. MH	Cleavage	Glycans Pos.	Protein Name	Prot. Id	Scan Time	
1 | 191...	4	91	R.MITGVPWYS[+972.31694]TR.L	S9(OGlycan / 972.3169)	Hex(6)	636.6	1.2	3	761.6496	761.6625	-16.96		2282.9344	2282.9731	Specific	9	>sp|P0CE91|PAU18_YEAST Seripauperin-18 OS=Saccharomyces cerevisiae (strain ATC...	3409	14.9982	sample=1 perioc

Proteins Text filter...

Prot. Rank	Protein Name	|Log Prob|	Best |Log Prob|	Best Score	# Spectra	# Uniq. Peps.	# Mod Peps.	% Cov.	# AAs	Intensity
1 | 1	>sp|P32612|PAU2_YEAST Seripauperin-2 OS=Saccharomyces cerevisiae (strain ATCC 204508 / S288c) OX=559...	304.25	17.48	982.9	381	64	61	67.5	120	1.839e+7
2 | 2	>sp|P0CE85|PAU19_YEAST Seripauperin-19 OS=Saccharomyces cerevisiae (strain ATCC 204508 / S288c) OX=5...	138.61	11.51	683.8	93	32	31	42.7	124	3.148e+6
3 | 3	>sp|P43575|PAU5_YEAST Seripauperin-5 OS=Saccharomyces cerevisiae (strain ATCC 204508 / S288c) OX=559...	129.60	12.22	721.6	114	28	27	53.3	122	7.378e+6
4 | 4	>sp|P0CE91|PAU18_YEAST Seripauperin-18 OS=Saccharomyces cerevisiae (strain ATCC 204508 / S288c) OX=5...	62.54	11.00	666.6	13	9	9	9.2	120	3.115e+5

Spectrum (double click to dock / undock)

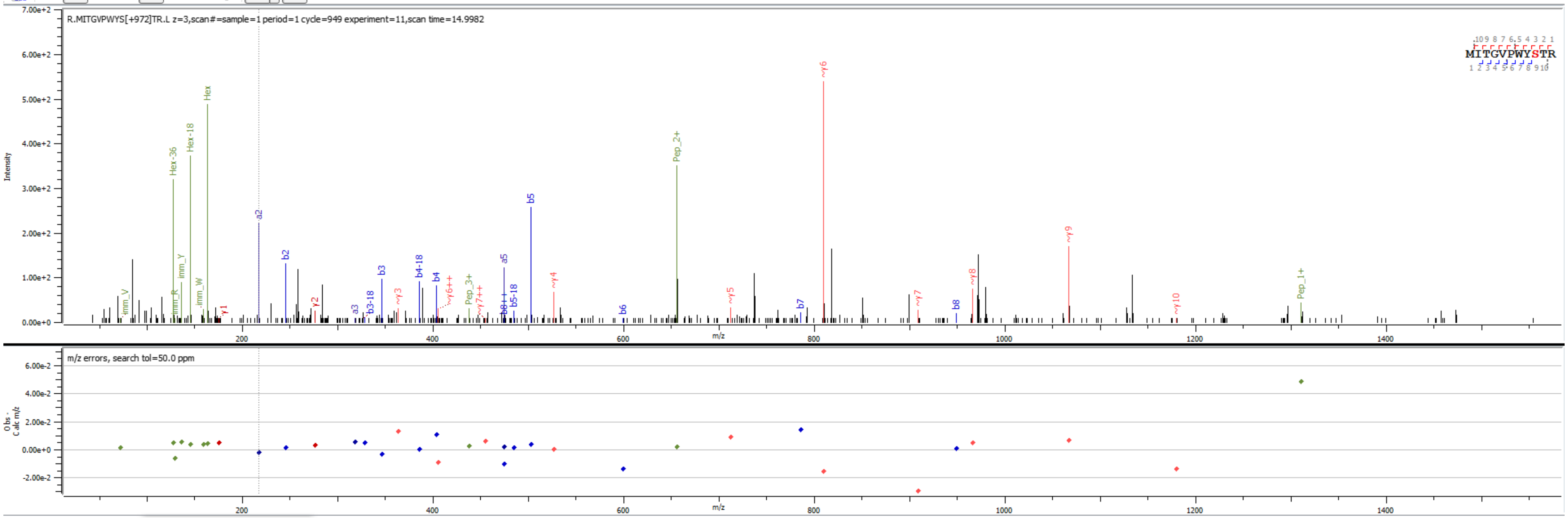

Sample S4 R.MITGVPWYS[+972.317]TR.L

Peptides

PID	Prot. Rank	Pos.	Sequence	Mods (variable)	Glycans	Score	elta Mo Score	z	Obs. m/z	Calc. m/z	ppm err.	Off-By-X	Obs. MH	Calc. MH	Cleavage	Glycans Pos.	Protein Name	Prot. Id	Scan Time	
1 | 191...	4	91	R.MITGVPWYS[+1134.36976]TR.L	S9(OGlycan / 1134.3698)	Hex(7)	542.3	1.2	2	1223.0150	1223.0166	-1.30		2445.0227	2445.0259	Specific	9	>sp|P0CE91|PAU18_YEAST Seripauperin-18 OS=Saccharomyces cerevisiae (strain ATC...	3409	14.9209	sample=1 perioc

Proteins

Prot. Rank	Protein Name	|Log Prob|	Best |Log Prob|	Best Score	# Spectra	# Uniq. Peps.	# Mod Peps.	% Cov.	# AAs	Intensity
1 | 1	>sp|P32612|PAU2_YEAST Seripauperin-2 OS=Saccharomyces cerevisiae (strain ATCC 204508 / S288c) OX=559...	304.25	17.48	982.9	381	64	61	67.5	120	1.839e+7
2 | 2	>sp|P0CE85|PAU19_YEAST Seripauperin-19 OS=Saccharomyces cerevisiae (strain ATCC 204508 / S288c) OX=5...	138.61	11.51	683.8	93	32	31	42.7	124	3.148e+6
3 | 3	>sp|P43575|PAU5_YEAST Seripauperin-5 OS=Saccharomyces cerevisiae (strain ATCC 204508 / S288c) OX=559...	129.60	12.22	721.6	114	28	27	53.3	122	7.378e+6
4 | 4	>sp|P0CE91|PAU18_YEAST Seripauperin-18 OS=Saccharomyces cerevisiae (strain ATCC 204508 / S288c) OX=5...	62.54	11.00	666.6	13	9	9	9.2	120	3.115e+5

Spectrum (double click to dock / undock)

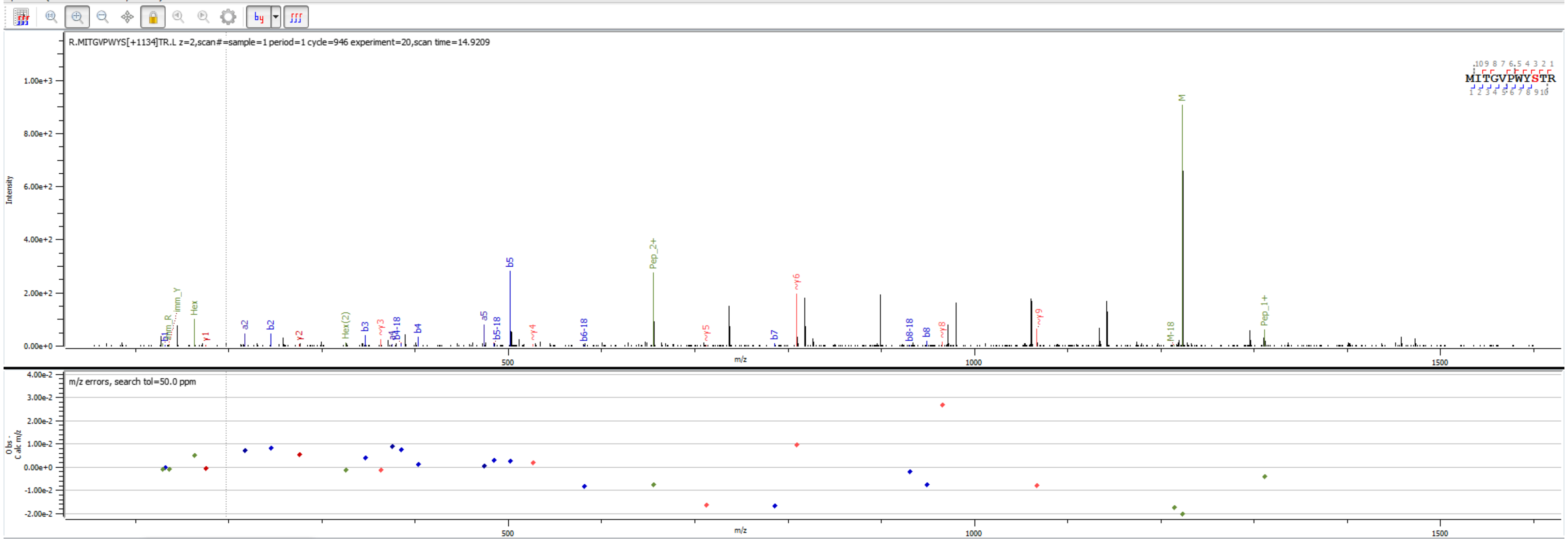

Sample S4

Peptides

PID	Prot. Rank	Pos.	Sequence	Mods (variable)	Glycans	Score	elta Mo Score	z	Obs. m/z	Calc. m/z	ppm err.	Off-By-X	Obs. MH	Calc. MH	Cleavage	Glycans Pos.	Protein Name	Prot. Id	Scan Time	
1 | 199...	5	91	R.MITGVPWYS[+1134.36976]TR.L	S9(OGlycan / 1134.3698)	Hex(7)	621.0	1.2	3	815.6651	815.6802	-18.44		2444.9808	2445.0259	Specific	9	>sp|P0CE91|PAU18_YEAST Seripauperin-18 OS=Saccharomyces cerevisiae (strain ATC...	3409	14.7414	sample=1 perioc

Proteins

Prot. Rank	Protein Name	|Log Prob|	Best |Log Prob|	Best Score	# Spectra	# Uniq. Peps.	# Mod Peps.	% Cov.	# AAs	Intensity
1 | 1	>sp|Q3E770|PAU9_YEAST Seripauperin-9 OS=Saccharomyces cerevisiae (strain ATCC 204508 / S288c) OX=559...	248.32	15.68	955.8	342	58	55	67.5	120	1.797e+7

Spectrum (double click to dock / undock)

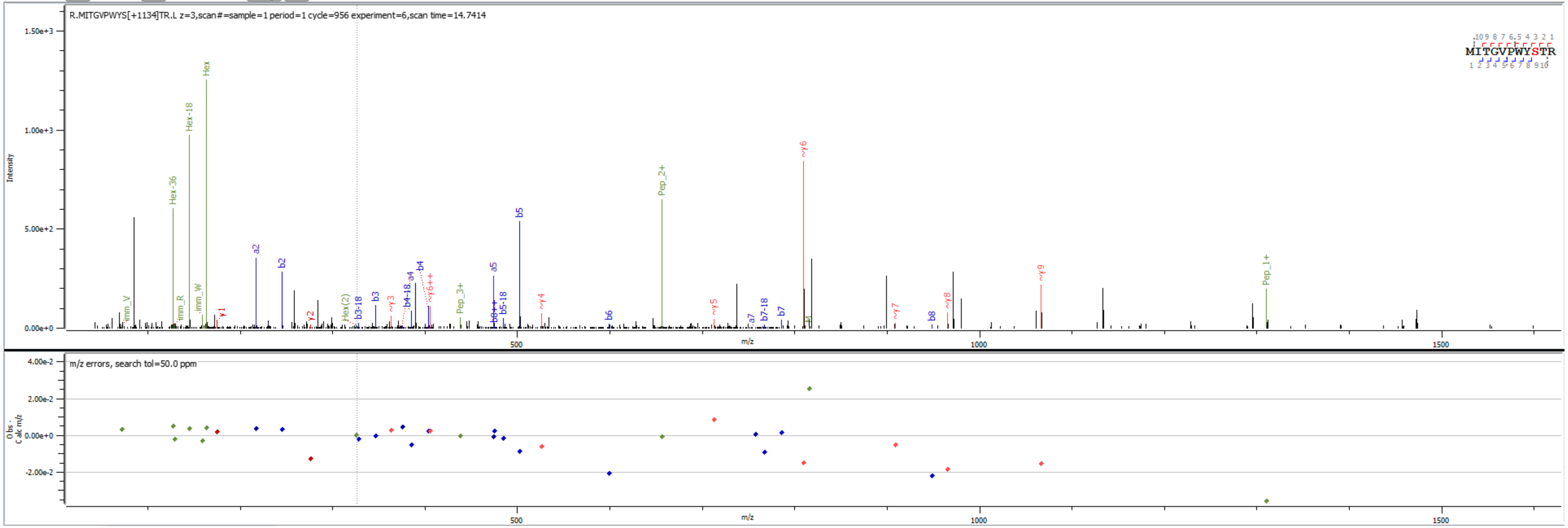

Sample S3 R.MITGVPWYS[+1134.370]TR.L

Peptides

PID	Prot. Rank	Pos.	Sequence	Mods (variable)	Glycans	Score	elta Mo Score	z	Obs. m/z	Calc. m/z	ppm err.	Off-By-X	Obs. MH	Calc. MH	Cleavage	Glycans Pos.	Protein Name	Prot. Id	Scan Time	
1 | 540...	1	47	R.AHLAEYYS[+162.05282]F.Q	S8(OGlycan / 162.0528)	Hex(1)	545.3	277.6	2	631.7762	631.7824	-9.85		1262.5451	1262.5575	CRagged	8	>sp|P43575|PAU5_YEAST Seripauperin-5 OS=Saccharomyces cerevisiae (strain ATCC 2...	3411	15.0035	sample=1 perioc

Proteins

	Prot. Rank	Protein Name	|Log Prob|	Best |Log Prob|	Best Score	# Spectra	# Uniq. Peps.	# Mod Peps.	% Cov.	# AAs	Intensity
☑	1 | 1	>sp|P43575|PAU5_YEAST Seripauperin-5 OS=Saccharomyces cerevisiae (strain ATCC 204508 / S288c) OX=559...	327.47	16.74	1004.1	632	70	67	68.9	122	3.742e+7
☑	2 | 2	>sp|P32612|PAU2_YEAST Seripauperin-2 OS=Saccharomyces cerevisiae (strain ATCC 204508 / S288c) OX=559...	178.33	13.24	726.5	270	60	58	58.3	120	7.688e+6

Spectrum (double click to dock / undock)

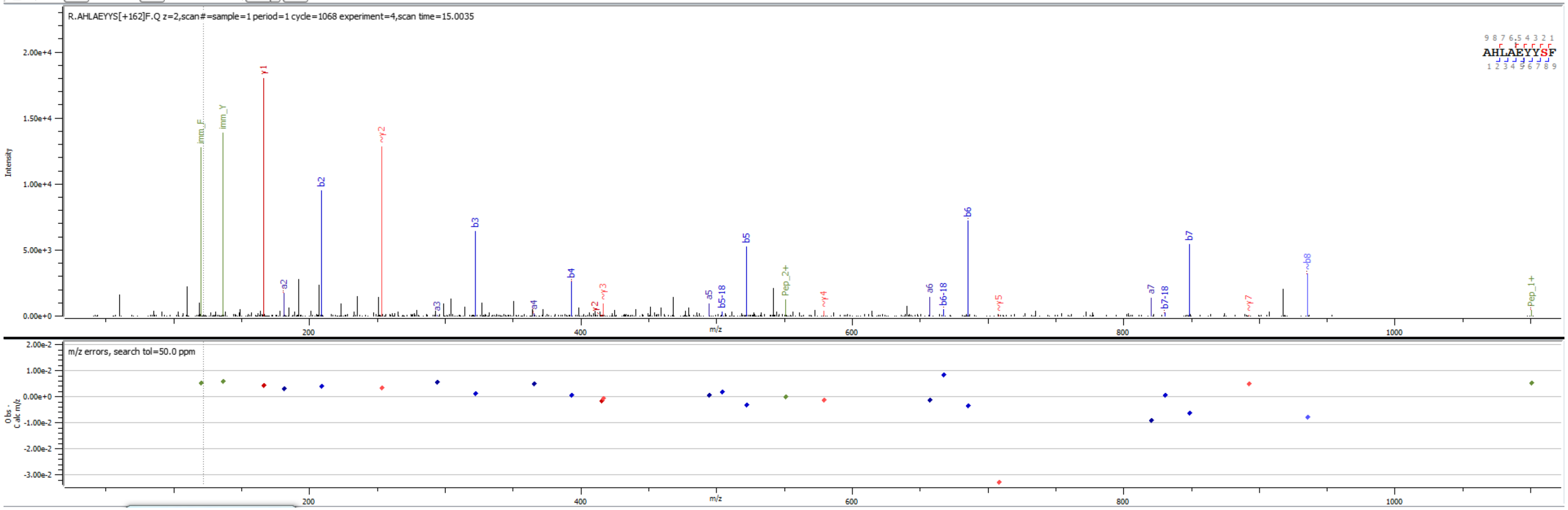

Sample S1 R.AHLAEYYS[+162.053]F.Q

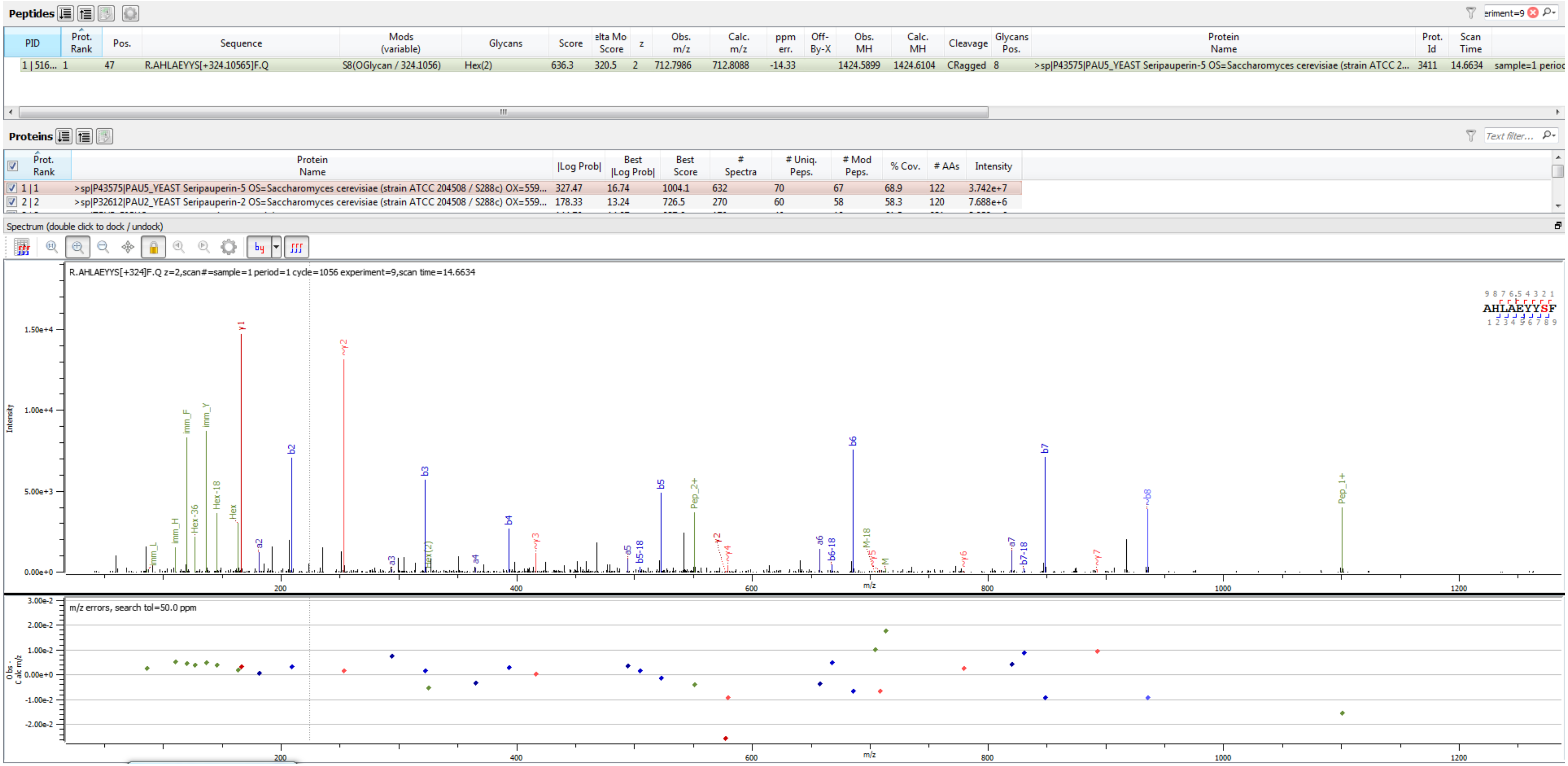

Sample S1 R.AHLAEYYS[+324.106]F.Q

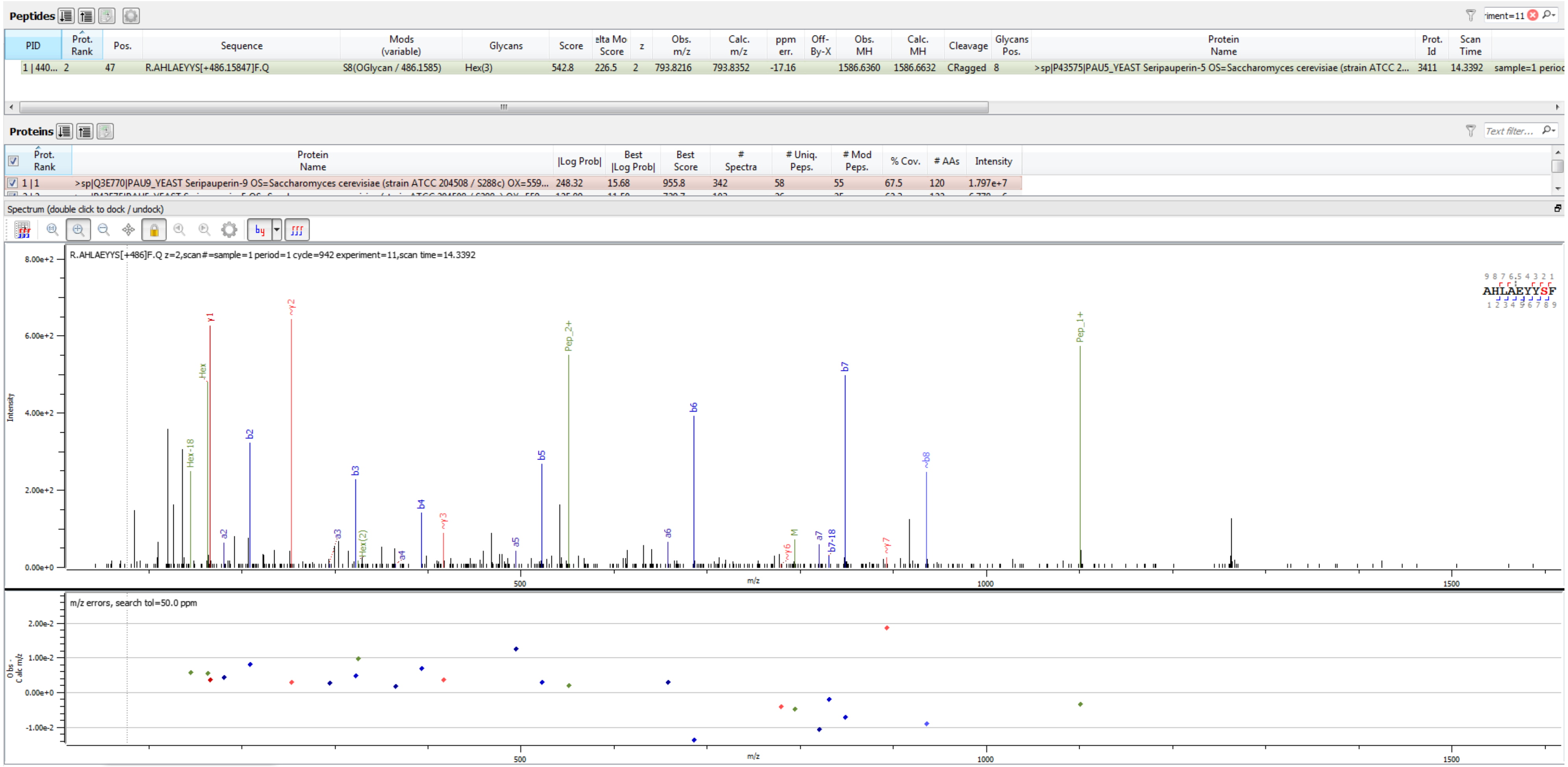

Sample S3 R.AHLAEYYS[+486.158]F.Q

Peptides

PID	Prot. Rank	Pos.	Sequence	Mods (variable)	Glycans	Score	elta Mo Score	z	Obs. m/z	Calc. m/z	ppm err.	Off-By-X	Obs. MH	Calc. MH	Cleavage	Glycans Pos.	Protein Name	Prot. Id	Scan Time	
1 | 108...	1	47	R.AHLAEYYS[+648.21129]F.Q	S8(OGlycan / 648.2113)	Hex(4)	451.9	184.4	2	874.8477	874.8616	-16.00		1748.6880	1748.7160	CRagged	8	>sp|P43575|PAU5_YEAST Seripauperin-5 OS=Saccharomyces cerevisiae (strain ATCC 2...	3411	14.6684	sample=1 perioc

Proteins

Prot. Rank	Protein Name	|Log Prob|	Best |Log Prob|	Best Score	# Spectra	# Uniq. Peps.	# Mod Peps.	% Cov.	# AAs	Intensity
1 | 1	>sp|P43575|PAU5_YEAST Seripauperin-5 OS=Saccharomyces cerevisiae (strain ATCC 204508 / S288c) OX=559...	327.47	16.74	1004.1	632	70	67	68.9	122	3.742e+7
2 | 2	>sp|P32612|PAU2_YEAST Seripauperin-2 OS=Saccharomyces cerevisiae (strain ATCC 204508 / S288c) OX=559...	178.33	13.24	726.5	270	60	58	58.3	120	7.688e+6

Spectrum (double click to dock / undock)

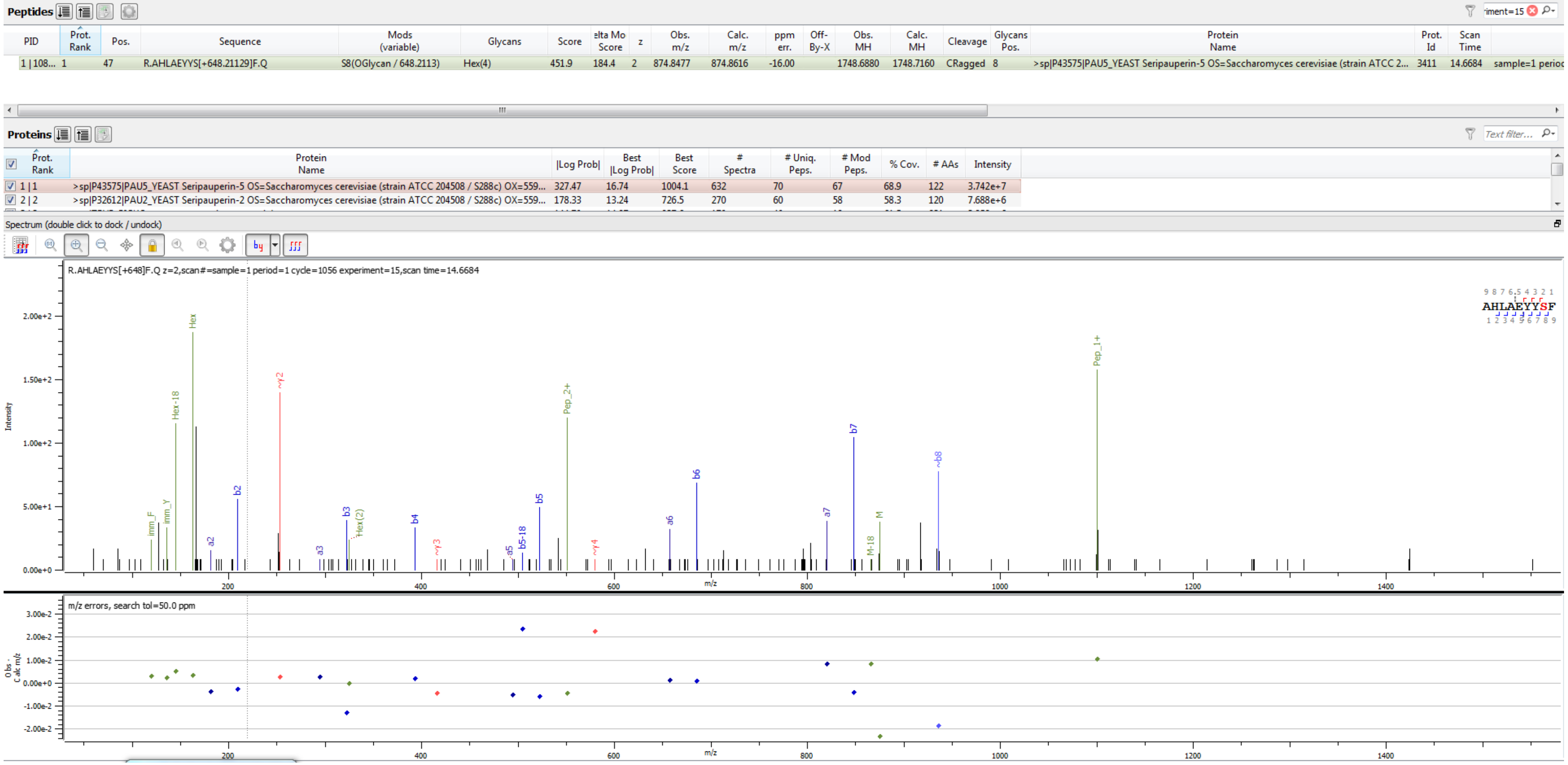

Sample S1 R.AHLAEYYS[+648.211]F.Q

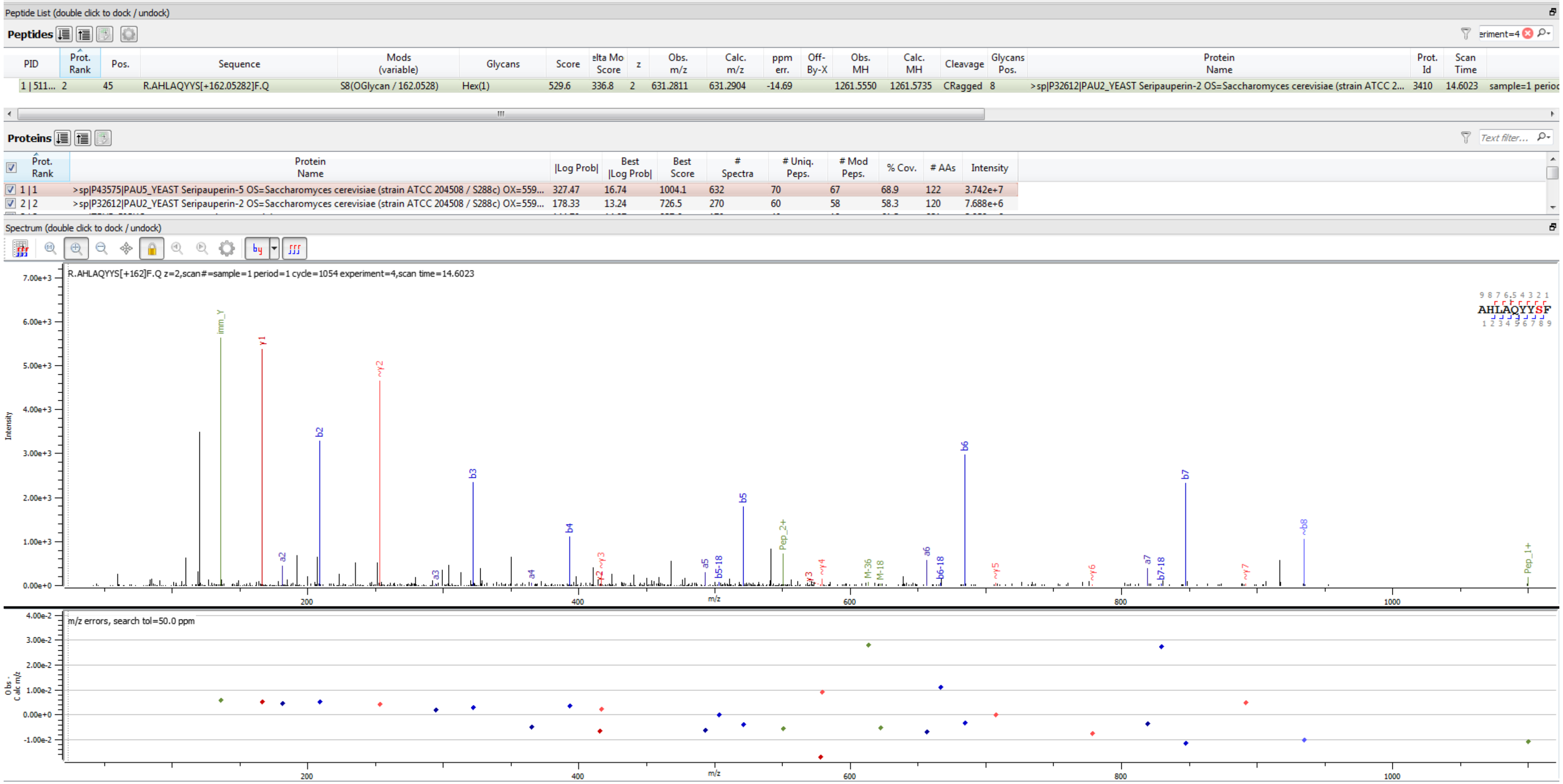

Sample S1 R.AHLAQYYS[+162.053]F.Q

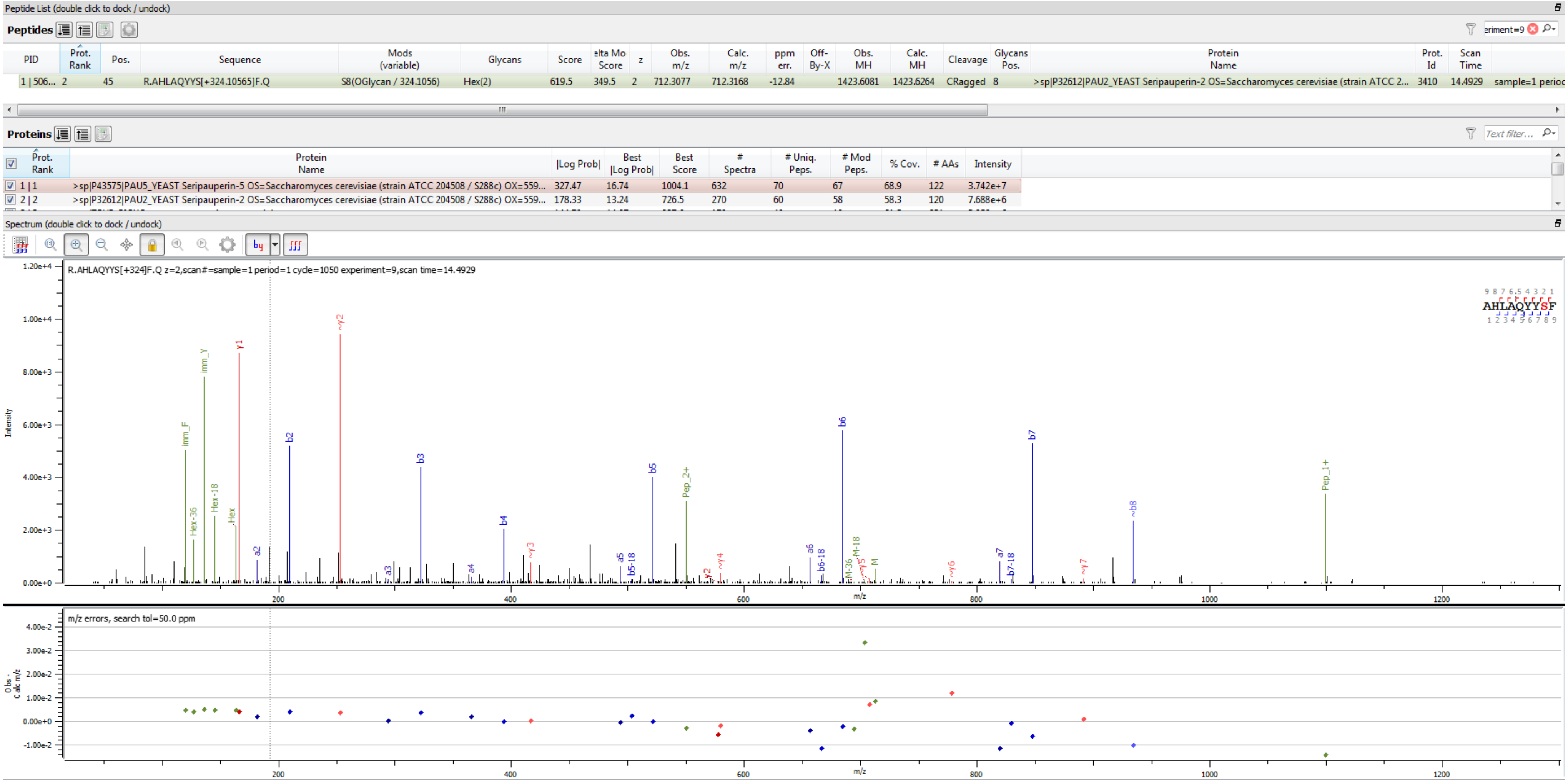

Sample S1 R.AHLAQYYS[+324.106]F.Q

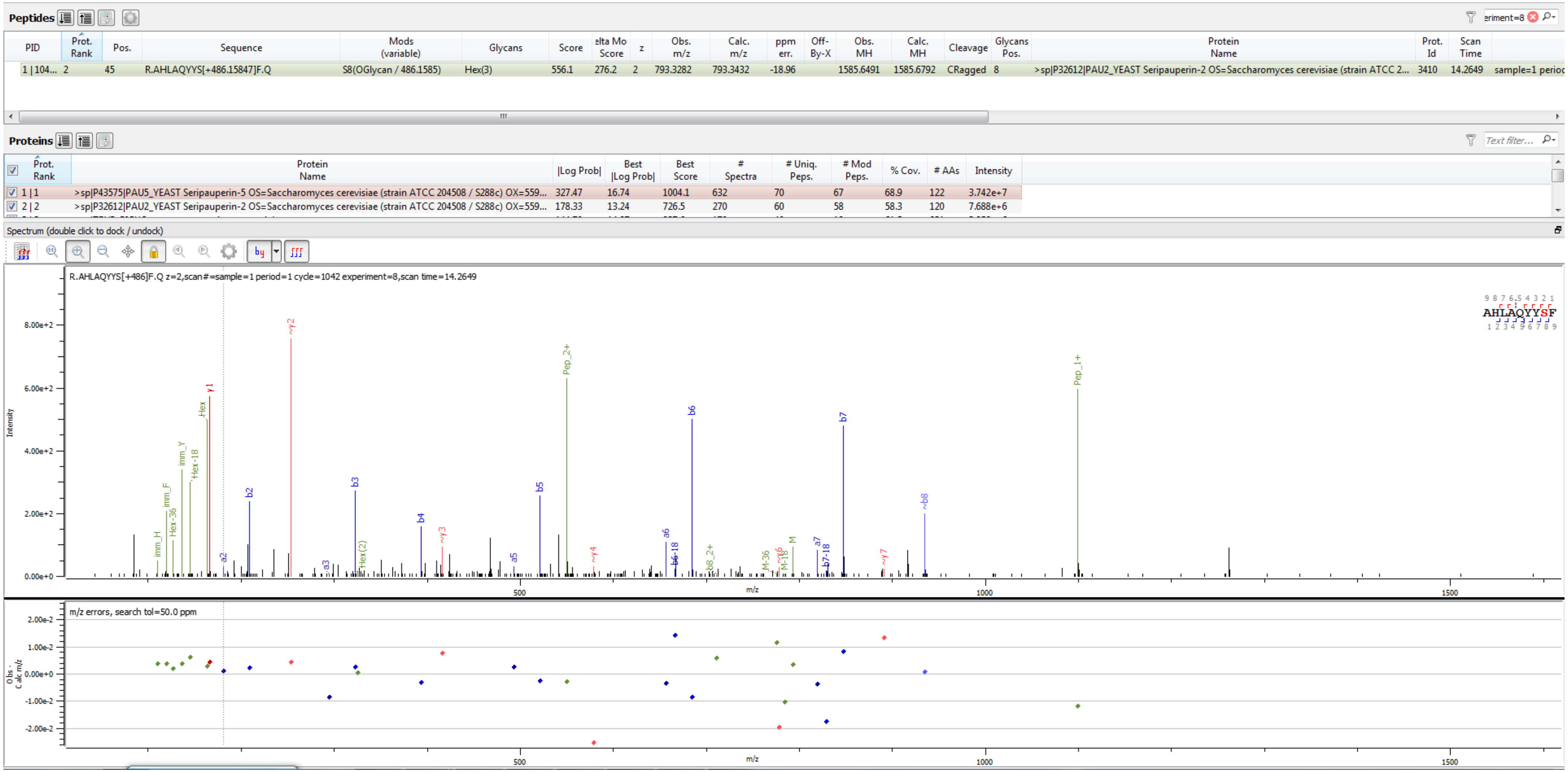
Peptides

PID	Prot. Rank	Pos.	Sequence	Mods (variable)	Glycans	Score	elta Mo Score	z	Obs. m/z	Calc. m/z	ppm err.	Off-By-X	Obs. MH	Calc. MH	Cleavage	Glycans Pos.	Protein Name	Prot. Id	Scan Time	
1 | 104...	2	45	R.AHLAQYYS[+486.15847]F.Q	S8(OGlycan / 486.1585)	Hex(3)	556.1	276.2	2	793.3282	793.3432	-18.96		1585.6491	1585.6792	CRagged	8	>sp|P32612|PAU2_YEAST Seripauperin-2 OS=Saccharomyces cerevisiae (strain ATCC 2...	3410	14.2649	sample=1 perioc

Proteins

Prot. Rank	Protein Name	|Log Prob|	Best |Log Prob|	Best Score	# Spectra	# Uniq. Peps.	# Mod Peps.	% Cov.	# AAs	Intensity
1 | 1	>sp|P43575|PAU5_YEAST Seripauperin-5 OS=Saccharomyces cerevisiae (strain ATCC 204508 / S288c) OX=559...	327.47	16.74	1004.1	632	70	67	68.9	122	3.742e+7
2 | 2	>sp|P32612|PAU2_YEAST Seripauperin-2 OS=Saccharomyces cerevisiae (strain ATCC 204508 / S288c) OX=559...	178.33	13.24	726.5	270	60	58	58.3	120	7.688e+6

Sample S1 R.AHLAQYYS[+486.158]F.Q

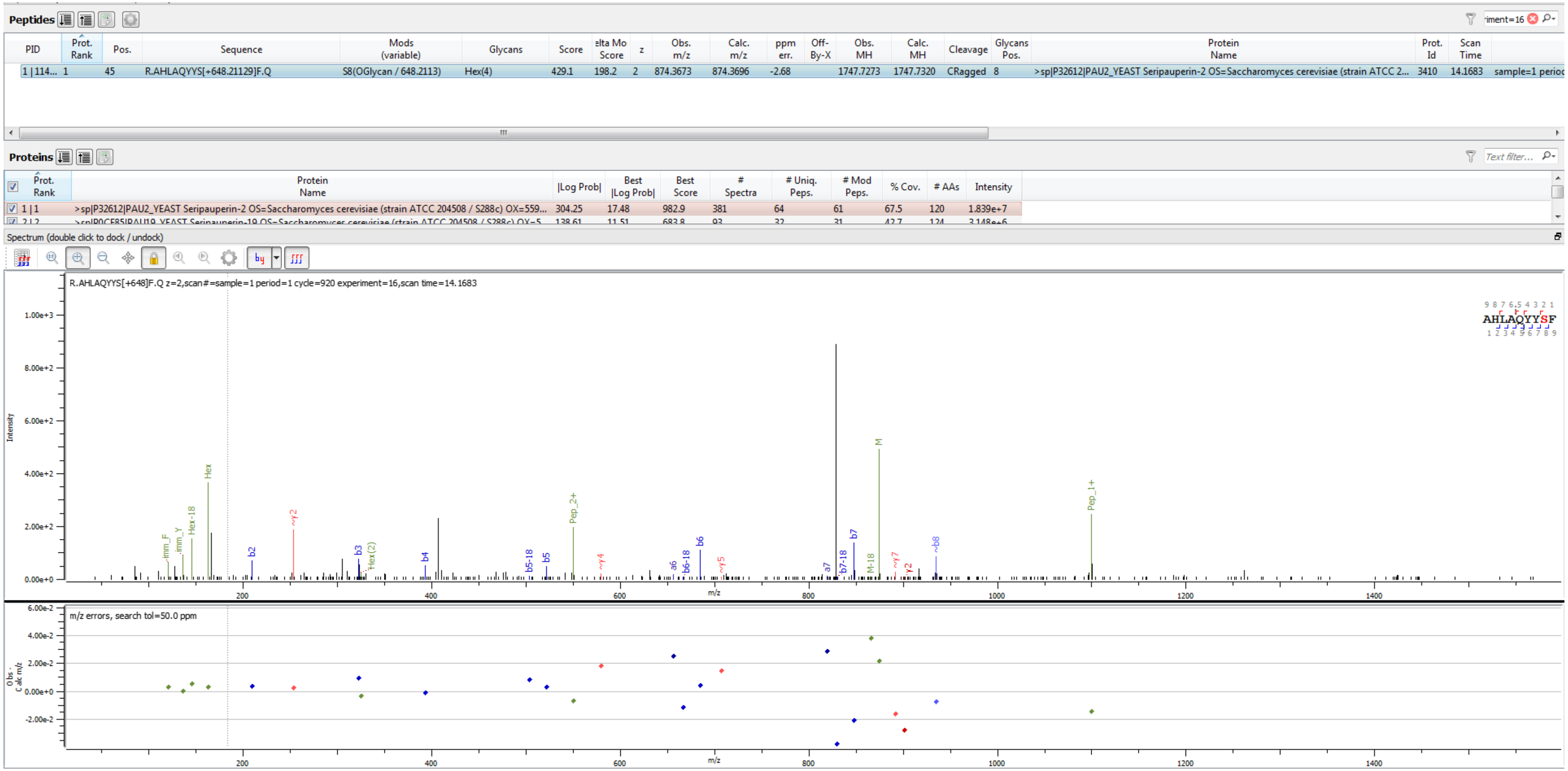

Sample S4 R.AHLAQYYS[+648.211]F.Q

Peptides

PID	Prot. Rank	Pos.	Sequence	Mods (variable)	Glycans	Score	elta Mo Score	z	Obs. m/z	Calc. m/z	ppm err.	Off-By-X	Obs. MH	Calc. MH	Cleavage	Glycans Pos.	Protein Name	Prot. Id	Scan Time	
1 | 6483	1	102	R.LKPAISSALS[+486.15847]K.D	S10(OGlycan / 486.1585)	Hex(3)	468.1	0.0	2	800.9109	800.9244	-16.89		1600.8144	1600.8415	Specific	10	>sp|Q3E770|PAU9_YEAST Seripauperin-9 OS=Saccharomyces cerevisiae (strain ATCC 2...	3461	10.6253	sample=1 perioc

Proteins

Prot. Rank	Protein Name	|Log Prob|	Best |Log Prob|	Best Score	# Spectra	# Uniq. Peps.	# Mod Peps.	% Cov.	# AAs	Intensity
1 | 1	>sp|Q3E770|PAU9_YEAST Seripauperin-9 OS=Saccharomyces cerevisiae (strain ATCC 204508 / S288c) OX=559...	248.32	15.68	955.8	342	58	55	67.5	120	1.797e+7

Spectrum (double click to dock / undock)

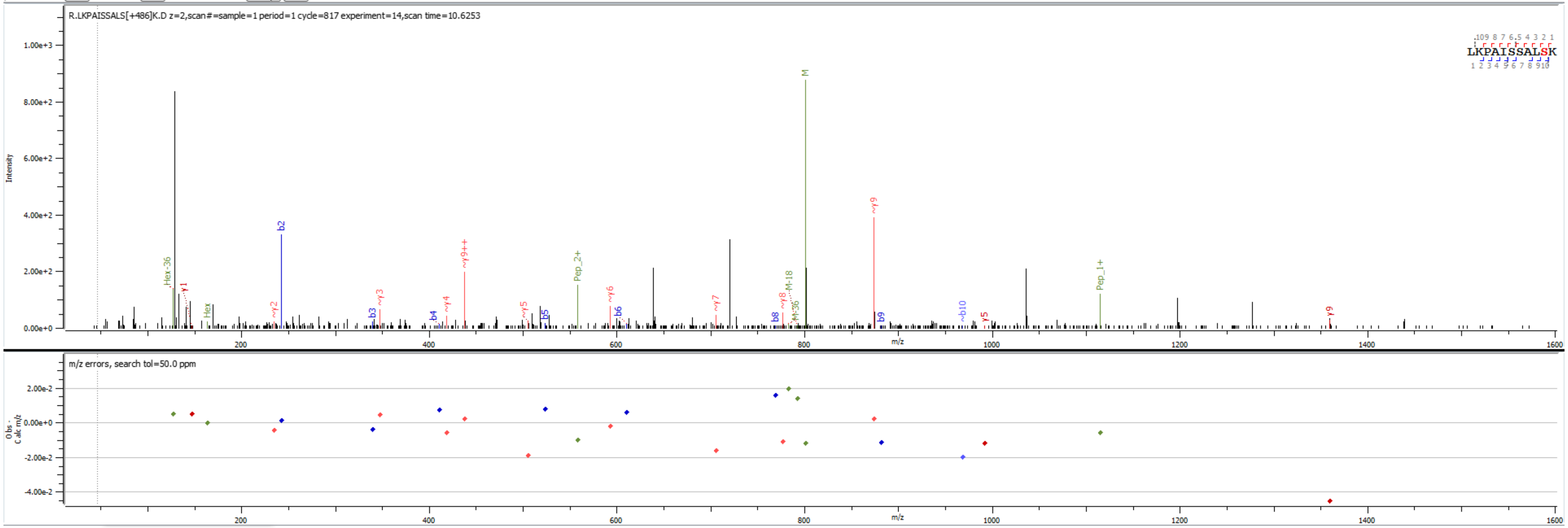

Sample S3 R.LKPAISSALS[+486.158]K.D

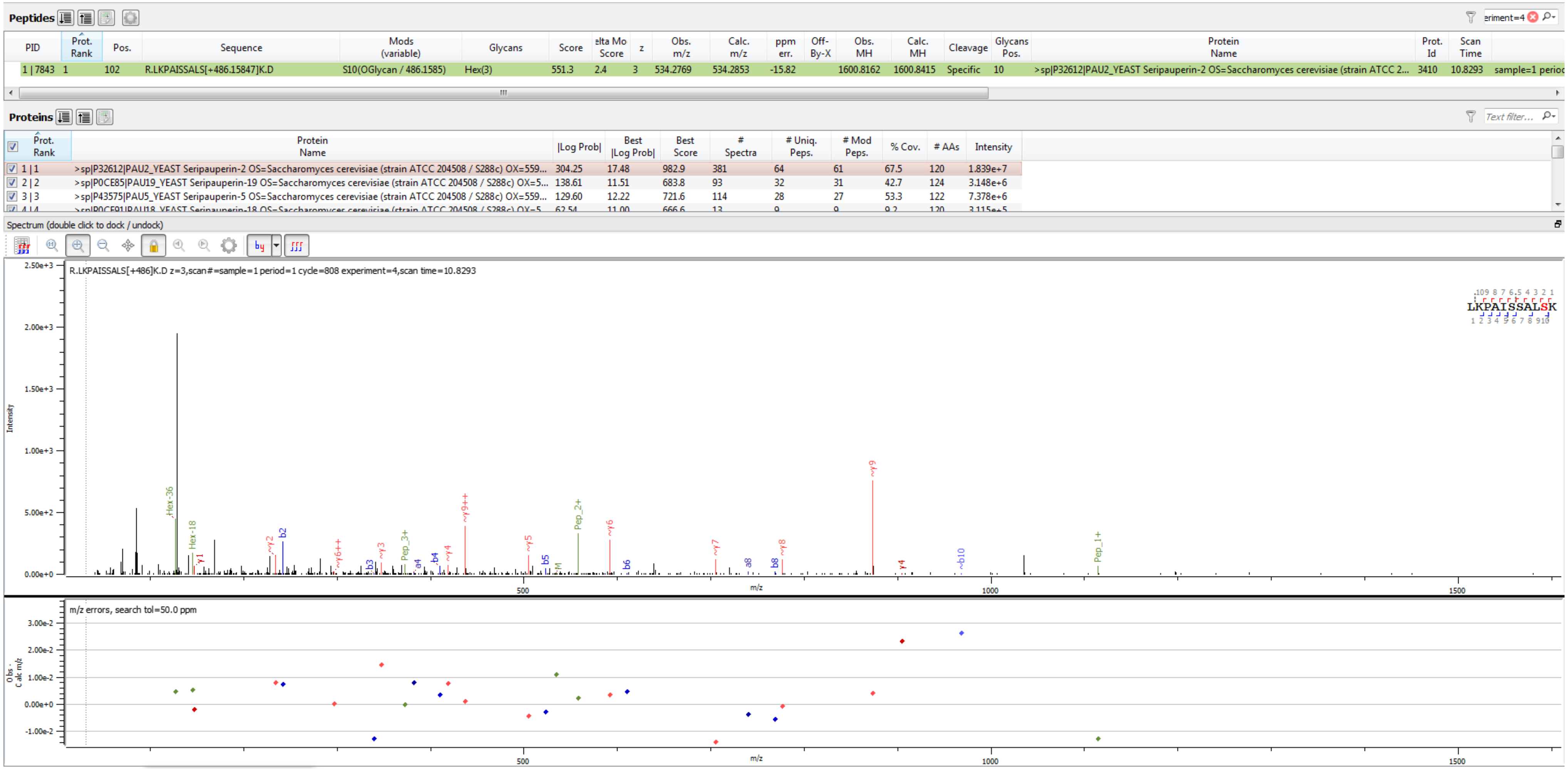

Sample S4 R.LKPAISSALS[+486.158]K.D

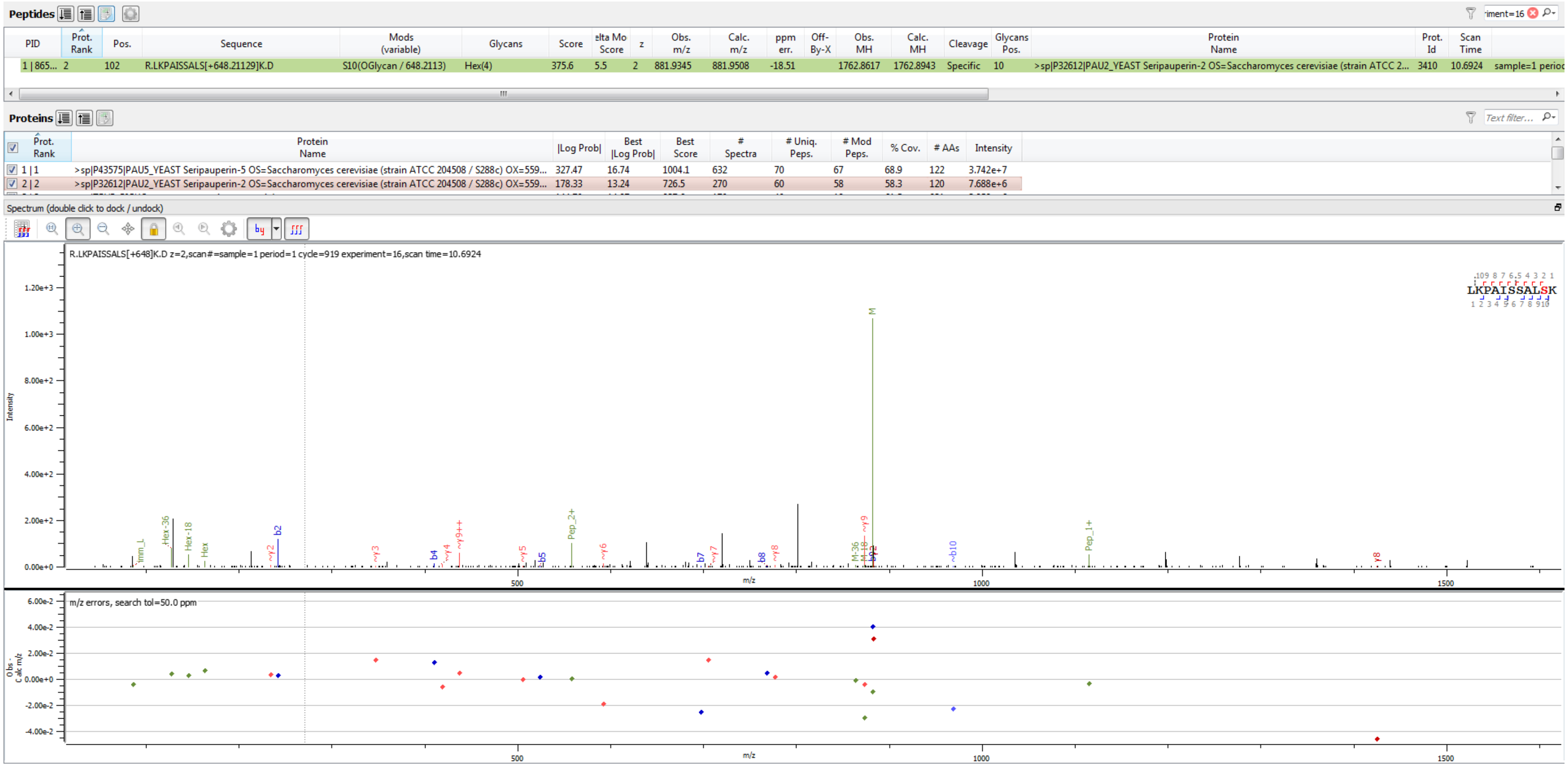

Sample S1 R.LKPAISSALS[+648.211]K.D

Peptides

PID	Prot. Rank	Pos.	Sequence	Mods (variable)	Glycans	Score	elta Mo Score	z	Obs. m/z	Calc. m/z	ppm err.	Off-By-X	Obs. MH	Calc. MH	Cleavage	Glycans Pos.	Protein Name	Prot. Id	Scan Time	
1 | 2290	1	102	R.LKPAISS[+648.21129]ALSK.D	S7(OGlycan / 648.2113)	Hex(4)	485.6	0.0	3	588.2979	588.3030	-8.60		1762.8791	1762.8943	Specific	7	>sp|Q3E770|PAU9_YEAST Seripauperin-9 OS=Saccharomyces cerevisiae (strain ATCC 2...	3461	10.5024	sample=1 perioc

Proteins

Prot. Rank	Protein Name	|Log Prob|	Best |Log Prob|	Best Score	# Spectra	# Uniq. Peps.	# Mod Peps.	% Cov.	# AAs	Intensity
1 | 1	>sp|Q3E770|PAU9_YEAST Seripauperin-9 OS=Saccharomyces cerevisiae (strain ATCC 204508 / S288c) OX=559...	288.60	16.66	988.6	452	61	56	60.8	120	1.023e+7
2 | 2	>sp|Q07987|PAU23_YEAST Seripauperin-23 OS=Saccharomyces cerevisiae (strain ATCC 204508 / S288c) OX=5...	160.38	13.28	718.6	124	39	36	43.5	124	2.323e+6
3 | 3	>sp|P43575|PAU5_YEAST Seripauperin-5 OS=Saccharomyces cerevisiae (strain ATCC 204508 / S288c) OX=559...	142.55	14.15	737.1	167	35	33	42.6	122	6.136e+6

Spectrum (double click to dock / undock)

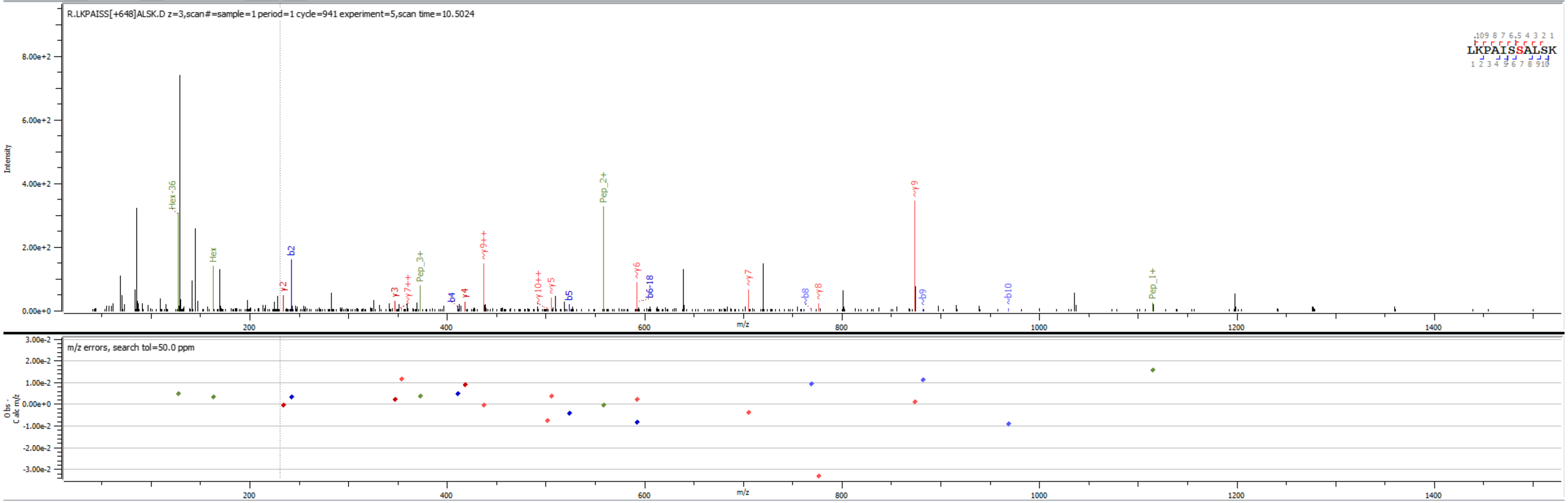

Sample S6 R.LKPAISS[+648.211]ALSK.D

Peptides

PID	Prot. Rank	Pos.	Sequence	Mods (variable)	Glycans	Score	elta Mo Score	z	Obs. m/z	Calc. m/z	ppm err.	Off-By-X	Obs. MH	Calc. MH	Cleavage	Glycans Pos.	Protein Name	Prot. Id	Scan Time	
1 | 881...	1	102	R.LKPAIS[+810.26412]SALSK.D	S6(OGlycan / 810.2641)	Hex(5)	397.6	6.6	2	962.9589	962.9772	-18.96		1924.9106	1924.9471	Specific	6	>sp|P32612|PAU2_YEAST Seripauperin-2 OS=Saccharomyces cerevisiae (strain ATCC 2...	3410	10.0036	sample=1 perioc

Proteins

Prot. Rank	Protein Name	|Log Prob|	Best |Log Prob|	Best Score	# Spectra	# Uniq. Peps.	# Mod Peps.	% Cov.	# AAs	Intensity
1 | 1	>sp|P32612|PAU2_YEAST Seripauperin-2 OS=Saccharomyces cerevisiae (strain ATCC 204508 / S288c) OX=559...	304.25	17.48	982.9	381	64	61	67.5	120	1.839e+7
2 | 2	>sp|P0CE85|PAU19_YEAST Seripauperin-19 OS=Saccharomyces cerevisiae (strain ATCC 204508 / S288c) OX=5...	138.61	11.51	683.8	93	32	31	42.7	124	3.148e+6

Spectrum (double click to dock / undock)

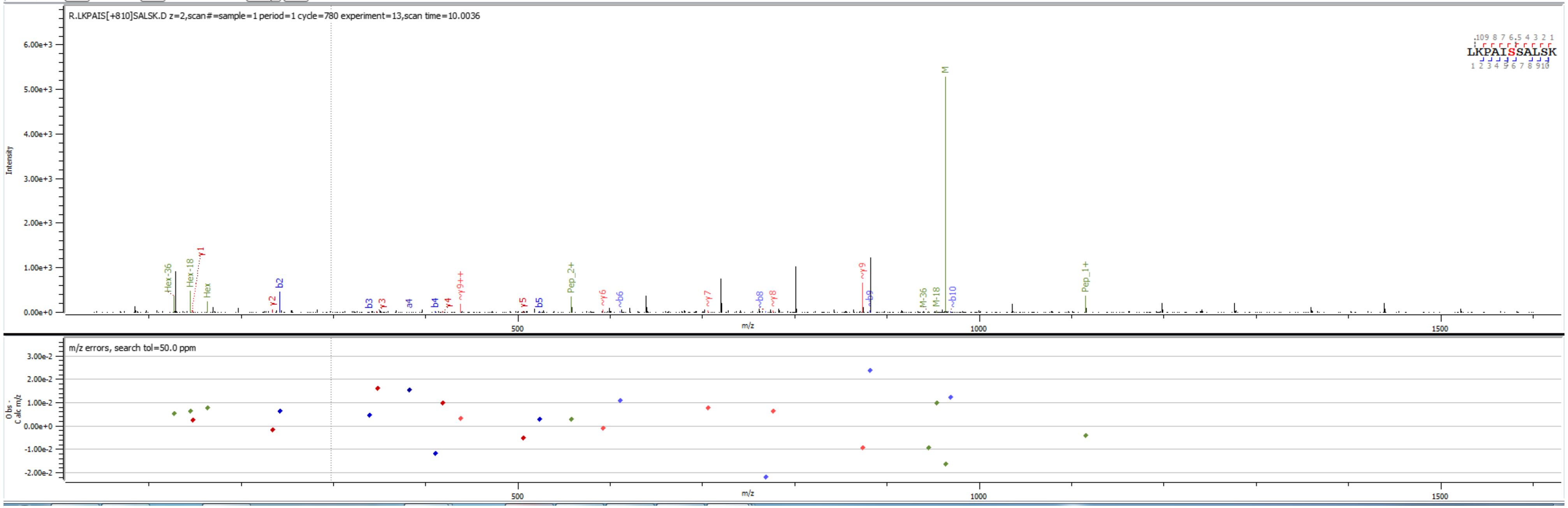

Sample S1 R.LKPAIS[+810.264]SALSK.D

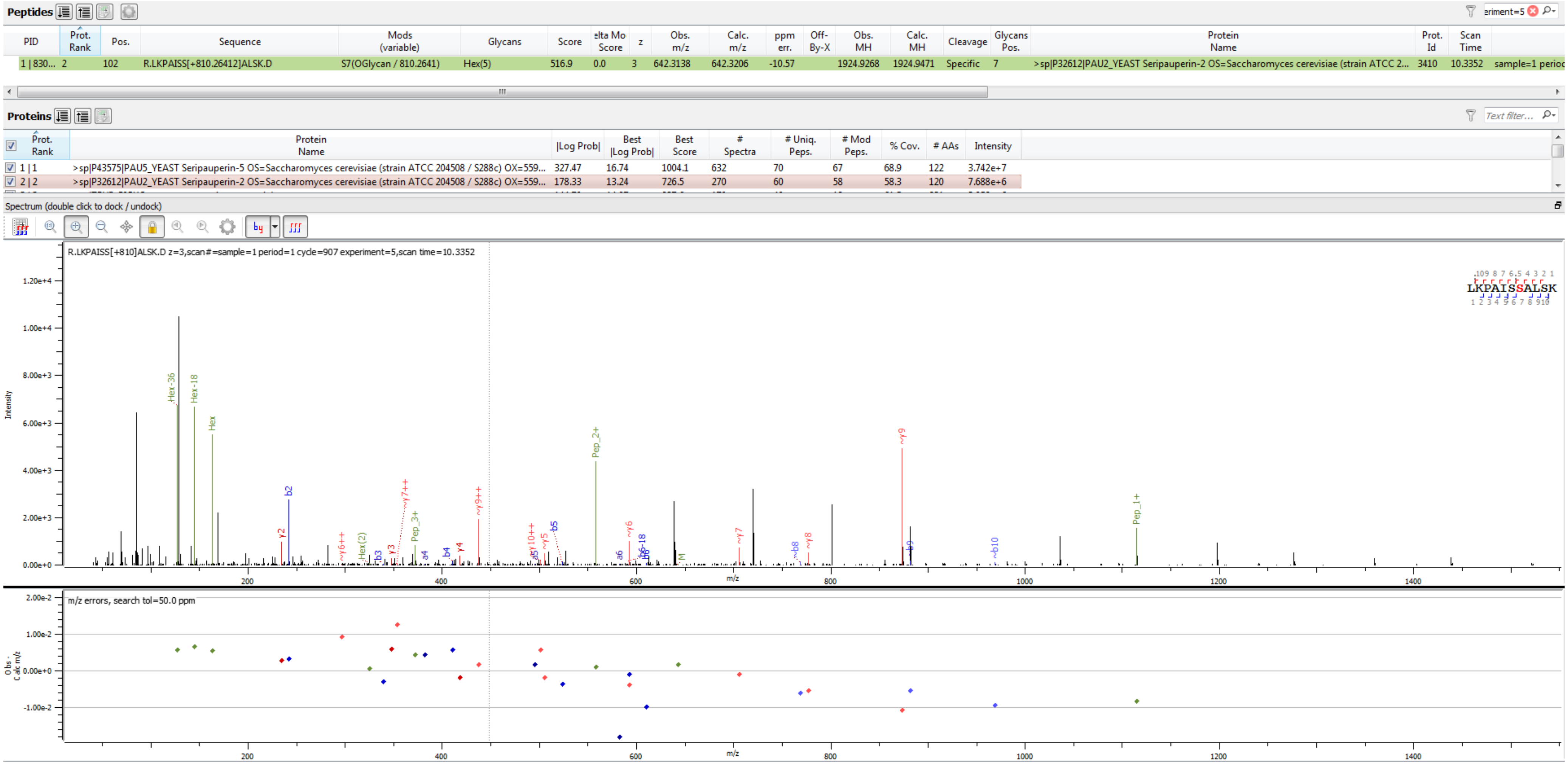

Sample S1 R.LKPAISS[+810.264]ALSK.D

Peptides

PID	Prot. Rank	Pos.	Sequence	Mods (variable)	Glycans	Score	elta Mo Score	z	Obs. m/z	Calc. m/z	ppm err.	Off-By-X	Obs. MH	Calc. MH	Cleavage	Glycans Pos.	Protein Name	Prot. Id	Scan Time	
1 | 893...	1	102	R.LKPAISS[+972.31694]ALSK.D	S7(OGlycan / 972.3169)	Hex(6)	404.6	1.2	2	1043.9853	1044.0036	-17.57		2086.9633	2087.0000	Specific	7	>sp|P32612|PAU2_YEAST Seripauperin-2 OS=Saccharomyces cerevisiae (strain ATCC 2...	3410	10.1262	sample=1 perioc

Proteins

Prot. Rank	Protein Name	|Log Prob|	Best |Log Prob|	Best Score	# Spectra	# Uniq. Peps.	# Mod Peps.	% Cov.	# AAs	Intensity
1 | 1	>sp|P32612|PAU2_YEAST Seripauperin-2 OS=Saccharomyces cerevisiae (strain ATCC 204508 / S288c) OX=559...	304.25	17.48	982.9	381	64	61	67.5	120	1.839e+7
2 | 2	>sp|P0CF85|PAU19_YEAST Seripauperin-19 OS=Saccharomyces cerevisiae (strain ATCC 204508 / S288c) OX=5...	138.61	11.51	683.8	93	32	31	42.7	124	3.148e+6

Spectrum (double click to dock / undock)

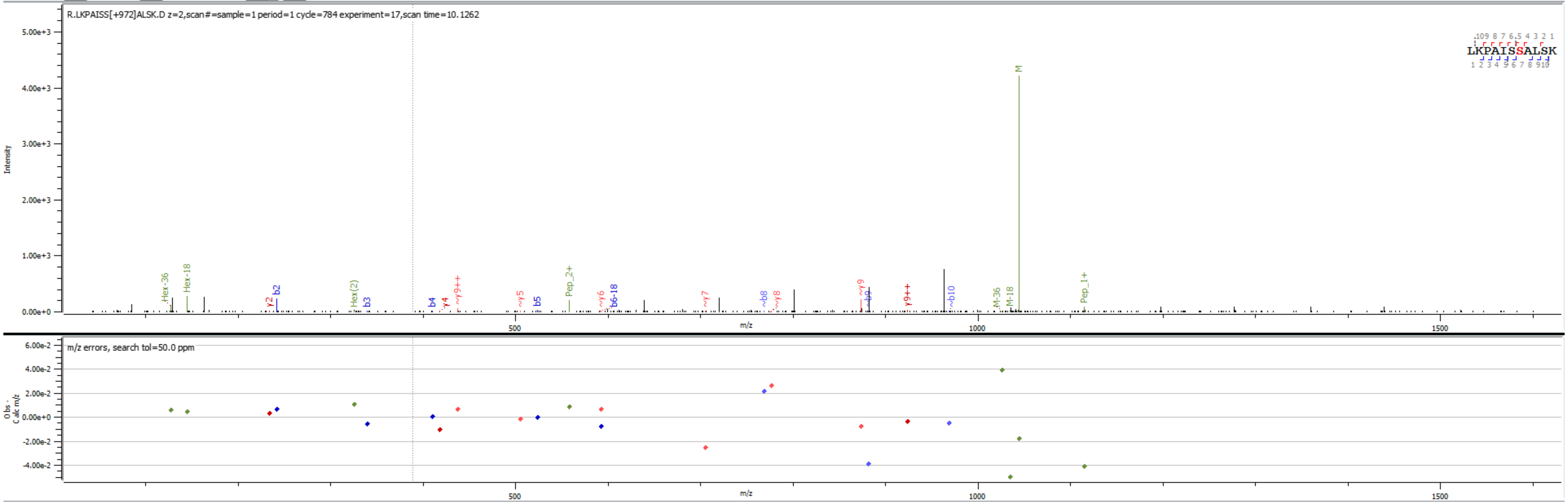

Sample S4 R.LKPAISS[+972.317]ALSK.D

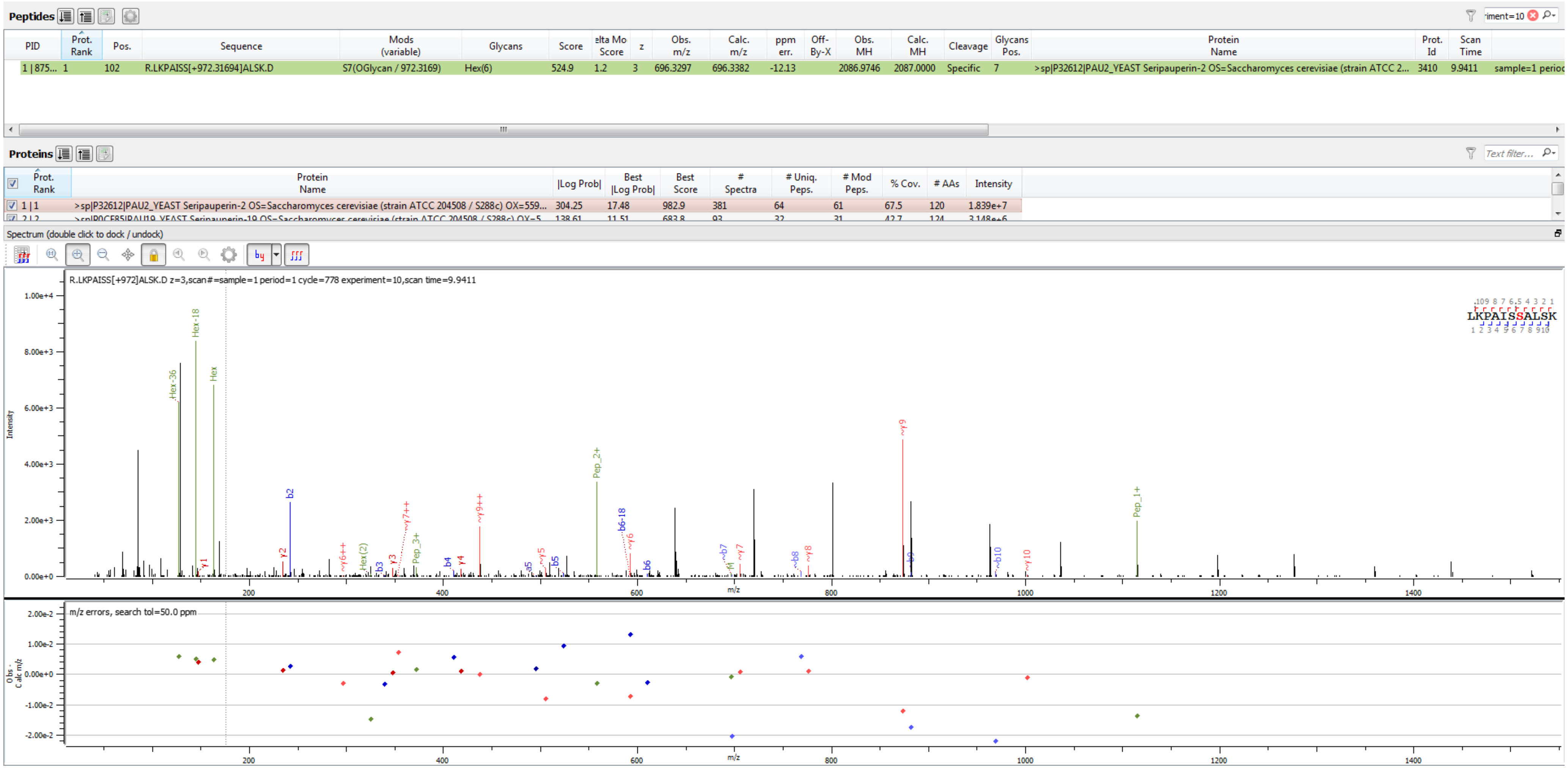

Sample S4 R.LKPAISS[+972.317]ALSK.D

Peptides

PID	Prot. Rank	Pos.	Sequence	Mods (variable)	Glycans	Score	elta Mo Score	z	Obs. m/z	Calc. m/z	ppm err.	Off-By-X	Obs. MH	Calc. MH	Cleavage	Glycans Pos.	Protein Name	Prot. Id	Scan Time	
1 | 169...	1	102	R.LKPAIS[+1134.36976]SALSK.D	S6(OGlycan / 1134.3698)	Hex(7)	512.5	2.0	3	750.3455	750.3558	-13.73		2249.0219	2249.0528	Specific	6	>sp|Q3E770|PAU9_YEAST Seripauperin-9 OS=Saccharomyces cerevisiae (strain ATCC 2...	3461	9.6747	sample=1 perioc

Proteins

Prot. Rank	Protein Name	|Log Prob|	Best |Log Prob|	Best Score	# Spectra	# Uniq. Peps.	# Mod Peps.	% Cov.	# AAs	Intensity
3 | 3	>sp|P0CE85|PAU19_YEAST Seripauperin-19 OS=Saccharomyces cerevisiae (strain ATCC 204508 / S288c) OX=5...	123.82	11.06	692.3	77	31	30	42.7	124	2.619e+6
4 | 4	>sp|K2C1_HUMAN|(Common contaminant protein)	75.52	13.70	921.5	17	8	0	13.8	643	1.827e+5

Spectrum (double click to dock / undock)

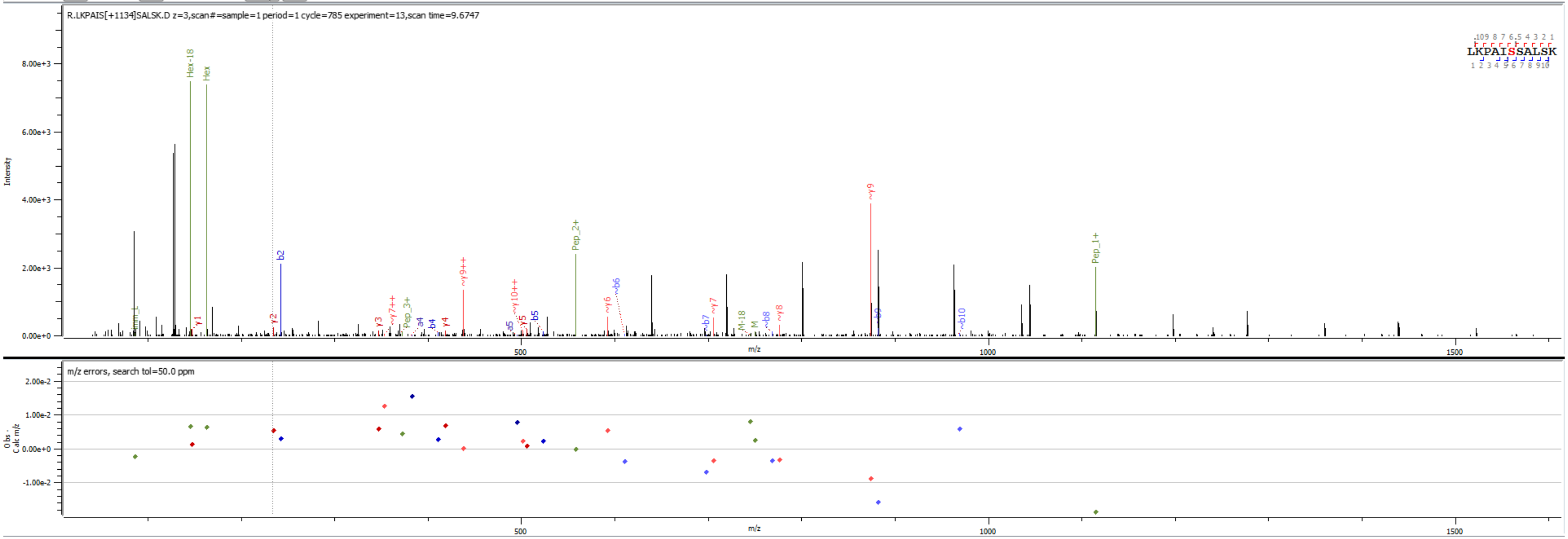

Sample S3 R.LKPAIS[+1134.370]SALSK.D

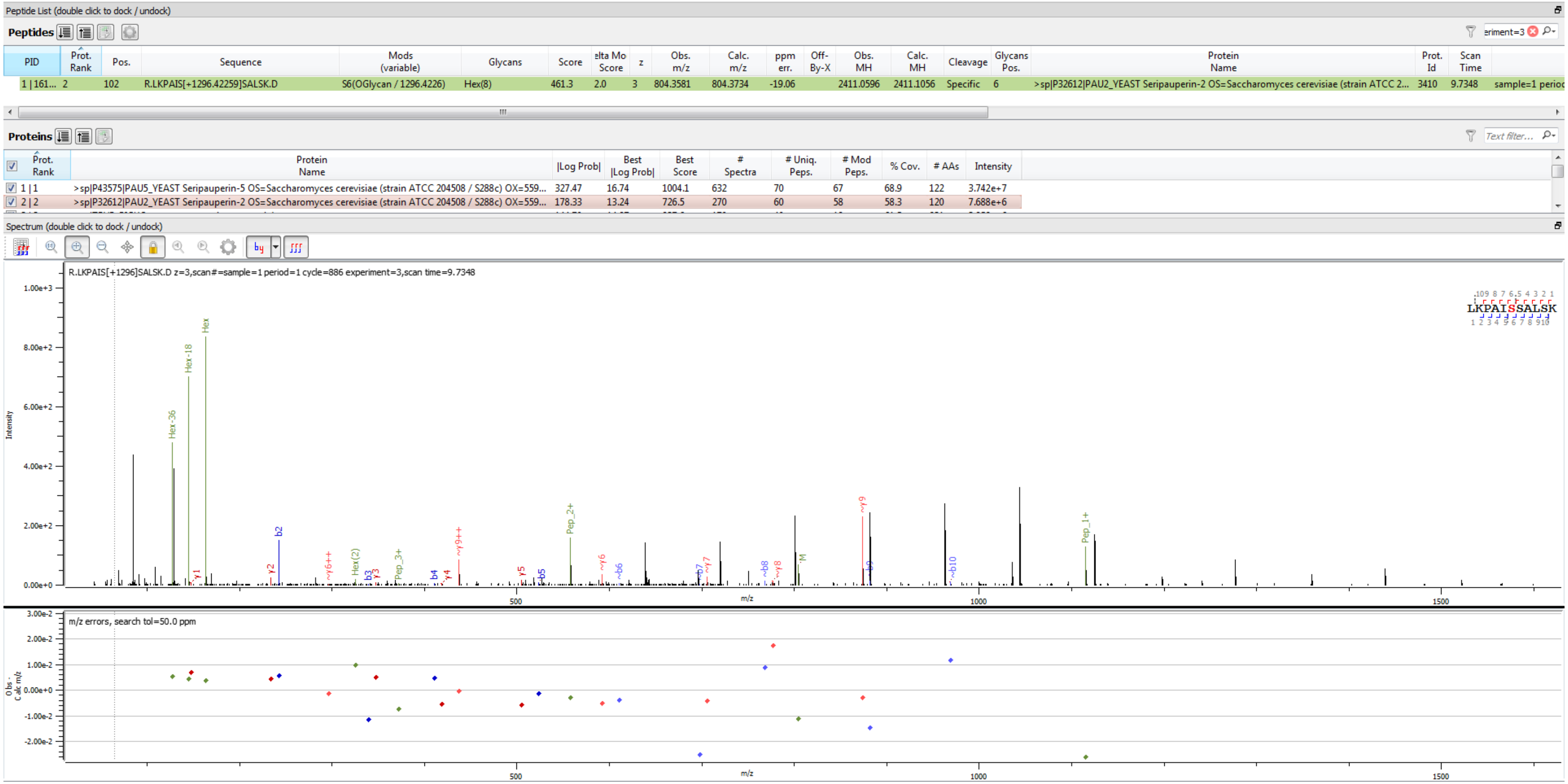

Sample S1 R.LKPAIS[+1296.423]SALSK.D

Peptides

PID	Prot. Rank	Pos.	Sequence	Mods (variable)	Glycans	Score	elta Mo Score	z	Obs. m/z	Calc. m/z	ppm err.	Off-By-X	Obs. MH	Calc. MH	Cleavage	Glycans Pos.	Protein Name	Prot. Id	Scan Time	
1 | 111...	3	95	V.ITGVPWYS[+648.21129]TR.L	S8(OGlycan / 648.2113)	Hex(4)	482.1	0.0	2	914.4069	914.4171	-11.17		1827.8066	1827.8270	NRagged	8	>sp|P0CE85|PAU19_YEAST Seripauperin-19 OS=Saccharomyces cerevisiae (strain ATC...	3420	13.9354	sample=1 perioc

Proteins

	Prot. Rank	Protein Name	|Log Prob|	Best |Log Prob|	Best Score	# Spectra	# Uniq. Peps.	# Mod Peps.	% Cov.	# AAs	Intensity
☑	3 | 3	>sp|P0CE85|PAU19_YEAST Seripauperin-19 OS=Saccharomyces cerevisiae (strain ATCC 204508 / S288c) OX=5...	123.82	11.06	692.3	77	31	30	42.7	124	2.619e+6
☑	4 | 4	>sp|K2C1_HUMAN|(Common contaminant protein)	75.52	13.70	921.5	17	8	0	13.8	643	1.827e+5

Spectrum (double click to dock / undock)

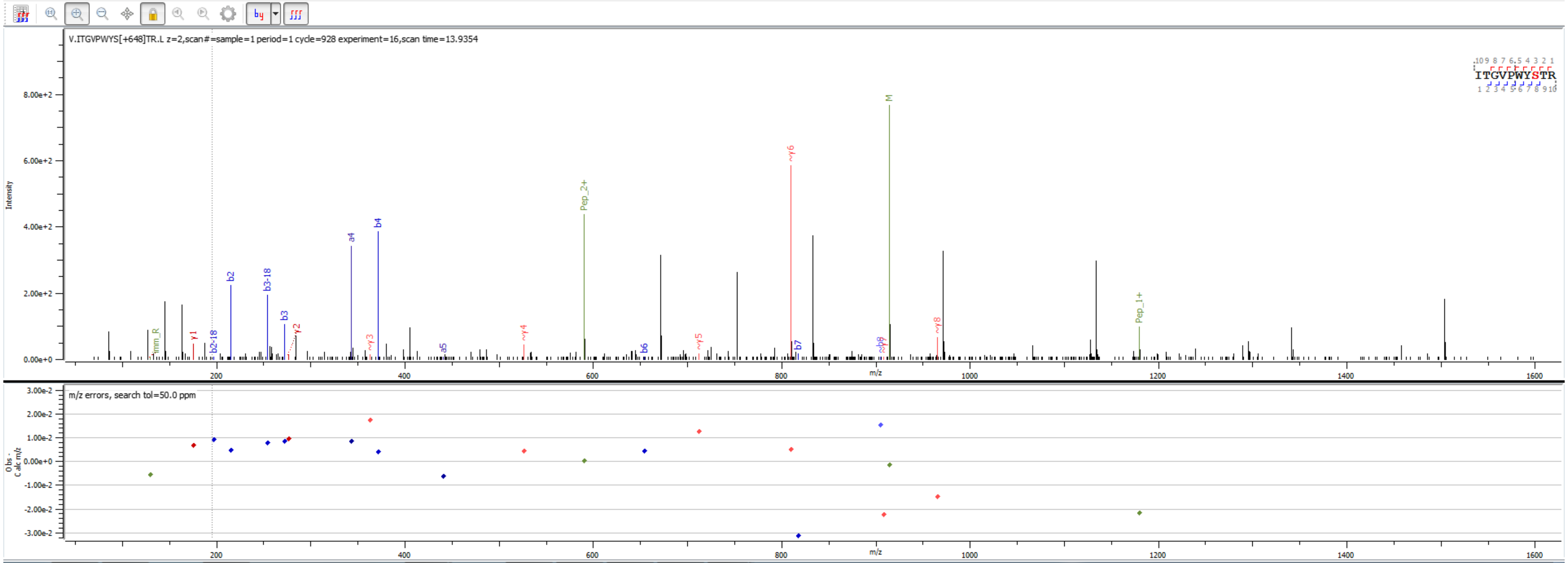

Sample S3 V.ITGVPWYS[+648.211]TR.L

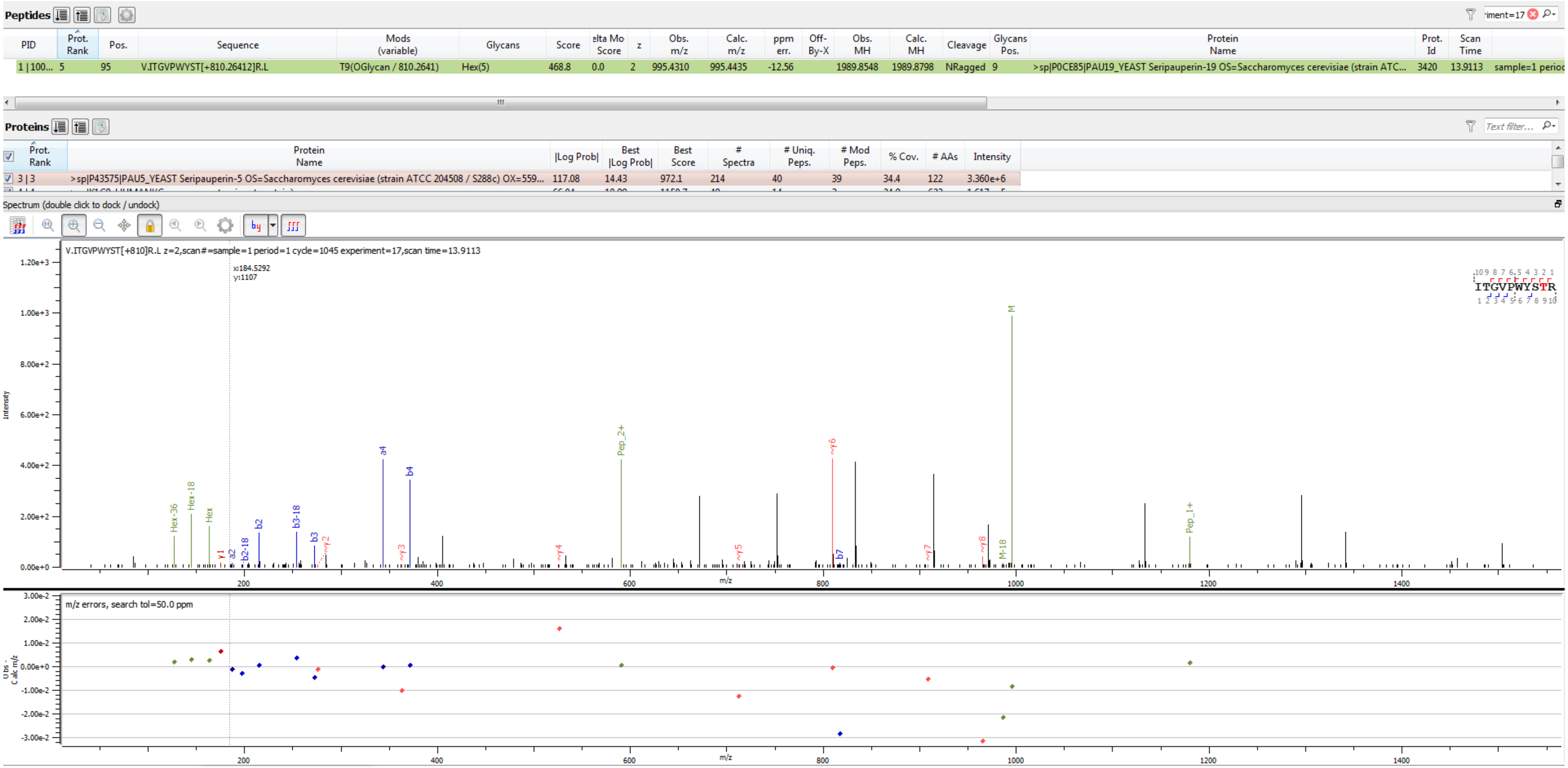

Sample S2 V.ITGVPWYST[+810.264]R.L

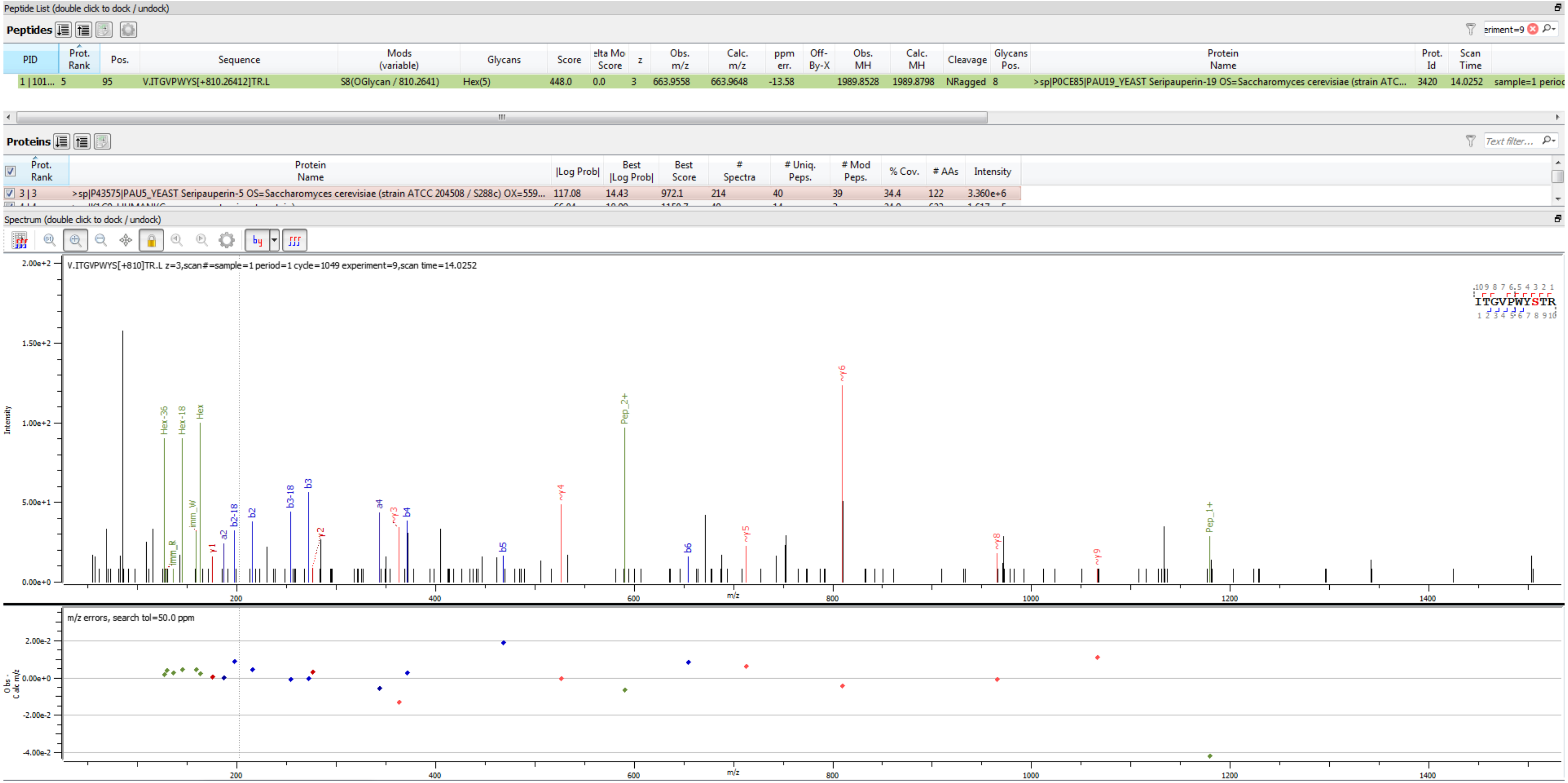

Sample S2 V.ITGVPWYS[+810.264]TR.L

Peptides

PID	Prot. Rank	Pos.	Sequence	Mods (variable)	Glycans	Score	elta Mo Score	z	Obs. m/z	Calc. m/z	ppm err.	Off-By-X	Obs. MH	Calc. MH	Cleavage	Glycans Pos.	Protein Name	Prot. Id	Scan Time	
1 | 126...	5	95	V.ITGVPWYS[+972.31694]TR.L	S8(OGlycan / 972.3169)	Hex(6)	445.2	1.2	2	1076.4524	1076.4699	-16.26		2151.8976	2151.9326	NRagged	8	>sp|P0CE85|PAU19_YEAST Seripauperin-19 OS=Saccharomyces cerevisiae (strain ATC...	3420	13.7051	sample=1 perioc

Proteins

Prot. Rank	Protein Name	|Log Prob|	Best |Log Prob|	Best Score	# Spectra	# Uniq. Peps.	# Mod Peps.	% Cov.	# AAs	Intensity
3 | 3	>sp|P43575|PAU5_YEAST Seripauperin-5 OS=Saccharomyces cerevisiae (strain ATCC 204508 / S288c) OX=559...	117.08	14.43	972.1	214	40	39	34.4	122	3.360e+6

Spectrum (double click to dock / undock)

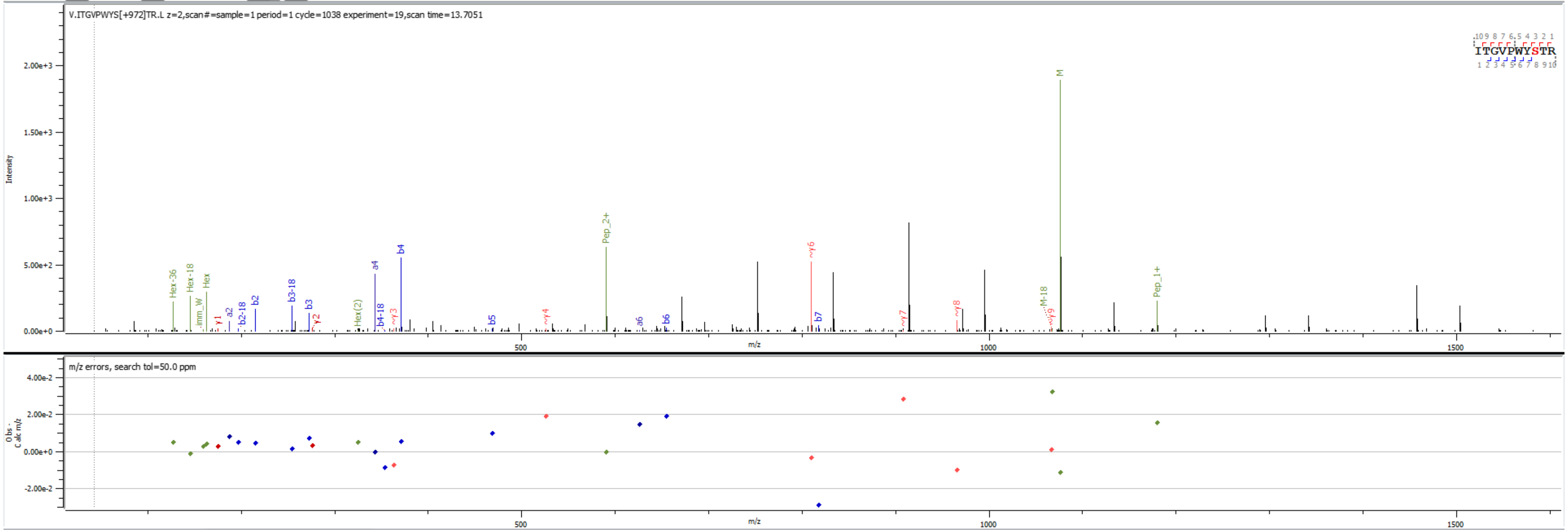

Sample S2 V.ITGVPWYS[+972.317]TR.L

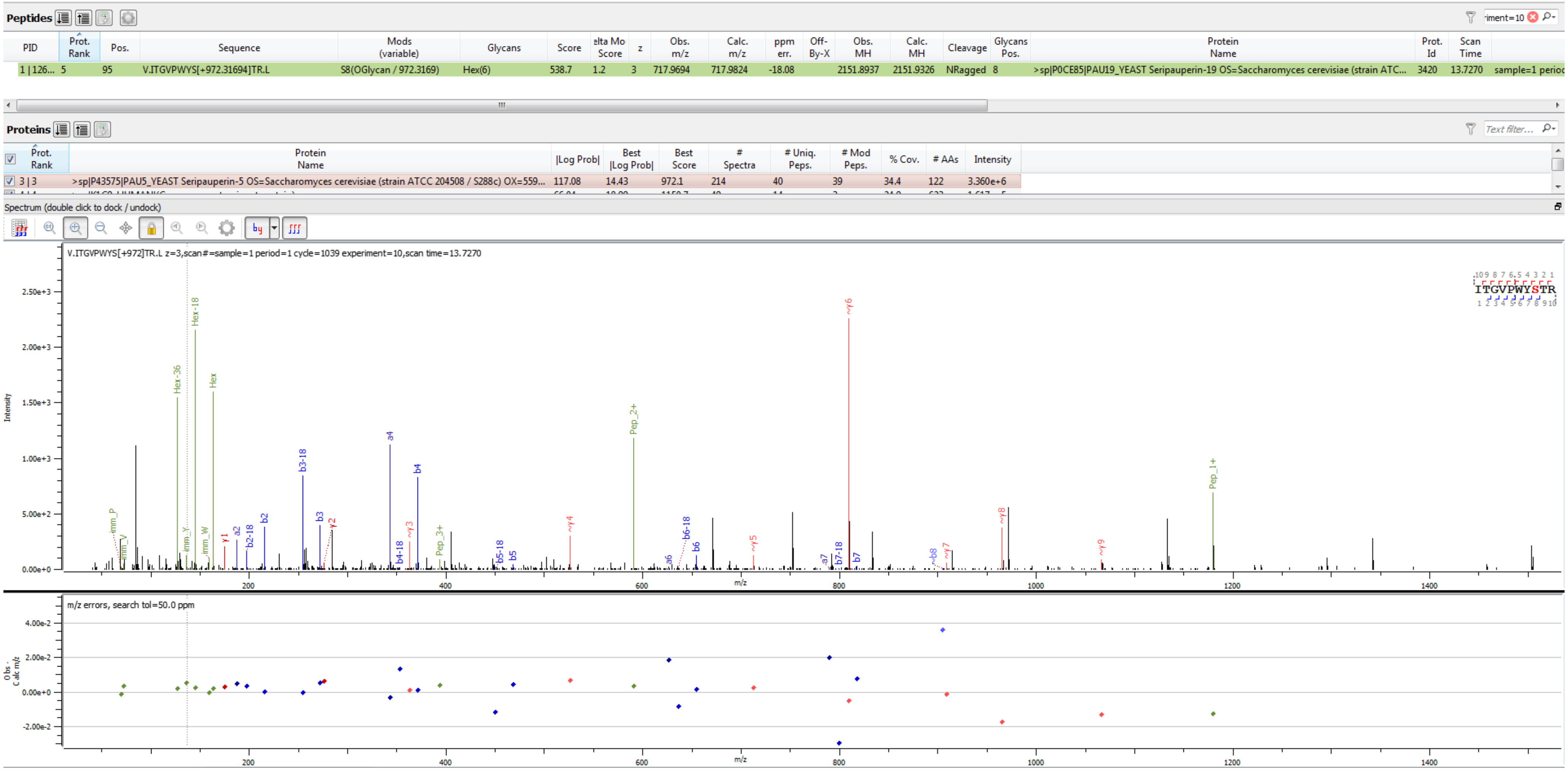

Sample S2 V.ITGVPWYS[+972.317]TR.L

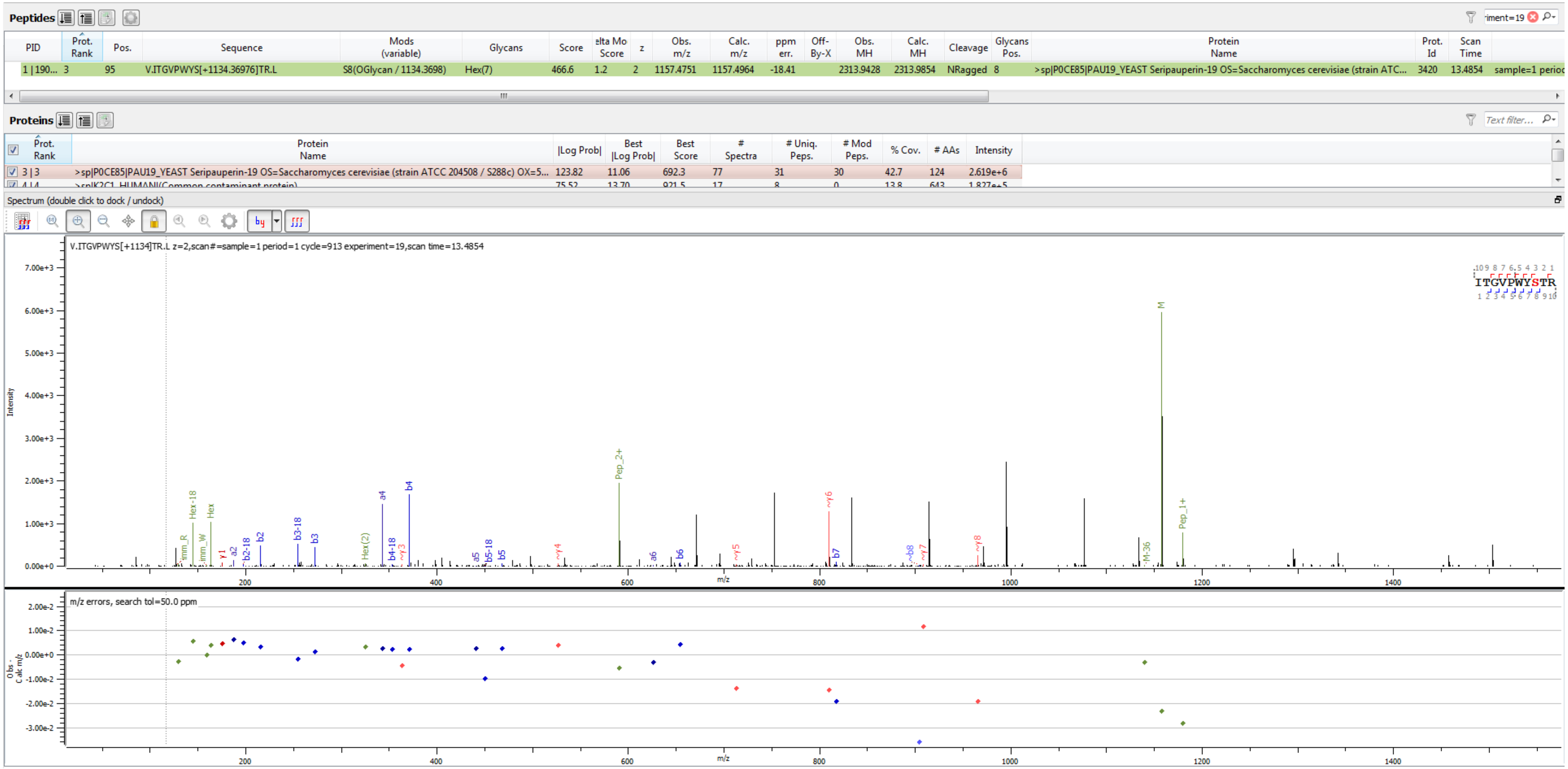

Sample S3 V.ITGVPWYS[+1134.370]TR.L

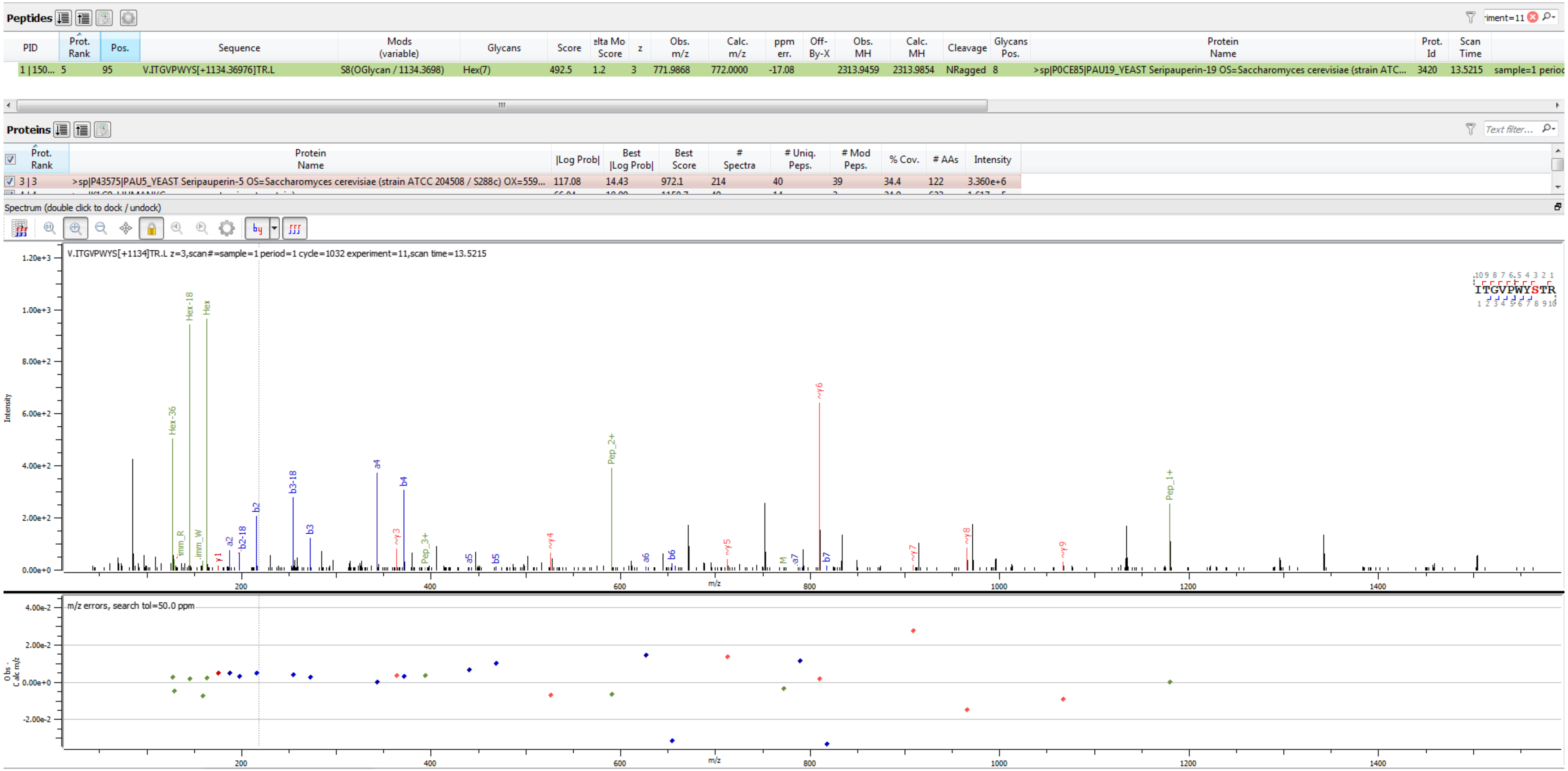

Sample S2 V.ITGVPWYS[+1134.370]TR.L

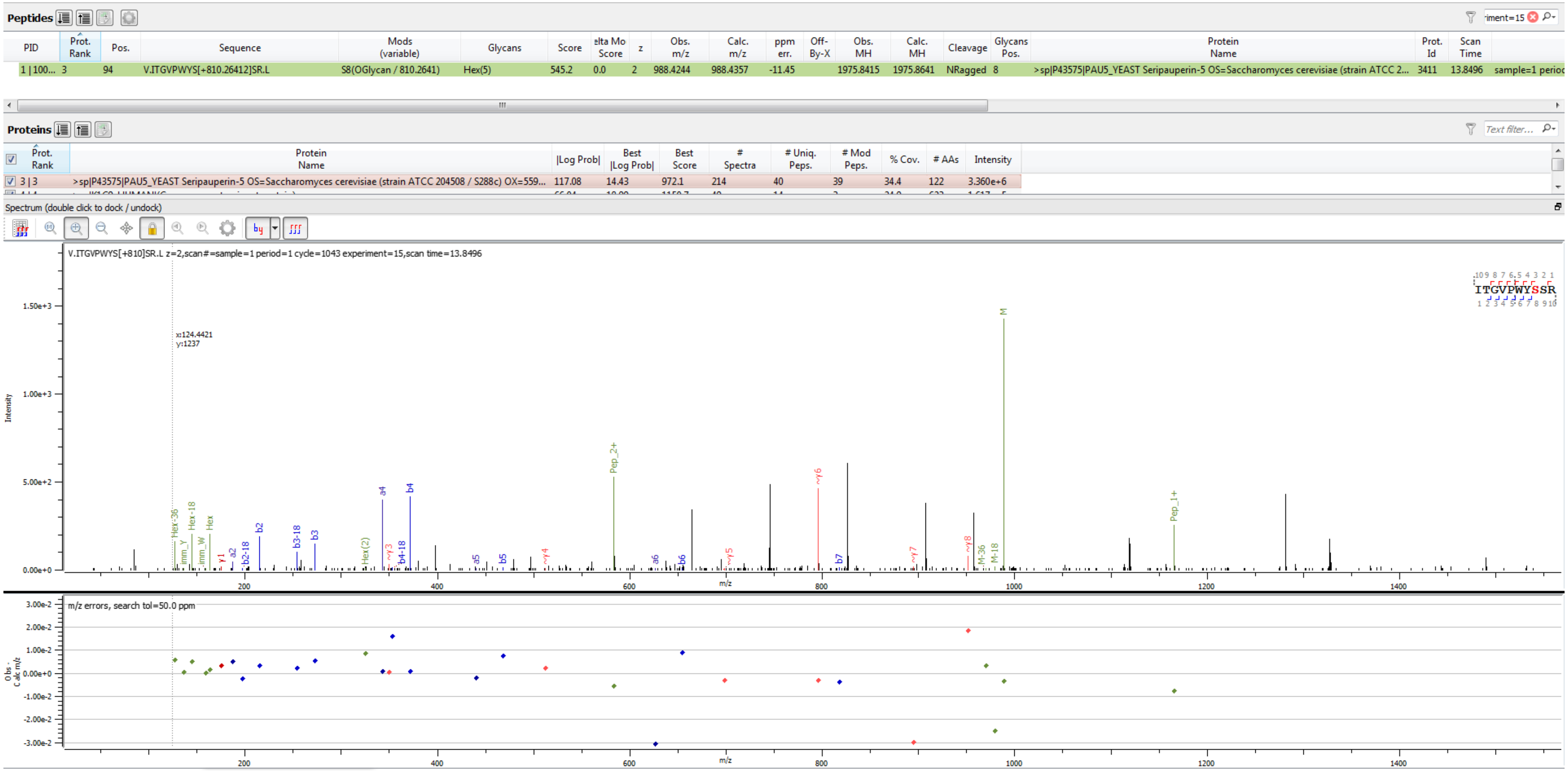

Sample S2 V.ITGVPWYS[+810.264]SR.L

Peptides

PID	Prot. Rank	Pos.	Sequence	Mods (variable)	Glycans	Score	elta Mo Score	z	Obs. m/z	Calc. m/z	ppm err.	Off-By-X	Obs. MH	Calc. MH	Cleavage	Glycans Pos.	Protein Name	Prot. Id	Scan Time	
1 | 834...	1	94	V.ITGVPWYSS[+810.26412]R.L	S9(OGlycan / 810.2641)	Hex(5)	580.7	0.0	3	659.2827	659.2929	-15.52		1975.8335	1975.8641	NRagged	9	>sp|P43575|PAU5_YEAST Seripauperin-5 OS=Saccharomyces cerevisiae (strain ATCC 2...	3411	13.8578	sample=1 perioc

Proteins

Prot. Rank	Protein Name	|Log Prob|	Best |Log Prob|	Best Score	# Spectra	# Uniq. Peps.	# Mod Peps.	% Cov.	# AAs	Intensity
1 | 1	>sp|P43575|PAU5_YEAST Seripauperin-5 OS=Saccharomyces cerevisiae (strain ATCC 204508 / S288c) OX=559...	136.57	14.25	968.4	163	30	27	28.7	122	1.440e+6

Spectrum (double click to dock / undock)

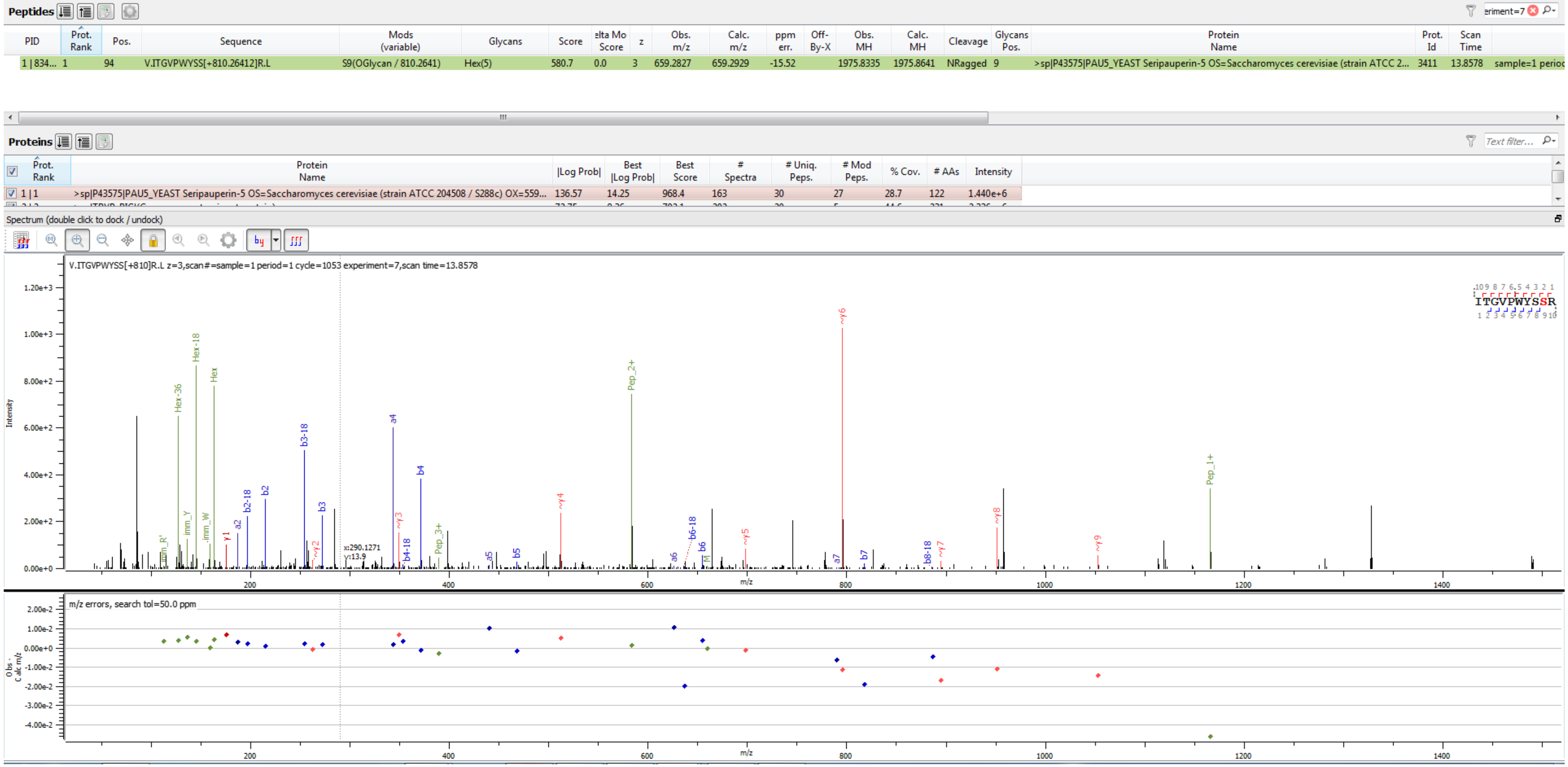

Sample S5 V.ITGVPWYSS[+810.264]R.L

Peptides

PID	Prot. Rank	Pos.	Sequence	Mods (variable)	Glycans	Score	elta Mo Score	z	Obs. m/z	Calc. m/z	ppm err.	Off-By-X	Obs. MH	Calc. MH	Cleavage	Glycans Pos.	Protein Name	Prot. Id	Scan Time	
1 | 126...	3	94	V.ITGVPWYS[+972.31694]SR.L	S8(OGlycan / 972.3169)	Hex(6)	481.7	1.2	2	1069.4505	1069.4621	-10.91		2137.8936	2137.9170	NRagged	8	>sp|P43575|PAU5_YEAST Seripauperin-5 OS=Saccharomyces cerevisiae (strain ATCC 2...	3411	13.5588	sample=1 perioc

Proteins

Prot. Rank	Protein Name	|Log Prob|	Best |Log Prob|	Best Score	# Spectra	# Uniq. Peps.	# Mod Peps.	% Cov.	# AAs	Intensity
3 | 3	>sp|P43575|PAU5_YEAST Seripauperin-5 OS=Saccharomyces cerevisiae (strain ATCC 204508 / S288c) OX=559...	117.08	14.43	972.1	214	40	39	34.4	122	3.360e+6

Spectrum (double click to dock / undock)

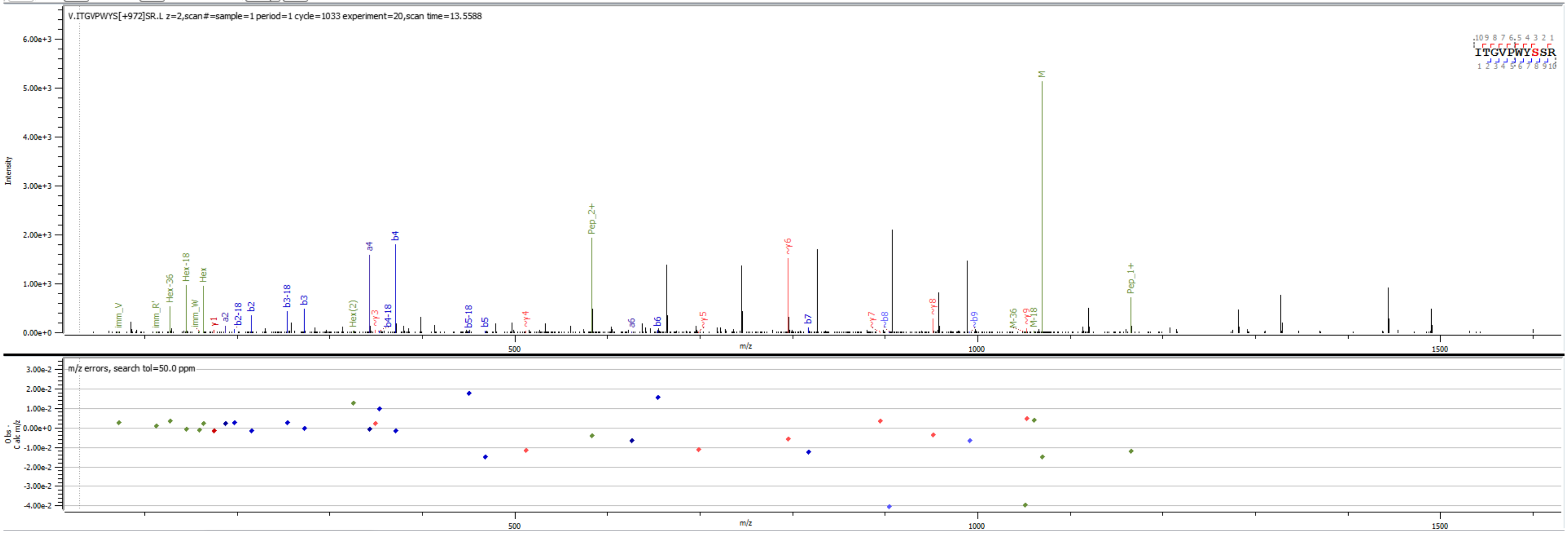

Sample S2 V.ITGVPWYS[+972.317]SR.L

Peptides

PID	Prot. Rank	Pos.	Sequence	Mods (variable)	Glycans	Score	elta Mo Score	z	Obs. m/z	Calc. m/z	ppm err.	Off-By-X	Obs. MH	Calc. MH	Cleavage	Glycans Pos.	Protein Name	Prot. Id	Scan Time	
1 | 125...	3	94	V.ITGVPWYS[+972.31694]SR.L	S8(OGlycan / 972.3169)	Hex(6)	532.3	1.2	3	713.3003	713.3105	-14.33		2137.8863	2137.9170	NRagged	8	>sp|P43575|PAU5_YEAST Seripauperin-5 OS=Saccharomyces cerevisiae (strain ATCC 2...	3411	13.5199	sample=1 perioc

Proteins

Prot. Rank	Protein Name	|Log Prob|	Best |Log Prob|	Best Score	# Spectra	# Uniq. Peps.	# Mod Peps.	% Cov.	# AAs	Intensity
3 | 3	>sp|P43575|PAU5_YEAST Seripauperin-5 OS=Saccharomyces cerevisiae (strain ATCC 204508 / S288c) OX=559...	117.08	14.43	972.1	214	40	39	34.4	122	3.360e+6
[illegible]	[illegible]	[illegible]	[illegible]	[illegible]	[illegible]	[illegible]	[illegible]	[illegible]	[illegible]	[illegible]

Spectrum (double click to dock / undock)

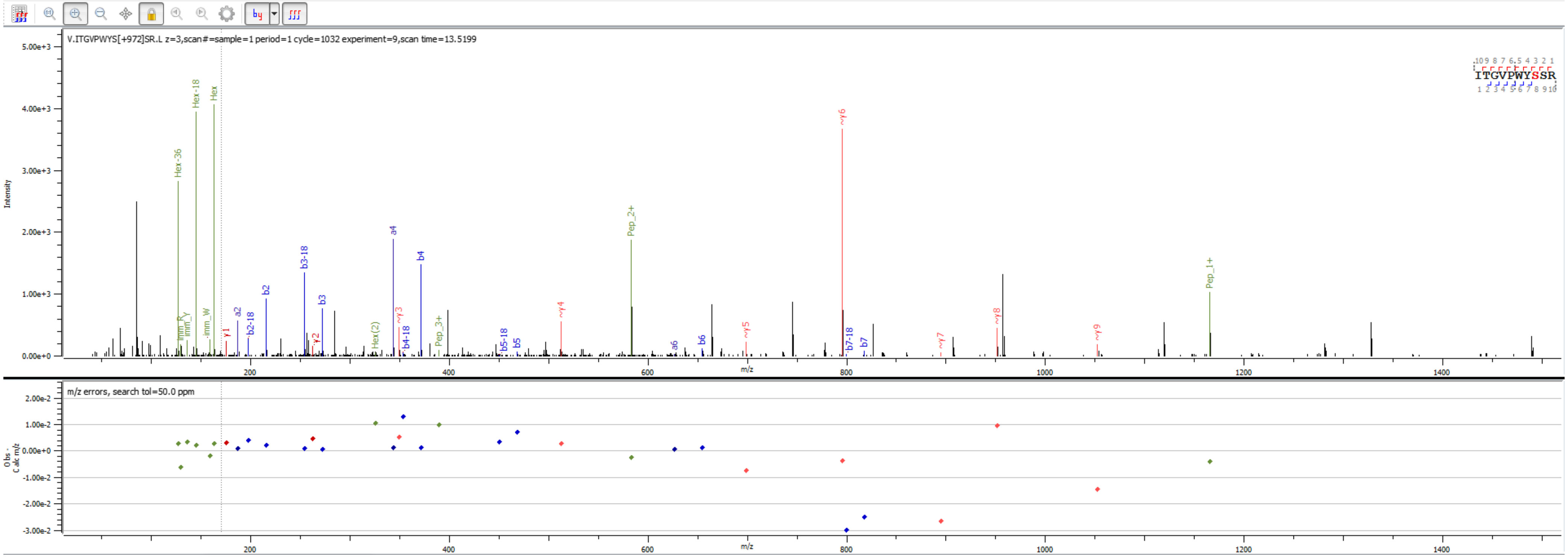

Sample S2 V.ITGVPWYS[+972.317]SR.L

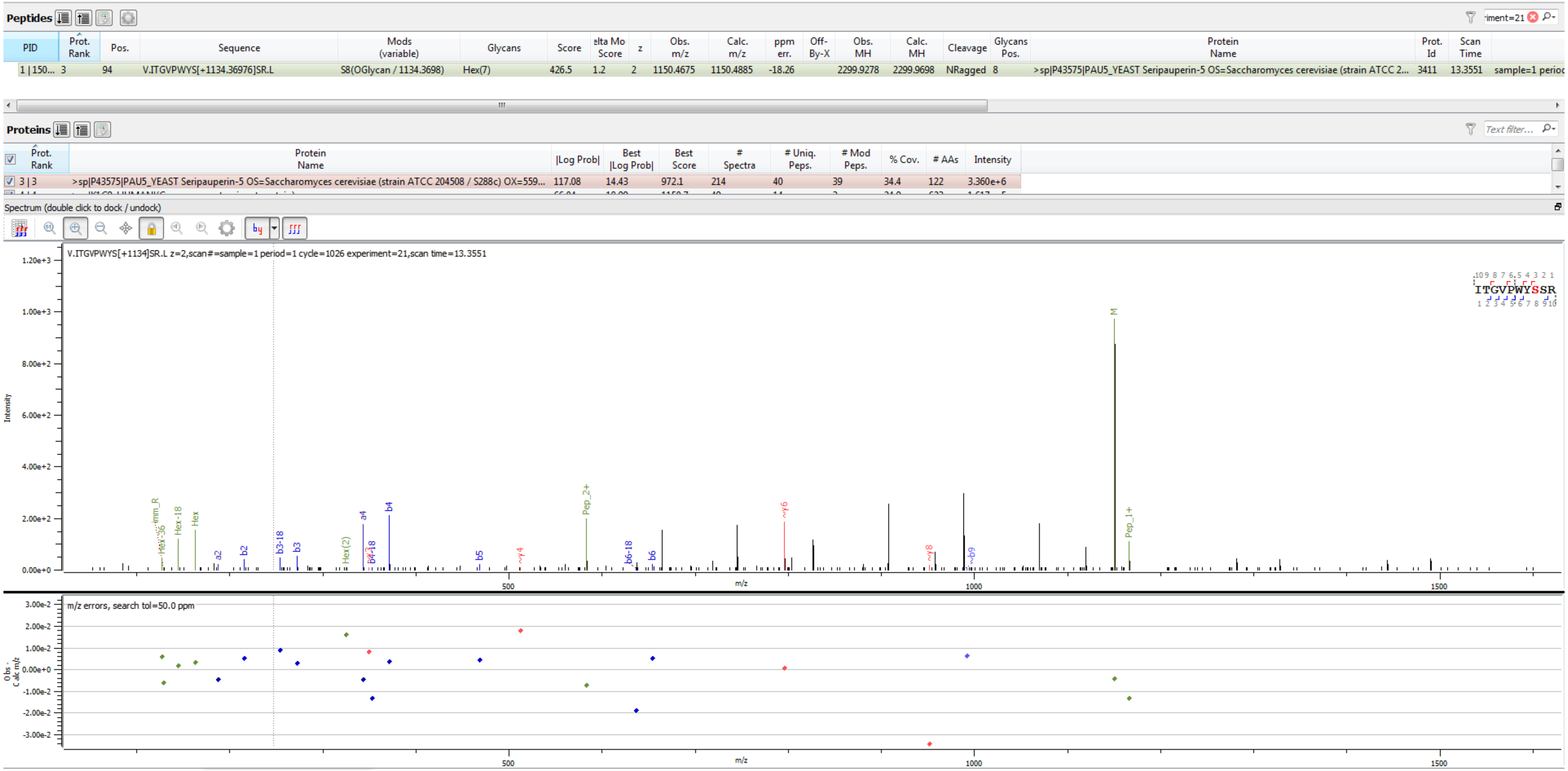

Sample S2 V.ITGVPWYS[+1134.370]SR.L

Peptides

PID	Prot. Rank	Pos.	Sequence	Mods (variable)	Glycans	Score	elta Mo Score	z	Obs. m/z	Calc. m/z	ppm err.	Off-By-X	Obs. MH	Calc. MH	Cleavage	Glycans Pos.	Protein Name	Prot. Id	Scan Time	
1 | 473...	1	47	R.AHLAEYYS[+324.10565]FQ.A	S8(OGlycan / 324.1056)	Hex(2)	573.4	326.7	2	776.8299	776.8381	-10.56		1552.6526	1552.6689	CRagged	8	>sp|P43575|PAU5_YEAST Seripauperin-5 OS=Saccharomyces cerevisiae (strain ATCC 2...	3411	14.0075	sample=1 perioc

Proteins

Prot. Rank	Protein Name	|Log Prob|	Best |Log Prob|	Best Score	# Spectra	# Uniq. Peps.	# Mod Peps.	% Cov.	# AAs	Intensity
1 | 1	>sp|P43575|PAU5_YEAST Seripauperin-5 OS=Saccharomyces cerevisiae (strain ATCC 204508 / S288c) OX=559...	327.47	16.74	1004.1	632	70	67	68.9	122	3.742e+7

Spectrum (double click to dock / undock)

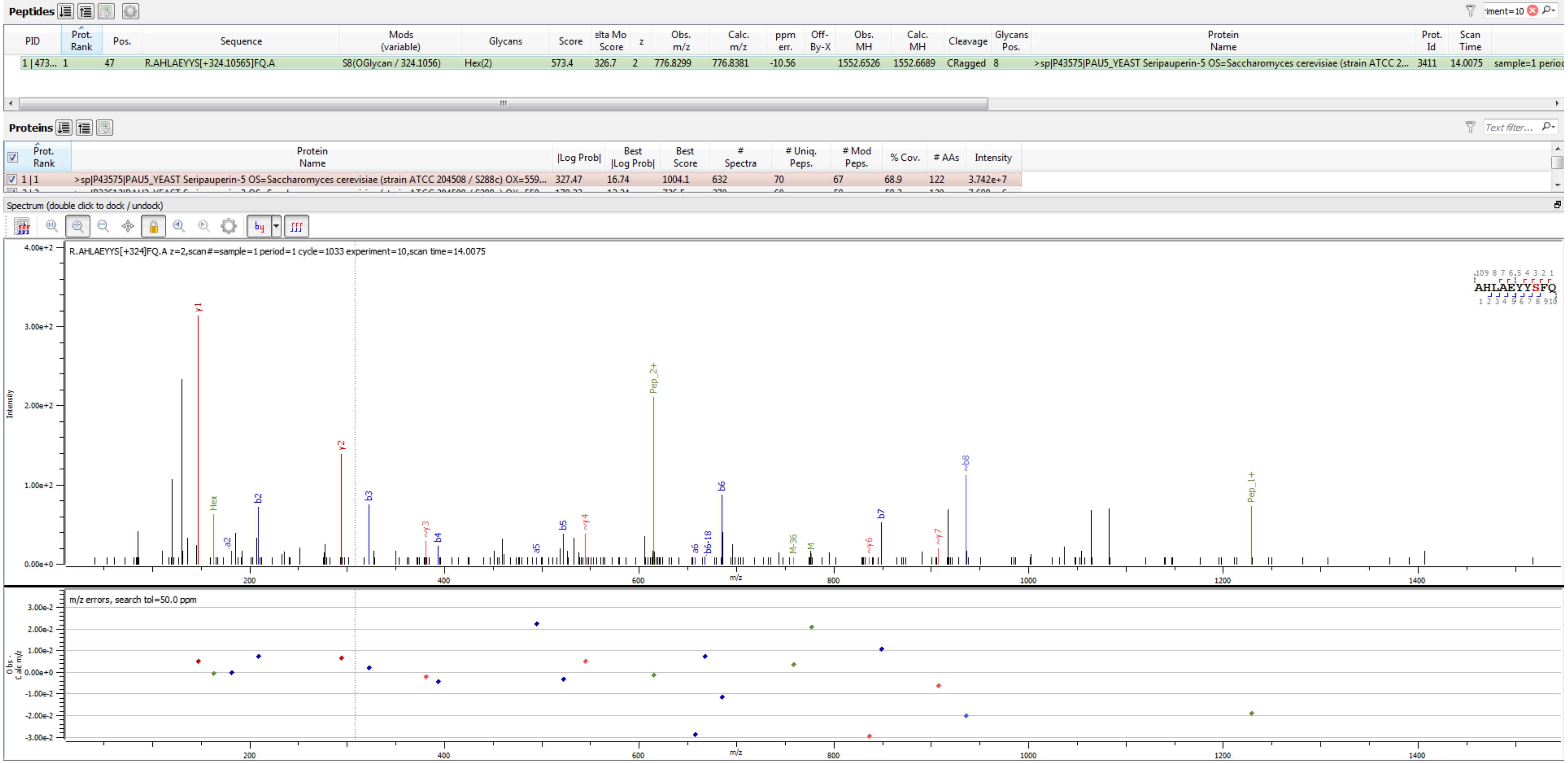

Sample S1 R.AHLAEYYS[+324.106]FQ.A

Peptides

PID	Prot. Rank	Pos.	Sequence	Mods (variable)	Glycans	Score	elta Mo Score	z	Obs. m/z	Calc. m/z	ppm err.	Off-By-X	Obs. MH	Calc. MH	Cleavage	Glycans Pos.	Protein Name	Prot. Id	Scan Time	
1 | 953...	2	105	R.LRPAISSALS[+648.21129]K.D	S10(OGlycan / 648.2113)	Hex(4)	559.8	0.0	3	597.6273	597.6383	-18.53		1790.8673	1790.9005	Specific	10	>sp|P0CE85|PAU19_YEAST Seripauperin-19 OS=Saccharomyces cerevisiae (strain ATC...	3420	10.5948	sample=1 perioc

Proteins

	Prot. Rank	Protein Name	|Log Prob|	Best |Log Prob|	Best Score	# Spectra	# Uniq. Peps.	# Mod Peps.	% Cov.	# AAs	Intensity
☑	2 | 2	>sp|P0CE85|PAU19_YEAST Seripauperin-19 OS=Saccharomyces cerevisiae (strain ATCC 204508 / S288c) OX=5...	138.61	11.51	683.8	93	32	31	42.7	124	3.148e+6
☑	3 | 3	>sp|P43575|PAU5_YEAST Seripauperin-5 OS=Saccharomyces cerevisiae (strain ATCC 204508 / S288c) OX=559	129.60	12.22	721.6	114	28	27	53.3	122	7.378e+6

Spectrum (double click to dock / undock)

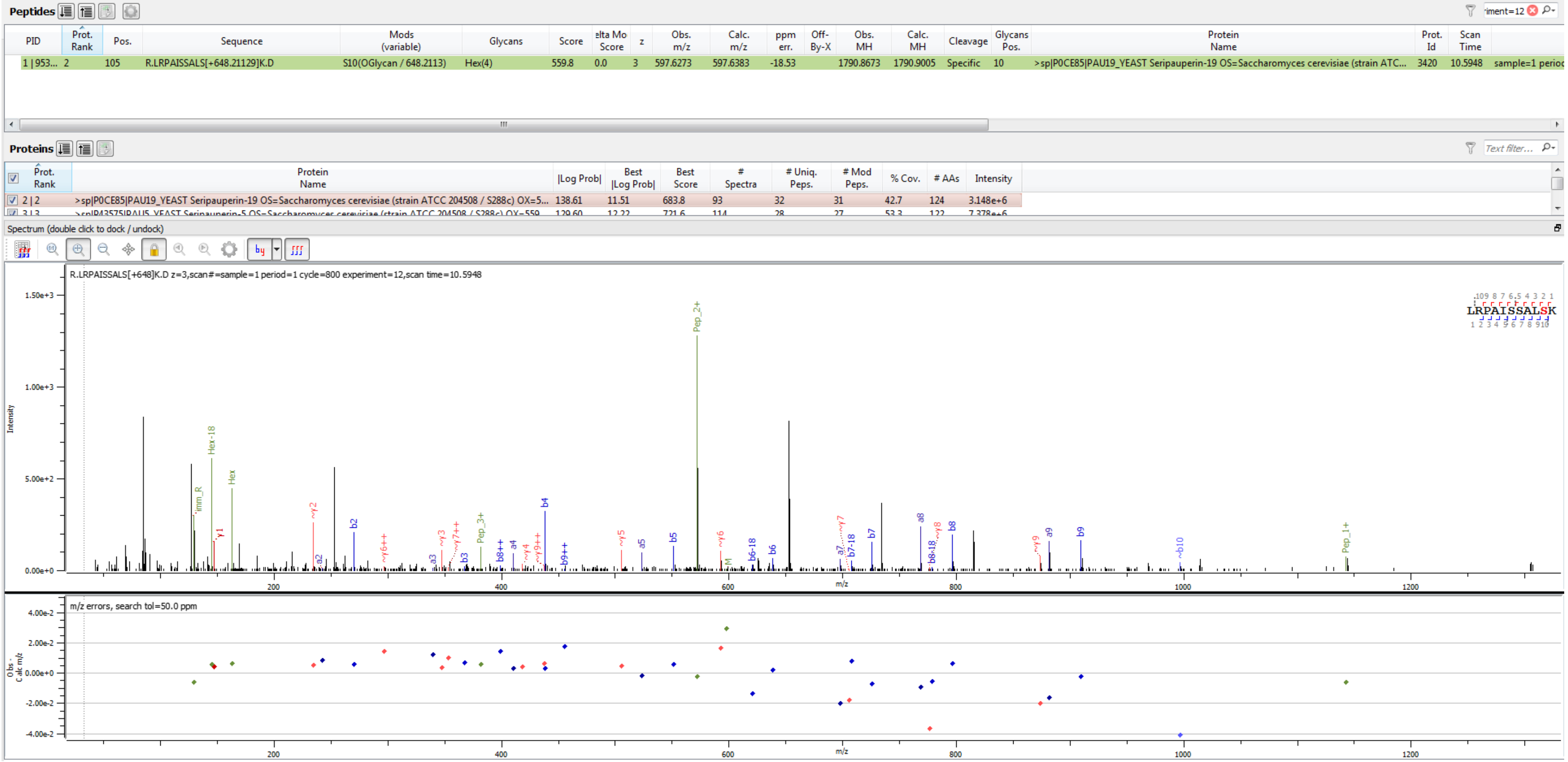

Sample S4 R.LRPAISSALS[+648.211]K.D

Peptides

PID	Prot. Rank	Pos.	Sequence	Mods (variable)	Glycans	Score	elta Mo Score	z	Obs. m/z	Calc. m/z	ppm err.	Off-By-X	Obs. MH	Calc. MH	Cleavage	Glycans Pos.	Protein Name	Prot. Id	Scan Time	
1 | 805...	2	105	R.LRPAISSALS[+810.26412]K.D	S10(OGlycan / 810.2641)	Hex(5)	554.9	0.0	3	651.6456	651.6559	-15.94		1952.9222	1952.9533	Specific	10	>sp|Q07987|PAU23_YEAST Seripauperin-23 OS=Saccharomyces cerevisiae (strain ATC...	3438	10.3538	sample=1 perioc

Proteins

Prot. Rank	Protein Name	|Log Prob|	Best |Log Prob|	Best Score	# Spectra	# Uniq. Peps.	# Mod Peps.	% Cov.	# AAs	Intensity
1 | 1	>sp|Q3E770|PAU9_YEAST Seripauperin-9 OS=Saccharomyces cerevisiae (strain ATCC 204508 / S288c) OX=559...	288.60	16.66	988.6	452	61	56	60.8	120	1.023e+7
2 | 2	>sp|Q07987|PAU23_YEAST Seripauperin-23 OS=Saccharomyces cerevisiae (strain ATCC 204508 / S288c) OX=5...	160.38	13.28	718.6	124	39	36	43.5	124	2.323e+6
3 | 3	>sp|P43575|PAU5_YEAST Seripauperin-5 OS=Saccharomyces cerevisiae (strain ATCC 204508 / S288c) OX=559...	142.55	14.15	737.1	167	35	33	42.6	122	6.136e+6

Spectrum (double click to dock / undock)

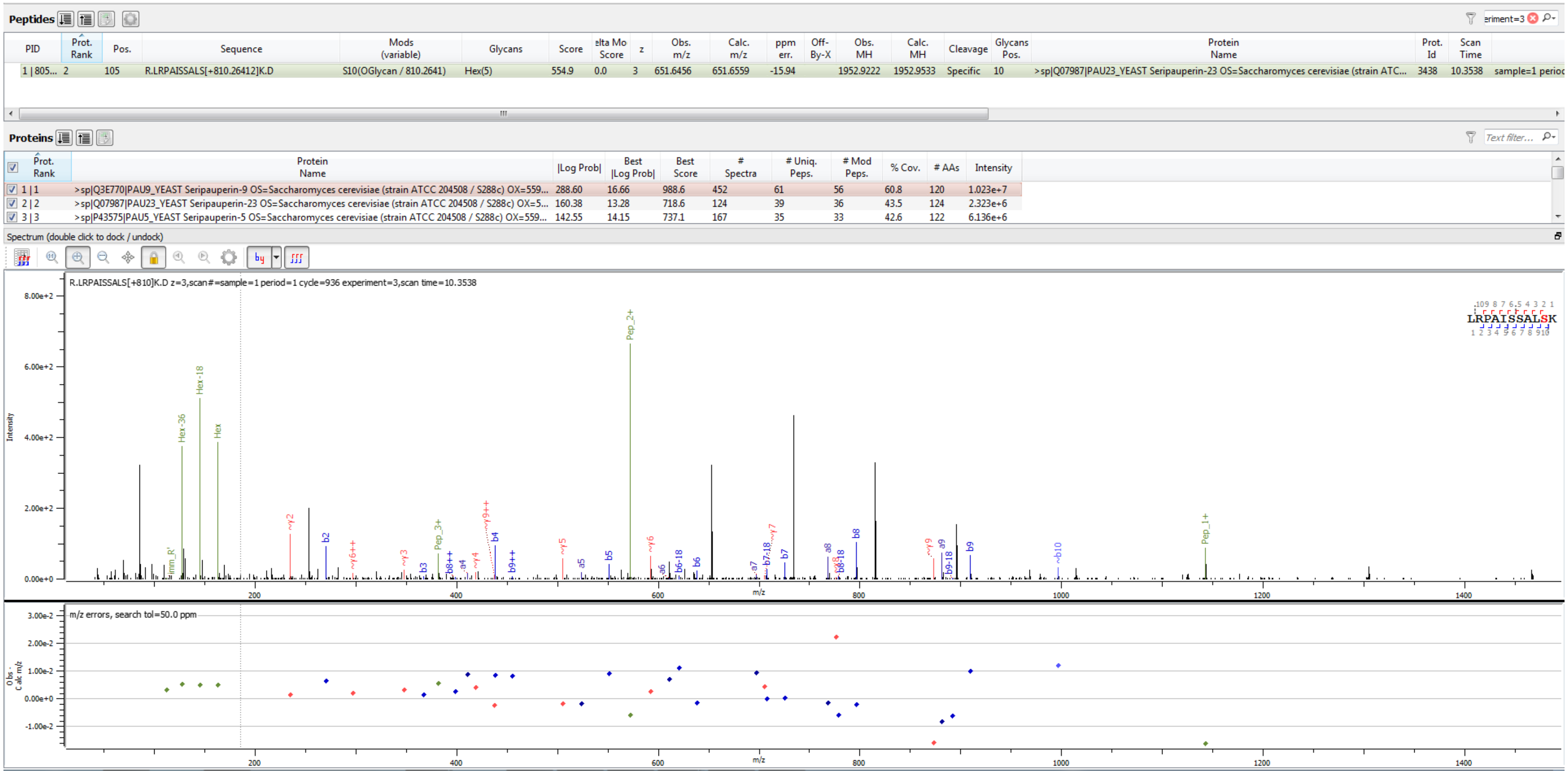

Sample S6 R.LRPAISSALS[+810.264]K.D

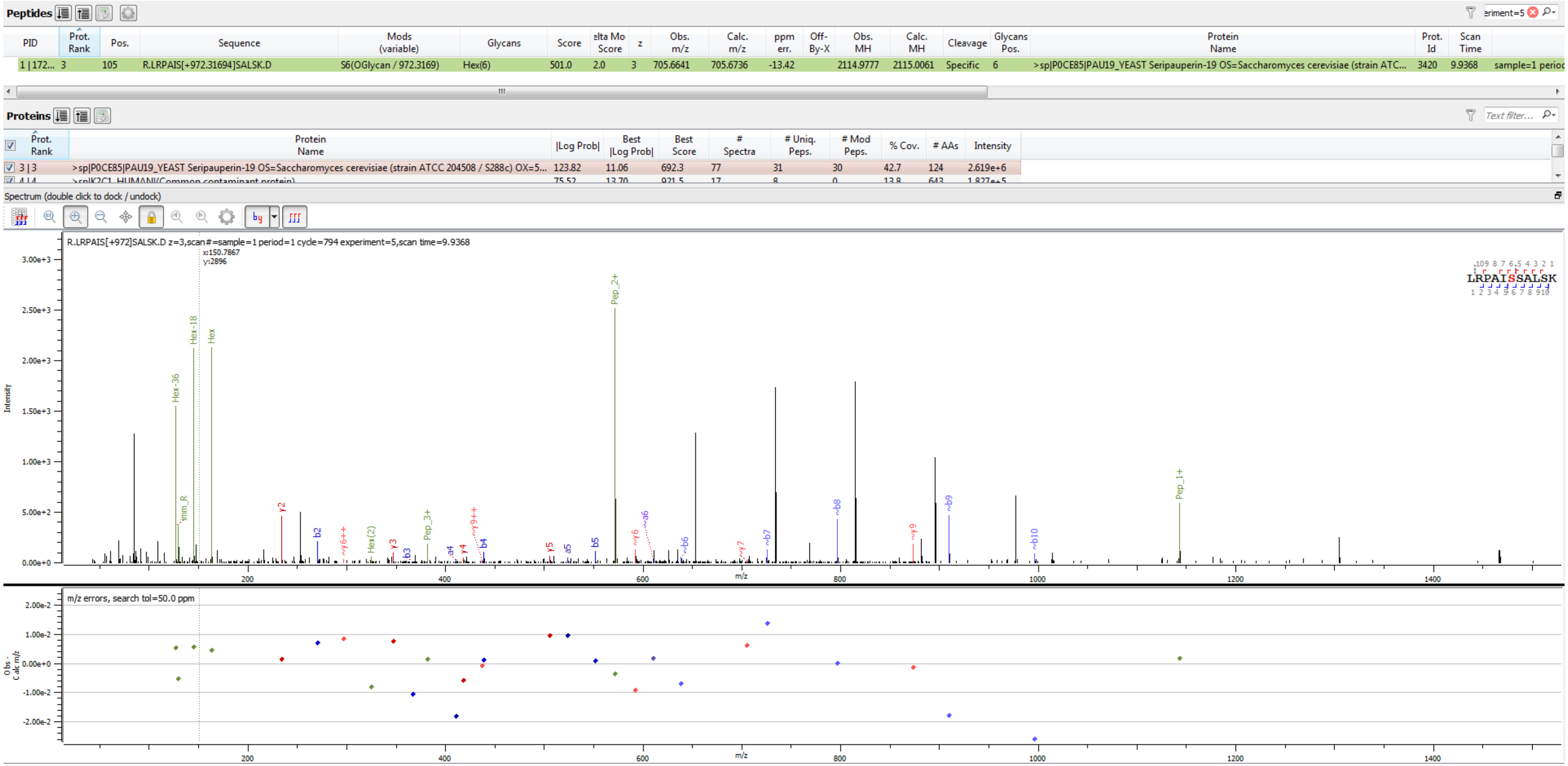

Sample S3 R.LRPAIS[+972.317]SALSK.D

Peptides

PID	Prot. Rank	Pos.	Sequence	Mods (variable)	Glycans	Score	elta Mo Score	z	Obs. m/z	Calc. m/z	ppm err.	Off-By-X	Obs. MH	Calc. MH	Cleavage	Glycans Pos.	Protein Name	Prot. Id	Scan Time	
1 | 163...	4	105	R.LRPAISS[+1134.36976]ALSK.D	S7(OGlycan / 1134.3698)	Hex(7)	393.5	3.6	3	759.6760	759.6912	-19.95		2277.0135	2277.0589	Specific	7	>sp|Q07987|PAU23_YEAST Seripauperin-23 OS=Saccharomyces cerevisiae (strain ATC...	3438	9.9396	sample=1 perioc

Proteins

Prot. Rank	Protein Name	|Log Prob|	Best |Log Prob|	Best Score	# Spectra	# Uniq. Peps.	# Mod Peps.	% Cov.	# AAs	Intensity
1 | 1	>sp|P43575|PAU5_YEAST Seripauperin-5 OS=Saccharomyces cerevisiae (strain ATCC 204508 / S288c) OX=559...	327.47	16.74	1004.1	632	70	67	68.9	122	3.742e+7

Spectrum (double click to dock / undock)

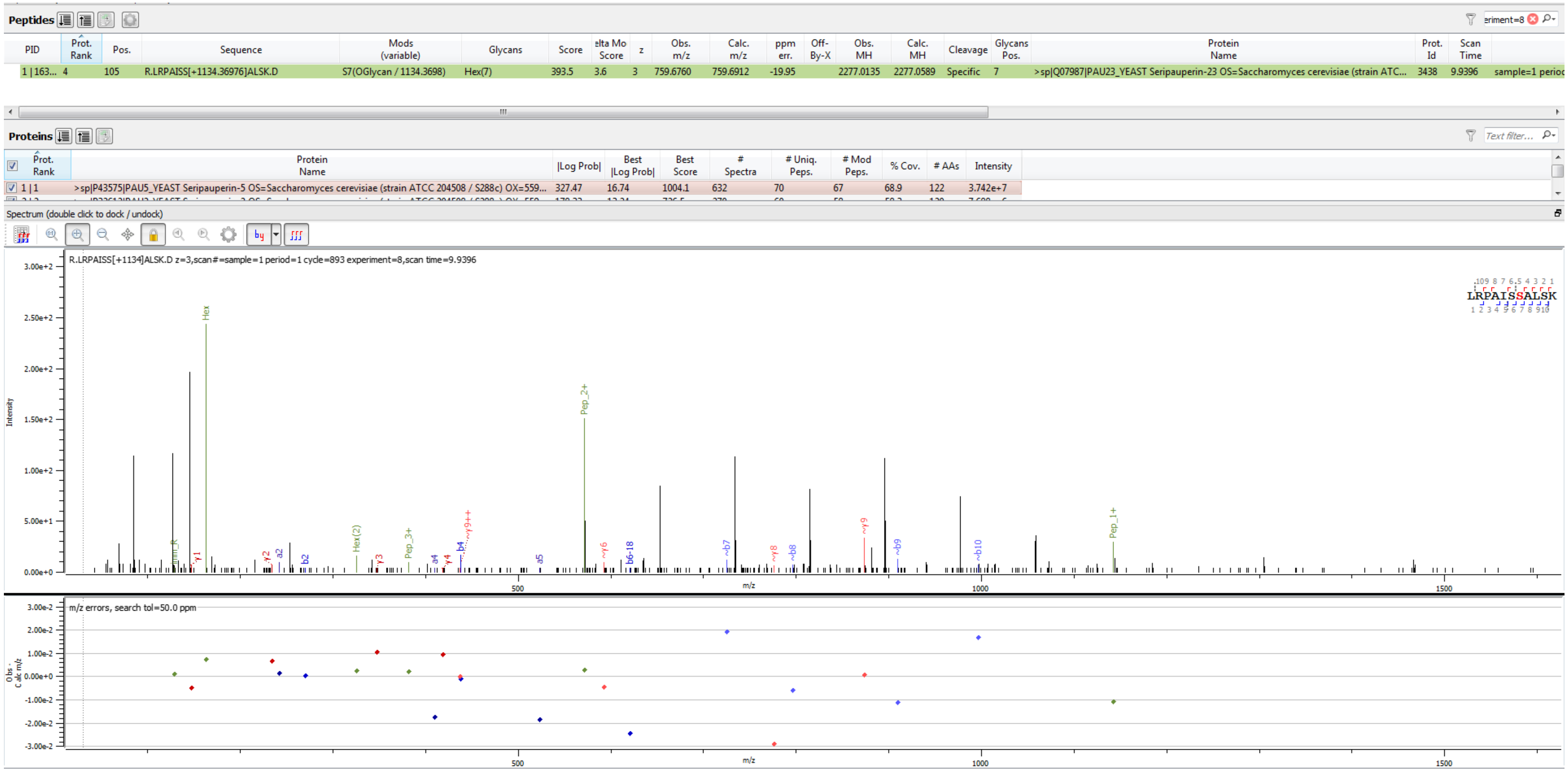

Sample S1 R.LRPAISS[+1134.370]ALSK.D

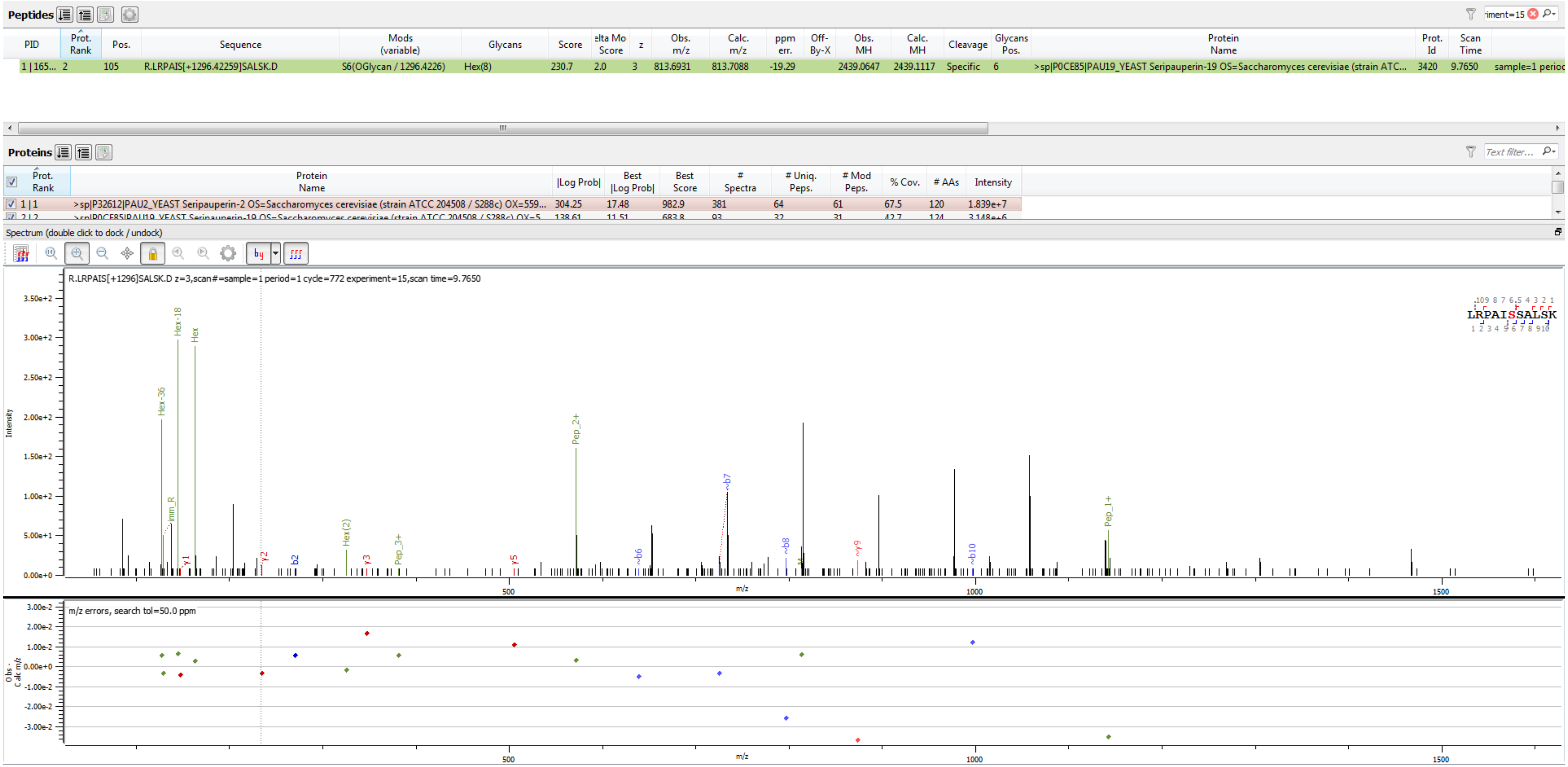

Sample S4 R.LRPAIS[+1296.423]SALSK.D

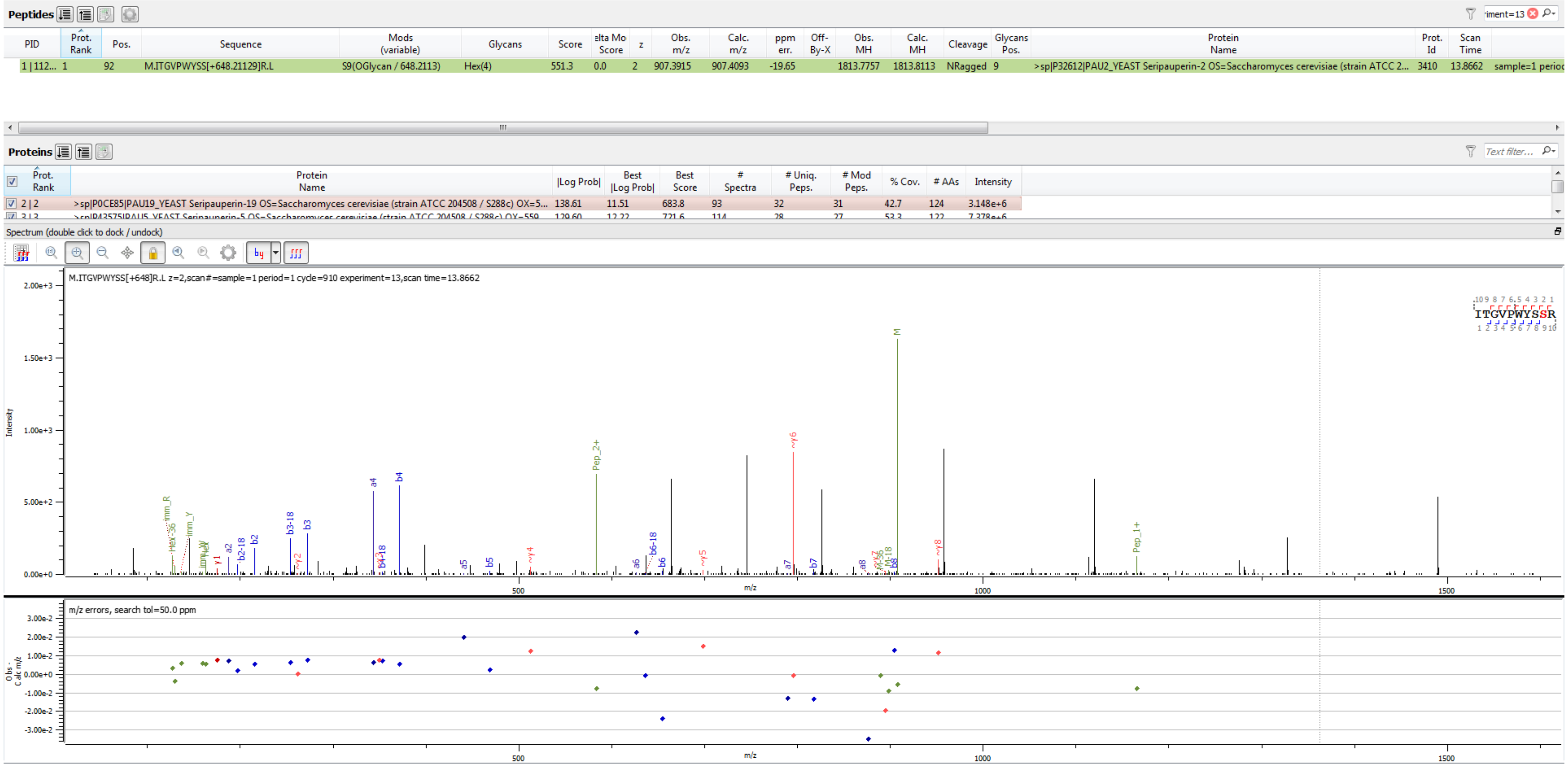

Sample S4 M.ITGVPWYSS[+648.211]R.L

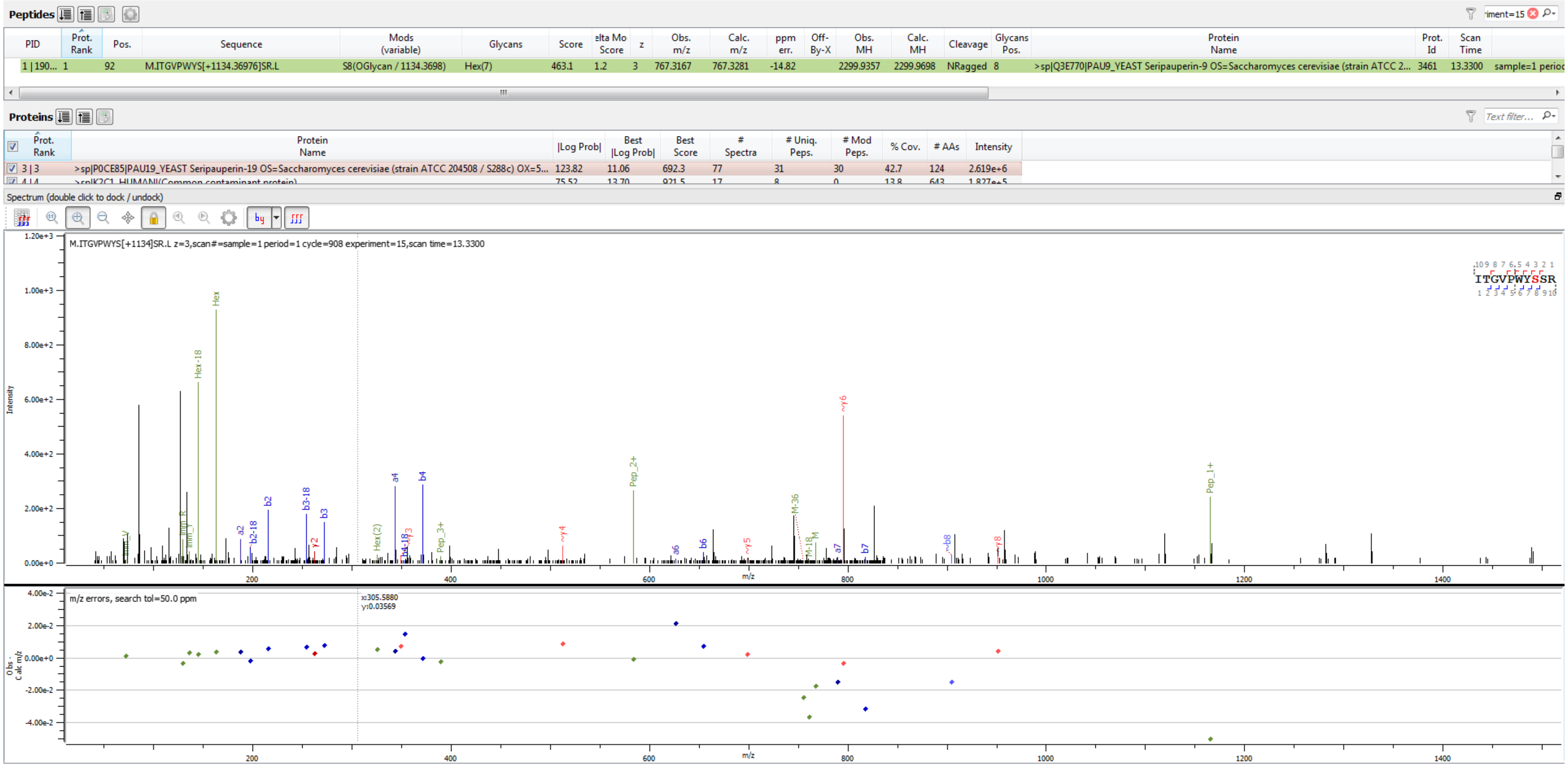

Sample S3 M.ITGVPWYS[+1134.370]SR.L

Peptides

'iment=17

PID	Prot. Rank	Pos.	Sequence	Mods (variable)	Glycans	Score	elta Mo Score	z	Obs. m/z	Calc. m/z	ppm err.	Off-By-X	Obs. MH	Calc. MH	Cleavage	Glycans Pos.	Protein Name	Prot. Id	Scan Time	
1 | 180...	1	92	M.ITGVPWYS[+1296.42259]SR.L	S8(OGlycan / 1296.4226)	Hex(8)	474.5	1.2	3	821.3375	821.3457	-10.00		2461.9980	2462.0226	NRagged	8	>sp|P32612|PAU2_YEAST Seripauperin-2 OS=Saccharomyces cerevisiae (strain ATCC 2...	3410	13.1470	sample=1 perioc

Proteins

Text filter...

☑ Prot. Rank	Protein Name	|Log Prob|	Best |Log Prob|	Best Score	# Spectra	# Uniq. Peps.	# Mod Peps.	% Cov.	# AAs	Intensity
☑ 1 | 1	>sp|P32612|PAU2_YEAST Seripauperin-2 OS=Saccharomyces cerevisiae (strain ATCC 204508 / S288c) OX=559...	304.25	17.48	982.9	381	64	61	67.5	120	1.839e+7
☑ 2 | 2	>sp|P0CE85|PAU19_YEAST Seripauperin-19 OS=Saccharomyces cerevisiae (strain ATCC 204508 / S288c) OX=5...	138.61	11.51	683.8	93	32	31	42.7	124	3.148e+6

Spectrum (double click to dock / undock)

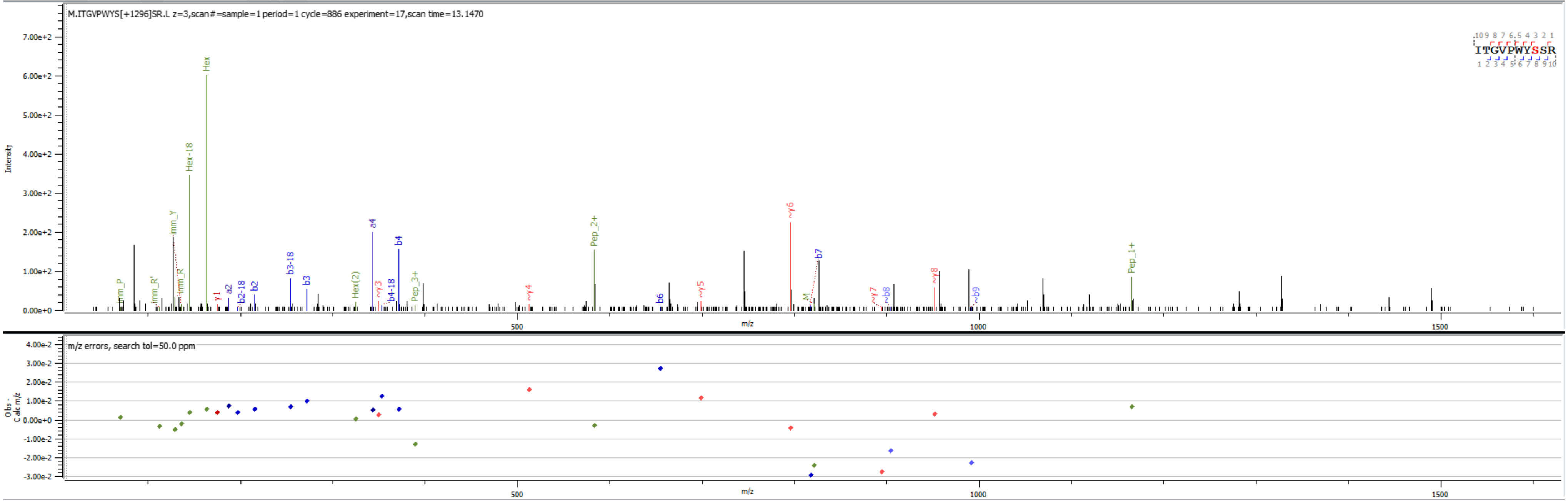

Sample S4 M.ITGVPWYS[+1296.423]SR.L

Peptides

PID	Prot. Rank	Pos.	Sequence	Mods (variable)	Glycans	Score	elta Mo Score	z	Obs. m/z	Calc. m/z	ppm err.	Off-By-X	Obs. MH	Calc. MH	Cleavage	Glycans Pos.	Protein Name	Prot. Id	Scan Time	
1 | 358...	1	104	R.LKPAISS[+324.10565]AL.S	S7(OGlycan / 324.1056)	Hex(2)	496.0	8.1	2	612.3255	612.3345	-14.67		1223.6437	1223.6617	CRagged	7	>sp|P43575|PAU5_YEAST Seripauperin-5 OS=Saccharomyces cerevisiae (strain ATCC 2...	3411	13.0583	sample=1 perioc

Proteins

Prot. Rank	Protein Name	|Log Prob|	Best |Log Prob|	Best Score	# Spectra	# Uniq. Peps.	# Mod Peps.	% Cov.	# AAs	Intensity
1 | 1	>sp|P43575|PAU5_YEAST Seripauperin-5 OS=Saccharomyces cerevisiae (strain ATCC 204508 / S288c) OX=559...	327.47	16.74	1004.1	632	70	67	68.9	122	3.742e+7
[illegible]	[illegible]	[illegible]	[illegible]	[illegible]	[illegible]	[illegible]	[illegible]	[illegible]	[illegible]	[illegible]

Spectrum (double click to dock / undock)

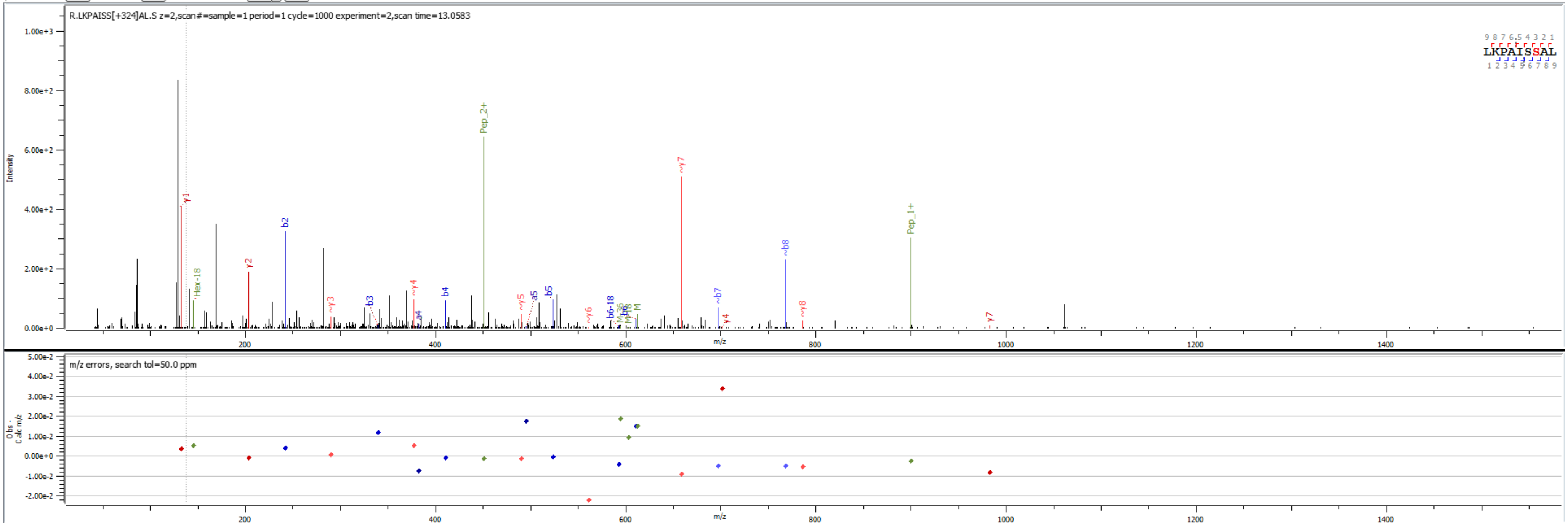

Sample S1 R.LKPAISS[+324.106]AL.S

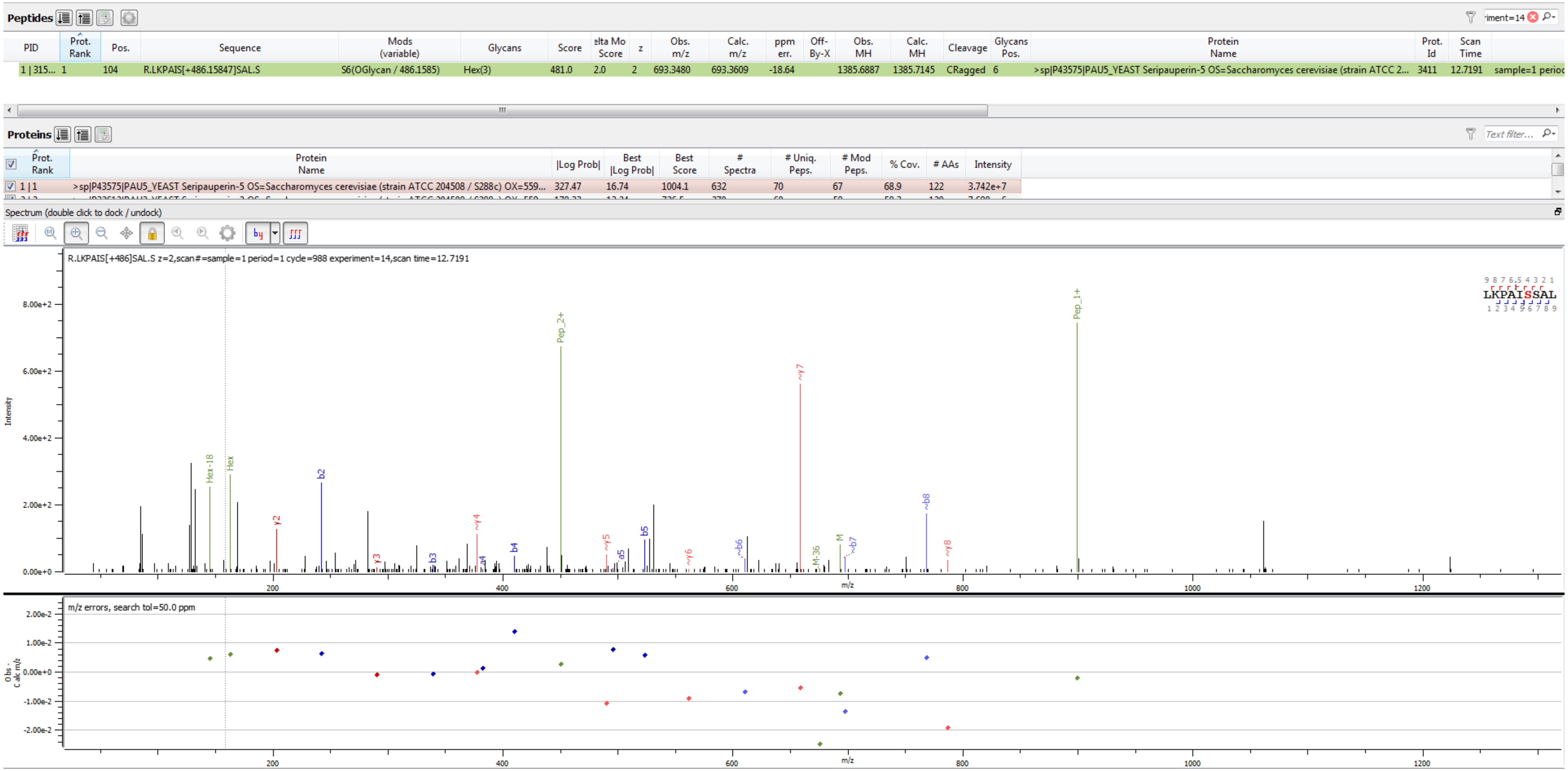

Sample S1 R.LKPAIS[+486.158]SAL.S

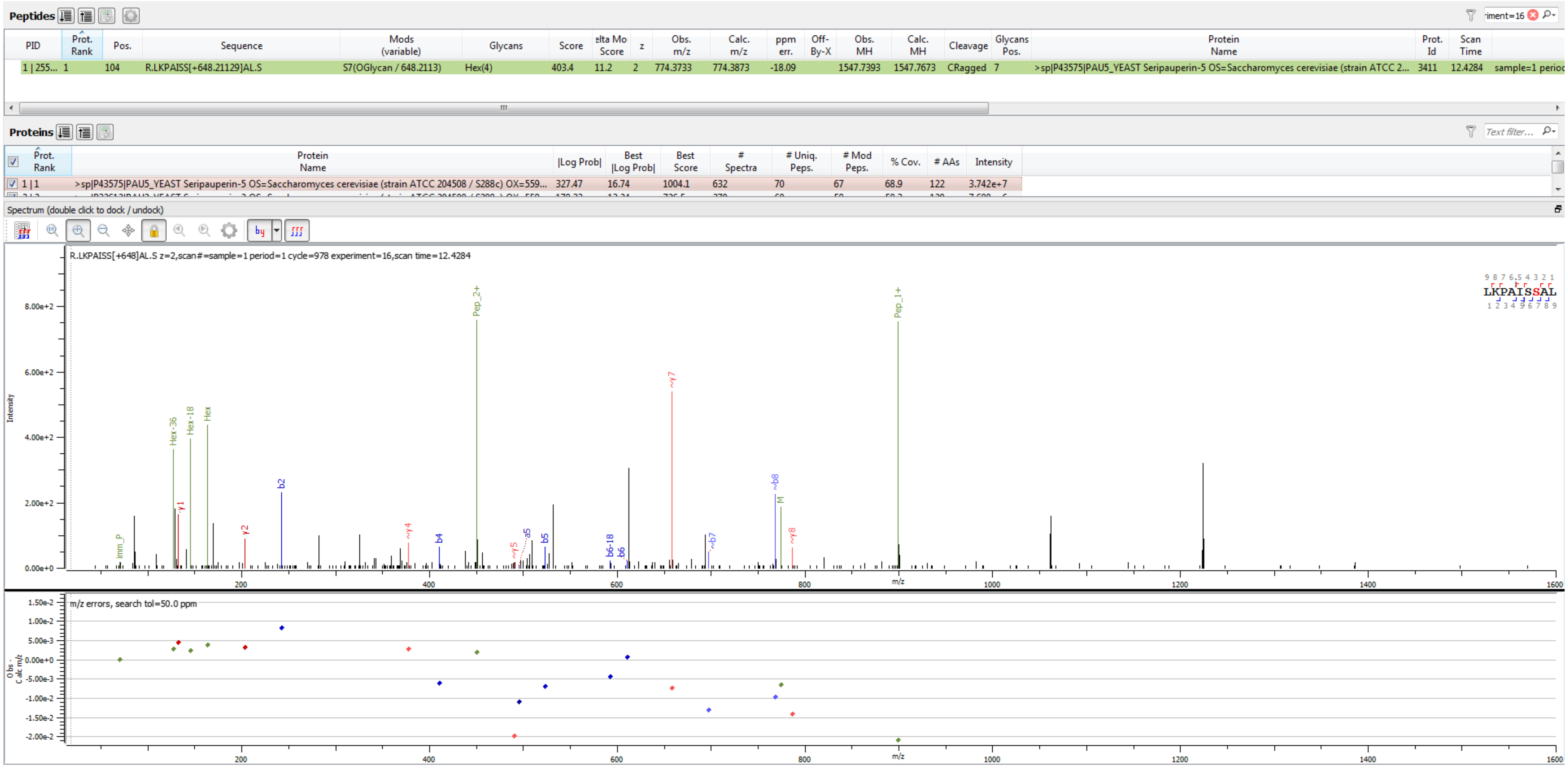

Sample S1 R.LKPAISS[+648.211]AL.S

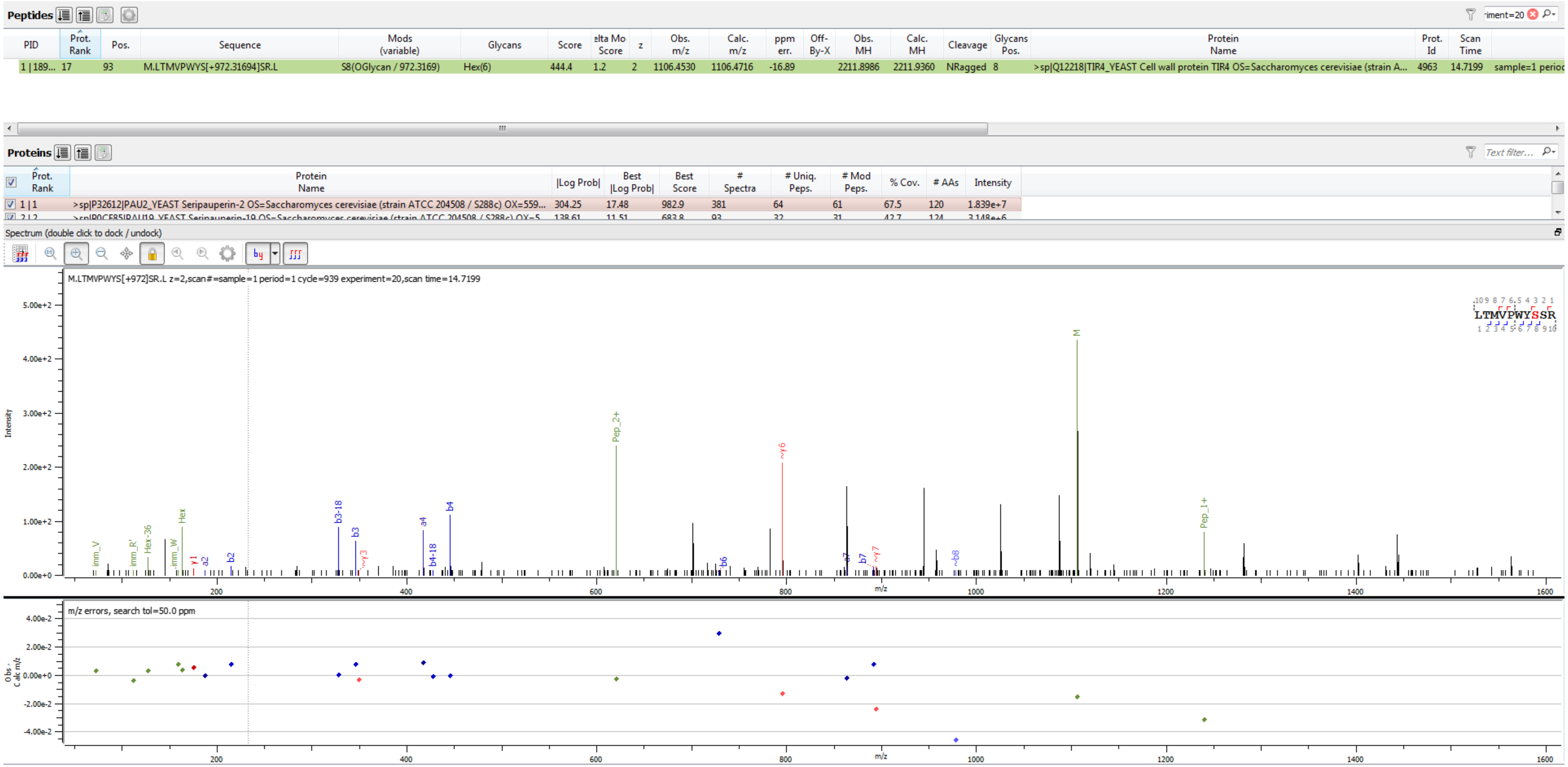

Sample S4 M.LTMVPWYS[+972.317]SR.L

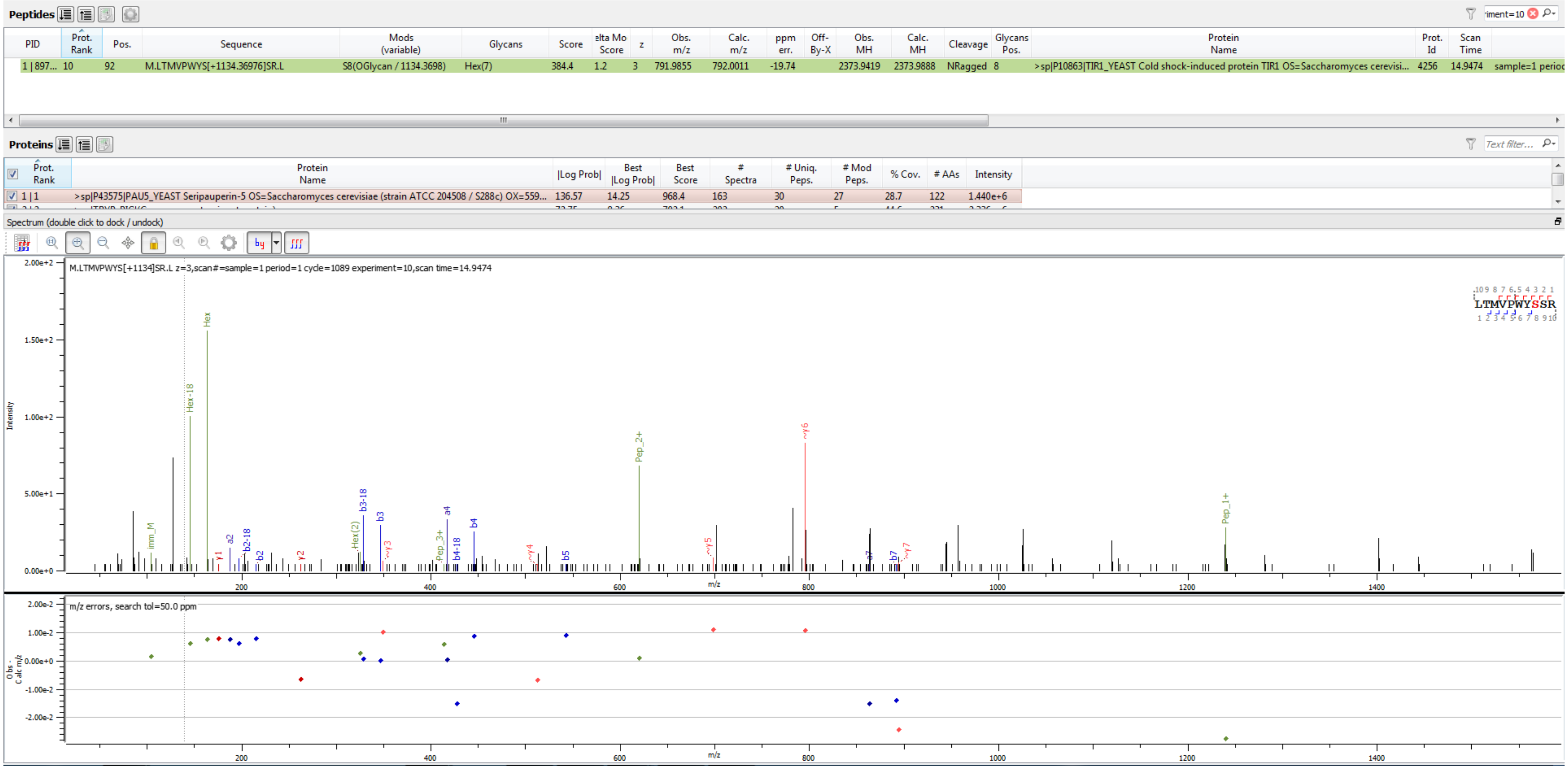

Sample S4 M.LTMVPWYS[+1134.370]SR.L

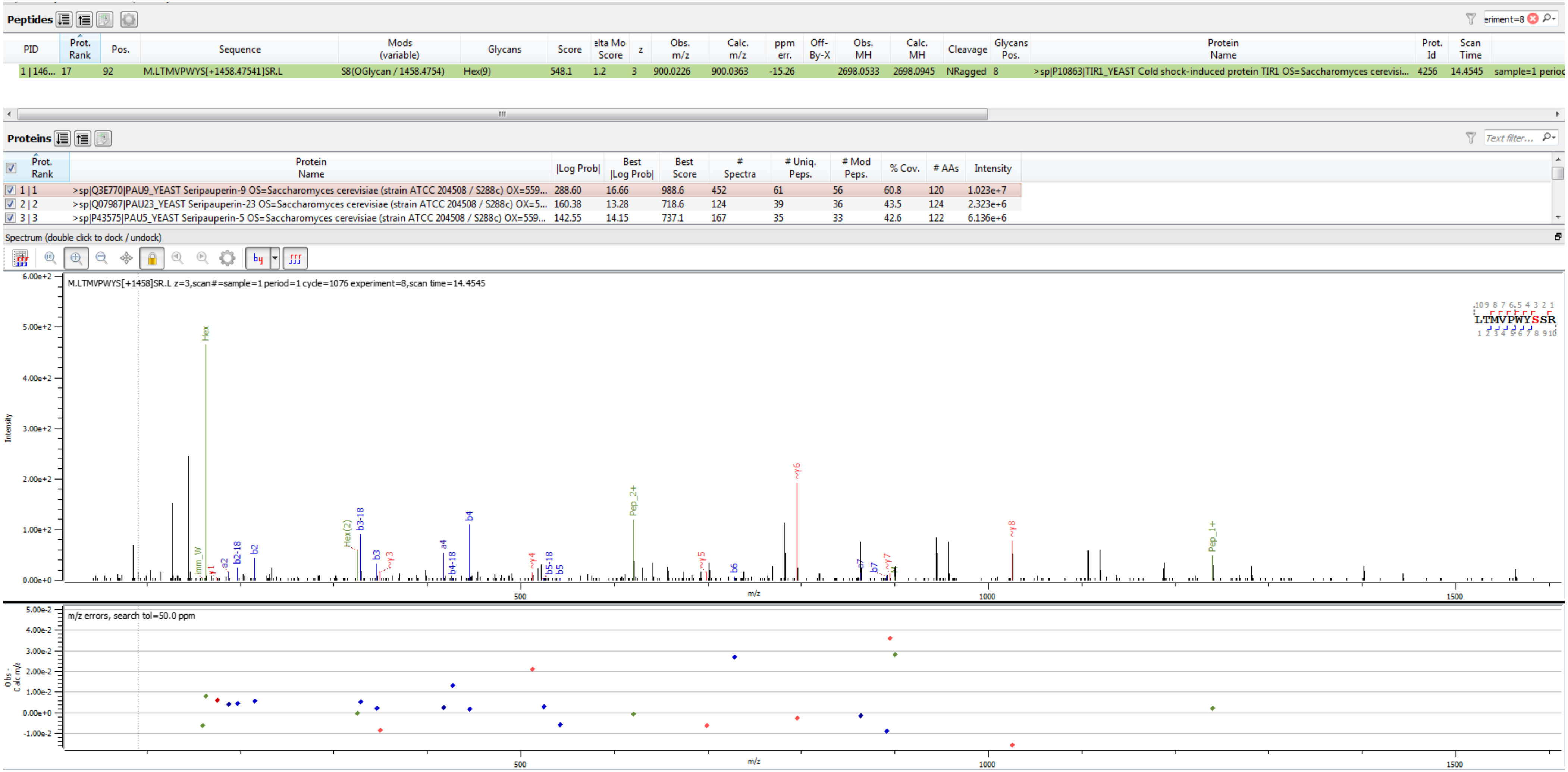

Sample S6 M.LTMVPWYS[+1458.475]SR.L

Peptides

PID	Prot. Rank	Pos.	Sequence	Mods (variable)	Glycans	Score	elta Mo Score	z	Obs. m/z	Calc. m/z	ppm err.	Off-By-X	Obs. MH	Calc. MH	Cleavage	Glycans Pos.	Protein Name	Prot. Id	Scan Time	
1 | 262...	3	105	R.LRPAISS[+486.15847]AL.S	S7(OGlycan / 486.1585)	Hex(3)	481.0	17.7	2	707.3562	707.3640	-10.92		1413.7052	1413.7206	CRagged	7	>sp|P0CE85|PAU19_YEAST Seripauperin-19 OS=Saccharomyces cerevisiae (strain ATC...	3420	12.7089	sample=1 peri

Proteins

	Prot. Rank	Protein Name	|Log Prob|	Best |Log Prob|	Best Score	# Spectra	# Uniq. Peps.	# Mod Peps.	% Cov.	# AAs	Intensity
☑	3 | 3	>sp|P0CE85|PAU19_YEAST Seripauperin-19 OS=Saccharomyces cerevisiae (strain ATCC 204508 / S288c) OX=5...	123.82	11.06	692.3	77	31	30	42.7	124	2.619e+6
☑	4 | 4	>sp|K2C1_HUMAN|(Common contaminant protein)	75.52	13.70	921.5	17	8	0	13.8	643	1.827e+5

Spectrum (double click to dock / undock)

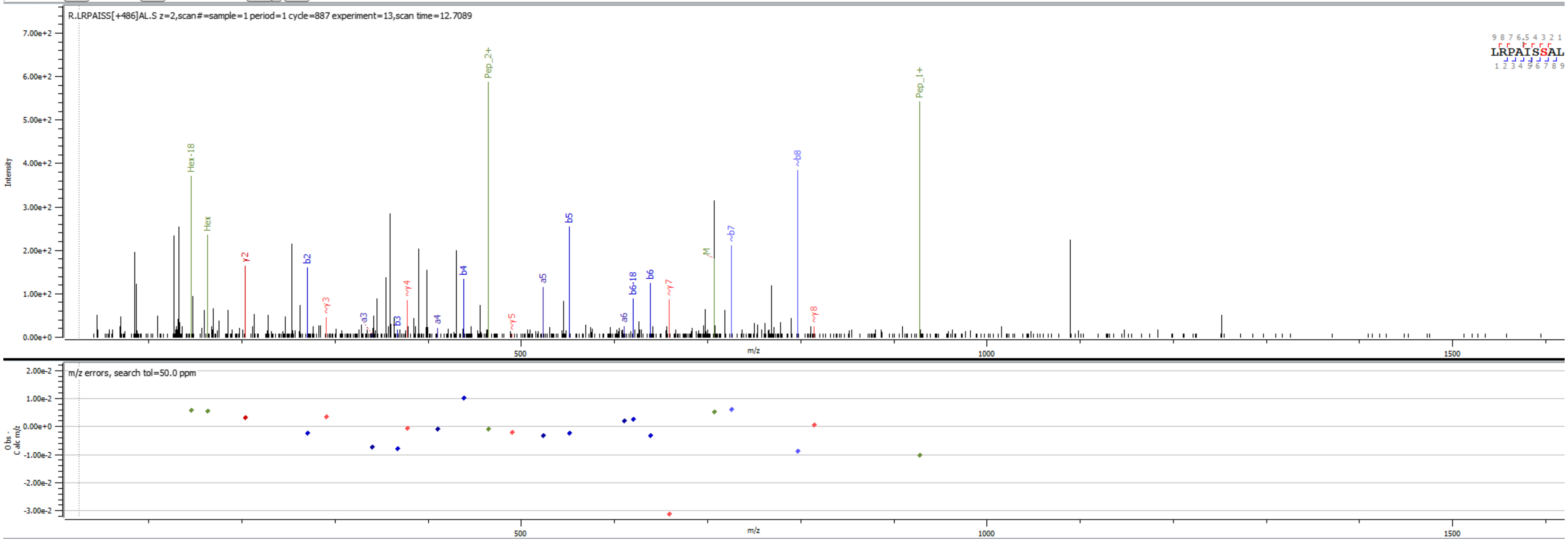

Sample S3 R.LRPAISS[+486.158]AL.S

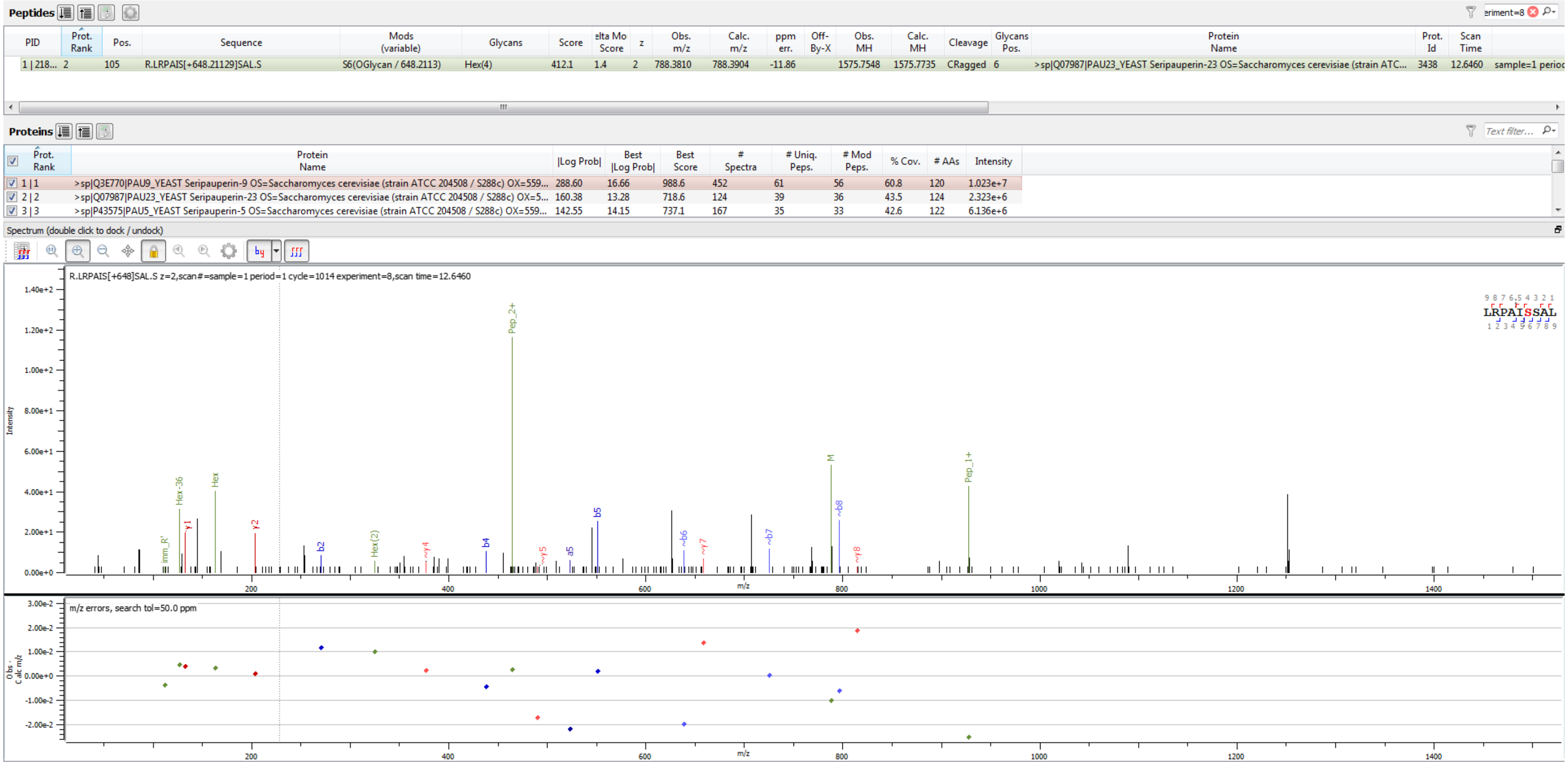

Sample S6 R.LRPAIS[+648.211]SAL.S

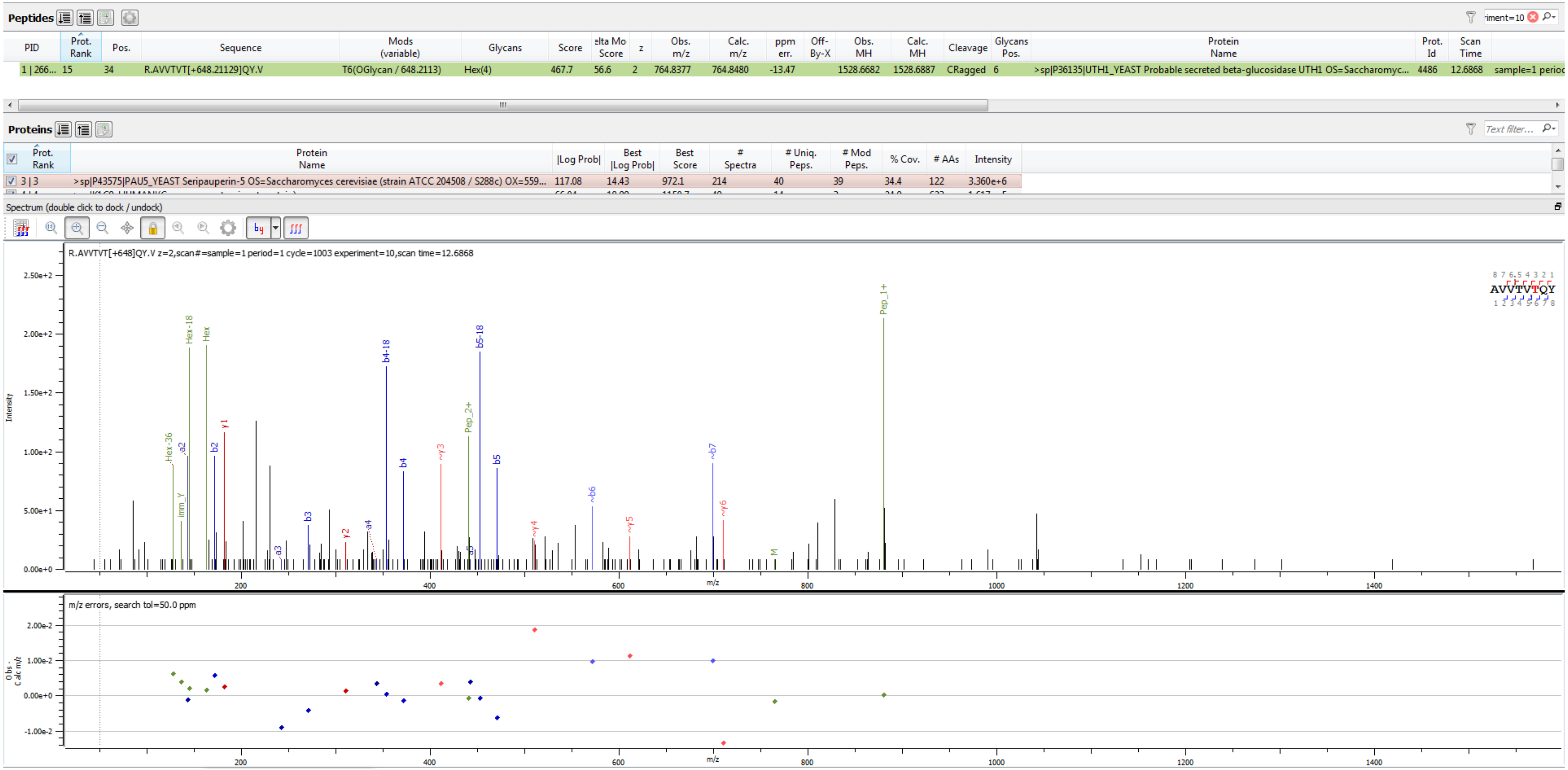

Sample S2 R.AVVTVT[+648.211]QY.V

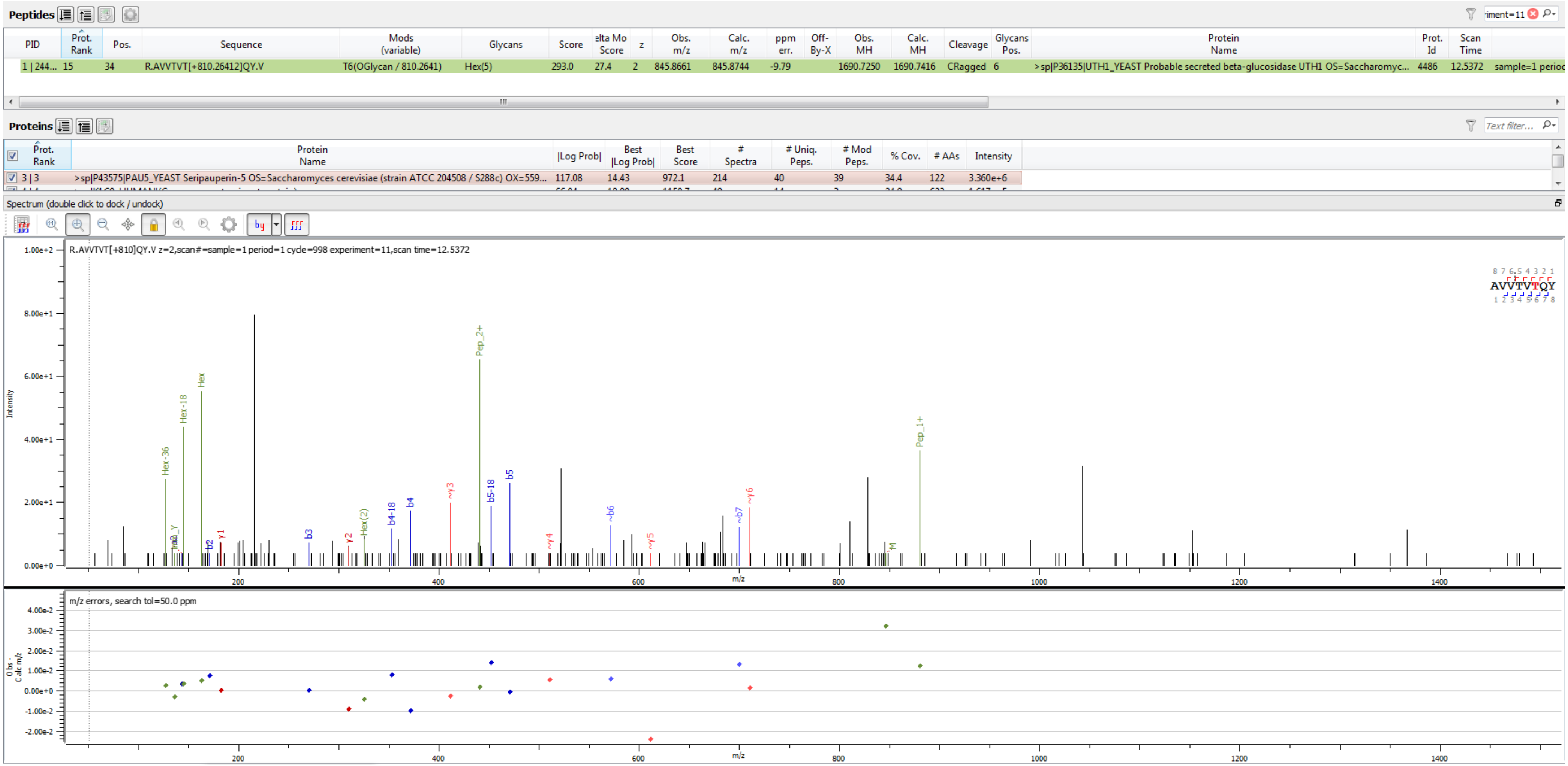

Sample S2 R.AVVTVT[+810.264]QY.V

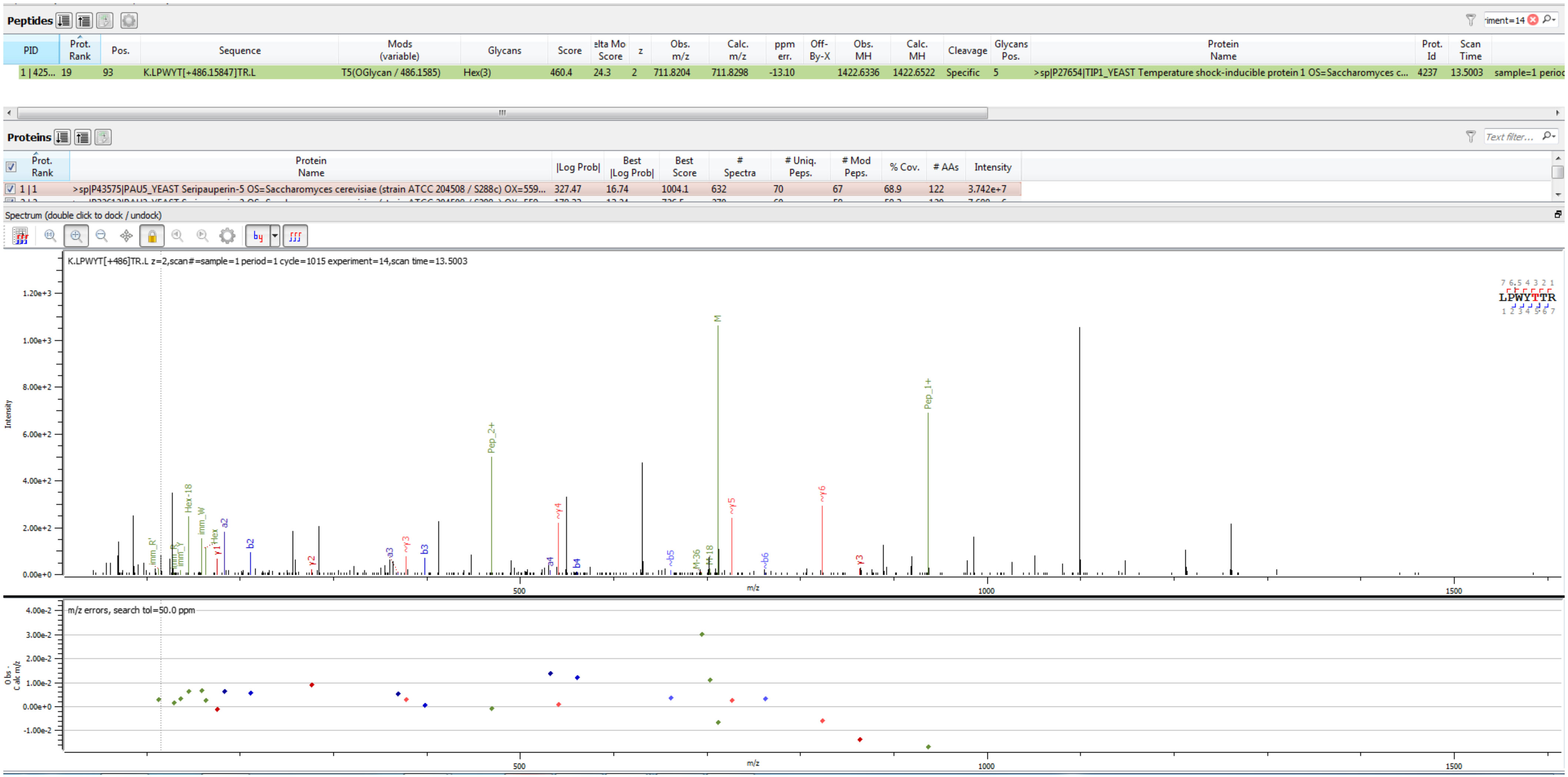

Sample S1 K.LPWYT[+486.158]TR.L

Peptides

PID	Prot. Rank	Pos.	Sequence	Mods (variable)	Glycans	Score	elta Mo Score	z	Obs. m/z	Calc. m/z	ppm err.	Off-By-X	Obs. MH	Calc. MH	Cleavage	Glycans Pos.	Protein Name	Prot. Id	Scan Time	
1 | 316...	22	93	K.LPWYT[+648.21129]TR.L	T5(OGlycan / 648.2113)	Hex(4)	408.8	1.0	2	792.8440	792.8562	-15.32		1584.6808	1584.7051	Specific	5	>sp|P27654|TIP1_YEAST Temperature shock-inducible protein 1 OS=Saccharomyces c...	4237	13.4180	sample=1 perio[illegible]

Proteins

Prot. Rank	Protein Name	|Log Prob|	Best |Log Prob|	Best Score	# Spectra	# Uniq. Peps.	# Mod Peps.	% Cov.	# AAs	Intensity
1 | 1	>sp|Q3E770|PAU9_YEAST Seripauperin-9 OS=Saccharomyces cerevisiae (strain ATCC 204508 / S288c) OX=559...	288.60	16.66	988.6	452	61	56	60.8	120	1.023e+7
2 | 2	>sp|Q07987|PAU23_YEAST Seripauperin-23 OS=Saccharomyces cerevisiae (strain ATCC 204508 / S288c) OX=5...	160.38	13.28	718.6	124	39	36	43.5	124	2.323e+6
3 | 3	>sp|P43575|PAU5_YEAST Seripauperin-5 OS=Saccharomyces cerevisiae (strain ATCC 204508 / S288c) OX=559...	142.55	14.15	737.1	167	35	33	42.6	122	6.136e+6

Spectrum (double click to dock / undock)

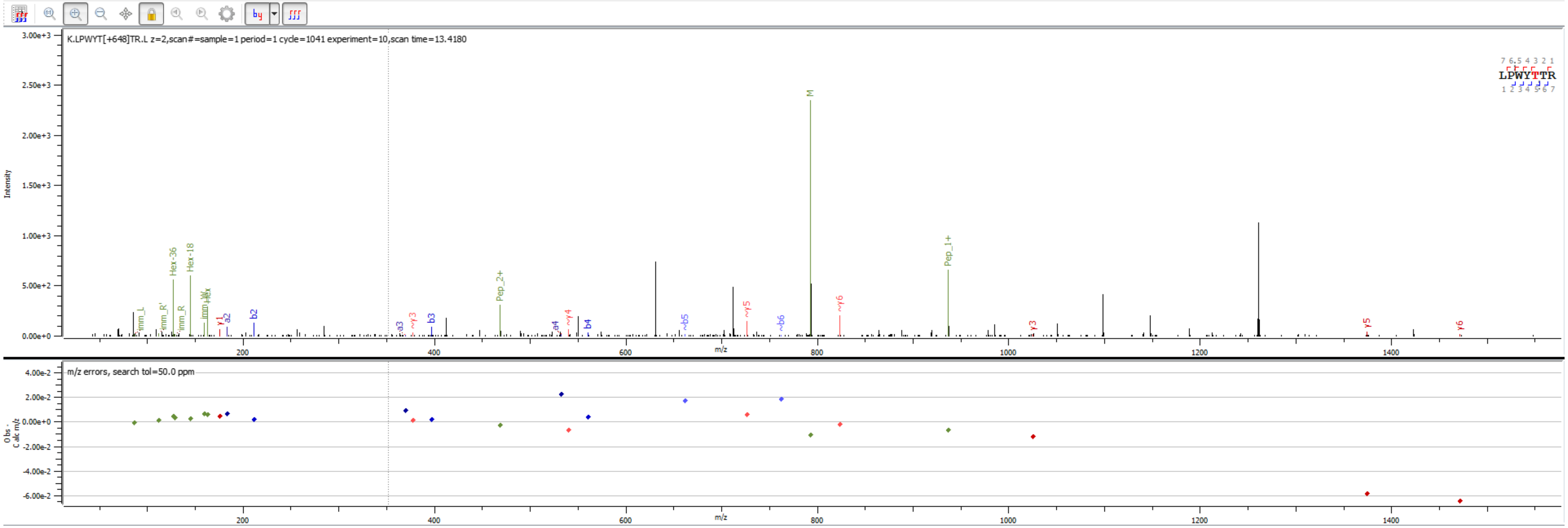

Sample S6 K.LPWYT[+648.211]TR.L

Peptides

PID	Prot. Rank	Pos.	Sequence	Mods (variable)	Glycans	Score	elta Mo Score	z	Obs. m/z	Calc. m/z	ppm err.	Off-By-X	Obs. MH	Calc. MH	Cleavage	Glycans Pos.	Protein Name	Prot. Id	Scan Time	
1 | 109...	21	33	R.VNLIELAVYVS[+324.10565]DIR.A	S11(OGlycan / 324.1056)	Hex(2)	552.7	316.1	2	964.4951	964.5091	-14.52		1927.9830	1928.0110	Specific	11	>sp|P47179|DAN4_YEAST Cell wall protein DAN4 OS=Saccharomyces cerevisiae (strain ...	273	19.6318	sample=1 perioc

eriment=7

Proteins

Text filter...

Prot. Rank	Protein Name	|Log Prob|	Best |Log Prob|	Best Score	# Spectra	# Uniq. Peps.	# Mod Peps.	% Cov.	# AAs	Intensity
1 | 1	>sp|Q3E770|PAU9_YEAST Seripauperin-9 OS=Saccharomyces cerevisiae (strain ATCC 204508 / S288c) OX=559...	288.60	16.66	988.6	452	61	56	60.8	120	1.023e+7
2 | 2	>sp|Q07987|PAU23_YEAST Seripauperin-23 OS=Saccharomyces cerevisiae (strain ATCC 204508 / S288c) OX=5...	160.38	13.28	718.6	124	39	36	43.5	124	2.323e+6
3 | 3	>sp|P43575|PAU5_YEAST Seripauperin-5 OS=Saccharomyces cerevisiae (strain ATCC 204508 / S288c) OX=559...	142.55	14.15	737.1	167	35	33	42.6	122	6.136e+6

Spectrum (double click to dock / undock)

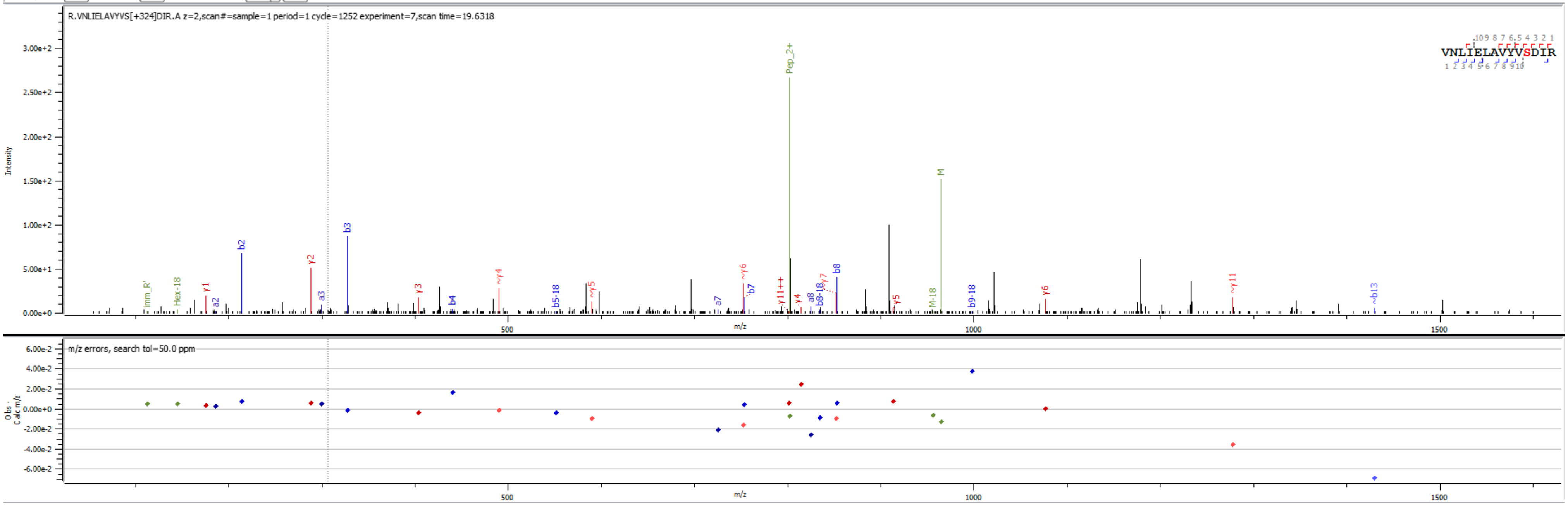

Sample S6 R.VNLIELAVYVS[+324.106]DIR.A

Peptides

PID	Prot. Rank	Pos.	Sequence	Mods (variable)	Glycans	Score	elta Mo Score	z	Obs. m/z	Calc. m/z	ppm err.	Off-By-X	Obs. MH	Calc. MH	Cleavage	Glycans Pos.	Protein Name	Prot. Id	Scan Time	
1 | 109...	21	33	R.VNLIELAVYVS[+324.10565]DIR.A	S11(OGlycan / 324.1056)	Hex(2)	448.7	266.1	3	643.3320	643.3419	-15.34		1927.9814	1928.0110	Specific	11	>sp|P47179|DAN4_YEAST Cell wall protein DAN4 OS=Saccharomyces cerevisiae (strain ...	273	19.6517	sample=1 perioc

Proteins

Prot. Rank	Protein Name	|Log Prob|	Best |Log Prob|	Best Score	# Spectra	# Uniq. Peps.	# Mod Peps.	% Cov.	# AAs	Intensity
1 | 1	>sp|Q3E770|PAU9_YEAST Seripauperin-9 OS=Saccharomyces cerevisiae (strain ATCC 204508 / S288c) OX=559...	288.60	16.66	988.6	452	61	56	60.8	120	1.023e+7
2 | 2	>sp|Q07987|PAU23_YEAST Seripauperin-23 OS=Saccharomyces cerevisiae (strain ATCC 204508 / S288c) OX=5...	160.38	13.28	718.6	124	39	36	43.5	124	2.323e+6
3 | 3	>sp|P43575|PAU5_YEAST Seripauperin-5 OS=Saccharomyces cerevisiae (strain ATCC 204508 / S288c) OX=559...	142.55	14.15	737.1	167	35	33	42.6	122	6.136e+6

Spectrum (double click to dock / undock)

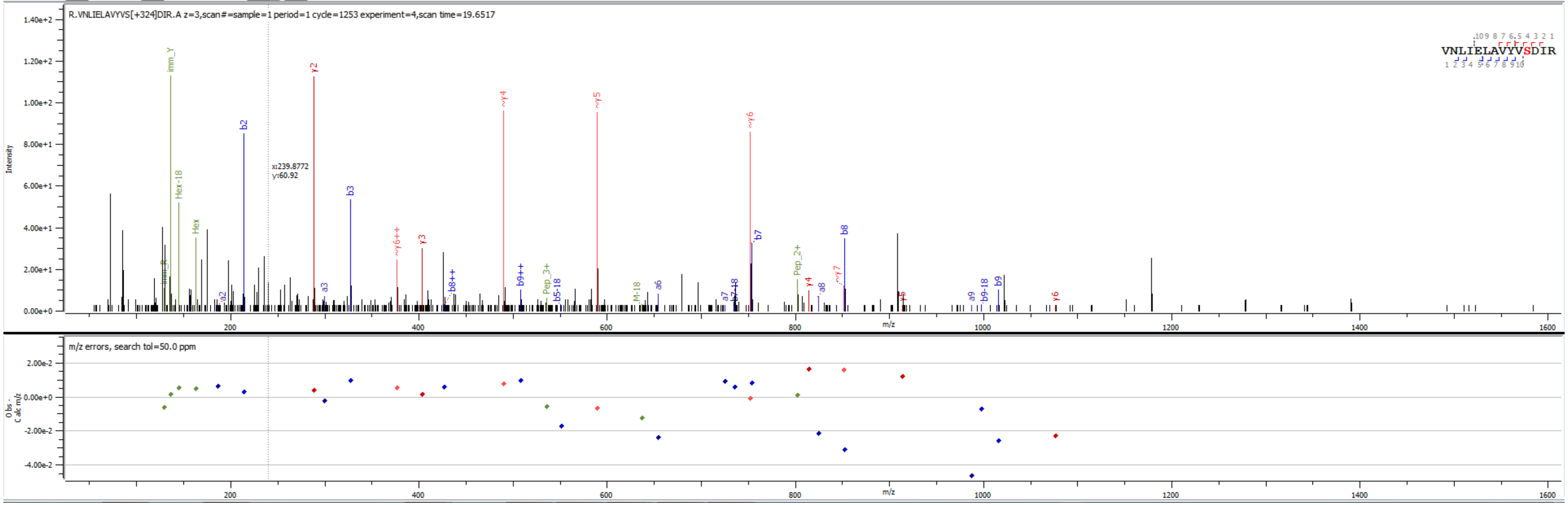

Sample S6 R.VNLIELAVYVS[+324.106]DIR.A

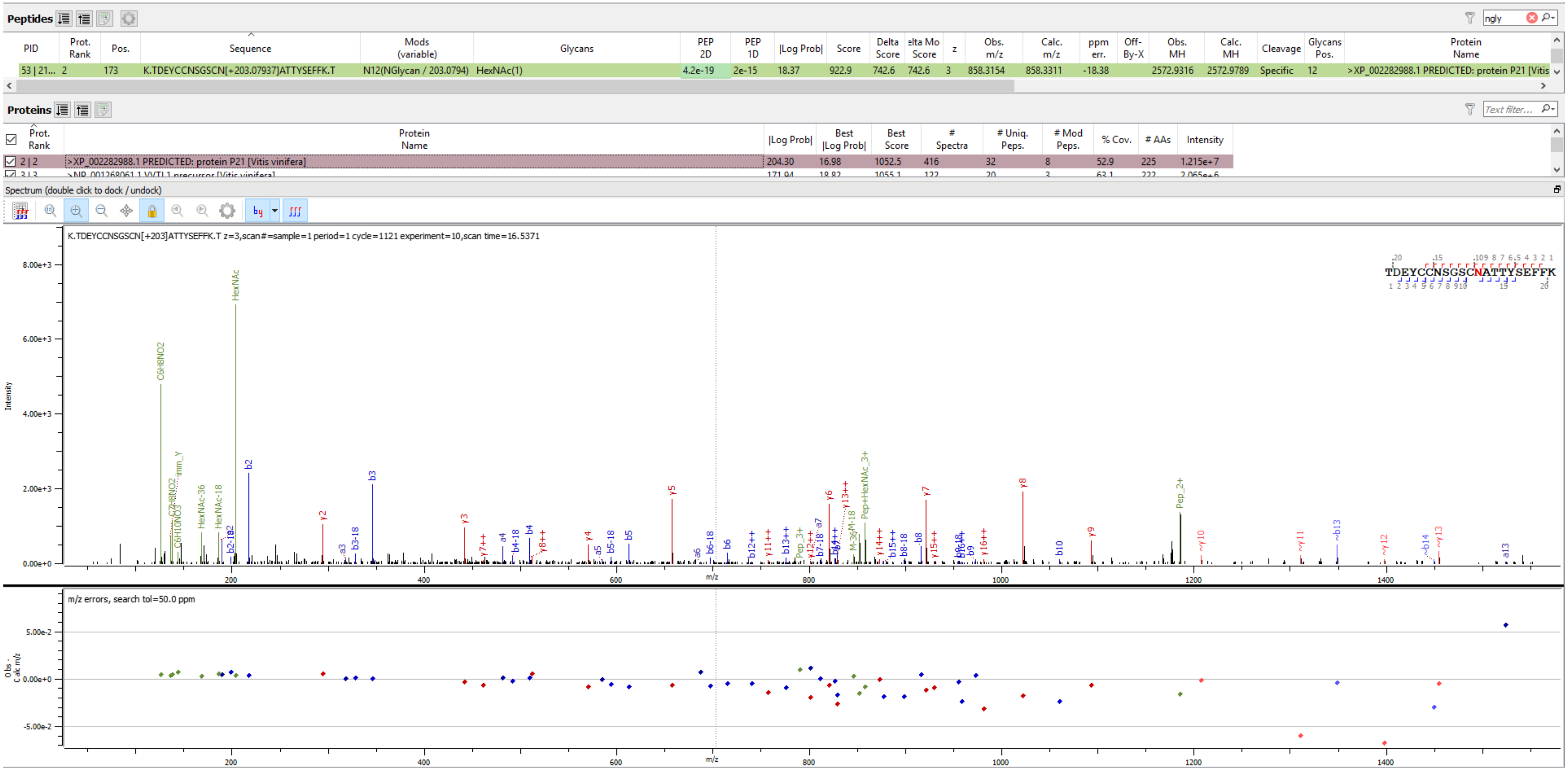

Peptides

PID	Prot. Rank	Pos.	Sequence	Mods (variable)	Glycans	PEP 2D	PEP 1D	|Log Prob|	Score	Delta Score	elta Mo Score	z	Obs. m/z	Calc. m/z	ppm err.	Off-By-X	Obs. MH	Calc. MH	Cleavage	Glycans Pos.	Protein Name
53 | 21...	2	173	K.TDEYCCNSGSCN[+203.07937]ATTYSEFFK.T	N12(NGlycan / 203.0794)	HexNAc(1)	4.2e-19	2e-15	18.37	922.9	742.6	742.6	3	858.3154	858.3311	-18.38		2572.9316	2572.9789	Specific	12	>XP_002282988.1 PREDICTED: protein P21 [Vitis

Proteins

Prot. Rank	Protein Name	|Log Prob|	Best |Log Prob|	Best Score	# Spectra	# Uniq. Peps.	# Mod Peps.	% Cov.	# AAs	Intensity
2 | 2	>XP_002282988.1 PREDICTED: protein P21 [Vitis vinifera]	204.30	16.98	1052.5	416	32	8	52.9	225	1.215e+7
3 | 3	>NP_001268061.1 VVTL1 precursor [Vitis vinifera]	171.94	18.82	1055.1	122	20	3	63.1	222	2.065e+6

Sample S1 K.TDEYCCNSGSCN[+203.079]ATTYSEFFK.T

Peptides

PID	Prot. Rank	Pos.	Sequence	Mods (variable)	Glycans	PEP 2D	PEP 1D	|Log Prob|	Score	Delta Score	elta Mo Score	z	Obs. m/z	Calc. m/z	ppm err.	Off-By-X	Obs. MH	Calc. MH	Cleavage	Glycans Pos.	Protein Name
32 | 24...	2	173	K.TDEYCCNSGSCN[+1170.41738]ATTYSEFFK.T	N12(NGlycan / 1170.41...	HexNAc(2)Hex(3)Fuc(1)Pent(1)	3.7e-19	1.7e-15	18.43	912.0	683.4	683.4	4	885.8177	885.8347	-19.17		3540.2490	3540.3169	Specific	12	>XP_002282988.1 PREDICTED: protein P21 [Vitis

Proteins

Prot. Rank	Protein Name	|Log Prob|	Best |Log Prob|	Best Score	# Spectra	# Uniq. Peps.	# Mod Peps.	% Cov.	# AAs	Intensity
2 | 2	>XP_002282988.1 PREDICTED: protein P21 [Vitis vinifera]	204.30	16.98	1052.5	416	32	8	52.9	225	1.215e+7

Spectrum (double click to dock / undock)

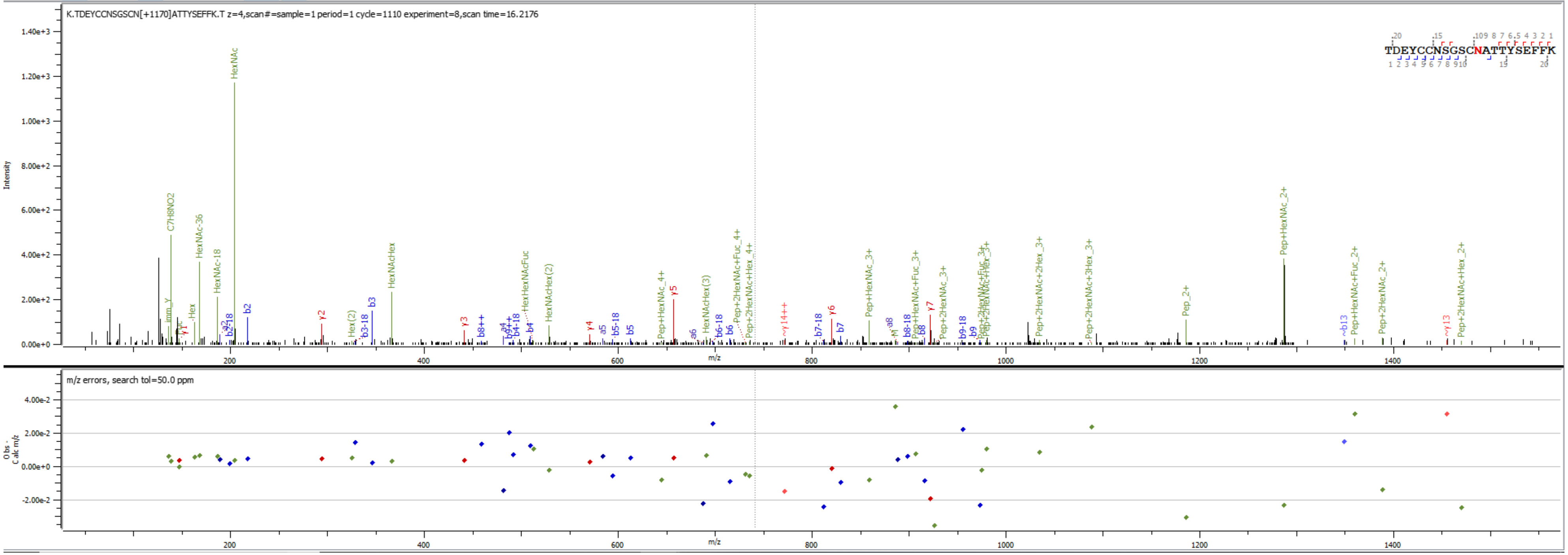

Sample S1 K.TDEYCCNSGSCN[+1170.417]ATTYSEFFK.T

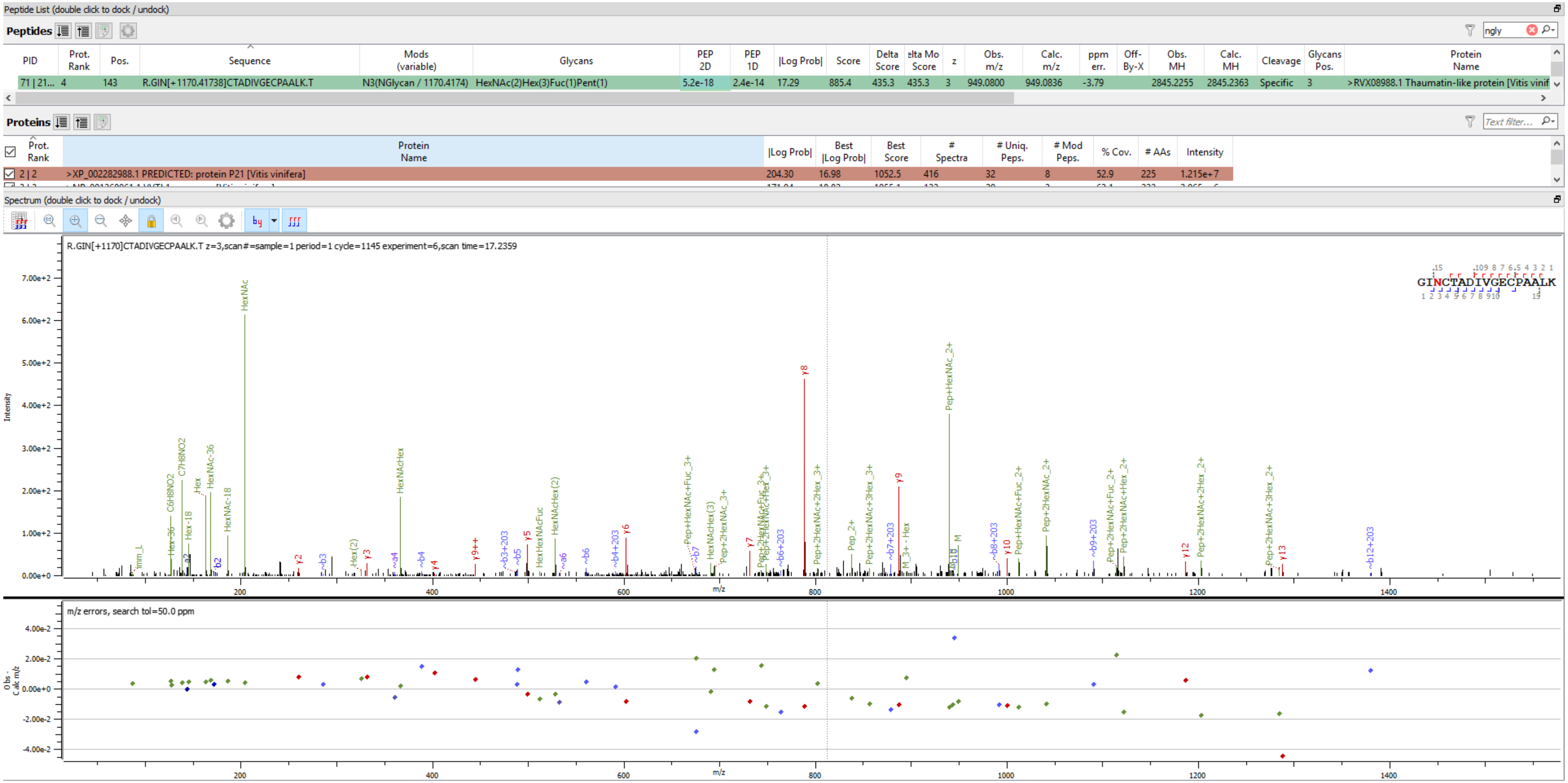

Sample S1 R.GIN[+1170.417]CTADIVGECPAALK.T

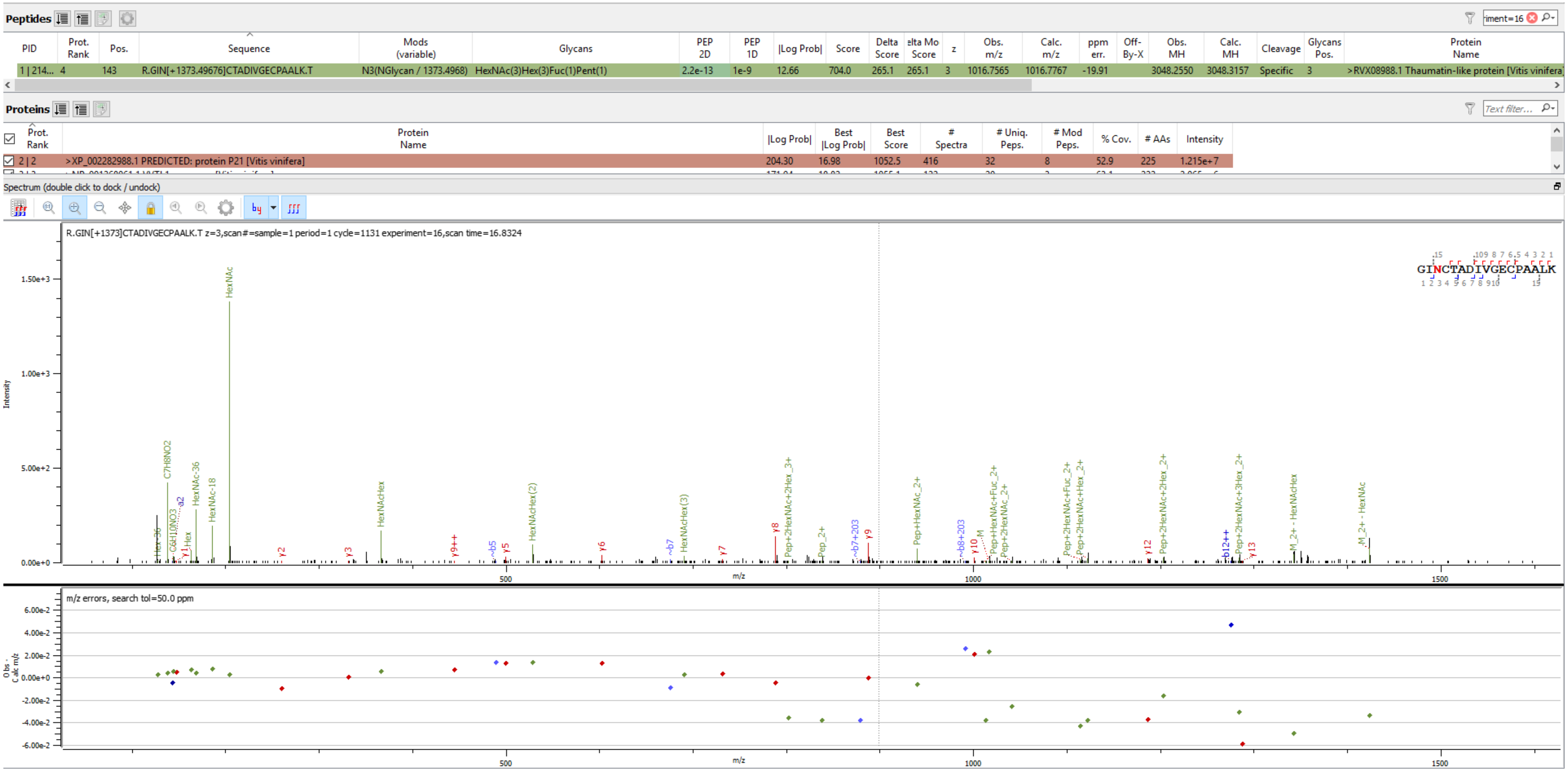

Sample S1 R.GIN[+1373.497]CTADIVGECPAALK.T

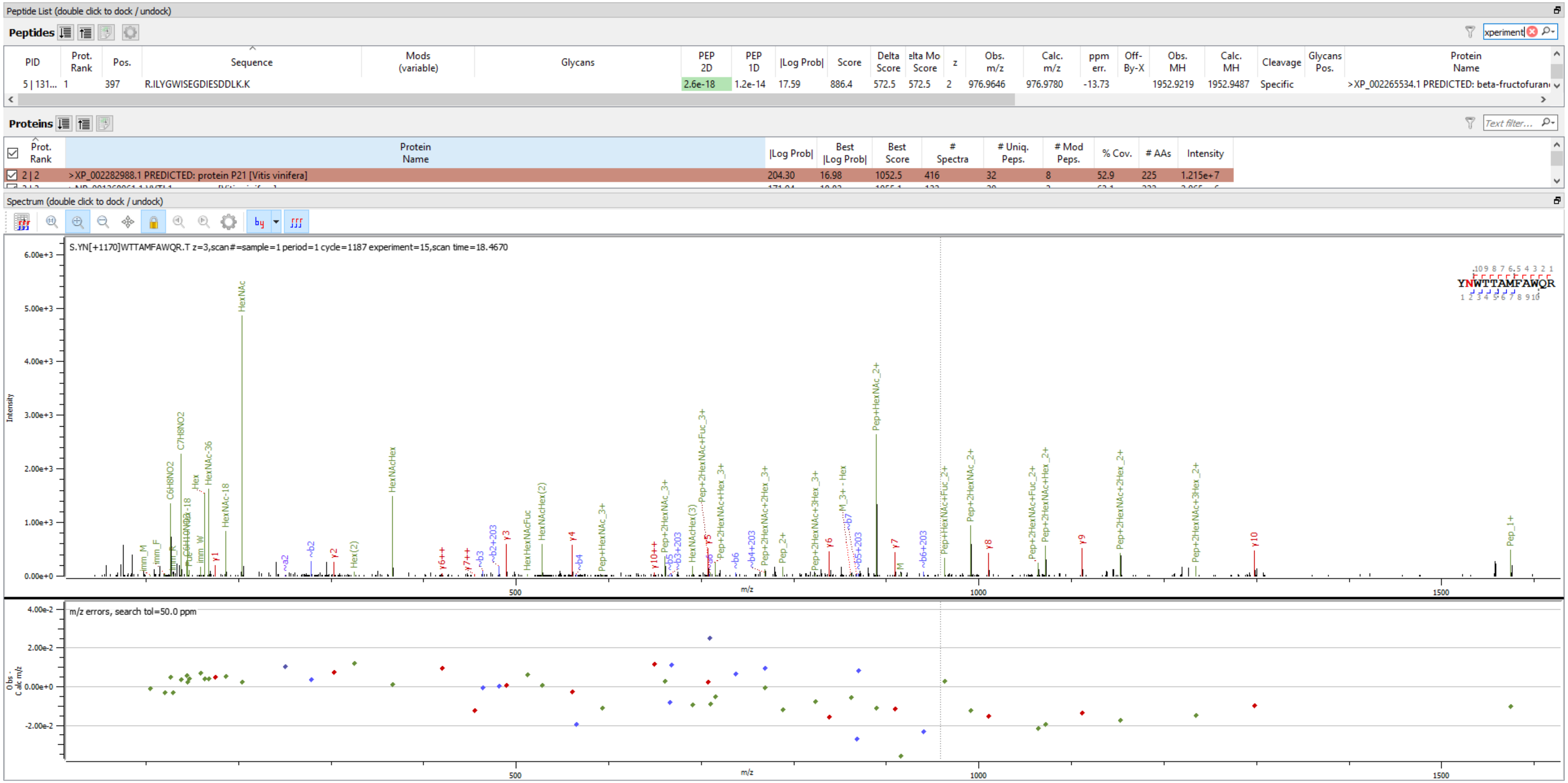

Sample S1 S.YN[+1170.417]WTTAMFAWQR.T

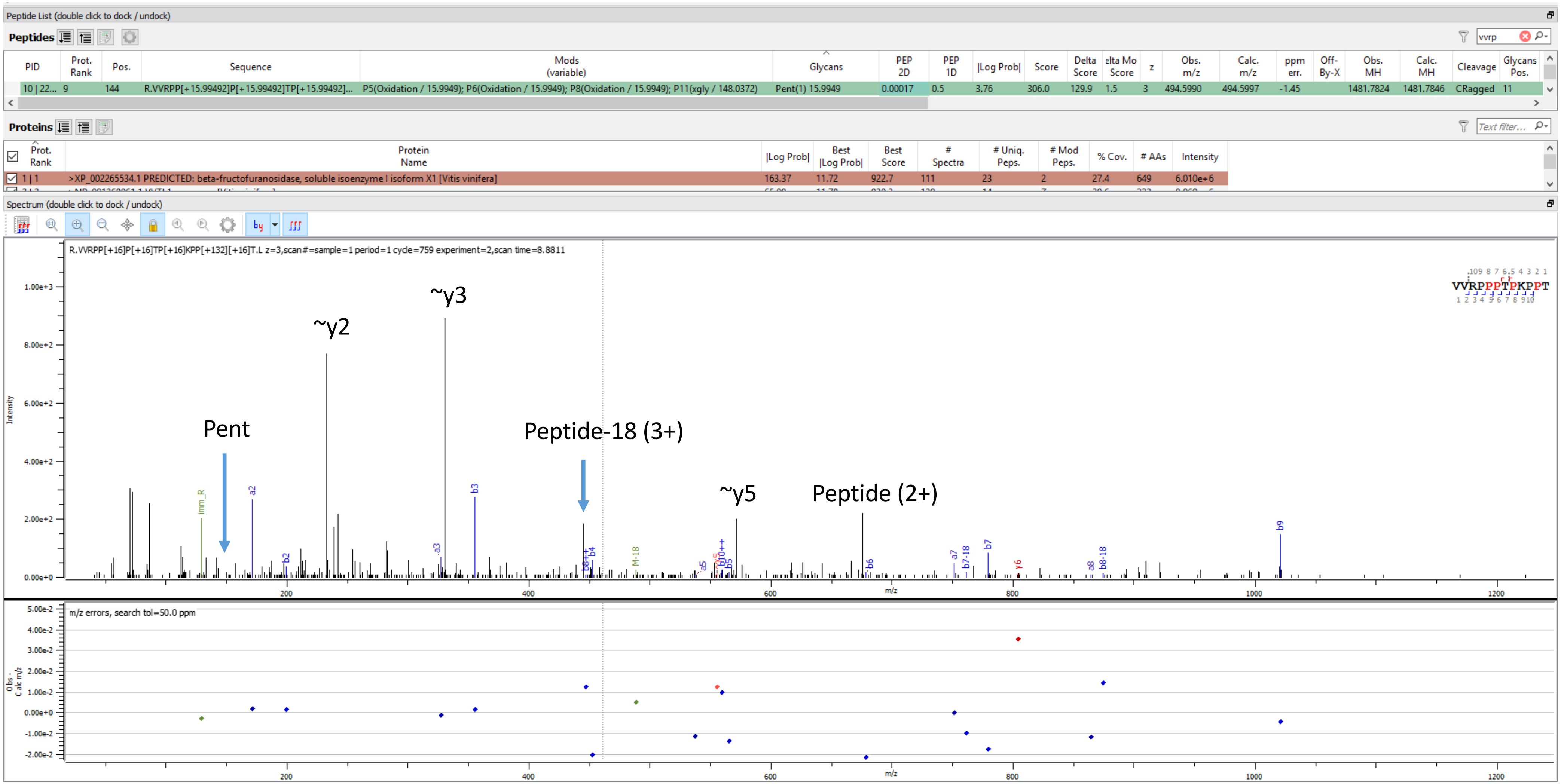

Sample S3 R.VVRPP[+15.995]P[+15.995]TP[+15.995]KPP[+132.042][+15.995]T.L

Peptides

PID	Prot. Rank	Pos.	Sequence	Mods (variable)	Glycans	PEP 2D	PEP 1D	|Log Prob|	Score	Delta Score	elta Mo Score	z	Obs. m/z	Calc. m/z	ppm err.	Off-By-X	Obs. MH	Calc. MH	Cleavage	Glycans Pos.	Protein Name	Prot. Id	Sc Ti
36 | 37...	7	144	R.VVRPP[+15.99492]P[+15.99492]TP[+15.99492]...	P5(Oxidation / 15.9949)...	Pent(2) 15.9949	0.011	0.99	1.97	196.6	63.5	8.6	3	538.6098	538.6138	-7.47		1613.8148	1613.8268	CRagged	11	>NP_001268136.1 proline-rich cell wall protein-like prec...	414	9.66

Proteins

Prot. Rank	Protein Name	|Log Prob|	Best |Log Prob|	Best Score	# Spectra	# Uniq. Peps.	# Mod Peps.	% Cov.	# AAs	Intensity
1 | 2	>NP_001268061.1 VVTL1 precursor [Vitis vinifera]	70.42	12.34	951.5	133	11	2	39.2	222	8.075e+6
2 | 4	>NP_001268074.1 lipid transfer protein precursor [Vitis vinifera]	31.89	13.13	997.2	62	8	1	35.3	119	3.655e+6

Spectrum (double click to dock / undock)

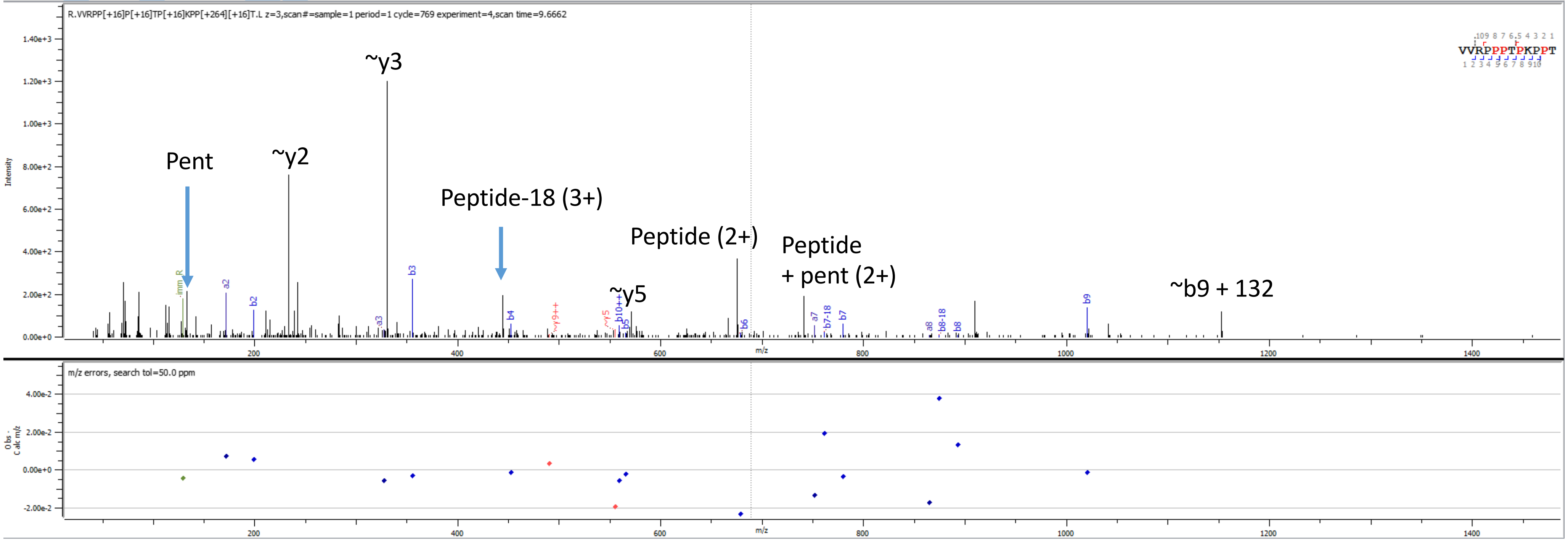

Sample S4 R.VVRPP[+15.995]P[+15.995]TP[+15.995]KPP[+264.085][+15.995]T.L

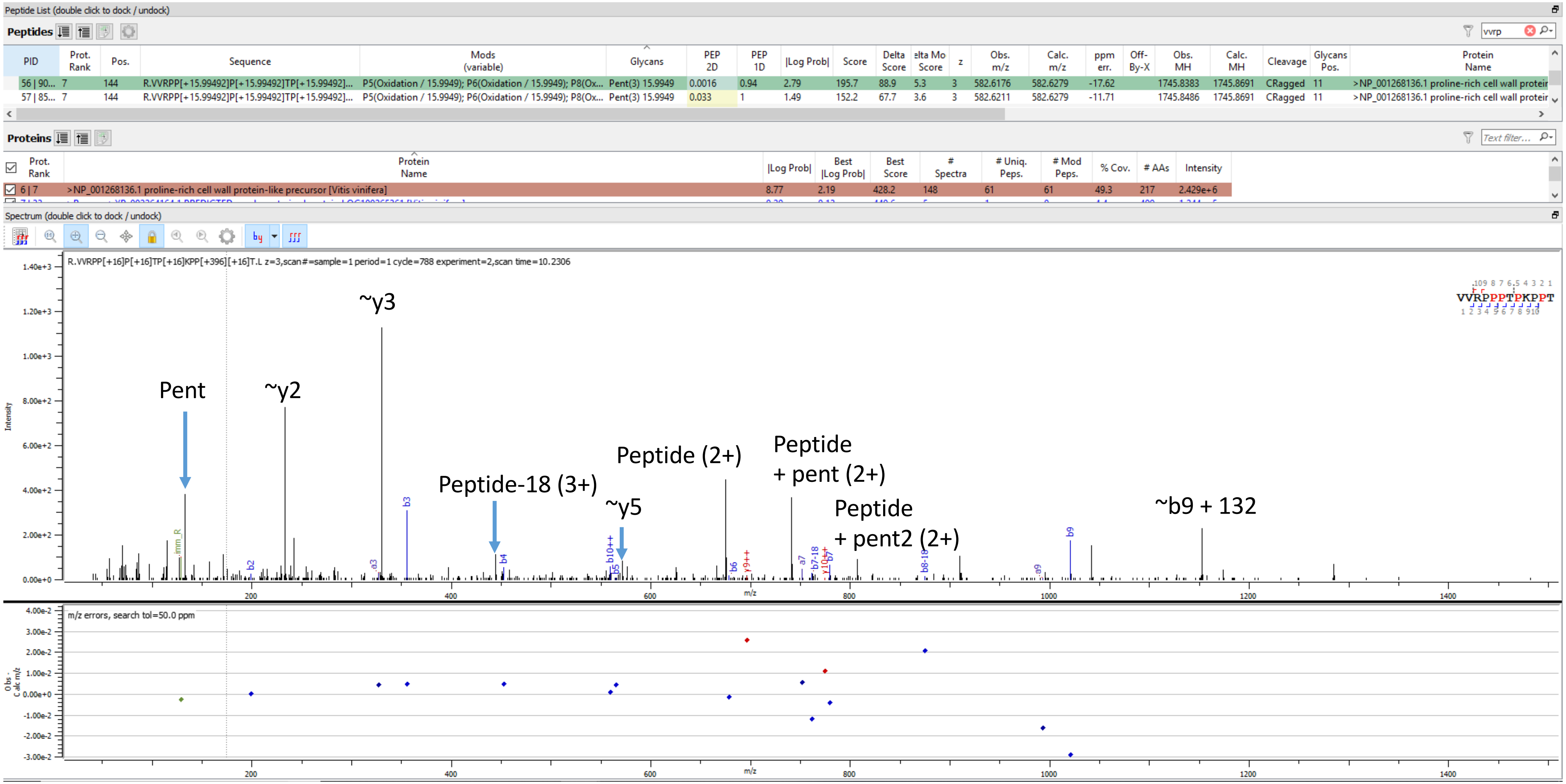

Sample S4 R.VVRPP[+15.995]P[+15.995]TP[+15.995]KPP[+396.127][+15.995]T.L

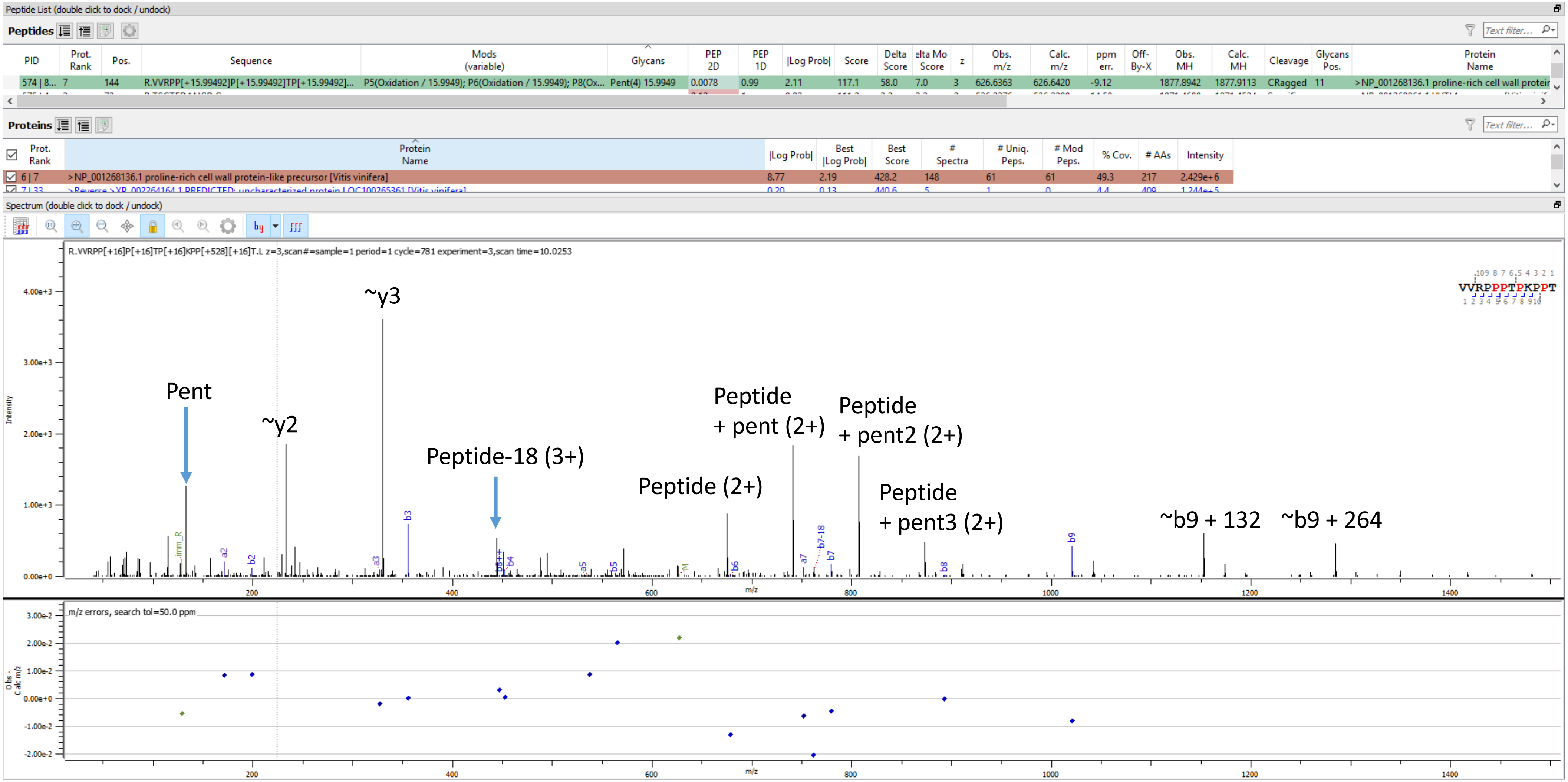

Sample S4 R.VVRPP[+15.995]P[+15.995]TP[+15.995]KPP[+528.169][+15.995]T.L

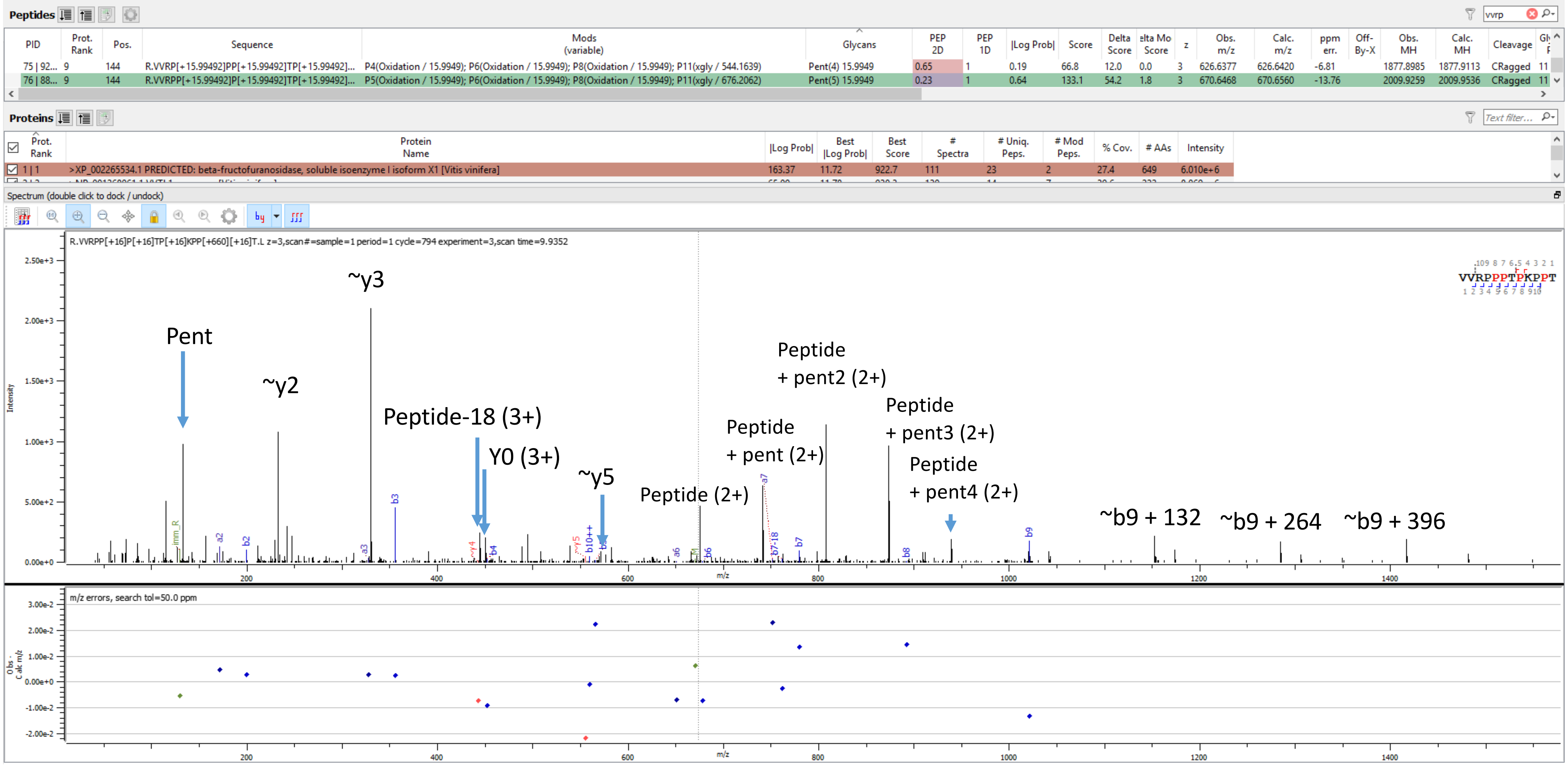

Sample S3 R.VVRPP[+15.995]P[+15.995]TP[+15.995]KPP[+660.211][+15.995]T.L

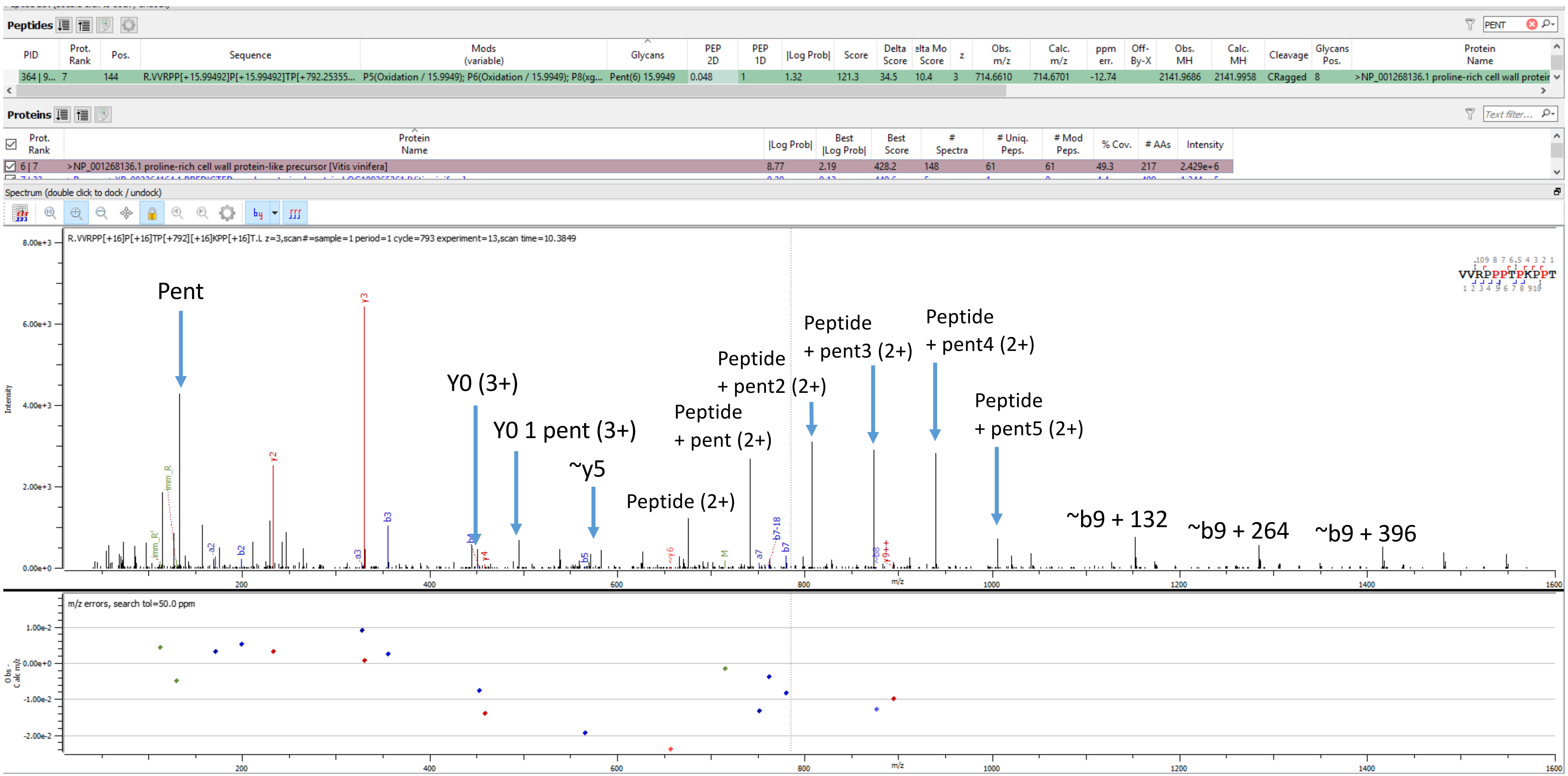

Sample S4 R.VVRPP[+15.995]P[+15.995]TP[+792.254][+15.995]KPP[+15.995]T.L
