# Supplementary material for: Quantitative Data-Independent Acquisition Glycoproteomics of Sparkling Wine
Source: Mol Cell Proteomics. 2020 Dec 21;20:100020. doi: 10.1074/mcp.RA120.002181 (PMC8724608; doi:10.1074/mcp.RA120.002181)
Supplement: MCPRO_MCP/2020/002181_2 [file mmc2.pdf]

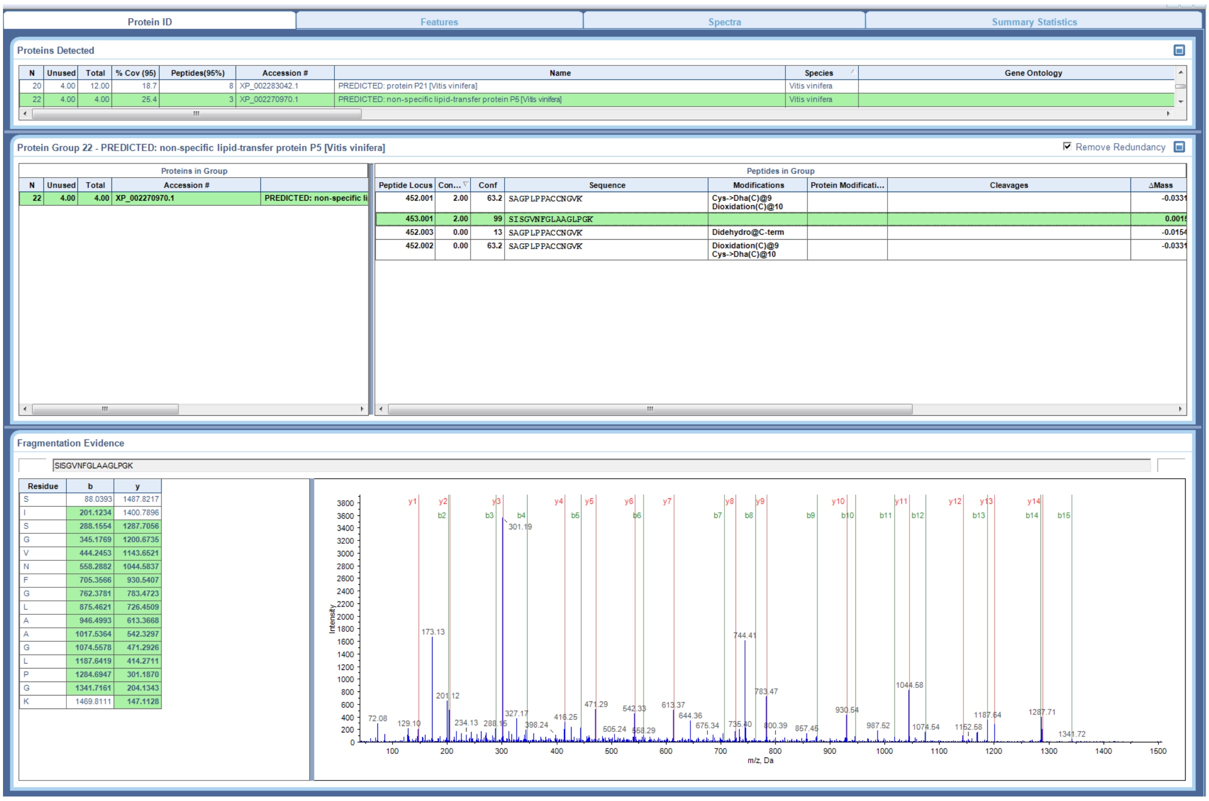

Supplementary Figure S2a. MS/MS of SISGVNFGLAAGLPGK identifying XP_002270970.1 PREDICTED: non-specific lipid-transfer protein P5 [Vitis vinifera].

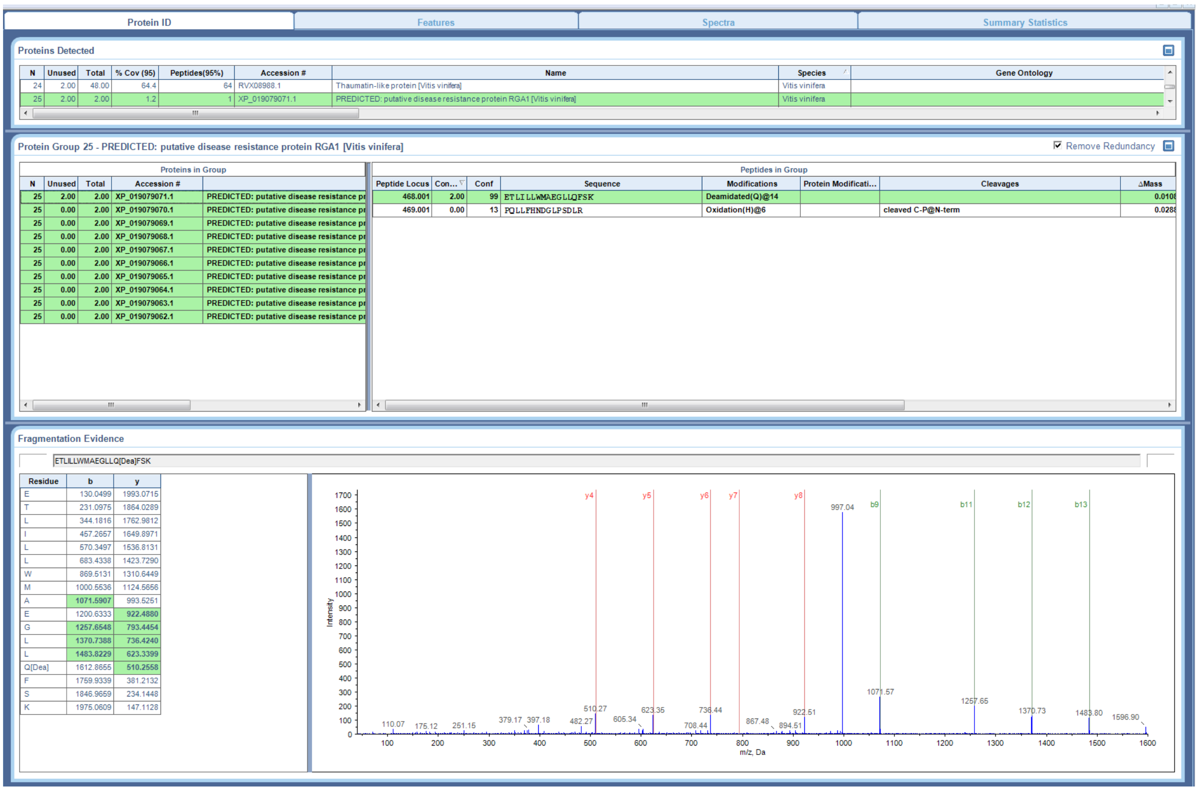

Supplementary Figure S2b. MS/MS of ETLILLWMAEGLLQFSK identifying XP_019079071.1 PREDICTED: putative disease resistance protein RGA1 [Vitis vinifera].

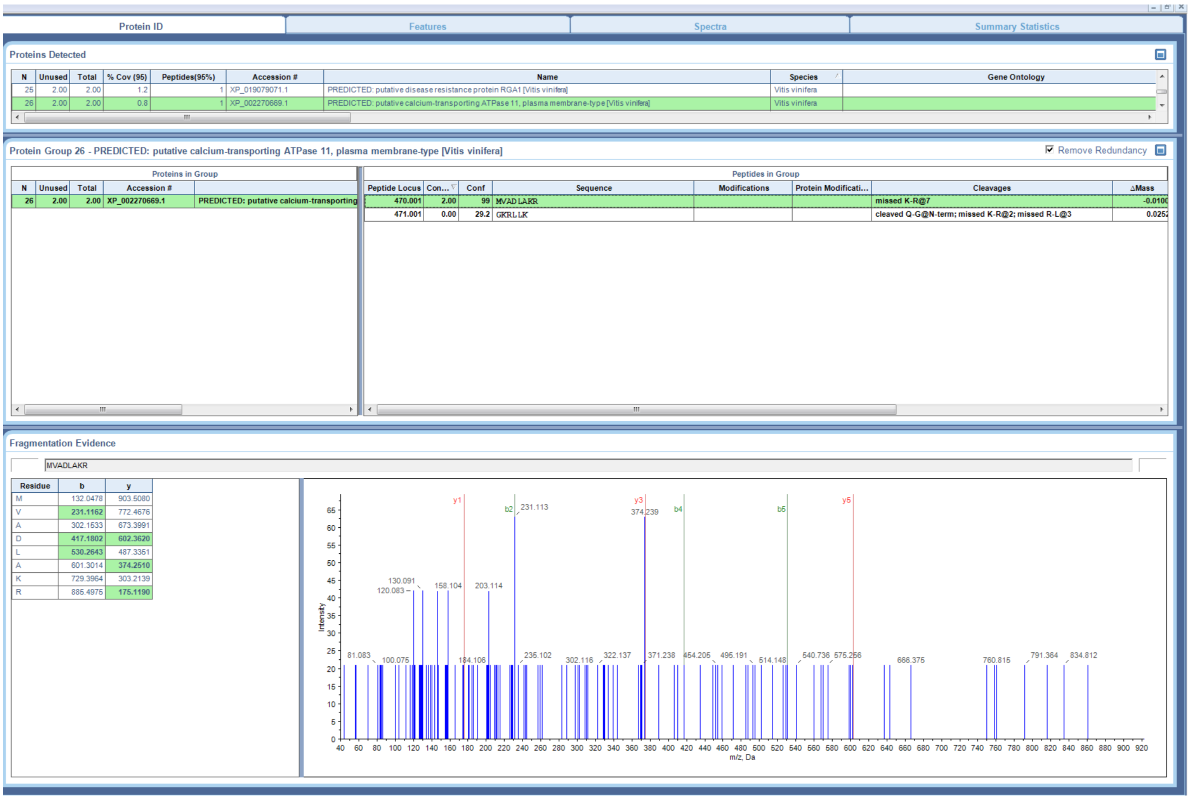

Supplementary Figure S2c. MS/MS of MVADLAKR identifying XP_002270669.1 PREDICTED: putative calcium-transporting ATPase 11, plasma membrane-type [Vitis vinifera].

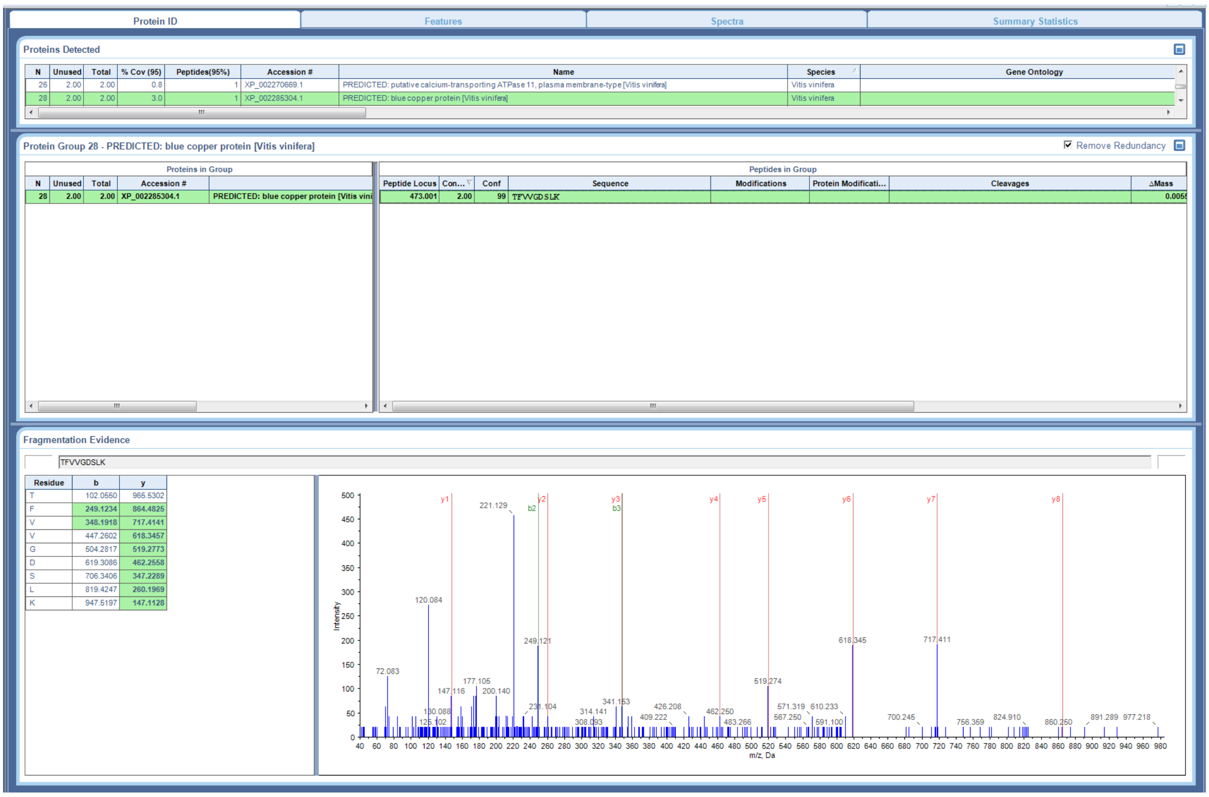

Supplementary Figure S2d. MS/MS of TFVVGDSLK identifying XP_002285304.1 PREDICTED: blue copper protein [Vitis vinifera].

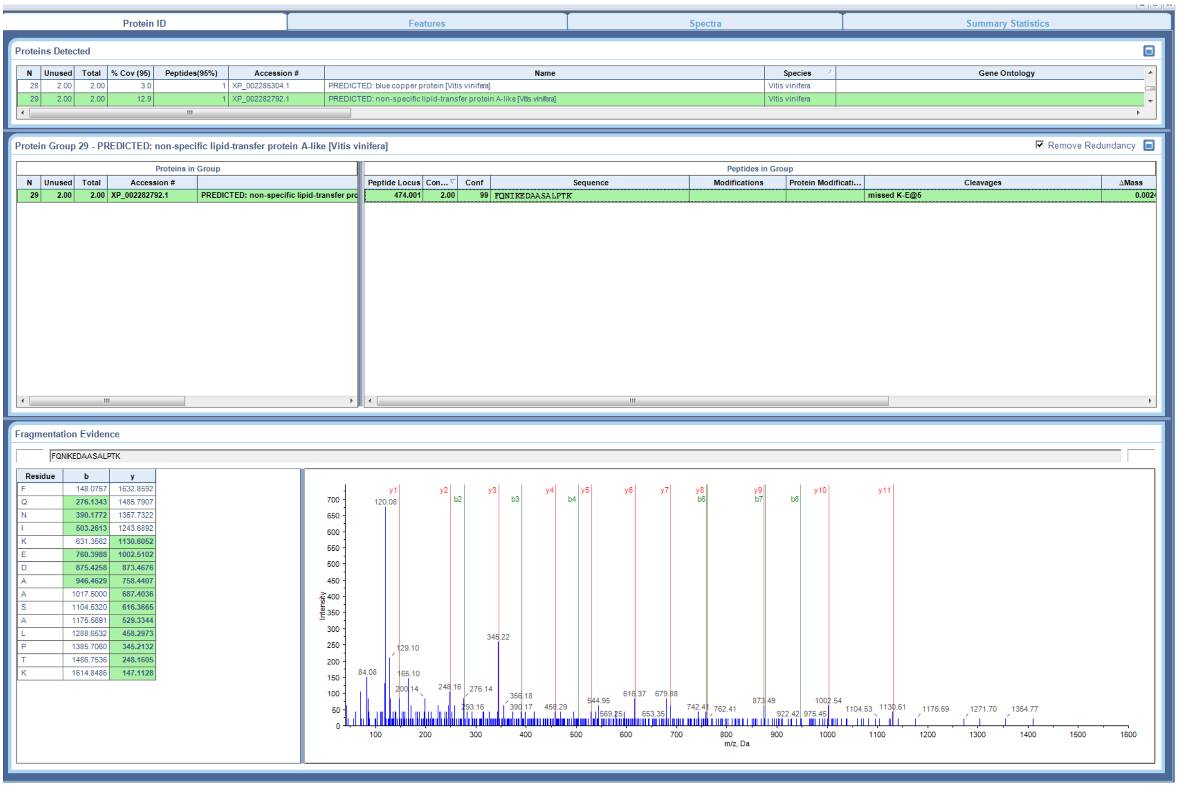

Supplementary Figure S2e. MS/MS of FQNIKEDAASALPTK identifying XP_002282792.1 PREDICTED: non-specific lipid-transfer protein A-like [Vitis vinifera].

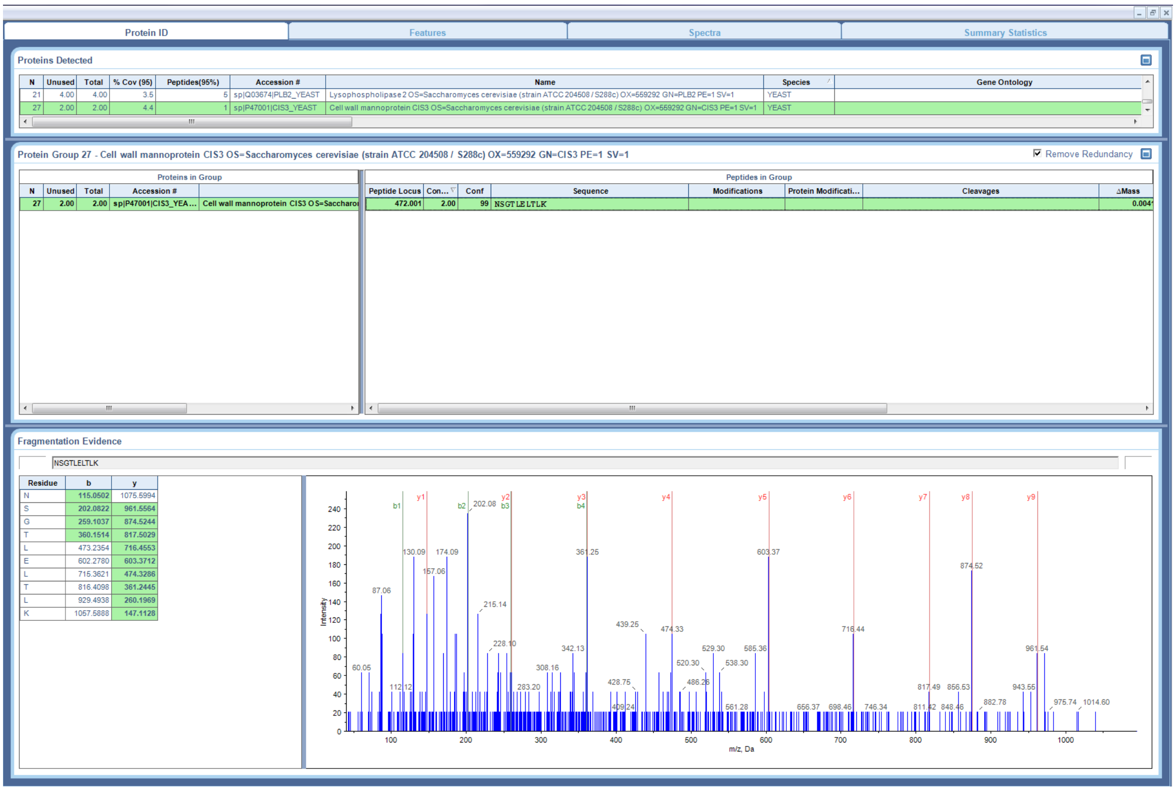

Supplementary Figure S2f. MS/MS of NSGTLELTLK identifying sp|P47001|CIS3_YEAST Cell wall mannoprotein CIS3 OS=Saccharomyces cerevisiae (strain ATCC 204508 / S288c) OX=559292 GN=CIS3 PE=1 SV=1.
